# Epigenetic transgenerational inheritance of somatic transcriptomes and epigenetic control regions

**DOI:** 10.1186/gb-2012-13-10-r91

**Published:** 2012-10-03

**Authors:** Michael K Skinner, Manikkam Mohan, Md M Haque, Bin Zhang, Marina I Savenkova

**Affiliations:** 1 Center for Reproductive Biology, School of Biological Sciences, Washington State University, Pullman, WA 99164-4236, USA; 2 Department of Genetics and Genomic Sciences, Institute of Genomics and Multiscale Biology, Mount Sinai School of Medicine, New York, NY 10029, USA

## Abstract

**Background:**

Environmentally induced epigenetic transgenerational inheritance of adult onset disease involves a variety of phenotypic changes, suggesting a general alteration in genome activity.

**Results:**

Investigation of different tissue transcriptomes in male and female F3 generation vinclozolin versus control lineage rats demonstrated all tissues examined had transgenerational transcriptomes. The microarrays from 11 different tissues were compared with a gene bionetwork analysis. Although each tissue transgenerational transcriptome was unique, common cellular pathways and processes were identified between the tissues. A cluster analysis identified gene modules with coordinated gene expression and each had unique gene networks regulating tissue-specific gene expression and function. A large number of statistically significant over-represented clusters of genes were identified in the genome for both males and females. These gene clusters ranged from 2-5 megabases in size, and a number of them corresponded to the epimutations previously identified in sperm that transmit the epigenetic transgenerational inheritance of disease phenotypes.

**Conclusions:**

Combined observations demonstrate that all tissues derived from the epigenetically altered germ line develop transgenerational transcriptomes unique to the tissue, but common epigenetic control regions in the genome may coordinately regulate these tissue-specific transcriptomes. This systems biology approach provides insight into the molecular mechanisms involved in the epigenetic transgenerational inheritance of a variety of adult onset disease phenotypes.

## Background

Epigenetic transgenerational inheritance involves the germ line transmission of epigenetic marks between generations that alter genome activity and phenotype [1-3]. Environmental factors (for example, toxicants or nutrition) at a critical time during fetal gonadal sex-determination have been shown to alter DNA methylation programming of the germ line to promote the presence of imprinted-like sites that can be transmitted through the sperm to subsequent generations [1,4]. Animals derived from a germ line with an altered epigenome have been shown to develop adult-onset disease or abnormalities such as spermatogenic cell defects, mammary tumors, prostate disease, kidney disease, immune abnormalities and ovarian defects [5-7]. The epigenetic transgenerational inheritance of such abnormal phenotypes has been shown to develop in F1 to F4 generations after environmental exposure of only an individual F0 generation gestating female [1]. Recently, we have found a variety of environmental toxicants (plastics, pesticides, dioxin (TCDD), hydrocarbons, and vinclozolin) can promote the epigenetic transgenerational inheritance of adult-onset disease phenotypes [8]. Similar observations of epigenetic transgenerational inheritance of altered phenotypes have been shown in worms [9], flies [10], plants [11], rodents [1,5] and humans [12]. Environmentally induced epigenetic transgenerational inheritance provides an additional mechanism to consider in disease etiology and areas of biology such as evolution [2,13]. The current study was designed to provide insights into how a male germ line with an altered epigenome can transmit a variety of altered disease states and phenotypes.

During migration down the genital ridge to colonize the fetal gonad, the primordial germ cells undergo an erasure of DNA methylation to allow a pluripotent state for the stem cell; then, at the onset of gonadal sex determination, DNA re-methylation is initiated in a sex-specific manner to generate the male or female germ line [2,14,15]. The germ line re-methylation is completed later in gonadal development. This developmental period in the mammal is the most sensitive to environmental insults for altering the epigenome (DNA methylation) of the male germ line [1,2,16]. After fertilization the paternal and maternal alleles are demethylated to, in part, develop the pluripotent state of the embryonic stem cells; re-methylation of these is then initiated at the blastula stage of embryonic development [2,14]. A set of imprinted genes escapes this de-methylation to allow a specific DNA methylation pattern to be maintained and transferred between generations [17,18]. The ability of environmentally induced epigenetic transgenerational inheritance to transmit specific epigenetic changes between generations suggests the germ line epimutations act similarly to imprinted-like sites that, although they undergo developmental programming, develop a permanently programmed DNA methylation pattern [2,4]. Observations suggest environmentally induced epigenetic transgenerational inheritance involves the development of programmed epimutations in the germ line (sperm) that then escape the de-methylation after fertilization to transmit an altered epigenome between generations.

After fertilization the gametes transmit their genetics and epigenetics into the developing embryo and subsequently to all somatic cell types derived from the embryo. The altered sperm epigenome can then promote a cascade of altered epigenetic and genetic transcriptome changes into the developing cell types and tissues [19]. Therefore, the speculation is that all cells and tissues will have an altered transcriptome. These altered transcriptomes would appear throughout development to generate an adult tissue or cell type with an altered differentiated state associated with this transgenerational transcriptome [16,19]. Previously, epigenetic transgenerational inheritance of an altered testis transcriptome [20] and ovarian granulosa cell transcriptome [7] has been observed. Although some tissues may be resistant to dramatic alterations in physiology due to these transcriptome changes, other tissues that are sensitive will have an increased susceptibility to develop disease [2,7,16,20]. The current study was designed to investigate the epigenetic transgenerational inheritance of transcriptomes in a variety of different tissues and investigate potential gene bionetworks involved.

Gene expression of a specific cell type or tissue goes through a continuous cascade of changes from a stem cell through development to a stable adult differentiated state [7]. Similarly, the epigenome goes through a cascade of developmental changes to reach a stable epigenome in the adult associated with specific cell types [19]. The genetic and epigenetic components interact throughout development to promote the developmental and subsequent adult state of differentiation [16]. The classic paradigm for the regulation of gene expression involves the ability to alter promoter activity to regulate the expression of the adjacent gene. The epigenome plays an important role in this mechanism through histone modifications that fine tune the expression of the adjacent gene [21]. In contrast to histones, DNA methylation can be distal and not correlated with promoter regions, yet appears to regulate genome activity [22,23]. Although major alterations in DNA methylation of promoters clearly can alter gene expression, distal regulatory sites also have an important role in gene regulation [22,24]. One of the best examples of such a mechanism involves imprinted genes such as *H19 *and *IGF2 *[17]. The DNA methylation region of the imprinted gene in the promoter of the adjacent gene regulates allele-specific gene regulation for a wide number of genes. An additional role for these epigenetic DNA methylation sites can also be to influence distal gene expression through an imprinting control region (ICR) [23].

The ICR for *IGF2 *and *H19 *[17,25] has been shown to act through long non-coding RNA (lncRNA) and distally for over a megabase in either direction to regulate the expression of multiple genes [26,27]. Therefore, an epigenetic DNA methylation region can regulate the expression of a number of distal genes [17,28]. Similar observations have also been made in plant systems [29,30]. The speculation is made that a large family of epigenetic sites will have the ability to regulate the expression of multiple genes distally. These regions we term 'epigenetic control regions' (ECRs). The ICR previously identified will likely be a subset of a larger family of such regions not required to have an imprinted gene characteristic, but use a variety of mechanisms from non-coding RNA to chromatin structural changes. The current study was designed to identify the potential presence of such ECRs in the epigenetic transgenerational inheritance model investigated. The existence of such ECRs can help explain how subtle changes in the epigenome may have dramatic effects on the transcriptome of a cell type or tissue.

Environmentally induced epigenetic transgenerational inheritance of adult-onset disease and phenotypic variation [2] involves the germ line transmission of an imprinted-like epigenome (for example, DNA methylation) [4] that subsequently affects the transcriptomes of all cell types and tissues throughout the life of the individual derived from that germ line. The current study identifies transgenerational transcriptomes in all the tissues investigated in both female and male progeny. A systems biology approach was used to investigate the molecular and cellular pathways and processes common to the epigenetic transgenerational inheritance of the tissue transcriptomes identified. Gene bionetwork analysis was used to identify underlying gene networks that may provide insight into the epigenetic control of the differential gene expression. Combined observations identified potential ECRs that help explain, in part, how a tissue-specific transgenerational transcriptome was generated and how a subtle alteration in the germ line epigenome may promote adult onset disease phenotypes.

## Results

### Transgenerational transcriptomes

**Figure 1:**
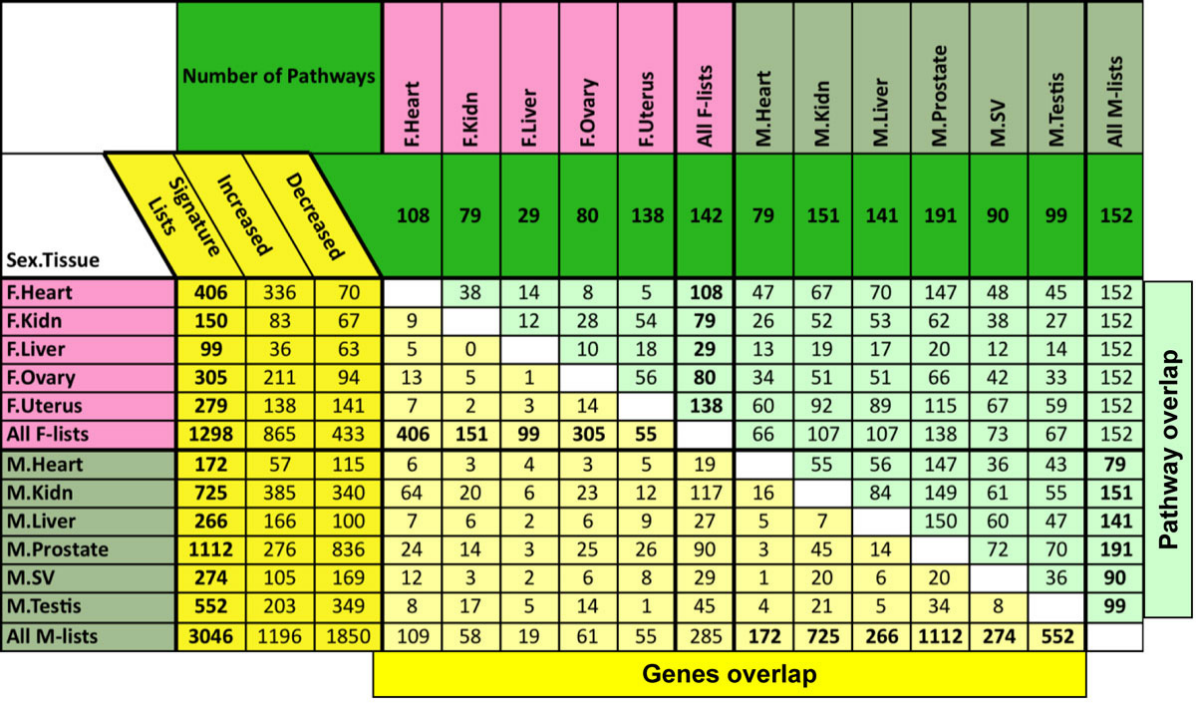
**Number of differentially expressed genes and pathways that overlap between signature lists**. The total number of genes or pathways for a signature list is shown in bold and only pathways with three or more affected genes are counted. F, female; M, male; SV, seminal vesicle.

The experimental design involved developing F3 generation Harlan Sprague Dawley rat control and vinclozolin lineage male and female adult animals as previously described [1,5]. The F0 generation gestating females were transiently exposed to vinclozolin or vehicle (DMSO) control during embryonic day 8 to 14 (E8 to E14) and then F1 generation offspring bred to produce the F2 generation followed by production of the F3 generation as described in the Materials and methods. No sibling or cousin breedings were used to avoid any inbreeding artifacts. Animals were aged to 4 months and then sacrificed to collect from males the testis, seminal vesicle, prostate, liver, kidney and heart; and from females the ovary, uterus, liver, kidney and heart. A total of six different control and six different vinclozolin F3 generation lineage animals, each one from different litters, were used and microarrays ran on each tissue using three pools of two animals each. A total of 66 microarrays were run on F3 generation control and vinclozolin lineage male and female rat tissues. The microarray data were obtained and compared for quality control as shown in Additional file 1. All microarrays within a tissue set compared well with no outliers, so all were used in subsequent data analysis. A comparison of control lineage and vinclozolin lineage tissues was made to identify the differentially expressed genes consistent between all animals and microarrays with a minimum of a 1.2-fold change in expression and mean difference of raw signal >10 as previously described [31]. As outlined in the Materials and methods, since a 20% alteration in gene expression can have cellular and biological impacts, particularly for transcription factors, the gene expression used a 1.2-fold cutoff that had a statistical difference rather than minimize the list with a more stringent cutoff value. The mean difference cutoff was used to eliminate background level signal expression changes. Differential gene expression with a statistical significance of *P *< 0.05 was used to identify the differentially expressed gene sets for each tissue; these are termed the 'signature list'. These less stringent criteria led to a relatively larger number of genes for the subsequent network analysis that can further filter out noisy signal using advanced soft thresholding techniques. The signature lists for all tissues are presented in Additional file 5 and genes categorized functionally. A summary of the signature list gene sets is presented in Figure 1.

**Figure 2:**
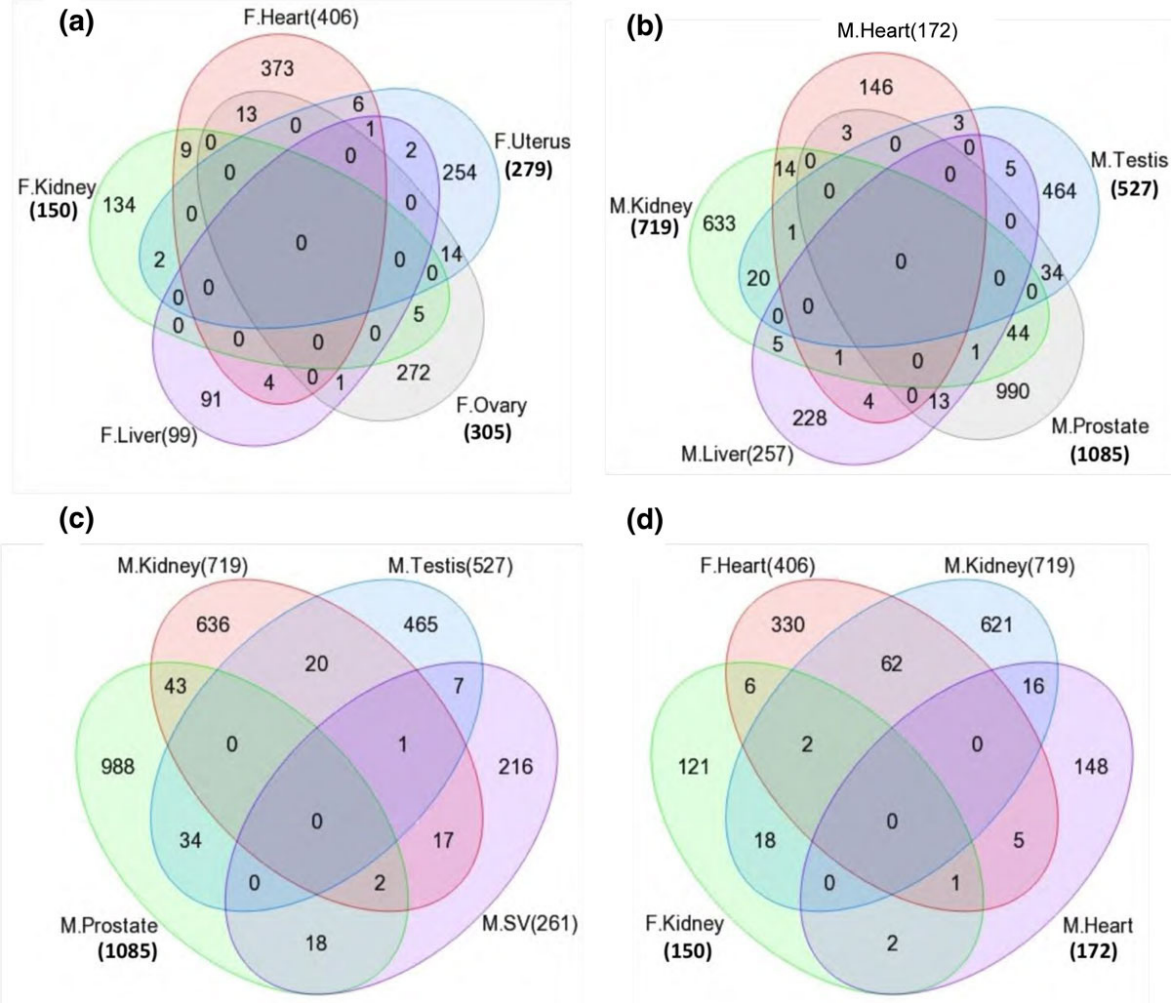
**Venn diagrams of male and female tissue signature lists of F3 generation vinclozolin lineage differentially expressed genes**. **(a) **Female (F) heart, kidney, liver, uterus, and ovary. **(b) **Male (M) heart, kidney, liver, testis, and prostate. **(c) **Male kidney, testis, seminal vesicle (SV), and prostate. **(d) **Female heart and kidney and male heart and kidney. Numbers in brackets are the total number of genes in the signature list.

The general overlap of genes between the tissues and between males and females is shown in Figure 1. These differentially expressed genes in the various tissues represent transgenerational transcriptomes in the F3 generation. No predominant overlap with large numbers of differentially exposed genes were found between the different tissues and between male and female lists (Figure 1). A specific comparison of genes between the tissues for male and female is presented in Figure 2. Venn diagrams show the majority of differentially expressed genes are tissue-specific with negligible overlap among all tissues. Therefore, each tissue had a predominantly unique transgenerational transcriptome and negligible overlap was observed between male and female tissues.

**Figure 3:**
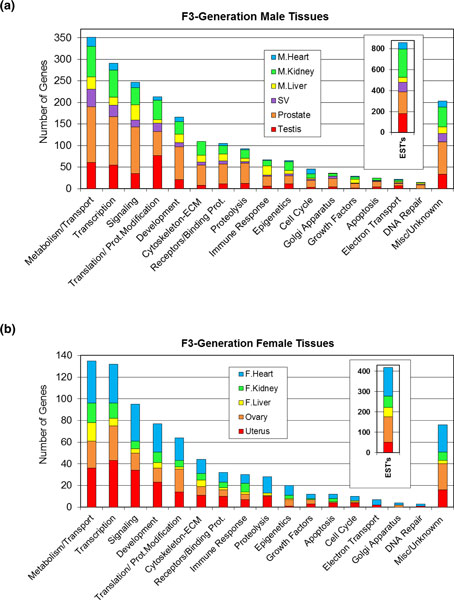
**Number of genes differentially expressed in F3 generation vinclozolin lineage tissues and their distribution among main functional categories**. **(a) **Male (M) heart, kidney, liver, testis, seminal vesicle (SV), and prostate. **(b) **Female (F) heart, kidney, liver, uterus, and ovary. ECM, extracellular matrix.

The specific differentially expressed genes were placed in Gene Ontology (GO) functional categories from Affimetrix annotations and similar trends were found among the different tissue signature lists and between the male and female lists. Therefore, no specific functional categories were predominant in any of the individual lists and no major differences exist. The categories are shown in Figure 3 for all tissues. Further analysis of specific cellular pathways and processes determined the number of genes associated with the various tissue signature lists. A list of those pathways containing the highest number of genes altered within the pathway or process for the top 30 is provided in Table 1. A more extensive list of differentially expressed genes correlating to specific pathways and processes is provided in Additional file 6. Observations demonstrate no predominant pathways or cellular processes were associated with the various signature lists. In contrast, a relatively large number of pathways and processes were influenced by all the tissue signature lists (Figure 1).

**Table 1:** Pathway enrichment for 11 male and female rat tissue signature lists

	11 male and female tissues	Sex and tissue												
		
		Male							Female					
			
		6 tissues	Heart	Kidney	Liver	Prostate	SV	Testis	5 tissues	Heart	Kidney	Liver	Ovary	Uterus
Pathway name														
Number of genes in input list	4059	3046	172	725	266	1112	274	552	1298	408	151	99	305	279
Total number of affected pathways	230	224	79	151	141	191	90	99	191	108	79	29	80	138
Pathways in cancer	45	32	3	7	6	13	1	2	17	6	3	1	3	4
Protein processing in endoplasmic reticulum	39	38	3	4	1	22	5	4	3	1			1	1
HTLV-I infection	39	31		8	3	14	3	2	10	4	2		1	3
RNA transport	39	31	1	8		17	3	6	11	4		1	2	4
Transcriptional misregulation in cancers	31	26	1	8	6	7	4	3	12	6	3	1		3
Herpes simplex infection	30	23	2	10	3	7	2	1	9	3	1		2	3
Lysosome	29	27	1	5	4	16	2	2	5	1			1	3
Ribosome	29	27	2	5		1		20	4	1	1	2		
Endocytosis	29	26	1	4	8	11	1	3	5	2				3
Phagosome	28	27	1	6	6	12		2	7		3			4
MAPK signaling pathway	28	21	1	7	3	7	1	2	8	3			1	4
Spliceosome	27	17		8	1	1	2	7	12	6	1		1	5
Regulation of actin cytoskeleton	26	24	1	6	6	9		3	5	3		1		1
Alzheimer's disease	26	22	2	4		7		10	5				2	3
Huntington's disease	26	22	1	6		2		14	5	1		1	2	1
Purine metabolism	26	22	1	6	2	4	2	6	7	2				5
Focal adhesion	26	21	2	2	7	8		3	9	3		2		4
Chemokine signaling pathway	24	23	3	4	6	9	1	1	4				2	2
Pyrimidine metabolism	24	20	1	6	1	6	1	5	6	1		1	1	3
Tuberculosis	24	20		6	6	9	1		9	1	1			7
Influenza A	23	21	1	5	2	11	1	3	5	1	2			2
Oxidative phosphorylation	23	20	1	4		5		11	5		1		1	3
Leukocyte transendothelial migration	22	18	2	1	7	8		1	6	4	1			1
Cytokine-cytokine receptor interaction	21	19	1	6	7	6			4	3				1
Osteoclast differentiation	21	18	1	2	6	10	1		5	1			1	3
Cell adhesion molecules	20	14	2	3	4	6	1		7	3	1		1	2
Insulin signaling pathway	19	13		2	3	6	2		6	2				4
mRNA surveillance pathway	19	13	1	4		5	2	4	8	4			2	2

HTLV, human T-lymphotropic virus; MAPK, mitogen-activated protein kinase; SV, seminal vesicle.

### Gene bionetwork analysis

**Figure 4:**
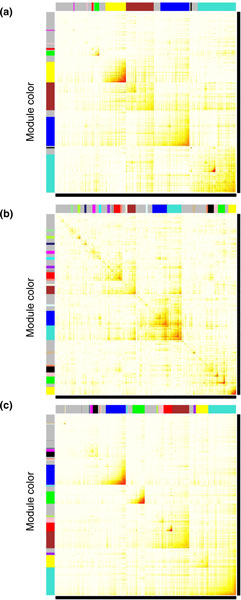
**Gene bionetwork cluster analysis of 11 male and female tissues with corresponding gene modules**. Topological overlap matrixes of the gene co-expression network consisting of genes differentially expressed in 11 tissues of the F3 vinclozolin lineage compared to F3 control lineage animals. Genes in the rows and columns are sorted by an agglomerative hierarchical clustering algorithm. The different shades of color signify the strength of the connections between the nodes (from white signifying not significantly correlated to red signifying highly significantly correlated). The topological overlap matrix strongly indicates highly interconnected subsets of genes (modules). Modules identified are colored along both column and row and are boxed. **(a) **Matrixes of the combined network for six male tissues. **(b) **Matrixes of the combined network for five female tissues. **(c) **Matrixes of the combined network for 11 male and female tissues.

Gene networks were investigated using a previously described bionetwork analysis method [31] that utilizes all the array data to examine coordinated gene expression and connectivity between specific genes [32,33]. Initially, cluster analysis of the differential gene expression lists was used to identify gene modules, which were then used to identify gene networks and functional categories. The connectivity index (k.in) for individual genes is shown in Additional file 5 and the number of connections for each gene with a cluster coefficient for male and female list comparisons is shown in Additional file 2. A cluster analysis was performed on the combined male tissue signature lists, the combined female tissue signature lists and a combination of all female and male signature lists (Figure 4). Gene modules were identified that involved coordinated gene expression and connectivity between the genes assessed. The modules are shown in colors on the axes, with white indicating no connectivity and red highest connectivity (Figure 4). The heat diagram identified modules as boxed gene sets and assigned them a specific color. The combined male and female cluster analysis demonstrates strong modularity (Figure 4c), but the sexually dimorphic transgenerational transcriptomes identified in Figure 2 suggest that sex-specific cluster analysis and modules will be more informative, and these were used in all subsequent analyses. A list of sex-specific modules and represented gene sets are shown in Table 2. Identification of co-expressed gene modules is actually a process to enhance the signal by filtering out noisy candidates using advanced soft thresholding and network techniques. To access the robustness of the approach with respect to different cutoffs for detecting differentially expressed genes, we also constructed additional male and female co-expression networks based on a more stringent mean difference cutoff of a 1.5-fold change in gene expression. The 1.5-fold networks have a smaller number of modules than their counterparts, but all the modules from the 1.5-fold networks all significantly overlapped (Fisher's exact test P-values < 1.6e-7) with the modules identified in the previous networks based on a mean difference cutoff of 1.2-fold change in gene expression.

**Table 2:** Overlap of male and female signature list genes with network modules

Signature list modules	Signature list	Tur	Blu	Brn	Red	Yel	Grn	Blk	Pink	Mag	Pur	Grn-yl	Tan	Sal	Cyn	M-blu	L cyn	Grey	L grn	L yl	Crl
**Female**		148	137	78	51	22	33	28	31	30	20	25	17	20	18	21	16	19	13	14	10
Heart	406	130	111	12	2	1	0	2	1	2	1	7	0	0	1	0	12	1	10	2	0
Kidney	151	1	13	0	5	4	1	2	0	22	1	10	0	1	17	2	0	0	0	0	0
Liver	99	2	6	3	0	0	0	1	0	1	0	5	0	0	0	2	2	0	1	0	0
Ovary	305	8	5	25	0	17	10	21	29	0	0	0	17	0	0	6	1	0	0	6	10
Uterus	279	7	0	38	44	0	14	2	1	0	18	2	0	19	0	8	1	18	0	3	0
All female lists, unique	1,298																				
																					
**Male**		1016	363	525	426	48	7	24	33	27											
Heart	172	5	32	35	21	3	0	0	3	1											
Kidney	725	86	199	114	84	8	3	2	21	12											
Liver	266	41	10	84	14	2	0	0	0	8											
Prostate	1,112	736	56	39	9	26	0	11	1	0											
SV	274	47	28	77	10	0	0	0	7	4											
Testis	552																				

Tur, turquoise; Blu, blue; Brn, brown; Yel, yellow; Grn, green; Blk, black; Mag, magenta; Pur, purple; Grn-yl, green-yellow; Sal, salmon; Cyn, cyan; M-blu, midnight blue; L cyn, light cyan; L grn, light green; L yl, light yellow; Crl, coral. SV, seminal vesicle.

The correlation of the gene modules with cellular pathways and processes is shown in Additional file 7. A relatively even distribution is observed for the various pathways with no significant over-representation. As observed with the tissue signature lists, similar pathways with the largest numbers of genes affected are represented (Additional file 7). Therefore, no predominant cellular pathway or process was observed within the gene modules identified.

Gene network analysis was performed to potentially identify the distinct or common connections between the various tissue signature lists and gene modules identified. A direct connection indicates a functional and/or binding interaction between genes while indirect connections indicate the association of a gene with a cellular process or function. This analysis used the literature-based Pathway Studio software described in the Materials and methods. Analysis of the female gene modules identified only one module (turquoise) that had a direct connection network (Additional file 3A). The gene network analysis of the male modules found that the yellow, brown and turquoise modules have direct connections (Additional file 3). None of the other female or male modules had direct connection gene networks. Therefore, no specific gene networks were common between the gene modules. The possibility that the tissue signature lists of differentially expressed genes may contain gene networks was also investigated. The majority of tissue signature lists confirmed the direct connection gene networks (Additional file 4). Analysis of the individual tissue gene networks did not show any major overlap or common regulatory gene sets within the different gene networks. Therefore, each tissue acquires a different and unique gene network that is also distinct between the sexes (sexually dimorphic; Additional file 4).

**Figure 5:**
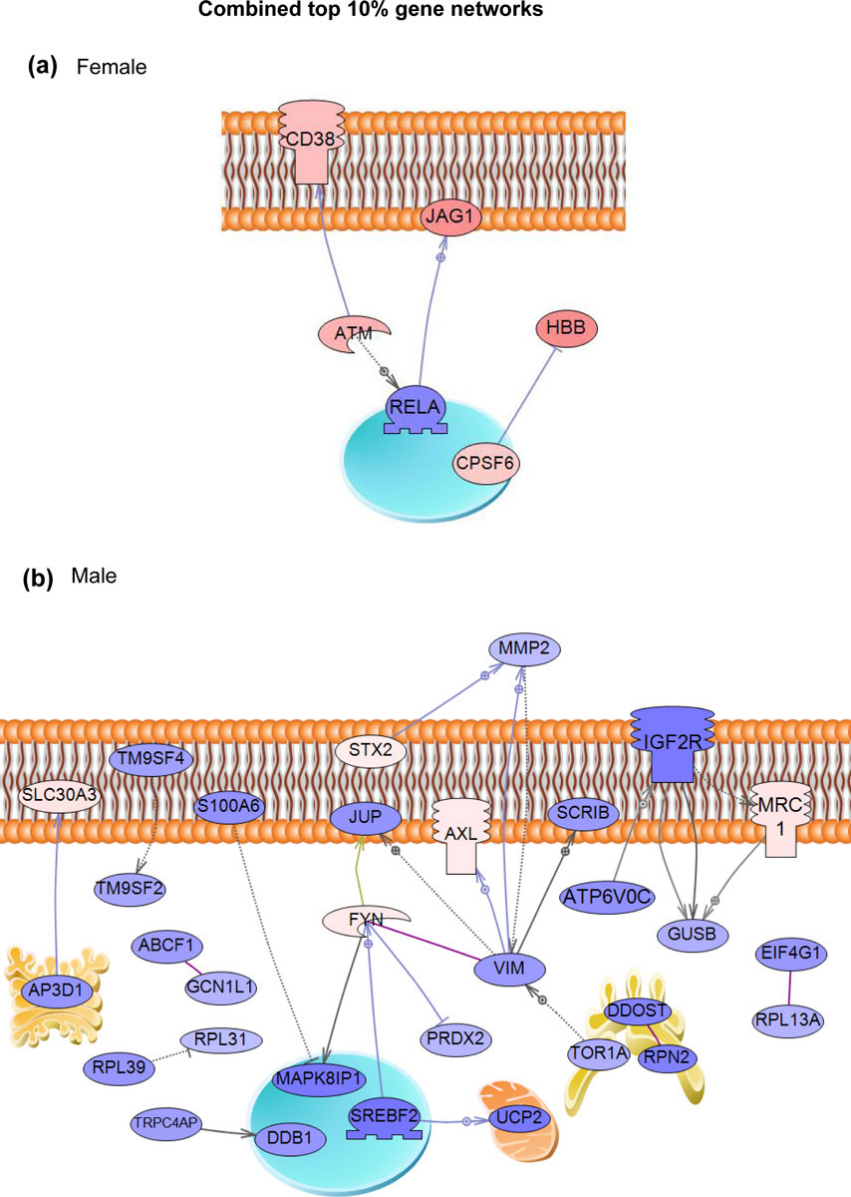
**Direct connection gene sub-networks for the top 10% interconnected genes from each module of the separate networks for female and male obtained by global literature analysis**. **(a) **Female; **(b) **male. Directly connected genes only are shown according to their location in the cell (on the membrane, in the Golgi apparatus, nucleus, or cytoplasm or outside the cell). Node shapes: oval and circle, protein; diamond, ligand; circle/oval on tripod platform, transcription factor; ice cream cone, receptor; crescent, kinase or protein kinase; irregular polygon, phosphatase. Color code: red, up-regulated genes; blue, down-regulated genes. Arrows with a plus sign indicate positive regulation/activation; arrows with a minus sign indicate negative regulation/inhibition; grey arrows represent regulation; lilac arrows represent expression; purple arrows represent binding; green arrows represent promoter binding; yellow arrows represent protein modification.

The cluster analysis (Figure 4) identified gene modules with genes with coordinated gene regulation and a connectivity index (k.in) was identified (Additional files 2 and 5). The top 10% of genes from each module with the highest connectivity index were combined for male (258 total genes) and female (75 total genes) gene modules, and gene networks identified for the male and female gene sets (Figure 5). The combined female gene module top 10% connectivity gene network identified only five directly connected genes as critical components of the network. This indicates the general lack of an underlying gene network in the female tissue modules. The combined male gene module network identified over 30 directly connected genes as critical components (Figure 5b). Although the tissue-specific gene networks are different and unique (Additional file 4), a combined gene network of the most highly connected and critical genes in the gene modules was identified for the male. Although a common gene network among the various tissues does not appear to be involved in the epigenetic transgenerational inheritance mechanism, a network involving the most connected genes between the tissues was identified for the male (Figure 5). Observations suggest additional molecular mechanisms may be involved.

### Epigenetic control regions

**Figure 6:**
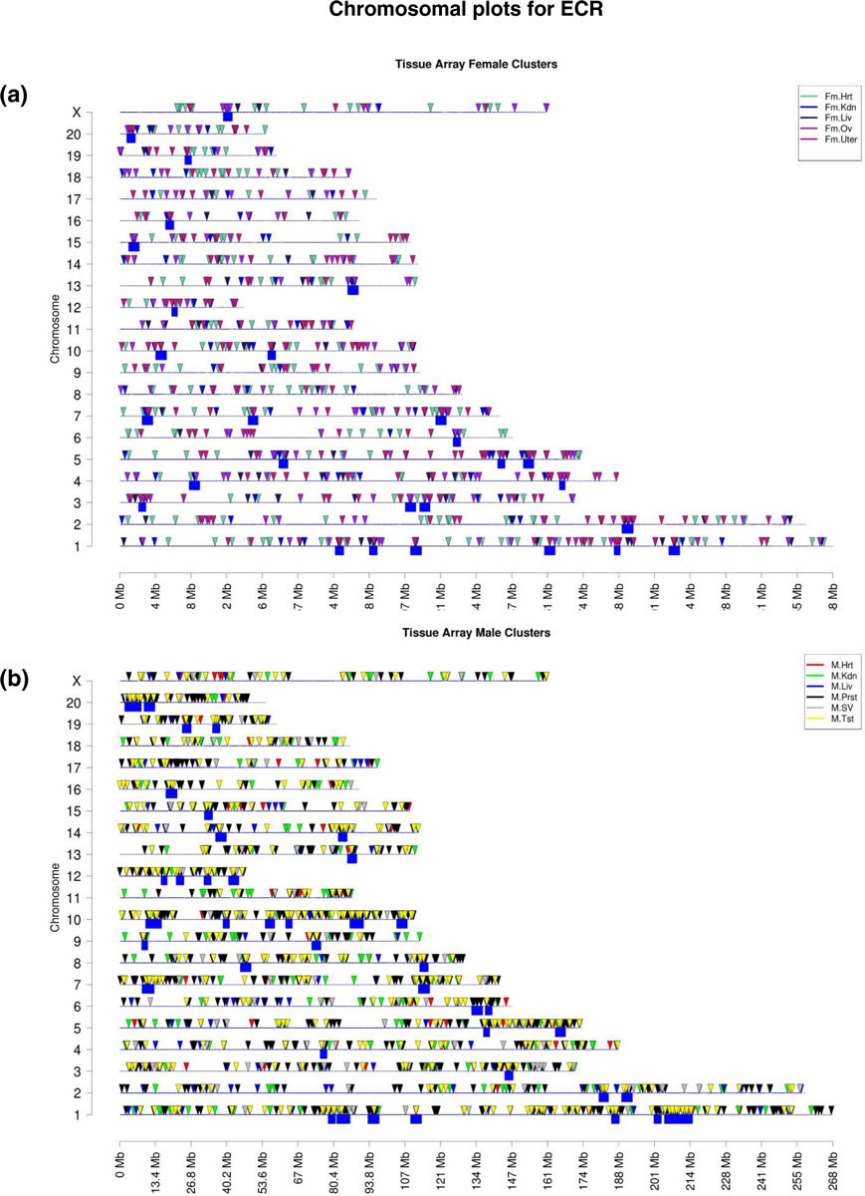
**Chromosomal locations of differentially expressed genes**. **(a) **Chromosomal plot of differential gene expression (arrow head) and ECRs (box) for five female tissue types (heart, kidney, liver, ovaries and uterus). **(b) **Chromosomal plot of ECRs for six male tissue types (heart, kidney, liver, prostate, seminal vesicle and testis). **(c) **Chromosomal plot showing clustering of male tissues and female tissues. Insets show tissue identification color code. F, female; M, male; SV, seminal vesicle.

**Figure 7:**
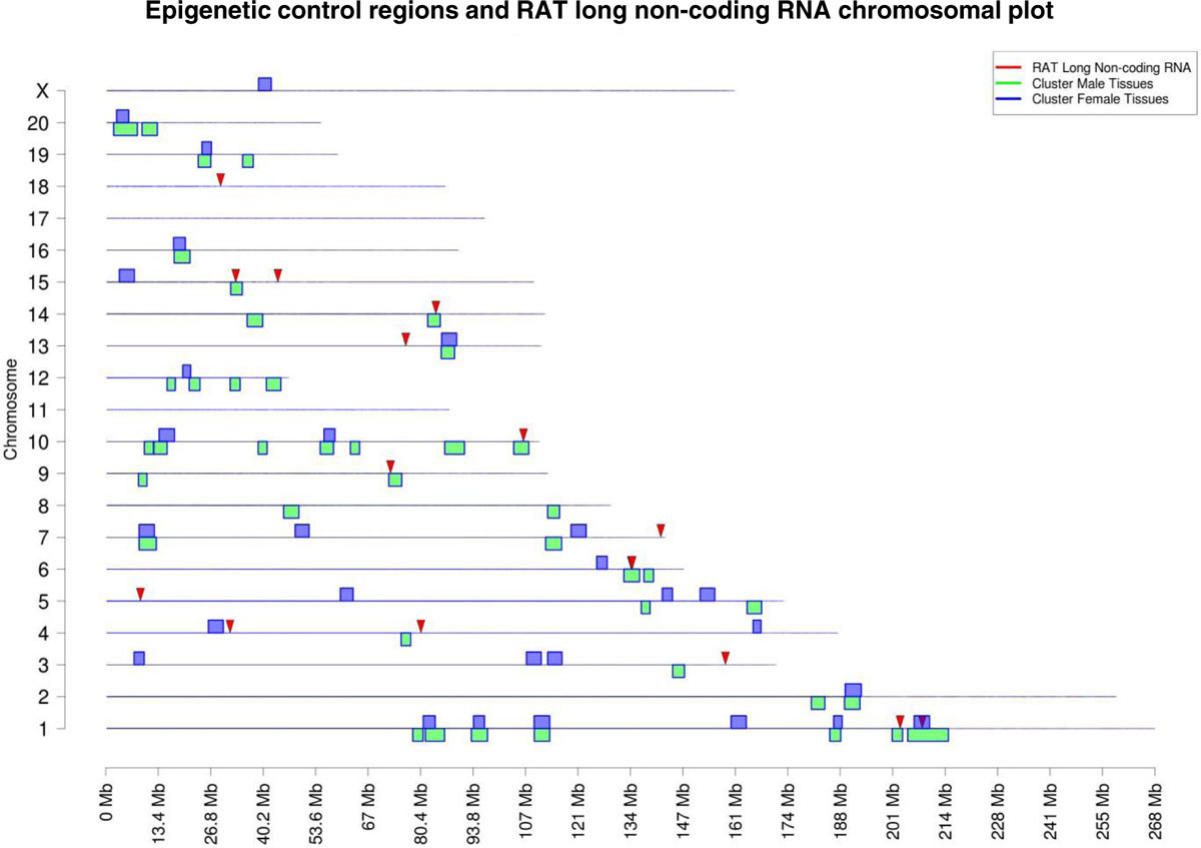
**Chromosomal plot showing gene clustering in epigenetic control regions of male tissues and female tissues overlapped with rat long non-coding RNA (arrowheads)**. Inset provides color code.

**Figure 8:**
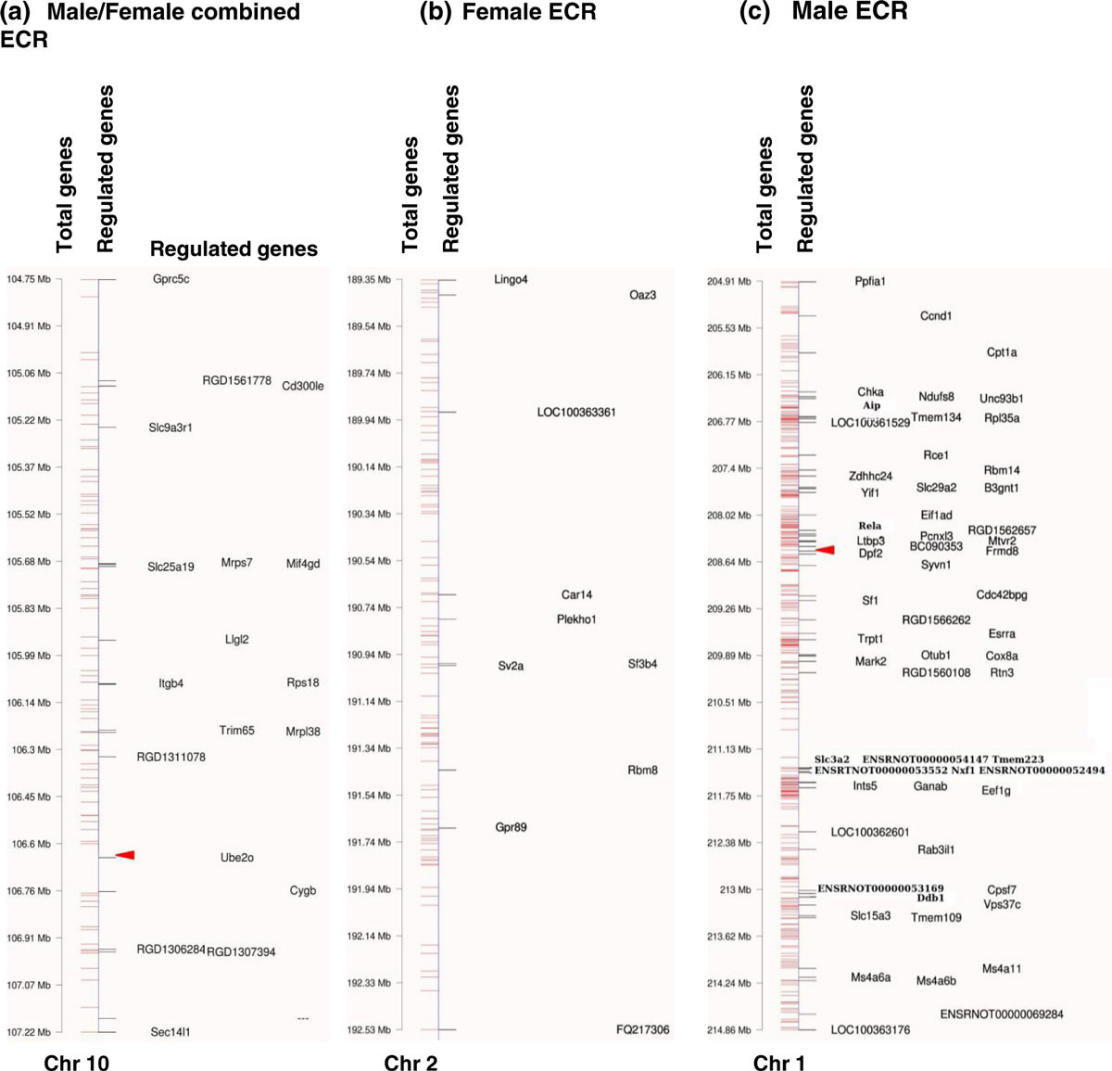
**Representative epigenetic control regions (ECRs) identifying regulated genes in single ECRs**. **(a) **ECR selected from male and female tissues combined (overlapped). **(b) **ECR selected from female only tissues. **(c) **ECR selected from male only tissues. The location of all genes (total genes) on chromosomes 1, 2 and 10 are shown in megabases and regulated genes are named. The arrowhead identifies the location of a known rat long non-coding RNA.

**Figure 9:**
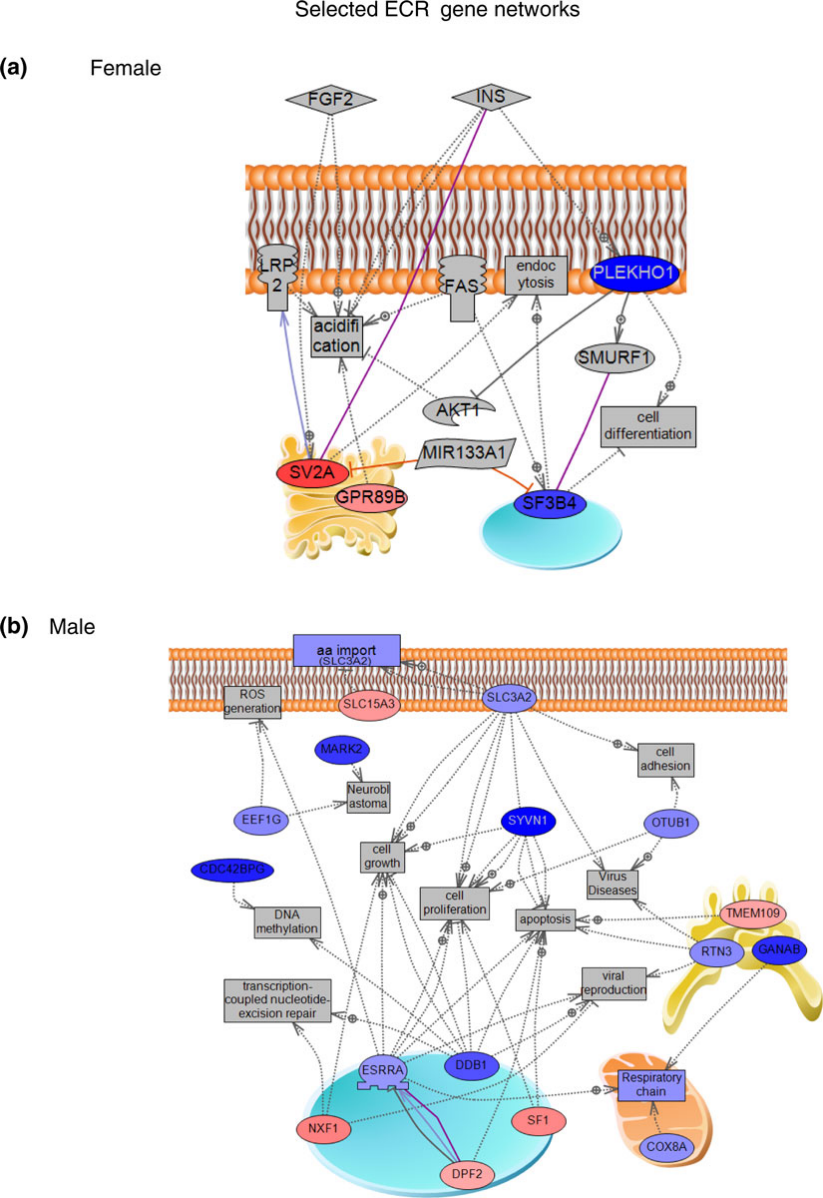
**Shortest cell processes connection gene sub-networks for genes of selected female ECR chr2-188**.8 and male ECR chr1-204.75. **(a) **ECR chr2-188.8. **(b) **Male ECR chr1-204.75. Node shapes: oval and circle, protein; diamond, ligand; circle/oval on tripod platform, transcription factor; ice cream cone, receptor; crescent, kinase or protein kinase; irregular polygon, phosphatase. Color code: red, up-regulated genes; blue, down-regulated genes. Arrows with a plus sign indicate positive regulation/activation; arrows with a minus sign indicate negative regulation/inhibition; grey arrows represent regulation; lilac arrows represent expression; purple arrows represent binding; green arrows represent promoter binding; yellow arrows represent protein modification. AA, amino acid; FAS; FGF, fibroblast growth factor; INS, insulin; LRP2, low density lipoprotein receptor-related protein 2; ROS, reactive oxygen species.

The total number of all differentially expressed genes in the tissue signature lists was 1,298 for female and 3,046 for male (Figure 1). The possibility that the chromosomal location of these genes may identify potential regulatory sites was investigated. All the genes for the female and male were mapped to their chromosomal locations and then a sliding window of 2 Mb was used to determine the regions with a statistically significant (Z test, *P *< 0.05) over-representation of regulated genes (Figure 6a,b). The analysis identified gene clusters in regions 2 to 5 Mb in size on nearly all chromosomes that have a statistically significant over-representation of regulated genes (Table 3). Several ECRs are up to 10 Mb, which we suspect involves adjacent ECRs. As these regions were associated with the epigenetic transgenerational inheritance of these tissue-specific transcriptomes, we termed them 'epigenetic control regions'. The specific ECRs are presented in Figure 7 for the female and male combined signature lists. A comparison of the female and male tissue ECRs demonstrated many were in common. The common and sex-specific ECRs are shown in Figure 7. The number of differentially regulated genes associated with these ECRs ranged from 5 to 70 (Table 3). Selected ECRs from the male and female were mapped to demonstrate the differentially expressed genes in the ECRs (Figure 8). An ECR common between male and female in chromosome 10 is shown in Figure 8a. The ECRs may provide a coordinated mechanism to regulate a set of functionally related genes that are expressed in different tissues (Additional file 8). Therefore, a limited number of regulatory sites such as the identified ECRs could regulate tissue-specific and sexually dimorphic gene expression from similar regions. However, the current study was designed simply to identify the ECRs, and their functional role remains to be established. The genes within the male and female ECRs were used to generate gene networks. The female ECR-associated genes generated a network with connection to cellular differentiation, cellular acidification and endocytosis (Figure 9a). The male ECR-associated genes generated a network linked with a larger number of cellular processes (Figure 9b). Therefore, no predominant gene network or cellular process was associated with the identified ECRs.

**Table 3:** Gene clusters and epigenetic control regions

					*P*-value range			
							
Cluster name	**Chr**.	Size (Mbp)	Start	Stop	Minimum	Maximum	Number of genes regulated	Overlap opposite sex cluster
**Female**								
Chr1-109.35	1	4	109350000	113350000	1.1E-30	8.2E-03	6	No
Chr1-159.65	1	3.9	159650000	163550000	2.1E-05	5.6E-04	4	No
Chr1-185.85	1	2.15	185850000	1.88E+08	8.2E-03	8.2E-03	5	Yes
Chr1-206.4	1	3.95	206400000	210350000	4.4E-09	8.2E-03	10	Yes
Chr1-81.05	1	3.05	81050000	84100000	8.2E-03	8.2E-03	5	Yes
Chr1-93.9	1	2.8	93900000	96700000	5.6E-04	8.2E-03	6	Yes
Chr2-188.8	2	4.1	188800000	192900000	2.1E-05	8.2E-03	10	Yes
Chr3-107.4	3	3.8	107400000	111200000	2.1E-05	8.2E-03	5	No
Chr3-112.8	3	3.7	112800000	116500000	5.6E-04	8.2E-03	6	No
Chr3-7.2	3	2.6	7200000	9800000	8.2E-03	8.2E-03	5	No
Chr4-165.3	4	2	165300000	167300000	8.2E-03	8.2E-03	5	No
Chr4-26.2	4	3.8	26200000	3.00E+07	8.2E-03	8.2E-03	4	No
Chr5-142.1	5	2.6	142100000	144700000	5.6E-04	8.2E-03	6	No
Chr5-151.75	5	3.75	151750000	155500000	2.1E-05	8.2E-03	8	No
Chr5-59.9	5	3.25	59900000	63150000	8.2E-03	8.2E-03	6	No
Chr6-125.35	6	2.7	125350000	128050000	5.6E-04	8.2E-03	6	No
Chr7-118.8	7	3.85	118800000	122650000	8.2E-03	8.2E-03	7	No
Chr7-48.35	7	3.55	48350000	51900000	2.1E-05	8.2E-03	6	Yes
Chr7-8.5	7	3.9	8500000	12400000	2.1E-05	8.2E-03	8	No
Chr10-55.7	10	2.8	55700000	58500000	8.2E-03	8.2E-03	5	Yes
Chr12-19.65	12	2	19650000	21650000	8.2E-03	8.2E-03	5	No
Chr13-85.75	13	3.85	85750000	89600000	2.1E-05	8.2E-03	9	Yes
Chr15-3.45	15	3.85	3450000	7300000	4.2E-07	8.2E-03	6	Yes
Chr16-17.3	16	3	17300000	20300000	5.6E-04	8.2E-03	6	No
Chr19-24.55	19	2.4	24550000	26950000	8.2E-03	8.2E-03	5	Yes
Chr20-2.75	20	3.1	2750000	5850000	5.6E-04	8.2E-03	7	Yes
Chrx-39	X	3.25	3.90E+07	42250000	2.1E-05	8.2E-03	7	No
								
**Male**								
Chr1-78.4	1	2.6	78400000	8.10E+07	4.9E-02	1.1E-02	17	Yes
Chr1-81.6	1	4.9	81600000	86500000	4.9E-02	7.1E-04	21	Yes
Chr1-93.35	1	4.1	93350000	97450000	4.9E-02	2.2E-05	22	Yes
Chr1-109.4	1	3.95	109400000	113350000	7.4E-11	1.2E-19	16	No
Chr1-184.85	1	2.8	184850000	187650000	4.9E-02	1.1E-02	12	Yes
Chr1-200.8	1	2.65	200800000	203450000	4.9E-02	2.4E-02	11	No
Chr1-204.75	1	10.4	204750000	215150000	4.9E-02	7.6E-08	70	Yes
Chr2-180.15	2	3.45	180150000	183600000	4.9E-02	1.9E-03	18	No
Chr2-188.65	2	3.9	188650000	192550000	4.9E-02	2.2E-05	18	Yes
Chr3-144.75	3	3	144750000	147750000	4.9E-02	4.9E-02	11	No
Chr4-75.4	4	2.45	75400000	77850000	2.4E-02	1.1E-02	12	No
Chr5-136.75	5	2.2	136750000	138950000	4.9E-02	4.9E-02	11	No
Chr5-163.75	5	3.7	163750000	167450000	4.9E-02	4.8E-03	18	No
Chr6-132.3	6	4	132300000	136300000	7.4E-11	5.4E-65	19	No
Chr6-137.4	6	2.45	137400000	139850000	4.9E-02	1.1E-02	13	No
Chr7-8.5	7	4.4	8500000	12900000	2.1E-05	8.2E-03	25	Yes
Chr7-112.3	7	4.1	112300000	116400000	4.9E-02	7.7E-05	22	No
Chr8-45.4	8	3.9	45400000	49300000	4.9E-02	7.1E-04	15	No
Chr8-112.8	8	3.05	112800000	115850000	4.9E-02	4.8E-03	14	No
Chr9-72.25	9	3.3	72250000	75550000	4.9E-02	1.1E-02	12	No
Chr9-8.35	9	2.15	8350000	10500000	4.9E-02	4.9E-02	10	No
Chr10-9.85	10	2.5	9850000	12350000	4.9E-02	4.9E-02	10	Yes
Chr10-12.15	10	3.5	12150000	15650000	2.4E-02	1.1E-02	16	Yes
Chr10-38.9	10	2.3	38900000	41200000	4.9E-02	4.9E-02	10	No
Chr10-54.75	10	3.4	54750000	58150000	4.9E-02	4.8E-03	14	Yes
Chr10-62.45	10	2.35	62450000	64800000	4.9E-02	4.9E-02	12	No
Chr10-86.55	10	5.05	86550000	91600000	4.9E-02	7.7E-05	28	No
Chr10-104.15	10	3.85	104150000	1.08E+08	4.9E-02	7.1E-04	18	No
Chr12-15.65	12	2.1	15650000	17750000	4.9E-02	4.9E-02	10	No
Chr12-21.3	12	2.75	21300000	24050000	4.9E-02	4.9E-02	11	Yes
Chr12-31.75	12	2.55	31750000	34300000	4.9E-02	2.4E-02	11	No
Chr12-41	12	3.7	4.10E+07	44700000	4.9E-02	7.7E-05	10	No
Chr13-85.65	13	3.4	85650000	89050000	4.9E-02	4.8E-03	14	Yes
Chr14-36.1	14	3.95	36100000	40050000	1.9E-03	6.1E-06	15	No
Chr14-82.2	14	3.2	82200000	85400000	4.9E-02	2.4E-02	13	No
Chr15-31.9	15	2.95	31900000	34850000	2.4E-02	4.8E-03	15	Yes
Chr16-17.45	16	4.05	17450000	21500000	4.9E-02	6.1E-06	20	Yes
Chr19-23.6	19	3.2	23600000	26800000	4.9E-02	4.8E-03	14	Yes
Chr19-34.95	19	2.7	34950000	37650000	4.9E-02	4.9E-02	11	No
Chr20-2	20	6.05	2.00E+06	8050000	4.9E-02	2.4E-04	34	Yes
Chr20-9.25	20	3.9	9250000	13150000	2.4E-02	4.8E-03	16	Yes

Previously, the ICRs identified have been shown to be associated with lncRNAs. Similar distal regulation involving lncRNAs has also been shown in plants [29,30]. The rat genome lncRNAs have not been fully characterized [34], but 20 rat lncRNAs have been reported. The possibility that these known rat lncRNAs may correlate with the identified ECRs was investigated (Figures 7 and 8). Interestingly, over half the known rat lncRNAs did correlate with the male and female ECRs. A full list of all these lncRNAs is provided in Additional file 9. Although more extensive characterization of the rat lncRNAs is required, those few known rat lncRNAs did correlate strongly with the identified ECRs. The functional role of these lncRNAs within the ECRs remains to be elucidated.

Vinclozolin-induced sperm epimutations associated with epigenetic transgenerational inheritance of adult-onset disease phenotypes have been reported [4]. Comparison of the chromosomal locations of 21 F3 generation sperm epimutations with the identified ECRs showed that they are correlated. Although specific sperm epigenetic alterations and clustered gene expression may be functionally related, further research regarding the specific epigenetic modifications within the ECRs remains to be investigated.

## Discussion

Environmentally induced epigenetic transgenerational inheritance of adult-onset disease requires an epigenetically modified germline to transmit an altered baseline epigenome between generations [1,2]. The current study utilized the commonly used agricultural fungicide vinclozolin [35], which has been shown to induce epigenetic transgenerational inheritance of disease [1,5] and permanently alter the sperm epigenome (DNA methylation) [4]. Vinclozolin has been shown to promote in F3 generation lineage animals a number of adult-onset diseases, including of testis, prostate, kidneys, the immune system, and behavior and cancer [5,36]. This high degree of a variety of adult-onset disease states suggests that baseline alteration of the sperm epigenome influences the subsequent development and function of most tissues and cell types [16]. Other factors shown to promote epigenetic transgenerational inheritance of disease include bisphenol A [8,37], dioxin [8,38], pesticides [1,8], hydrocarbons (jet fuel) [8] and nutrition [39,40]. Therefore, a number of environmental factors have been shown to promote epigenetic transgenerational inheritance of phenotypic variation and this occurs in most species [2]. The current study was designed to investigate how an altered germline epigenome promotes transgenerational adult-onset disease in a variety of different tissues.

Upon fertilization, the germline (egg or sperm) forms the zygote and the developing embryo undergoes a de-methylation of DNA to create the totipotent embryonic stem cell. As the early blastula embryo develops, DNA re-methylation is initiated, promoting tissue- and cell-specific differentiation [14,15]. A set of imprinted gene DNA methylation regions are protected from this de-methylation event to allow the specific DNA methylation pattern/programming to be transmitted between generations [17,41]. The identified vinclozolin-induced transgenerational alterations in the sperm epigenome (epimutations) [4] appear to be imprinted and transmit the altered DNA methylation regions between generations [2]. The mechanisms that allow a differential DNA methylation region to be protected from DNA de-methylation in the early embryo are not known, but are speculated to involve specific protein associations and/or other epigenetic factors. In addition, during early fetal gonadal development, the primordial germ cell DNA is de-methylated, which also involves imprinted genes. The imprinted sites are then re-methylated to maintain their original DNA methylation pattern/programming through unknown mechanisms. Therefore, how both imprinted sites and the transgenerational epimutations escape and/or reprogram to their original state remains to be elucidated and is a critical mechanism to be investigated in future studies. The epigenetic transgenerational inheritance of the altered sperm epigenome results in a modified baseline epigenome in the early embryo that will subsequently affect the epigenetic programming of all somatic cells and tissues [16,19]. The epigenome directly influences genome activity such that an altered baseline epigenome will promote altered transcriptomes in all somatic cells and tissues [16]. The current study was designed to test this hypothesis and examine the transcriptomes of a variety of tissues.

The previously observed epigenetic transgenerational inheritance of adult-onset disease involved disease in a variety of different tissues (prostate, kidney, testis, ovary), but no apparent disease in other tissues (liver, heart) [5]. Previous clinical observations have demonstrated that some tissues are more highly susceptible to develop disease than others. An alteration in the baseline epigenome and transcriptome of a tissue in certain tissues may increase susceptibility or promote disease, while others can tolerate the alterations and maintain normal function. The environmentally induced epigenetic transgenerational inheritance of adult-onset disease may be due to a baseline alteration in epigenomes and transcriptomes in somatic cells of tissues susceptible to these changes and disease.

The experimental design involved the isolation of six different tissues from males and five tissues from females. These tissues were obtained from young adult rats prior to any disease onset. The F3 generation control and vinclozolin lineage animals from different litters were used and tissues obtained from six different animals for each sex, tissue and lineage. A microarray analysis was used to assess transgenerational alterations in the tissue-specific transcriptomes between control versus vinclozolin lineage animals. The differentially expressed genes for a specific tissue are referred to as a signature list. Analysis of the various tissue signature lists demonstrated negligible overlap among tissues or between sexes. Therefore, the transgenerational transcriptomes were observed in all tissues, but each tissue had a sexually dimorphic tissue-specific transgenerational transcriptome. The hypothesis that an altered transgenerational germline epigenome would promote transgenerational alterations in all somatic transcriptomes is supported by the observations of the current study. The initial bioinformatics analysis involved examination of the various tissue signature lists to correlate the involvement of cellular signaling pathways or processes among the various signature lists. The majority of pathways included genes from each signature list, but none were predominant among the signature lists. Gene functional categories that were generally predominant in the cell, such as signaling or metabolism, were also the most predominant among the signature lists. Therefore, a common pathway or process was not present among the observed transgenerational transcriptomes.

A more extensive analysis of the differentially expressed genes in all the tissues involved a previously described gene bionetwork analysis [31,42]. The coordinated gene expression and connectivity between the regulated genes was considered in a cluster analysis (Figure 4). Gene modules of interconnected genes with coordinated gene expression were identified in both a combined male and female signature list analysis, and separate male and female analyses. Although defined modularity was identified in the combined analysis, the sexually dimorphic transgenerational transcriptomes and distinct tissue physiology suggested the separate male and female analyses would be more informative. The sex-specific modules were used to determine if any over-represented gene sets were present in specific tissues. Generally, each tissue had a specific module of differentially regulated genes (Table 2). For example, prostate was predominant in the male turquoise module and female heart in the female turquoise module. In contrast, in the analysis of cellular signaling pathways or processes, the gene modules did not have over-represented pathways (Additional file 7). The tissue-specific modules did not generally reflect a specific pathway or process. Therefore, the gene bionetwork analysis identified gene modules associated with specific tissues, but the modules did not generally contain predominant cellular pathways or processes.

The transgenerational transcriptome data analysis was extended with a literature-based gene network analysis. Direct connection networks (DCNs), involving genes with direct functional and/or binding links, were identified for a number of the male and female gene modules, but the majority did not have specific gene networks. Each DCN corresponds to a previously identified co-expressed gene module. Specifically, the nodes of a DCN were the members of the corresponding co-expressed gene module but the links in the DCN were based on the literature and known databases. The modules with an identified gene network suggest that those specific tissues and abnormal physiology are potentially regulated by the network (Table 2; Additional file 3). The female turquoise module associated with the heart, male yellow module associated with testis, male brown module associated with kidney, liver and seminal vesicle, and male turquoise module associated with prostate. Each of these gene networks is unique and provides a potential regulated gene set associated with abnormal tissue pathology. Future studies will need to consider these gene networks with regard to the pathophysiology of the specific tissues. An alternative gene network analysis involved the different tissue signature lists and tissue-specific direct connection gene network analysis (Additional file 4). Tissue-specific gene networks were identified for female heart, kidney, ovary and uterus, and for male heart, kidney and liver. Similar to the observed lack of overlap between the tissue-specific signature lists (Figure 2), negligible overlap was found between the tissue-specific gene networks (Additional file 4). These tissue-specific direct connection gene networks also provide regulated sub-networks of genes associated with the previously identified abnormal transgenerational tissue pathologies [5]. Interestingly, the gene network associated with the female turquoise module was similar to the female heart tissue-specific gene network. This regulated female heart network provides an interconnected gene set that could be investigated in future studies on heart pathophysiology. The final direct connection gene network analysis involved the combined male tissue and combined female tissue regulated gene sets. The combined female tissue network involved a small network of six genes, suggesting a gene network was not common among the different female tissues. The combined male tissue network involved a larger gene set of over 30 genes (Figure 5), which had elements similar to the male kidney network (Additional file 4). The similarities suggest this gene network may be associated with the observed kidney pathophysiology and needs to be investigated in future studies [5]. Although this combined male tissue direct connection gene network suggests a potential common regulatory gene set among the tissues, the tissue-specific transgenerational transcriptomes have negligible overlap (Figure 2) and distinct tissue-specific gene networks (Additional file 4). Observations suggest the transgenerational somatic transcriptomes are primarily tissue-specific without common gene networks or specific pathways associated with the adult-onset disease that developed in the specific tissues.

To understand how a limited number of sperm epimutations can lead to such a diverse gene expression profile between tissues, an epigenetic mechanism needs to be considered. As discussed, somatic cells and tissues will have a shift in the baseline epigenome derived from sperm that promotes distinct cellular and tissue differentiation [16,19]. Therefore, it is not surprising each cell type has a distinct epigenome and transcriptome to promote cell-specific differentiated functions. The classic dogma that a gene's promoter is the central regulatory site involved in regulating its expression is not sufficient to explain the over 4,000 genes differentially regulated between the different tissues examined (Figure 1). A potential alternative epigenetic mechanism involves an ECR that can regulate gene expression within a greater than 2 Mb region together with, for example, lncRNAs and chromatin structure. An example of such a mechanism has been previously described as an ICR, where an imprinted DNA methylation site (for example, *H19 *and *IGF2*) influences a lncRNA to regulate gene expression for over a megabase in either direction [17,22,23,27]. The imprinted *H19 *and *IGF2 *loci together with a lncRNA have been shown to distally regulate the expression of multiple different genes [17,25,26,28]. These ICRs are likely a small subset of a larger set of ECRs, most not involving imprinted gene sites. Another example has been shown in plants where lncRNAs regulate distal gene expression associated with specific plant physiological phenotypes [29,30]. The current study used the various tissue transgenerational transcriptomes to identify the potential presence of ECRs.

The ECRs were defined as having a statistically significant (Z test) over-representation of gene expression within an approximately 2 Mb region. The male and female sets of differentially expressed genes were used separately to identify regions with statistically significant (Z test) over-representation (*P *< 0.05). The differentially expressed genes were mapped to the chromosomes and then a 2 Mb sliding window was used to identify potential ECRs (Figures 6 and 7). For the male, over 40 ECRs were identified, and for the female, approximately 30 ECRs were identified. Approximately half the ECRs were found to be in common between male and female (Figure 7). The ECRs identified ranged from 2 to 5 Mb in size and the numbers of genes regulated ranged from 5 to 50 (Table 3). Interestingly, different genes in different tissues were found to be expressed within these ECRs (Additional file 8). The majority of the expression sites of currently known rat lncRNAs correlated with the identified ECRs (Figure 7; Additional file 9). Therefore, it is proposed that a single ECR could regulate tissue-specific gene expression that has been programmed during differentiation to express a specific set of genes within the ECR. This could explain how a limited number of epimutations could have a much broader effect on genome activity and clarify how tissue-specific transgenerational transcriptomes develop. The current study outlines the association of gene expression with the potential ECRs, but does not provide a functional link between epigenetic differential DNA methylation regions or lncRNAs and gene expression regulation within them. Therefore, future studies are now critical to assess the functional role of these ECRs and underlying epigenetic mechanisms.

## Conclusions

A systems biology approach was taken to elucidate the molecular mechanism(s) involved in environmentally induced epigenetic transgenerational inheritance of adult-onset disease. The current study identifies tissue-specific transgenerational transcriptomes with tissue-specific gene networks. A combination of epigenetic and genetic mechanisms is required to reach these differentiated tissue states that can not be explained through genetic or epigenetic mechanisms alone. The identification of potential epigenetic control regions that regulate regions of the genome in a coordinated manner may help explain in part the mechanism behind the process of emergence [43]. In a revolutionary systems biology consideration the emergence of a phenotype or process involves the coordinated and tissue-specific development of unique networks (modules) of gene expression [44]. Since the initial identification of epigenetics [45], its role in system development at the molecular level has been appreciated. The current study suggests a more genome-wide consideration involving ECRs and tissue-specific transcriptomes may contribute, in part, to our understanding of how environmental factors can influence biology and promote disease states.

Combined observations demonstrate that environmentally induced epigenetic transgenerational inheritance of adult-onset disease [2] involves germline (sperm) transmission of an altered epigenome [4] and these epimutations shift the base line epigenomes in all somatic tissues and cells derived from this germline [16]. This generates tissue-specific transgenerational transcriptomes that do not involve common gene networks or pathways, which associate with the adult-onset disease in the tissues. All tissues develop a transgenerational transcriptome, which helps explain the phenotypic variation observed. Some tissues are sensitive to shifts in their transcriptomes and develop disease, while others are resistant to disease development. The observation that all tissues develop a specific transgenerational transcriptome can help explain the mechanism behind complex disease syndromes. Those tissues sensitive to developing disease will be linked into a complex disease association due to these transgenerational transcriptome modifications. This epigenetic mechanism involves ECRs that can have dramatic effects on genome activity and promote tissue-specific phenomena. Although the functional roles of these ECRs remain to be investigated, their potential impact on expanding our concepts of gene regulation, the elucidation of emergent properties of unique gene networks, and providing links to various tissue functions and diseases are anticipated to be significant. The observations provided help elucidate the molecular mechanisms involved in environmentally induced epigenetic transgenerational inheritance of adult-onset disease and the phenotypic variation identified.

## Materials and methods

### Animal procedures

All experimental protocols involving rats were pre-approved by the Washington State University Animal Care and Use Committee. Hsd:Sprague Dawley^®^™SD^®^™ female and male rats of an outbred strain (Harlan, Indianapolis, IN, USA) were maintained in ventilated (up to 50 air exchanges per hour) isolator cages containing Aspen Sani chips (pinewood shavings from Harlan) as bedding, on a 14 h light: 10 h dark regimen, at a temperature of 70°F and humidity of 25% to 35%. Rats were fed *ad libitum *with standard rat diet (8640 Teklad 22/5 Rodent Diet; Harlan) and *ad libitum *tap water for drinking.

At proestrus as determined by daily vaginal smears, the female rats (90 days of age) were pair-mated with male rats (120 days). On the next day, the pairs were separated and vaginal smears were examined microscopically. In the event sperm were detected (day 0) the rats were tentatively considered pregnant. Vaginal smears were continued for monitoring diestrus status until day 7. Pregnant rats were then given daily intraperitoneal injections of vinclozolin (100 mg/kg/day) with an equal volume of sesame oil (Sigma, St. Louis, MO, USA) on days E8 through E14 of gestation [6]. Treatment groups were Control (DMSO vehicle) and Vinclozolin. The pregnant female rats treated with DMSO or vinclozolin were designated as the F0 generation.

The offspring of the F0 generation were the F1 generation. The F1 generation offspring were bred to other F1 animals of the same treatment group to generate an F2 generation and then F2 generation animals bred similarly to generate the F3 generation animals. No sibling or cousin breedings were performed so as to avoid inbreeding. Note that only the original F0 generation pregnant females were injected with the DMSO or vinclozolin.

Six female and six male rats of the F3 generation Control and Vinclozolin lineages at 120 days of age were euthanized by CO_2 _inhalation and cervical dislocation. Tissues, including testis, prostate, seminal vesicle, kidney, liver, heart, ovary and uterus, were dissected from rats and were processed and stored in TRIZOL (Invitrogen, Grand Island, NY, USA) at -80°C until RNA extraction. High quality RNA samples were assessed with gel electrophoresis and required a minimum OD260/280 ratio of 1.8. Three samples each of control and treated ovaries were applied to microarrays. For each of three Vinclozolin or Control microarray samples, RNA from two rats were pooled. The same pair of rats was used for each tissue type.

### Microarray analysis

The microarray hybridization and scanning was performed by the Genomics Core Laboratory, Center for Reproductive Biology, Washington State University, Pullman, WA using standard Affymetrix reagents and protocol. Briefly, mRNA was transcribed into cDNA with random primers, cRNA was transcribed, and single-stranded sense DNA was synthesized, which was fragmented and labeled with biotin. Biotin-labeled single-stranded DNA was then hybridized to the Rat Gene 1.0 ST microarrays containing more than 30,000 transcripts (Affymetrix, Santa Clara, CA, USA). Hybridized chips were scanned on an Affymetrix Scanner 3000. CEL files containing raw data were then pre-processed and analyzed with Partek Genomic Suite 6.5 software (Partek Incorporated, St Louis, MO, USA) using an RMA (Robust Multiarray Average), GC-content adjusted algorithm. Raw data pre-processing was performed in 11 groups, one for each male or female tissue. Comparison of array sample histogram graphs for each group showed that data for all chips were similar and appropriate for further analysis (Additional file 1).

The microarray quantitative data involve signals from an average 28 different oligonucleotides (probes) arrayed for each transcript and many genes are represented on the chip by several transcripts. The hybridization to each probe must be consistent to allow a statistically significant quantitative measure of the resulting gene expression signal. In contrast, a quantitative PCR procedure uses only two oligonucleotides and primer bias is a major factor in this type of analysis. Therefore, we did not attempt to use PCR-based approaches as we feel the microarray analysis is more accurate and reproducible without primer bias.

All microarray CEL files from this study have been deposited with the NCBI gene expression and hybridization array data repository Gene Expression Omnibus (GEO series accession number [GSE35839]) and can also be accessed through the Skinner Laboratory website [46]. For gene annotation, Affymetrix annotation file RaGene1_0stv1.na32.rn4.transcript.csv was used.

### Network analysis

The network analysis was restricted to genes differentially expressed between the control and the treatment groups based on previously established criteria of fold change of group means ≥1.2, a mean difference >10, and *P*-value ≤ 0.05. A change in gene expression of 20% for many genes, particularly transcriptome factors, has been shown to have important cellular and biological effects. Therefore, the 1.2-fold cutoff was selected to maintain all expression information and not a more stringent one to simply reduce the gene list size. To eliminate baseline signal gene expression changes, a mean difference >10 was used. All genes required a statistical difference *P *< 0.05 to be selected. The union of the differentially expressed genes from the tissues resulted in 5,266 genes for males and 1,909 for females being identified and used for constructing a weighted gene co-expression network [47,48]. Unlike traditional un-weighted gene co-expression networks in which two genes (nodes) are either connected or disconnected, the weighted gene co-expression network analysis assigns a connection weight to each gene pair using soft-thresholding and thus is robust to parameter selection. The weighted network analysis begins with a matrix of the Pearson correlations between all gene pairs, then converts the correlation matrix into an adjacency matrix using a power function: *f*(*x*) = *x^β^*. The parameter *β *of the power function is determined in such a way that the resulting adjacency matrix (that is, the weighted co-expression network) is approximately scale-free. To measure how well a network satisfies a scale-free topology, we use the fitting index proposed by Zhang and Horvath [47] (that is, the model fitting index *R^2 ^*of the linear model that regresses *log*(*p*(*k*)) on *log*(*k*) where k is connectivity and *p*(*k*) is the frequency distribution of connectivity). The fitting index of a perfect scale-free network is 1.

To explore the modular structures of the co-expression network, the adjacency matrix is further transformed into a topological overlap matrix [49]. As the topological overlap between two genes reflects not only their direct interaction but also their indirect interactions through all the other genes in the network. Previous studies [47,49] have shown that topological overlap leads to more cohesive and biologically meaningful modules. To identify modules of highly co-regulated genes, we used average linkage hierarchical clustering to group genes based on the topological overlap of their connectivity, followed by a dynamic cut-tree algorithm to dynamically cut clustering dendrogram branches into gene modules [50]. Such networks were generated from combined 6 male or 5 female differentially expressed gene sets (2 networks) or from combined male and female 11-tissue signature lists. From 9 to 20 modules were identified in either of 3 networks and the module size range was from 7 to 1,040 genes.

To distinguish between modules, each module was assigned a unique color identifier, with the remaining, poorly connected genes colored grey. The hierarchical clustering over the topological overlap matrix (TOM) and the identified modules is shown (Figure 4). In this type of map, the rows and the columns represent genes in a symmetric fashion, and the color intensity represents the interaction strength between genes. This connectivity map highlights that genes in the transcriptional network fall into distinct network modules, where genes within a given module are more interconnected with each other (blocks along the diagonal of the matrix) than with genes in other modules. There are a couple of network connectivity measures, but one particularly important one is the within module connectivity (k.in). The k.in of a gene was determined by taking the sum of its connection strengths (co-expression similarity) with all other genes in the module to which the gene belonged.

#### Gene co-expression cluster analysis clarification

Gene networks provide a convenient framework for exploring the context within which single genes operate. Networks are simply graphical models composed of nodes and edges. For gene co-expression clustering, an edge between two genes may indicate that the corresponding expression traits are correlated in a given population of interest. Depending on whether the interaction strength of two genes is considered, there are two different approaches for analyzing gene co-expression networks: 1) an unweighted network analysis that involves setting hard thresholds on the significance of the interactions; and 2) a weighted approach that avoids hard thresholds. Weighted gene co-expression networks preserve the continuous nature of gene-gene interactions at the transcriptional level and are robust to parameter selection. An important end product from the gene co-expression network analysis is a set of gene modules in which member genes are more highly correlated with each other than with genes outside a module. Most gene co-expression modules are enriched for GO functional annotations and are informative for identifying the functional components of the network that are associated with disease [51].

This gene co-expression clustering/network analysis (GCENA) has been increasingly used to identify gene sub-networks for prioritizing gene targets associated with a variety of common human diseases such as cancer and obesity [52-56]. One important end product of GCENA is the construction of gene modules composed of highly interconnected genes. A number of studies have demonstrated that co-expression network modules are generally enriched for known biological pathways, for genes that are linked to common genetic loci and for genes associated with disease [42,47,51-55,57,58]. In this way, one can identify key groups of genes that are perturbed by genetic loci that lead to disease, and that define at the molecular level disease states. Furthermore, these studies have also shown the importance of the hub genes in the modules associated with various phenotypes. For example, GCENA identified *ASPM*, a hub gene in the cell cycle module, as a molecular target of glioblastoma [55] and *MGC4504*, a hub gene in the unfolded protein response module, as a target potentially involved in susceptibility to atherosclerosis [53].

### Pathway and functional category analysis

Resulting lists of differentially expressed genes for each male or female tissue were analyzed for gene functional categories with GO categories from the Affymetrix annotation site. Each module generated in male or female network analysis were analyzed for KEGG (Kyoto Encyclopedia for Genes and Genome, Kyoto University, Japan) pathway enrichment using the KEGG website 'Search Pathway' tool. Global literature analysis of various gene lists was performed using Pathway Studio 8.0 software (Ariadne Genomics, Inc., Rockville, MD, USA) and used to generate the direct and indirect gene connection networks.

### Chromosomal location of ECRs

An R-code was developed to find chromosomal locations of ECRs. A 2 Mb sliding window with 50,000 base intervals was used to find the associated genes in each window. A Z-test statistical analysis with *P *< 0.05 was used on these windows to find the ones with over-representation of differentially expressed genes. The consecutive windows with over-represented genes were merged together to form clusters of genes termed ECRs. Typical ECR regions range from 2 to 5 Mb, with the largest being 10 Mb.

## Abbreviations

DCN: direct connection network; DMSO: dimethylsulfoxide; E: embryonic day; ECR: epigenetic control region; GCENA: gene co-expression clustering/networks analysis; GO: Gene Ontology; ICR: imprinting control region; k.in: connectivity index; lncRNA: long non-coding RNA.

## Competing interests

The authors declare that they have no competing interests.

## Authors' contributions

MKS designed the study; MM, MIS, MH and BZ performed the experiments and analysis; all authors reviewed the data; MKS wrote the manuscript; all authors edited the manuscript; all authors have read and approved the manuscript for publication.

## Supplementary Material

Additional file 1 **Figure S1 - microarray histogram quality control**. **(a-l) **Sample histograms and box plots for microarray raw (a) and pre-processed signal values, using a RMA (Robust Multiarray Average), GC-content-adjusted algorithm for 11 male and female tissues (b-l). Click here for file

Additional file 2 **Figure S2 - cluster coefficient and connections**. **(a,b) **Cluster coefficient versus number of connections for male (a) and female (b) network modules. Click here for file

Additional file 3 **Figure S3 - gene networks from gene modules**. **(a-d) **Direct connection sub-networks for female and male modules: (a) female turquoise; (b) male yellow; (c) male brown; (d) male turquoise. Shape and color codes are the same as for Figure 5. Click here for file

Additional file 4 **Figure S4 - gene networks from signature lists**. **(a-d) **Direct connection sub-networks for female and male tissue signature lists: (a) female heart; (b) female kidney; (c) male ovary; (d) uterus; (e) male heart; (f) male kidney; (g) male liver. Shape and color codes are the same as for Figure 5. Click here for file

Additional file 5 **Table S1 - differentially expressed genes in tissues**. Click here for file

Additional file 6 **Table S2 - pathway enrichment in signature lists**. Click here for file

Additional file 7 **Table S3 - pathway enrichment in tissue modules**. Click here for file

Additional file 8 **Table S4 - epigenetic control regions and gene expression**. Click here for file

Additional file 9 **Table S5 - lncRNA and epigenetic control regions**. Click here for file
