# Supplementary material for: Epigenetic transgenerational inheritance of somatic transcriptomes and epigenetic control regions
Source: Genome Biol. 2012 Oct 3;13(10):R91. doi: 10.1186/gb-2012-13-10-r91 (PMC3491419; doi:10.1186/gb-2012-13-10-r91)

Supplemental Figure S1 (Color)

Microarray Quality Control

A

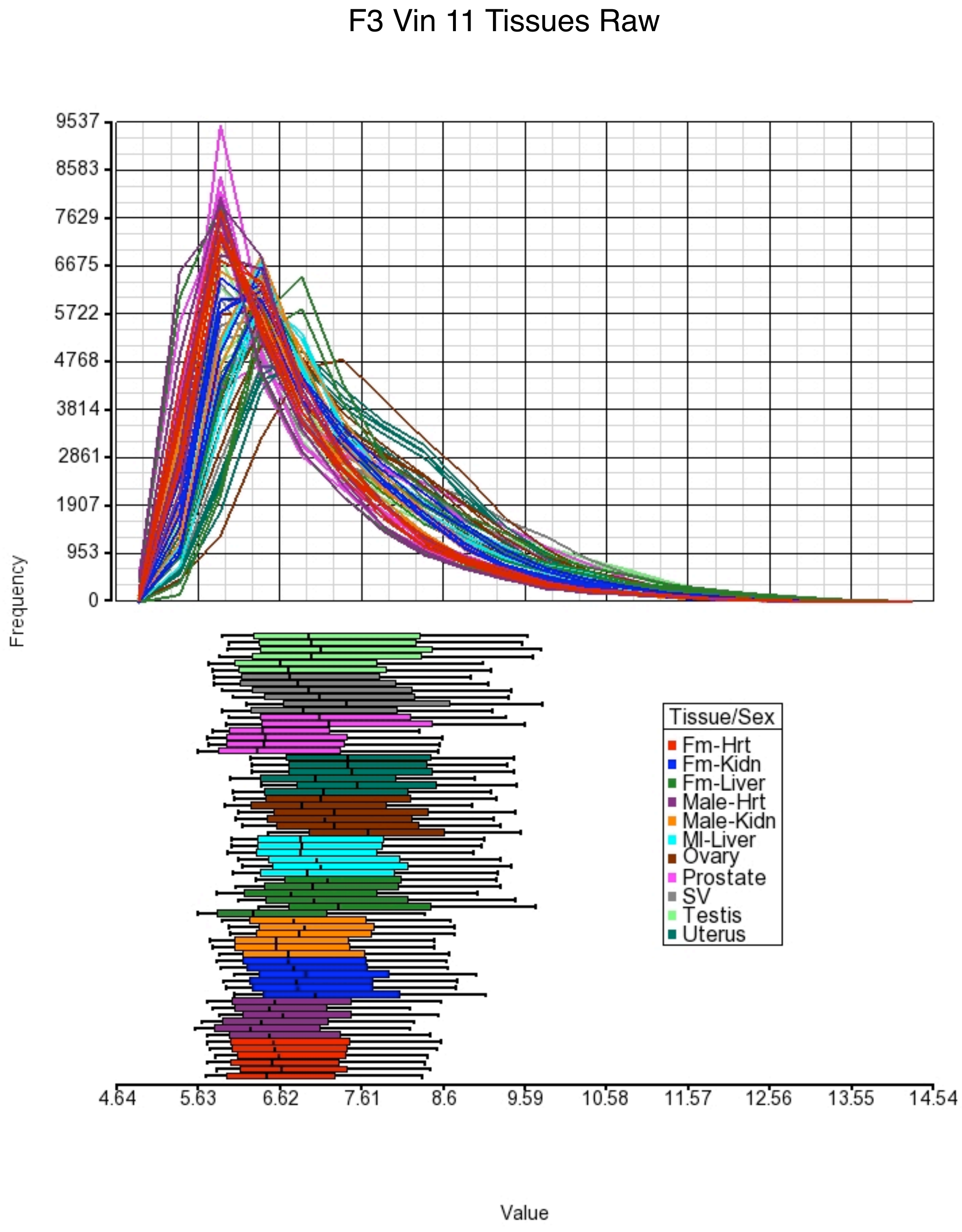

Supplemental Figure S1

Microarray Quality Control

B Female Heart

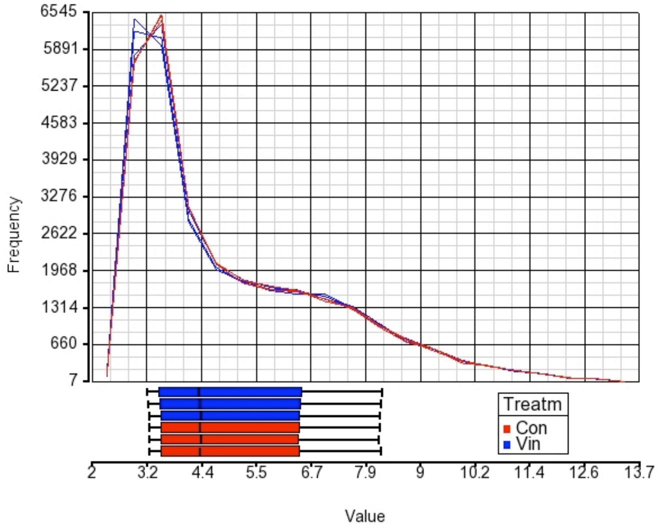

C Female Kidney

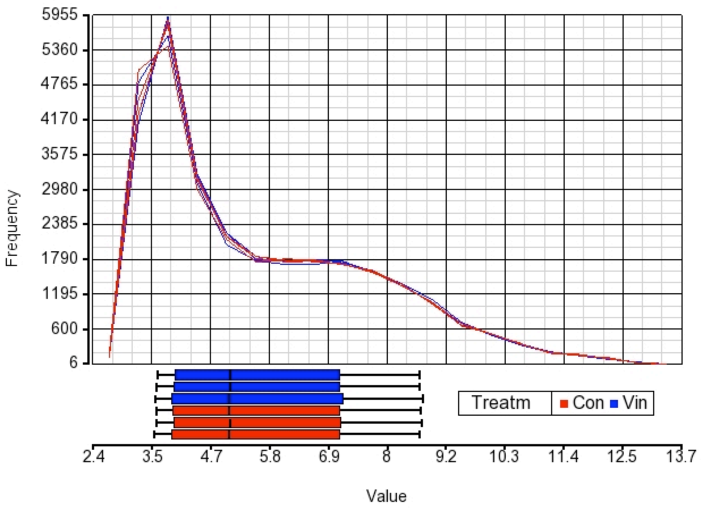

D Female Liver

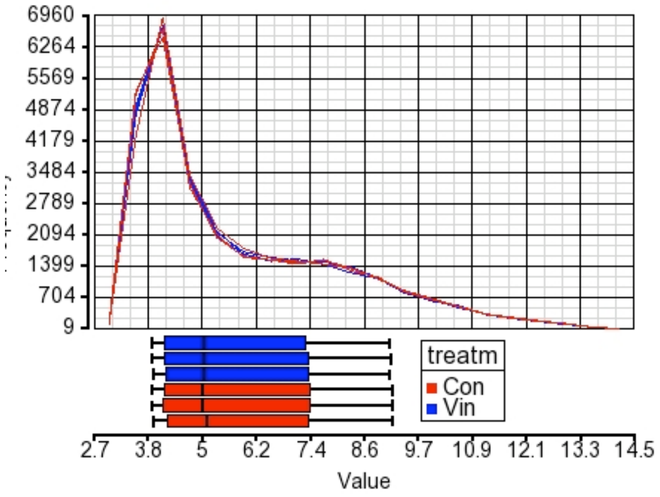

E Female Ovary

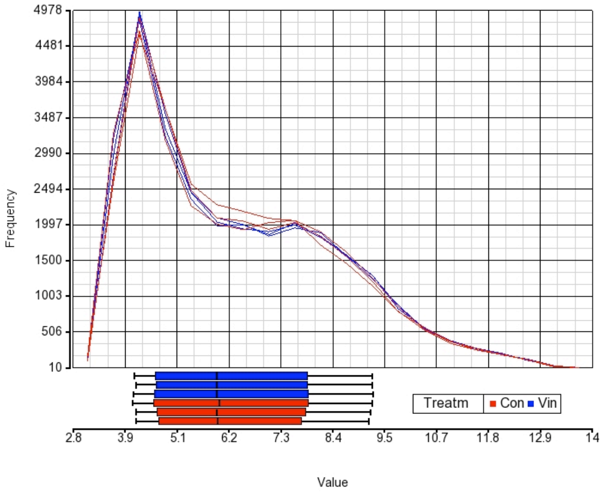

F Female Uterus

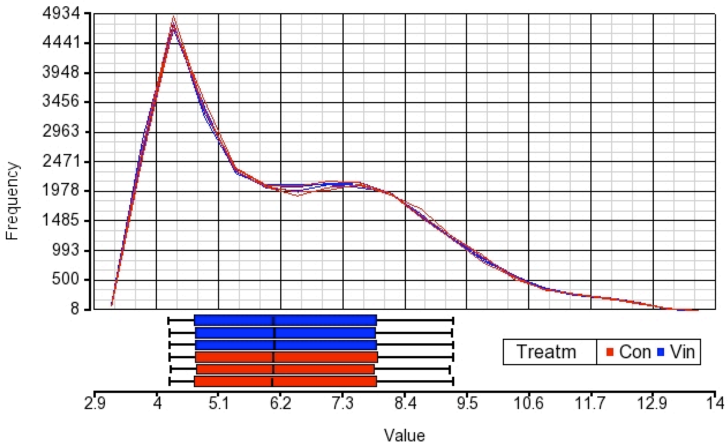

Supplemental Figure S1

G Male Heart

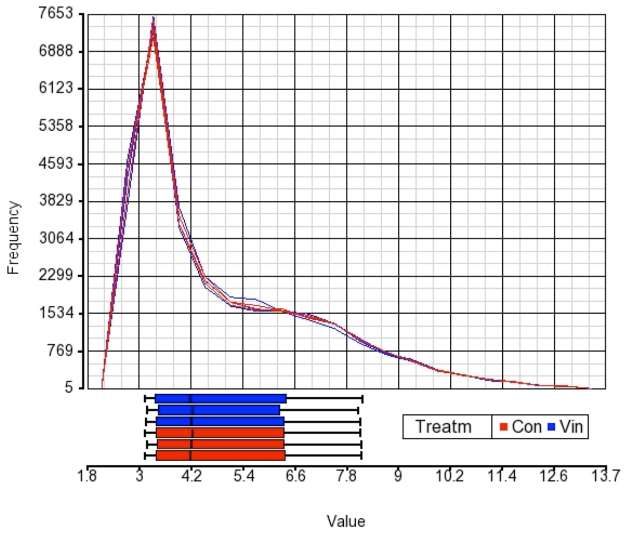

H Male Kidney

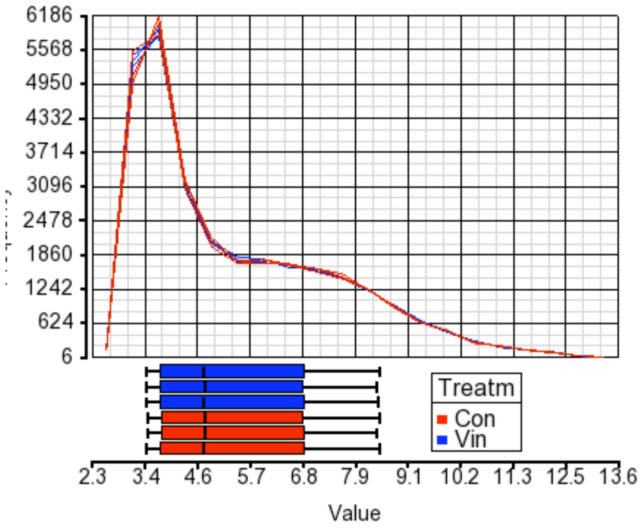

I Male Liver

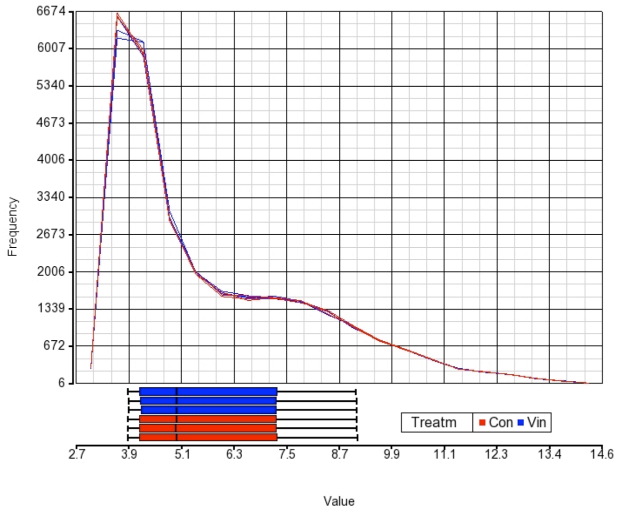

J Male Prostate

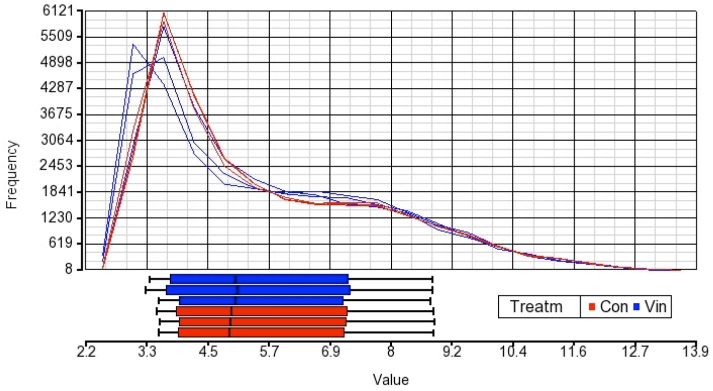

K Male SV

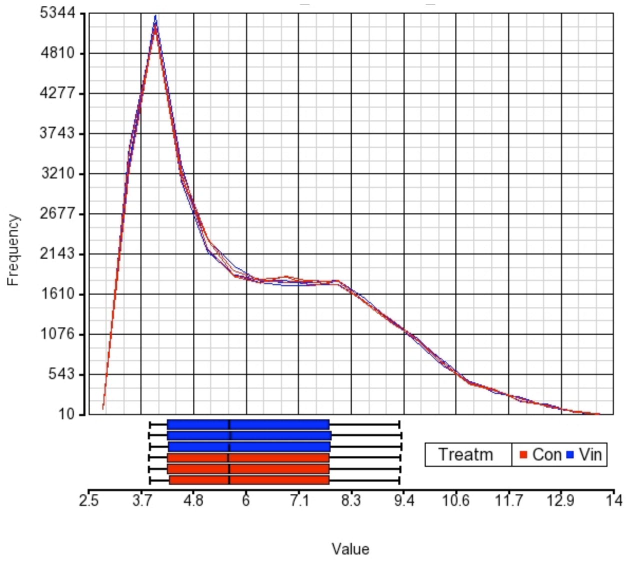

L Male Testis

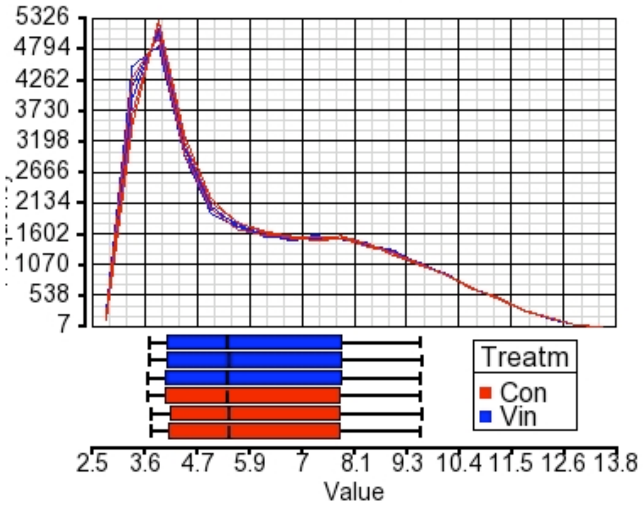

Supplement: Additional file 1 — Figure S1 - microarray histogram quality control. (a-l) Sample histograms and box plots for microarray raw (a) and pre-processed signal values, using a RMA (Robust Multiarray Average), GC-content-adjusted algorithm for 11 male and female tissues (b-l). [file gb-2012-13-10-r91-S1.pdf]
