# Supplementary figures and images for: Epigenetic transgenerational inheritance of somatic transcriptomes and epigenetic control regions
Source: Genome Biol. 2012 Oct 3;13(10):R91. doi: 10.1186/gb-2012-13-10-r91 (PMC3491419; doi:10.1186/gb-2012-13-10-r91)

**A. Male Combined Signature List Genes**

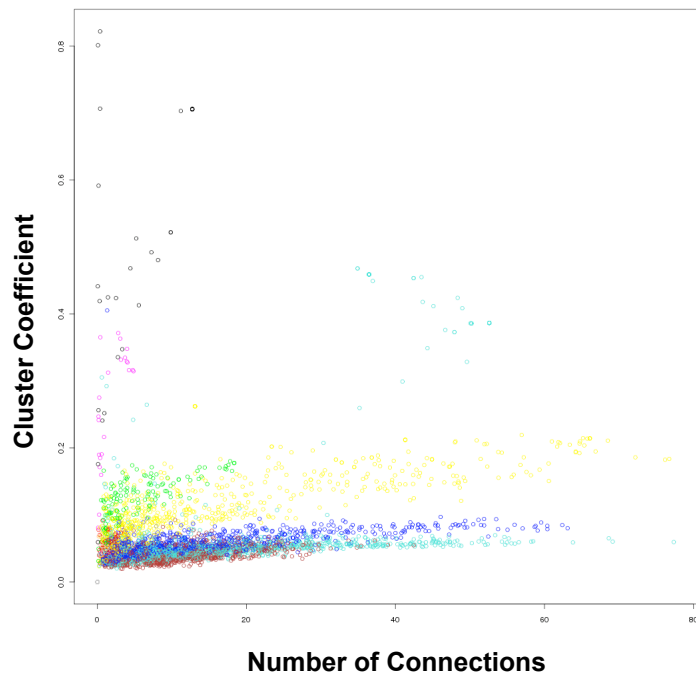

**B. Female Combined Signature List Genes**

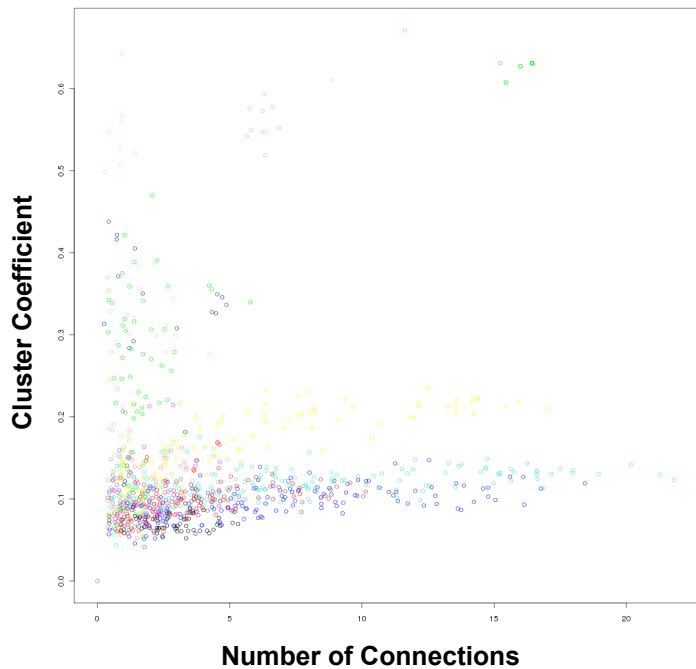

Supplement: Additional file 2 — Figure S2 - cluster coefficient and connections. (a,b) Cluster coefficient versus number of connections for male (a) and female (b) network modules. [file gb-2012-13-10-r91-S2.pdf]

Module Networks

A Female Turquoise

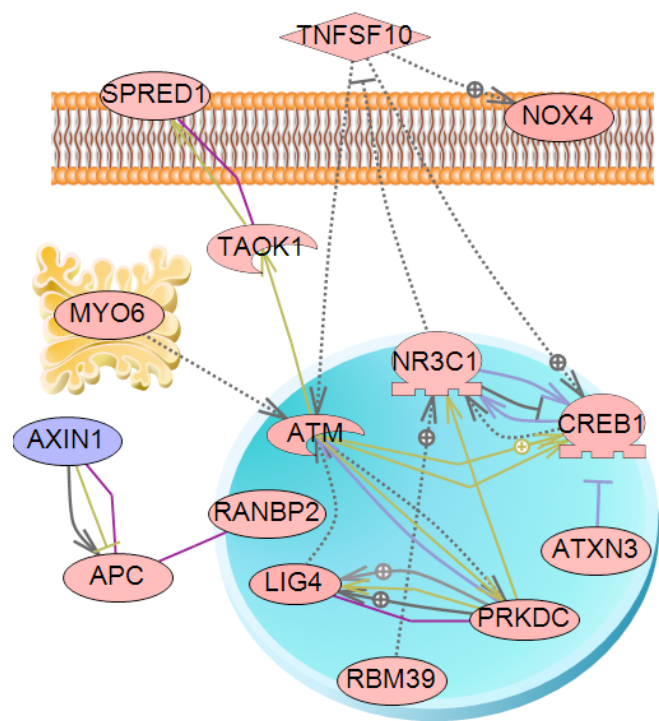

B Male Yellow

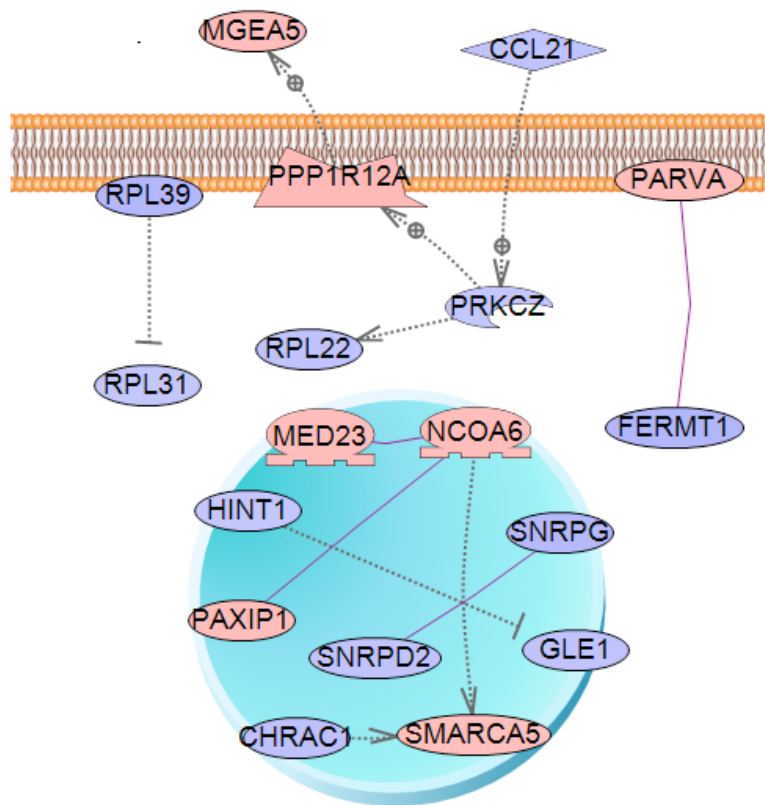

### C Male Brown

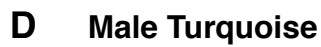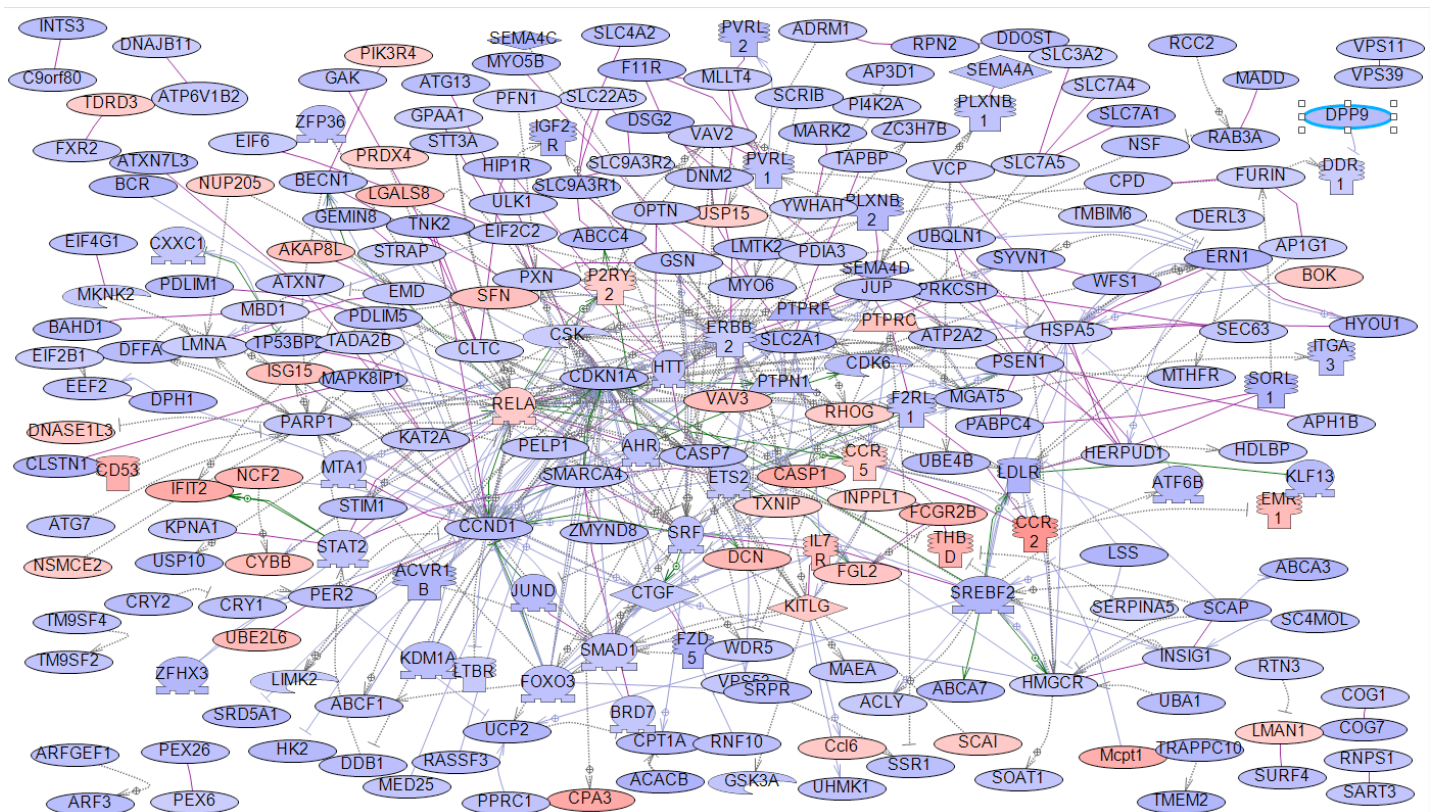

Supplement: Additional file 3 — Figure S3 - gene networks from gene modules. (a-d) Direct connection sub-networks for female and male modules: (a) female turquoise; (b) male yellow; (c) male brown; (d) male turquoise. Shape and color codes are the same as for Figure 5. [file gb-2012-13-10-r91-S3.pdf]
