# Supplementary material for: Epigenetic transgenerational inheritance of somatic transcriptomes and epigenetic control regions
Source: Genome Biol. 2012 Oct 3;13(10):R91. doi: 10.1186/gb-2012-13-10-r91 (PMC3491419; doi:10.1186/gb-2012-13-10-r91)

Supplemental Figure S4 (Color)

Tissue Gene Networks

A Female Heart

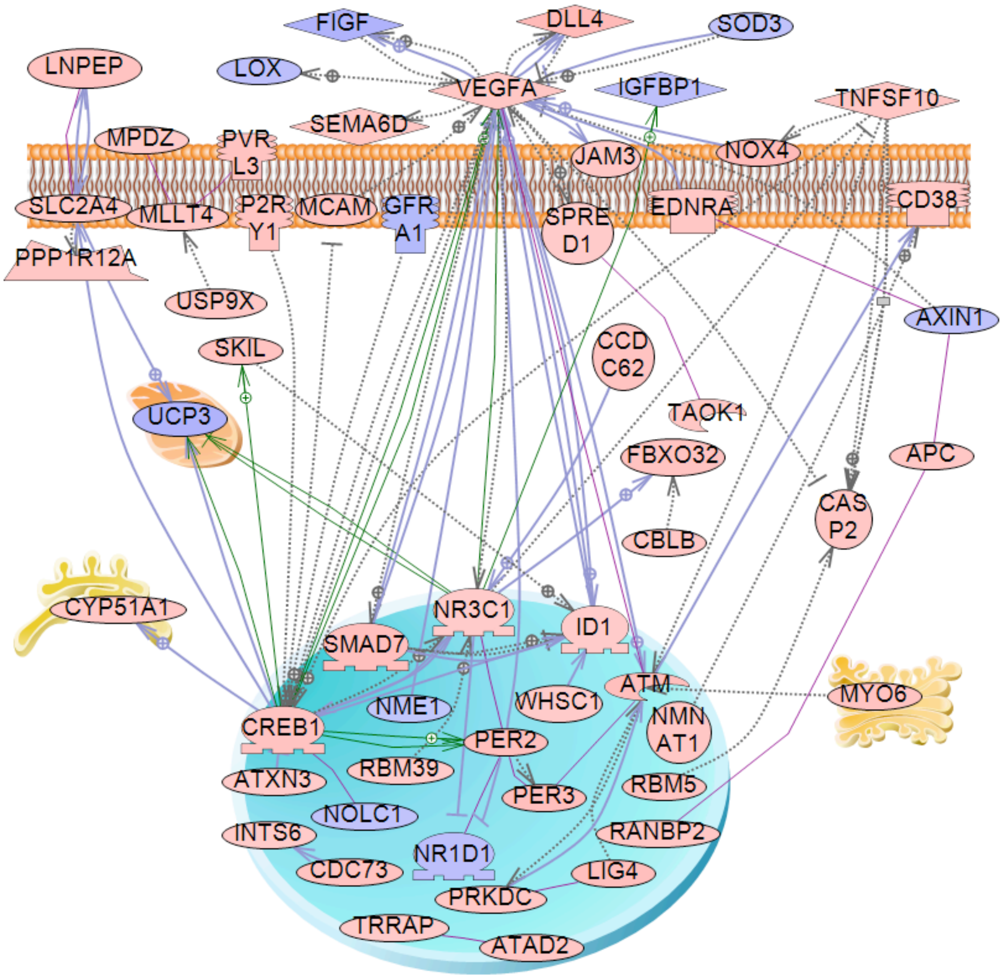

B Female Kidney

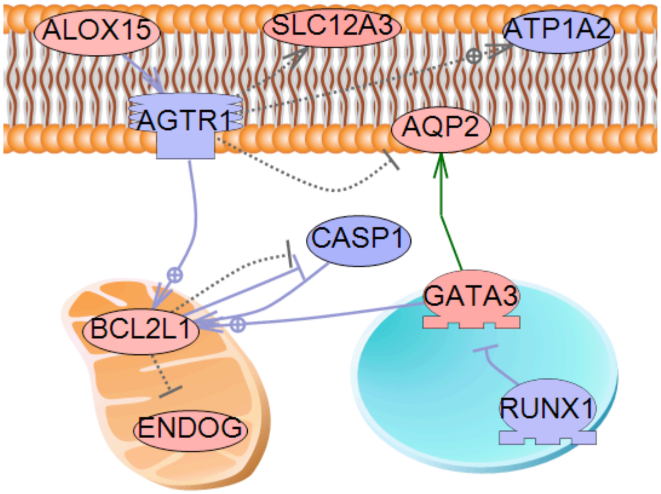

C Female Ovary

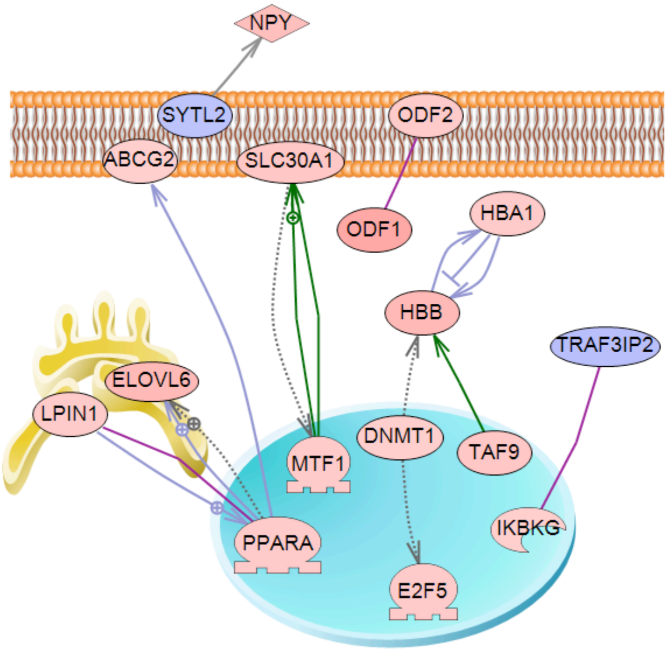

Supplemental Figure S4 cont.

D Female Uterus

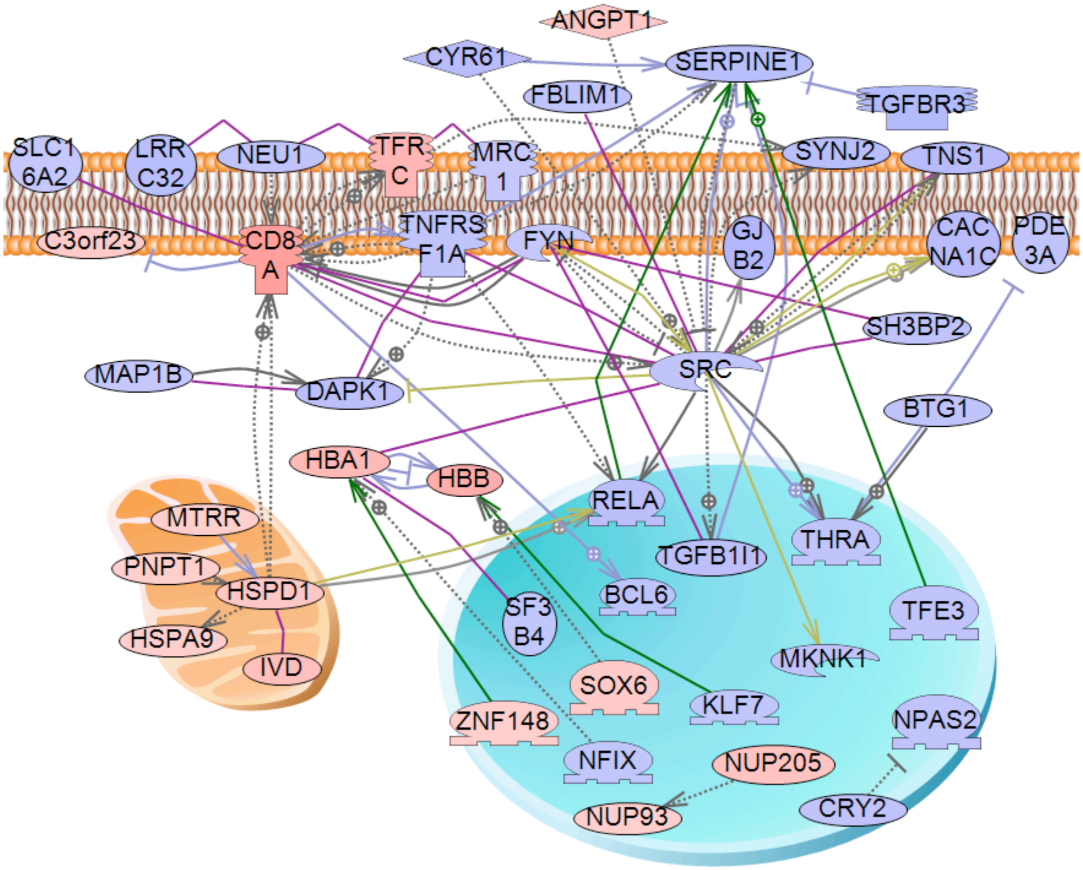

E Male Heart

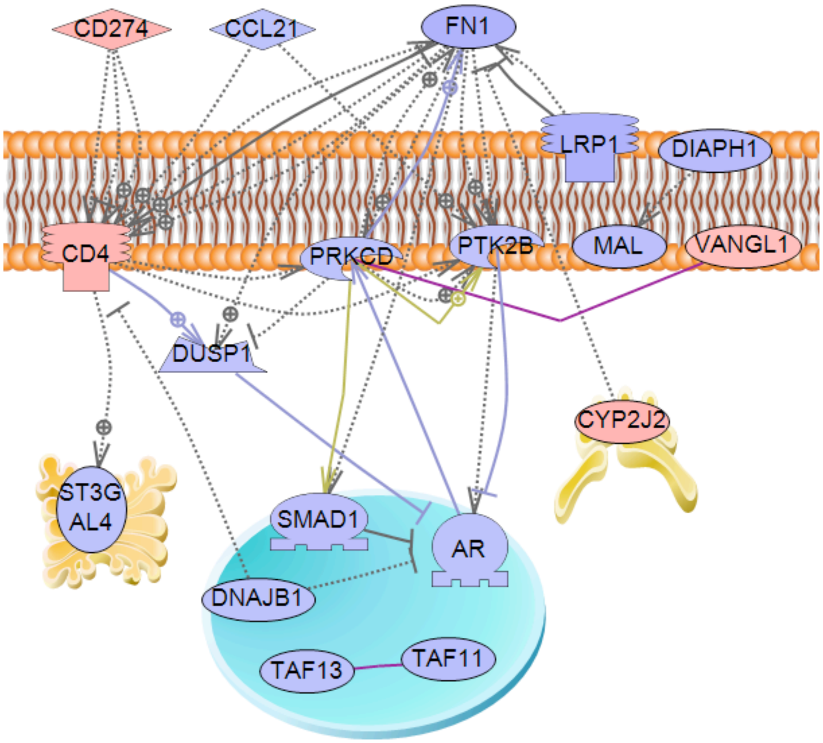

Supplemental Figure S4 cont.

F Male Kidney

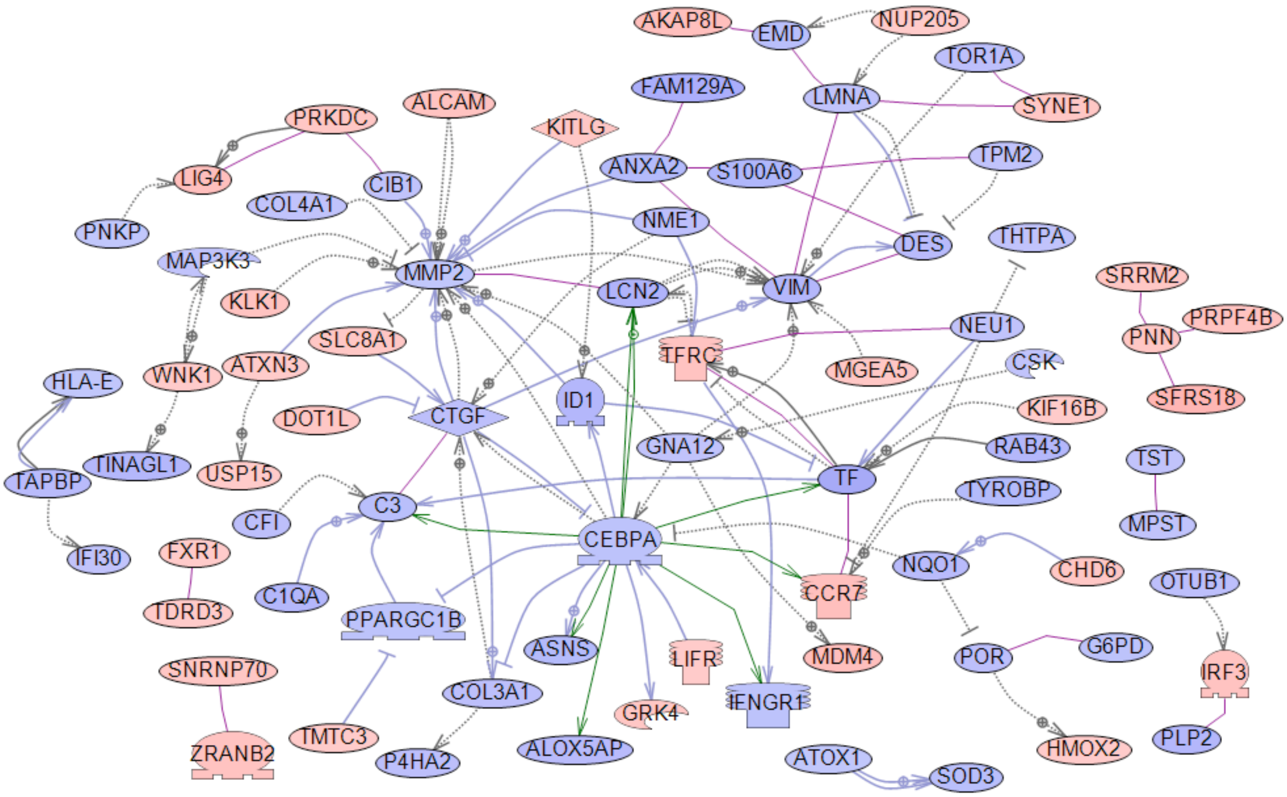

G Male Liver

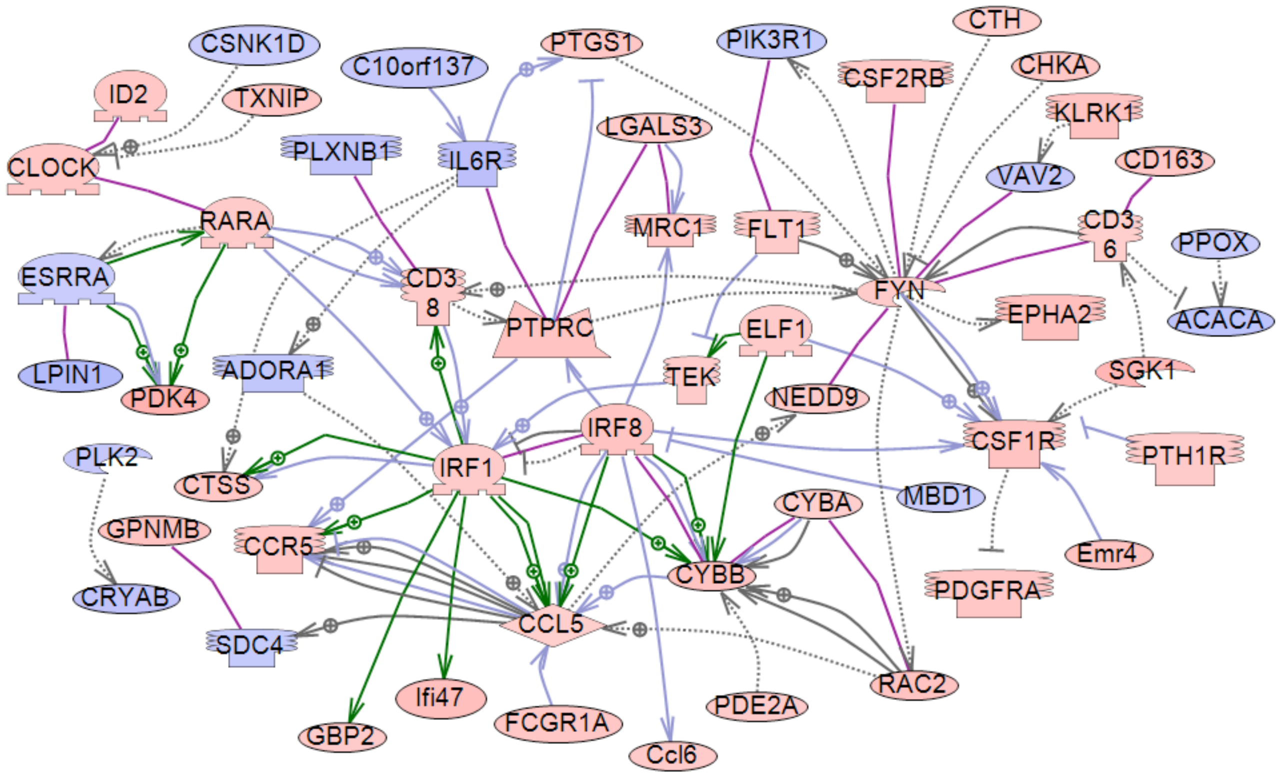

Supplement: Additional file 4 — Figure S4 - gene networks from signature lists. (a-d) Direct connection sub-networks for female and male tissue signature lists: (a) female heart; (b) female kidney; (c) male ovary; (d) uterus; (e) male heart; (f) male kidney; (g) male liver. Shape and color codes are the same as for Figure 5. [file gb-2012-13-10-r91-S4.pdf]
