# Supplementary material for: Epigenetic transgenerational inheritance of somatic transcriptomes and epigenetic control regions
Source: Genome Biol. 2012 Oct 3;13(10):R91. doi: 10.1186/gb-2012-13-10-r91 (PMC3491419; doi:10.1186/gb-2012-13-10-r91)
Supplement: Additional file 5 — Table S1 - differentially expressed genes in tissues. [file gb-2012-13-10-r91-S5.pdf]

Supplemental Table S1. Differentially Expressed Genes in F3 Generation Somatic Tissues

A. Female Heart

Gene Symbol	RefSeq/GenBank/ Ensemble Number	Affymetrix Probset ID	V/C	Mean Diff.	k.in	Mod	Gene Title
**APOPTOSIS**							
Casp2	NM_022522	10855020	1.24	39	1.8	blue	**caspase 2**
Tnfrsf21	NM_001108207	10926740	0.78	-27	0.0	grey	tumor necrosis factor receptor superfamily, member 21
Tnfsf10	NM_145681	10814528	1.23	51	3.9	turq	tumor necrosis factor (ligand) superfamily, member 10
Tnrc6b	NM_138845	10897821	1.22	37	0.0	grey	trinucleotide repeat containing 6B
**CELL CYCLE**							
Ccnl1	NM_053662	10823508	1.33	93	9.9	blue	cyclin L1
**Ccnl2**	**NM_001013094**	**10874708**	**1.27**	**111**	**16.8**	**blue**	**cyclin L2**
**Ccnt2**	**NM_001107171**	**10767280**	**1.48**	**146**	**14.2**	**blue**	**cyclin T2**
Cdc73	NM_001024769	10768305	1.30	100	6.1	turq	cell division cycle 73, Paf1/RNA polymerase II complex component, homolog (S. cerevisiae)
**CYTOSKELETON-ECM**							
Cldn16	NM_131905	10755027	0.74	-10	0.0	grey	claudin 16
Eml4	NM_001108008	10882543	1.23	61	5.5	turq	echinoderm microtubule associated protein like 4
Jam3	NM_001004269	10915910	1.23	35	2.1	turq	junctional adhesion molecule 3
Macf1	NM_001135758	10879555	1.36	132	10.2	turq	microtubule-actin crosslinking factor 1
**Mcam**	**NM_023983**	**10909446**	**1.35**	**35**	**1.8**	**l-grn**	**melanoma cell adhesion molecule**
Myh7b	NM_001107794	10841367	1.36	11	3.3	blue	myosin, heavy chain 7B, cardiac muscle, beta
Myo6	ENSRNOT00000016428	10911882	1.29	68	3.6	turq	myosin VI
Myo9a	NM_134335	10910577	1.22	45	12.5	turq	myosin IXA
Pcdh7	NM_001004087	10776978	1.50	37	6.2	blue	protocadherin 7
RGD1564809	ENSRNOT00000038184	10869125	1.25	24	3.0	red	similar to fibronectin type 3 and SPRY domain-containing protein
Smchd1	ENSRNOT00000019359	10930435	1.24	67	7.2	brwn	structural maintenance of chromosomes flexible hinge domain containing 1
Ttn	ENSRNOT00000032008	10846386	1.48	14	0.0	grey	titin
Zfc3h1	ENSRNOT00000058444	10895458	1.41	97	12.3	turq	zinc finger, C3H1-type containing
**DEVELOPMENT**							
Apc	NM_012499	10800844	1.23	47	5.5	turq	adenomatous polyposis coli
**Atm**	**NM_001106821**	**10917385**	**1.36**	**46**	**21.3**	**turq**	**ataxia telangiectasia mutated homolog (human)**
Atxn3	NM_021702	10891818	1.22	32	8.0	turq	ataxin 3
Bbs10	NM_001109286	10895394	1.29	17	6.4	turq	Bardet-Biedl syndrome 10
Diaph2	ENSRNOT00000034461	10934829	1.29	42	2.1	brwn	diaphanous homolog 2 (Drosophila)
Dmd	NM_012698	10933819	1.26	34	0.0	grey	dystrophin
**Dpy19l4**	**ENSRNOT00000037869**	**10875464**	**1.29**	**53**	**16.8**	**turq**	**dpy-19-like 4 (C. elegans)**
Emcn	NM_001004228	10819305	1.32	106	0.0	grey	endomucin
LOC686314	ENSRNOT00000040697	10799893	1.39	14	0.0	grey	similar to dachshund b
LOC687994	ENSRNOT00000030501	10867109	1.20	47	5.1	turq	similar to 82-kD FMRP Interacting Protein (chr5)
LOC687994	ENSRNOT00000030501	10736070	1.25	72	9.8	turq	similar to 82-kD FMRP Interacting Protein (chr10)
Nav3	NM_001191782	10902290	1.81	18	2.4	turq	neuron navigator 3
Ophn1	NM_001107848	10938556	1.36	39	0.0	grey	oligophrenin 1
Pcp4l1	NM_001126093	10769812	1.34	17	2.9	blue	Purkinje cell protein 4-like 1
Per2	NM_031678	10929842	1.38	20	1.0	mgnt	period circadian protein homolog 2 (Drosophila)
Per3	NM_023978	10881861	1.40	43	0.0	grey	period circadian protein homolog 3 (Drosophila)
Rapgef5	NM_001047915	10887774	1.39	49	0.9	l-cn	Rap guanine nucleotide exchange factor (GEF) 5
Rttn	NM_001170436	10803060	1.28	16	5.6	blue	rotatin
Sdccag1	NM_001170586	10890274	1.25	39	11.9	turq	serologically defined colon cancer antigen 1 (chr6)
**Sdccag1**	**NM_001170586**	**10719956**	**1.23**	**54**	**18.0**	**turq**	**serologically defined colon cancer antigen 1 (chr1)**
Sema6d	NM_001107768	10839327	1.26	15	0.0	grey	sema domain, transmembrane domain (TM), and cytoplasmic domain, (semaphorin) 6D
Setx	ENSRNOT00000017984	10834908	1.24	91	6.7	turq	senataxin
Skil	ENSRNOT00000013191	10822637	1.35	66	3.7	brwn	SKI-like oncogene
Spata5	NM_001108549	10815032	1.22	23	6.4	turq	spermatogenesis associated 5
Trpm7	AF375874	10849556	1.25	118	14.7	turq	transient receptor potential cation channel, subfamily M, member 7
Whsc1	NM_001191552	10704372	1.21	22	7.8	turq	Wolf-Hirschhorn syndrome candidate 1 (human)
**DNA REPAIR**							
Lig4	NM_001106095	10789670	1.40	81	15.7	turq	ligase IV, DNA, ATP-dependent
Rev1	NM_001108213	10927538	1.21	35	4.9	blue	REV1 homolog (S. cerevisiae)
**ELECTRON TRANSPORT**							
Cyp2d5	NM_173304	10905732	0.81	-11	0.0	grey	cytochrome P450, family 2, subfamily d, polypeptide 5
Cyp51	NM_012941	10853471	1.25	141	1.7	l-grn	cytochrome P450, family 51
Tmx4	NM_001100529	10850199	1.26	138	13.8	turq	thioredoxin-related transmembrane protein 4
Txndc16	ENSRNOT00000008372	10782804	1.21	25	3.2	turq	thioredoxin domain containing 16
Ucp3	NM_013167	10709083	0.59	-15	0.0	grey	uncoupling protein 3 (mitochondrial, proton carrier)

**EPIGENETICS**							
Chd6	NM_001107797	10851407	1.26	31	3.1	turq	chromodomain helicase DNA binding protein 6
Hist1h2ail	NM_001013056	10798455	1.24	83	6.1	turq	histone cluster 1, H2ai-like
Jmjd1c	NM_001191719	10832831	1.21	49	0.0	grey	jumonji domain containing 1C
Mettl11b	ENSRNOT00000039581	10769436	3.00	19	0.0	grey	methyltransferase like 11B
**Phf20l1**	**NM_001025702**	**10896862**	**1.30**	**83**	**16.6**	**turq**	**PHD finger protein 20-like 1**
RGD1561065	NM_001108253	10933017	1.20	17	0.0	grey	similar to mKIAA1111 protein
Syne1	AF452647	10717620	1.37	26	9.0	blue	spectrin repeat containing, nuclear envelope 1
Tmf1	NM_053671	10864351	1.24	43	4.3	brwn	TATA element modulatory factor 1
Tnp1	NM_017056	10928844	2.15	18	13.2	ylw	transition protein 1
**GOLGI APPARATUS**							
Ap4e1	NM_001191742	10839483	1.23	28	8.1	turq	adaptor-related protein complex 4, epsilon 1 subunit
Ogt	NM_017107	10934445	1.23	141	10.2	blue	O-linked N-acetylglucosamine (GlcNAc) transferase (UDP-N-acetylglucosamine:polypeptide-N-acetylglucosaminyl transferase)
**GROWTH FACTORS**							
Dll4	NM_001107760	10838813	1.44	22	0.0	grey	delta-like 4 (Drosophila)
Figf	NM_031761	10937725	0.57	-60	1.2	gr-yel	c-fos induced growth factor
Kng1	NM_012696	10751988	0.72	-42	0.0	grey	kininogen 1
Vegfa	NM_031836	10921772	1.27	136	3.1	blue	vascular endothelial growth factor A
**IMMUNE RESPONSE**							
**Cd38**	**NM_013127**	**10777232**	**1.27**	**19**	**1.5**	**l-cn**	**CD38 molecule**
Cfh	BC089845	10768236	0.78	-15	0.0	grey	complement factor H
Clk4	NM_001013041	10733321	1.31	92	8.0	blue	CDC like kinase 4
Fv1	NM_001014018	10706509	1.31	40	2.9	blue	Friend virus susceptibility 1
Pibf1	ENSRNOT00000036350	10781810	1.24	29	8.2	turq	progesterone immunomodulatory binding factor 1
Sigirr	NM_001024887	10726691	1.31	25	1.6	l-grn	single immunoglobulin and toll-interleukin 1 receptor (TIR) domain
Tmpo	NM_012887	10901823	1.29	62	5.1	blue	thymopoietin
Trim65	NM_001135714	10749124	1.21	12	0.8	l-grn	tripartite motif-containing 65
**METABOLISM & TRANSPORT**							
Aasdh	ENSRNOT00000002907	10772212	1.31	25	7.4	blue	aminoadipate-semialdehyde dehydrogenase
Arsk	NM_001047917	10820073	1.26	48	7.4	turq	arylsulfatase family, member K
Atad2	NM_001134879	10903864	1.25	15	2.4	turq	ATPase family, AAA domain containing 2
Atp6ap1l	NM_001191843	10820308	0.68	-14	0.0	grey	ATPase, H+ transporting, lysosomal accessory protein 1-like
Atp8a1	ENSRNOT00000041793	10772582	1.38	55	0.0	grey	ATPase, aminophospholipid transporter (APLT), class I, type 8A, member 1
Bche	NM_022942	10823678	1.51	50	7.8	turq	butyrylcholinesterase
Cacna2d1	NM_012919	10860374	1.32	32	0.0	grey	calcium channel, voltage-dependent, alpha2/delta subunit 1
Ces3	NM_133295	10931271	0.81	-58	0.7	red	carboxylesterase 3
Clcn5	NM_017106	10932584	1.25	34	0.0	grey	chloride channel 5
Gba2	NM_001013091	10876396	1.22	15	4.6	blue	glucosidase beta 2
Hlcs	ENSRNOT00000002291	10753247	0.80	-21	0.0	grey	holocarboxylase synthetase (biotin-(proprionyl-Coenzyme A-carboxylase (ATP-hydrolysing)) ligase)
Impdh1	NM_001108619	10861452	0.80	-18	0.0	grey	IMP (inosine monophosphate) dehydrogenase 1
Inpp4b	ENSRNOT00000024755	10810407	1.27	12	0.0	grey	inositol polyphosphate-4-phosphatase, type II
Lox	NM_017061	10804463	0.76	-19	1.5	l-cn	lysyl oxidase
Mat2a	NM_134351	10863221	1.25	300	8.0	turq	methionine adenosyltransferase II, alpha (chr4)
Mat2a	NM_134351	10767001	1.26	359	7.9	turq	methionine adenosyltransferase II, alpha (chr13)
Mthfd2	NM_001109398	10863512	1.26	16	2.7	turq	methylenetetrahydrofolate dehydrogenase (NADP+ dependent) 2, methenyltetrahydrofolate cyclohydrolase
Nme1	NM_138548	10746276	0.83	-33	0.0	grey	non-metastatic cells 1, protein (NM23A) expressed in
Nmnat1	NM_001037556	10881771	1.24	15	0.7	l-yel	nicotinamide nucleotide adenylyltransferase 1
Nox4	NM_053524	10708485	1.45	28	1.8	turq	NADPH oxidase 4
Nupl1	NM_139091	10784307	1.30	43	0.0	grey	nucleoporin like 1
Ogdhl	NM_001106062	10786774	1.23	11	0.0	grey	oxoglutarate dehydrogenase-like
Pigg	ENSRNOT00000033973	10775063	1.27	25	0.8	l-cn	phosphatidylinositol glycan anchor biosynthesis, class G
Pus3	NM_001108134	10908990	1.27	20	6.6	brwn	pseudouridylate synthase 3
Pusl1	NM_001108699	10882238	1.26	22	0.0	grey	pseudouridylate synthase-like 1
Rpgr	NM_001127601	10932343	0.78	-18	0.0	grey	retinitis pigmentosa GTPase regulator
Slc25a36	ENSRNOT00000018433	10810551	1.21	84	6.1	turq	solute carrier family 25, member 36
Slc28a2	NM_031664	10849275	1.28	74	0.0	grey	solute carrier family 28 (sodium-coupled nucleoside transporter), member 2
Slc2a4	NM_012751	10744324	1.25	16	0.0	grey	solute carrier family 2 (facilitated glucose transporter), member 4
Slc30a6	NM_001106708	10888565	1.24	34	8.2	turq	solute carrier family 30 (zinc transporter), member 6
Slc36a4	NM_001108127	10908111	1.28	32	5.1	turq	solute carrier family 36 (proton/amino acid symporter), member 4
Slc47a1	NM_001014118	10743387	1.27	14	2.6	turq	solute carrier family 47, member 1
Slc4a7	NM_058211	10782533	1.33	40	0.0	grey	solute carrier family 4, sodium bicarbonate cotransporter, member 7
Slc5a3	NM_053715	10750282	1.36	43	0.0	grey	solute carrier family 5 (inositol transporters), member 3
Slc7a6os	NM_139328	10810811	1.20	14	0.7	l-yel	solute carrier family 7, member 6 opposite strand
Sod3	NM_012880	10777108	0.80	-122	1.8	turq	superoxide dismutase 3, extracellular
Stard13	NM_001109060	10756289	1.28	27	3.7	blue	StAR-related lipid transfer (START) domain containing 13
Tnpo1	ENSRNOT00000020543	10820795	1.25	166	11.4	turq	transportin 1

Ugt2b34	NM_001191676	10771993	0.78	-11	0.0	grey	UDP glucuronosyltransferase 2 family, polypeptide B34
**PROTEOLYSIS**							
Adam19	NM_001160228	10732966	0.82	-13	0.0	grey	a disintegrin and metallopeptidase domain 19 (meltrin beta)
Adam4l1	NM_001109277	10890962	1.31	12	0.0	grey	a disintegrin and metallopeptidase domain 4-like 1
Lnpep	NM_001113403	10718324	1.32	201	8.0	turq	leucyl/cystinyl aminopeptidase
**LOC360990**	**ENSRNOT00000038258**	**10774497**	**1.21**	**89**	**21.8**	**turq**	**similar to ubiquitin specific protease 34**
Mmp11	NM_012980	10829630	0.83	-14	0.0	grey	matrix metallopeptidase 11
Pcsk7	NM_019246	10909704	1.25	24	1.5	l-grn	proprotein convertase subtilisin/kexin type 7
Prkrip1	NM_001098793	10757555	1.23	21	0.0	grey	Prkr interacting protein 1 (IL11 inducible)
Prss23	NM_001007691	10723576	0.82	-67	0.8	brwn	protease, serine, 23
**RGD1306353**	**ENSRNOT00000047731**	**10784101**	**1.34**	**61**	**17.2**	**turq**	**similar to Zinc finger protein 198 (Fused in myeloproliferative disorders protein) (Rearranged in atypical myeloproliferative disorder protein)**
Rnf139	NM_001127545	10896748	1.22	80	6.4	turq	ring finger protein 139
Rnf160	NM_001024235	10752865	1.24	57	10.4	turq	ring finger protein 160
Rnf213	ENSRNOT00000004904	10739992	1.47	40	0.0	grey	ring finger protein 213
Uba6	NM_001107213	10772072	1.23	108	9.3	turq	ubiquitin-like modifier activating enzyme 6
Usp9x	NM_001135893	10936775	1.23	251	15.0	turq	ubiquitin specific peptidase 9, X-linked
Vps13a	NM_001100975	10729124	1.32	118	6.3	turq	vacuolar protein sorting 13 homolog A (S. cerevisiae)
**RECEPTORS & BINDING PROTEINS**							
**Abcg3l2**	**NM_001014133**	**10775283**	**1.23**	**57**	**16.6**	**turq**	**ATP-binding cassette, sub-family G (WHITE), member 3-like 2**
Apbb3	NM_053957	10803961	1.25	18	11.4	blue	amyloid beta (A4) precursor protein-binding, family B, member 3
Ednra	NM_012550	10807022	1.27	35	0.0	grey	endothelin receptor type A
Gfra1	NM_012959	10731075	0.68	-15	0.0	grey	GDNF family receptor alpha 1
Igfbp1	NM_013144	10774141	0.75	-19	0.0	grey	insulin-like growth factor binding protein 1
Osbpl8	BC168899	10895366	1.27	129	2.6	turq	oxysterol binding protein-like 8
Ppfibp2	NM_001100582	10709691	1.32	40	0.0	grey	PTPRF interacting protein, binding protein 2 (liprin beta 2)
Ptbp2	NM_001005555	10826418	1.26	53	8.8	blue	polypyrimidine tract binding protein 2
Pvrl3	NM_001105883	10750982	1.33	55	6.9	turq	poliovirus receptor-related 3
**SIGNALING**							
Akap13	NM_001106271	10707953	1.35	114	7.5	blue	A kinase (PRKA) anchor protein 13
Alpk1	ENSRNOT00000030798	10826680	1.28	20	1.6	turq	alpha-kinase 1
Arl6ip1	AB190510	10725172	1.39	135	10.3	blue	ADP-ribosylation factor-like 6 interacting protein 1
Arrdc4	NM_001047853	10722720	0.75	-45	0.0	grey	arrestin domain containing 4
Ascc3	BC169103	10830699	1.23	74	9.1	turq	activating signal cointegrator 1 complex subunit 3
Axin1	NM_024405	10732553	0.81	-25	2.9	turq	axin 1
Creb1	NM_134443	10923929	1.30	47	6.3	turq	cAMP responsive element binding protein 1
Dtx4	NM_001047855	10729009	0.73	-19	0.8	brwn	deltex homolog 4 (Drosophila)
Elmod2	NM_001109506	10801518	1.26	36	14.7	turq	ELMO/CED-12 domain containing 2 (chr18)
**Elmod2**	**NM_001109506**	**10806873**	**1.27**	**42**	**18.0**	**turq**	**ELMO/CED-12 domain containing 2 (chr19)**
G3bp2	NM_001013989	10771720	1.21	170	7.1	brwn	GTPase activating protein (SH3 domain) binding protein 2
Garnl1	NM_020083	10890031	1.21	80	12.4	turq	GTPase activating Rap/RanGAP domain-like 1
Gpr89	NM_001139486	10825213	1.21	121	0.0	grey	G protein-coupled receptor 89
Grtp1	ENSRNOT00000057740	10789486	1.76	13	4.0	blue	growth hormone regulated TBC protein 1
LOC497978	BC110049	10746158	1.38	23	0.0	grey	similar to diacylglycerol kinase epsilon
Map4k3	NM_133407	10888056	1.26	45	4.7	blue	mitogen-activated protein kinase kinase kinase kinase 3
Mpdz	NM_019196	10877698	1.22	38	15.2	turq	multiple PDZ domain protein
P2ry1	NM_012800	10815652	1.33	31	0.0	grey	purinergic receptor P2Y, G-protein coupled, 1
Pik3c2a	NM_001108500	10725114	1.45	109	7.2	turq	phosphoinositide-3-kinase, class 2, alpha polypeptide
Pik3ip1	NM_001017453	10773695	1.36	25	1.2	blue	phosphoinositide-3-kinase interacting protein 1
Ppp1r12a	NM_053890	10895310	1.23	51	9.7	blue	protein phosphatase 1, regulatory (inhibitor) subunit 12A
Ptpn4	NM_001100479	10767132	1.38	96	11.3	turq	protein tyrosine phosphatase, non-receptor type 4
Ptprd	ENSRNOT00000066046	10869616	1.41	165	0.0	grey	protein tyrosine phosphatase, receptor type, D
Ranbp2	NM_001191604	10833492	1.23	234	15.6	turq	RAN binding protein 2
Rap2a	NM_053741	10782056	1.20	11	0.5	l-grn	RAS related protein 2a
Rasal2	NM_001107188	10769047	1.28	26	4.4	brwn	RAS protein activator like 2
RGD1562230	BC166848	10829933	1.28	16	0.0	grey	similar to catenin alpha 3
Rhoj	NM_001008320	10885299	0.78	-62	0.9	gr60	ras homolog gene family, member J
Shank3	NM_021676	10898622	1.27	10	0.0	grey	SH3 and multiple ankyrin repeat domains 3
Smad7	NM_030858	10802734	1.41	23	0.0	grey	SMAD family member 7
Smpdl3b	NM_001025737	10880339	1.35	17	1.5	l-grn	sphingomyelin phosphodiesterase, acid-like 3B
Spred1	NM_001047089	10838584	1.20	45	2.5	turq	sprouty-related, EVH1 domain containing 1
Taok1	NM_173327	10736420	1.23	148	13.5	turq	TAO kinase 1
Tnik	NM_001106422	10814594	1.25	12	1.3	l-cn	TRAF2 and NCK interacting kinase
**TRANSCRIPTION**							
Ankrd26	ENSRNOT00000056301	10864979	1.30	15	1.0	purple	ankyrin repeat domain 26
Baz2b	NM_001108260	10845470	1.21	29	4.0	turq	bromodomain adjacent to zinc finger domain, 2B
Cblb	NM_133601	10753747	1.25	25	4.6	blue	Cas-Br-M (murine) ecotropic retroviral transforming sequence b
Ccdc12	NM_001108783	10913714	0.78	-29	1.8	blue	coiled-coil domain containing 12
Ccdc62	NM_001134766	10761792	1.31	11	12.3	blue	coiled-coil domain containing 62

**Ddx17**	**NM_001015018**	**10897683**	**1.34**	**233**	**16.1**	**blue**	**DEAD (Asp-Glu-Ala-Asp) box polypeptide 17**
Fam134b	NM_001034912	10813934	1.20	57	0.7	l-cn	family with sequence similarity 134, member B
Fbxo32	NM_133521	10903896	1.33	78	0.0	grey	F-box protein 32
Foxp2	ENSRNOT00000034078	10853783	1.71	14	3.9	blue	forkhead box P2
Id1	NM_012797	10840890	1.20	15	0.0	grey	inhibitor of DNA binding 1
Ier2	NM_001009541	10806685	0.71	-15	0.0	grey	immediate early response 2
Kank3	NM_001108989	10894380	1.31	11	1.9	turq	KN motif and ankyrin repeat domains 3
LOC100361581	ENSRNOT00000040584	10833564	0.67	-29	0.0	grey	homeobox protein-like
Mcc	NM_001170534	10804316	1.22	33	0.6	l-grn	mutated in colorectal cancers
Mllt4	NM_013217	10703189	1.24	47	0.0	grey	myeloid/lymphoid or mixed-lineage leukemia (trithorax homolog, Drosophila); translocated to, 4
Ncbp1	NM_001014785	10868829	1.22	73	5.7	brwn	nuclear cap binding protein subunit 1, 80kDa
Nfxl1	NM_001170438	10772466	1.30	26	12.0	blue	nuclear transcription factor, X-box binding-like 1
Nolc1	NM_022869	10715721	0.82	-20	0.0	grey	nucleolar and coiled-body phosphoprotein 1
Nr1d1	NM_001113422	10746955	0.77	-70	3.8	brwn	nuclear receptor subfamily 1, group D, member 1
Nr3c1	NM_012576	10804132	1.22	139	13.3	turq	nuclear receptor subfamily 3, group C, member 1
Rb1cc1	NM_001107901	10875183	1.29	104	11.5	turq	RB1-inducible coiled-coil 1
Rel	ENSRNOT00000065110	10778763	1.32	25	0.0	grey	v-rel reticuloendotheliosis viral oncogene homolog (avian)
RGD1564836	ENSRNOT00000038940	10937775	0.79	-29	7.3	turq	similar to HMGA1b
RGD1565257	NM_001134550	10836650	1.29	133	7.1	turq	similar to zinc finger protein 650
Son	NM_001170327	10750219	1.22	147	10.8	turq	Son DNA binding protein
Spty2d1	ENSRNOT00000008420	10862747	1.35	27	0.0	grey	SPT2, Suppressor of Ty, domain containing 1 (S. cerevisiae)
Tet2	ENSRNOT00000046775	10826902	1.20	21	0.9	turq	tet oncogene family member 2
Trrap	NM_001105907	10760210	1.20	15	0.0	grey	transformation/transcription domain-associated protein
**Zcchc7**	**NM_001106658**	**10868662**	**1.40**	**141**	**15.6**	**blue**	**zinc finger, CCHC domain containing 7**
Zdhhc17	NM_001039340	10902294	1.29	70	8.7	turq	zinc finger, DHHC-type containing 17
Zfp386	NM_019620	10887669	1.33	38	9.2	turq	zinc finger protein 386 (Kruppel-like)
**Zmym2**	**ENSRNOT00000011684**	**10780737**	**1.23**	**107**	**16.6**	**turq**	**zinc finger, MYM-type 2**
Znf23	NM_001107428	10807828	1.20	18	5.3	blue	zinc finger protein 23 (KOX 16)
Znf292	NM_001008879	10875941	1.30	95	12.0	turq	zinc finger protein 292
Zswim6	ENSRNOT00000052233	10821218	1.26	14	0.0	grey	zinc finger, SWIM-type containing 6
LOC100360593	ENSRNOT00000025586	10720126	1.67	57	6.1	blue	zinc finger protein 59
**TRANSLATION & PROTEIN MODIFICATION**							
**Cpsf6**	**NM_001106785**	**10902552**	**1.20**	**42**	**15.0**	**blue**	**cleavage and polyadenylation specific factor 6**
Eif1	NM_001105837	10738170	0.76	-42	1.7	blue	eukaryotic translation initiation factor 1
Fusip1	NM_001025738	10872929	1.23	62	8.6	blue	FUS interacting protein (serine-arginine rich) 1
Ints6	ENSRNOT00000050190	10784416	1.21	16	3.5	blue	integrator complex subunit 6
Mrps14	NM_001105963	10764983	0.83	-41	0.0	grey	mitochondrial ribosomal protein S14
Mrps17	NM_001105923	10758007	0.83	-52	0.0	grey	mitochondrial ribosomal protein S17
Papolg	NM_001107244	10778773	1.20	28	6.1	turq	poly(A) polymerase gamma
Pcf11	ENSRNOT00000013552	10723669	1.20	36	4.9	turq	PCF11, cleavage and polyadenylation factor subunit, homolog (S. cerevisiae)
Prpf4b	NM_001011923	10798074	1.21	66	11.3	blue	PRP4 pre-mRNA processing factor 4 homolog B (yeast)
Rbm39	NM_001013207	10851173	1.22	325	14.7	turq	RNA binding motif protein 39
Rbm5	NM_001100548	10920170	1.33	93	11.3	blue	RNA binding motif protein 5
**Rpl7a**	**NM_001114391**	**10753737**	**1.67**	**62**	**16.1**	**blue**	**ribosomal protein L7a (chr11)**
**Rpl7a**	**NM_001114391**	**10797321**	**1.56**	**48**	**18.4**	**blue**	**ribosomal protein L7a (chr17)**
Sf3a2	NM_001011986	10900411	0.73	-14	0.0	grey	splicing factor 3a, subunit 2
Sfrs18	NM_001025274	10867882	1.28	78	12.5	blue	splicing factor, arginine/serine-rich 18
**Sfrs7**	**NM_001039035**	**10887981**	**1.31**	**119**	**13.9**	**blue**	**splicing factor, arginine/serine-rich 7, 35kDa**
Sh3bp5l	NM_001127581	10733829	1.21	16	2.6	mgnt	SH3 binding domain protein 5 like
Snrnp48	NM_001106107	10797956	1.24	31	5.9	turq	small nuclear ribonucleoprotein 48k (U11/U12)
Snrnp70	NM_001108483	10721802	1.26	122	4.6	blue	small nuclear ribonucleoprotein 70 (U1)
Unc45a	NM_001037647	10723039	1.35	34	0.0	grey	unc-45 homolog A (C. elegans)
Ythdc2	ENSRNOT00000060098	10801506	1.20	60	8.9	turq	YTH domain containing 2
**MISCELLANEOUS & UNKNOWN**							
Birc6	NM_001170596	10888460	1.24	118	15.2	turq	baculoviral IAP repeat-containing 6
Brwd3	ENSRNOT00000050336	10939018	1.30	33	14.5	turq	bromodomain and WD repeat domain containing 3
Fam118b	BC098017	10916052	1.24	19	5.2	turq	family with sequence similarity 118, member B
Fam126b	BC097998	10928275	1.32	76	13.2	turq	family with sequence similarity 126, member B
Fam135a	NM_001134597	10927055	1.27	76	7.0	turq	family with sequence similarity 135, member A
LOC314016	ENSRNOT00000006122	10884031	1.31	38	0.0	grey	similar to mKIAA0230 protein
LOC367289	ENSRNOT00000067589	10928584	1.24	62	3.8	turq	similar to CG4699-PA, isoform A
LOC499120	U78125	10705806	1.33	13	2.3	blue	hypothetical protein LOC499120
LOC500625	ENSRNOT00000006877	10883443	1.42	65	15.2	turq	hypothetical protein LOC500625
**LOC688452**	**FM212919**	**10703432**	**2.02**	**78**	**13.8**	**blue**	**hypothetical protein LOC688452**
MGC94190	BC090318	10761329	0.82	-50	6.1	turq	similar to 0610007L01Rik protein
MGC94282	NR_027366	10865838	0.67	-11	2.2	blue	similar to 5930416I19Rik protein
RGD1308023	ENSRNOT00000015637	10850492	1.28	45	1.3	l-cn	similar to CG5521-PA
**RGD1308143**	**BC083847**	**10925725**	**1.38**	**127**	**18.9**	**turq**	**similar to D330021B20 protein**

**RGD1308297**	**BC082039**	**10785745**	**1.26**	**70**	**14.8**	**blue**	**similar to CG10084-PA**
RGD1308616	ENSRNOT00000039235	10879244	1.34	30	6.8	blue	similar to KIAA0467 protein
RGD1359529	NM_001014193	10872829	1.44	260	7.0	blue	similar to chromosome 1 open reading frame 63
RGD1559496	NM_001106199	10808874	1.24	21	1.3	l-cn	similar to hypothetical protein
RGD1564317	XM_579774	10752970	0.80	-16	0.0	grey	RGD1564317
RGD1565690	ENSRNOT00000039316	10862818	1.33	34	0.0	grey	similar to mKIAA2027 protein
Tmem126b	NM_001106280	10723644	1.21	31	5.4	turq	transmembrane protein 126B
Tmem57	NM_001025699	10880564	1.32	51	0.0	grey	transmembrane protein 57
Tnrc6a	NM_001107549	10710695	1.21	48	1.4	cyan	trinucleotide repeat containing 6a
**Ttc14**	**NM_001107666**	**10814817**	**1.33**	**66**	**16.6**	**turq**	**tetratricopeptide repeat domain 14**
Ttc17	BC168714	10847668	1.21	38	0.0	grey	tetratricopeptide repeat domain 17
**EST's**							
LOC100363003	ENSRNOT00000066200	10794752	0.82	-66	0.0	grey	cDNA sequence BC034664-like
LOC100364912	ENSRNOT00000006697	10901039	1.30	218	13.7	turq	rCG29233-like
LOC100365715	ENSRNOT00000001232	10915310	1.21	12	3.9	turq	rCG31761-like
LOC100366213	XR_085848	10879660	1.25	26	3.8	turq	hypothetical LOC100366213
LOC290577	ENSRNOT00000060770	10786995	1.29	166	11.7	turq	hypothetical LOC290577
RGD1307235	NM_001134546	10763237	1.29	49	1.0	l-cn	similar to RIKEN cDNA 2310035C23
RGD1307897	BC091339	10884868	1.34	15	2.5	turq	similar to RIKEN cDNA C730036B14 gene
RGD1308923	NM_001108698	10874585	0.81	-15	0.0	grey	LOC362678
RGD1309374	ENSRNOT00000020492	10703327	1.25	20	10.6	blue	LOC361485
RGD1310507	NM_001047891	10919658	1.20	22	0.0	grey	similar to RIKEN cDNA 1300017J02
RGD1560108	NM_001109161	10713518	1.38	27	2.5	blue	similar to RIKEN cDNA 2700081O15
Mir7a-1	NR_031795	10793838	1.23	17	3.5	blue	microRNA mir-7a-1
	BC090353	10728028	1.89	2083	11.0	blue	
	ENSRNOT00000000260	10746156	0.74	-15	0.0	grey	
	ENSRNOT00000001815	10760818	1.25	23	8.8	blue	
	ENSRNOT00000002231	10753300	1.26	54	6.9	blue	
	**ENSRNOT00000006727**	**10777290**	**1.27**	**52**	**1.8**	**l-cn**	
	ENSRNOT00000012501	10911158	1.20	54	3.0	turq	
	ENSRNOT00000012671	10733624	1.29	86	7.5	turq	
	ENSRNOT00000015387	10815763	1.31	85	0.6	l-grn	
	ENSRNOT00000015827	10883321	1.22	26	0.0	grey	
	ENSRNOT00000016227	10916791	1.26	16	12.6	blue	
	ENSRNOT00000020883	10823254	1.34	71	7.1	blue	
	**ENSRNOT00000021181**	**10821771**	**1.20**	**87**	**17.5**	**turq**	
	**ENSRNOT00000022344**	**10814940**	**1.25**	**151**	**16.2**	**turq**	
	ENSRNOT00000024516	10729852	0.82	-44	2.2	gr-yel	
	ENSRNOT00000027019	10801254	1.45	18	5.5	blue	
	ENSRNOT00000030200	10840224	1.33	11	6.2	blue	
	ENSRNOT00000031814	10925923	0.83	-10	1.1	brwn	
	ENSRNOT00000035247	10755897	1.28	48	15.6	turq	
	ENSRNOT00000035876	10812834	1.51	19	2.9	turq	
	ENSRNOT00000035991	10846346	1.52	14	0.0	grey	
	ENSRNOT00000036063	10923727	1.43	128	8.9	turq	
	ENSRNOT00000039392	10801071	0.80	-17	2.7	turq	
	**ENSRNOT00000039420**	**10911476**	**1.75**	**45**	**14.6**	**blue**	
	ENSRNOT00000039879	10897891	0.83	-33	0.0	grey	
	ENSRNOT00000039948	10767044	0.75	-116	0.0	grey	
	ENSRNOT00000041674	10731416	0.81	-10	0.0	grey	
	ENSRNOT00000043970	10934560	0.75	-13	2.6	blue	
	ENSRNOT00000044552	10822904	1.22	73	0.0	grey	
	ENSRNOT00000048364	10900112	1.38	22	0.0	grey	
	ENSRNOT00000051851	10939758	0.78	-13	5.4	turq	
	ENSRNOT00000052461	10939899	0.82	-11	0.9	gr-yel	
	ENSRNOT00000052487	10827448	1.23	13	0.0	grey	
	ENSRNOT00000052610	10910770	1.28	28	8.3	blue	
	ENSRNOT00000052659	10784223	1.46	38	6.0	blue	
	ENSRNOT00000052722	10737647	1.20	10	7.6	blue	
	ENSRNOT00000052997	10908106	1.41	50	3.1	blue	
	ENSRNOT00000053086	10811999	0.75	-268	4.1	gr-yel	
	ENSRNOT00000053371	10863267	1.22	17	5.0	blue	
	ENSRNOT00000053846	10702161	1.47	12	0.0	grey	
	ENSRNOT00000053919	10755129	0.77	-331	3.1	gr-yel	
	ENSRNOT00000054058	10910768	1.25	13	5.2	blue	
	ENSRNOT00000054087	10864356	1.36	21	0.0	grey	
	ENSRNOT00000054304	10716148	1.37	20	2.3	blue	
	ENSRNOT00000055921	10849643	1.33	12	2.6	blue	
	ENSRNOT00000057090	10857382	1.20	35	0.0	grey	

	ENSRNOT00000057159	10766878	2.23	72	8.4	blue	
	ENSRNOT00000059520	10772514	0.77	-21	0.0	grey	
	ENSRNOT00000059561	10831804	0.82	-49	0.8	l-grn	
	ENSRNOT00000060917	10932310	1.20	18	2.3	blue	
	ENSRNOT00000063495	10892977	1.52	32	12.8	blue	
	**ENSRNOT00000065961**	**10801606**	**1.29**	**198**	**20.2**	**turq**	
	ENSRNOT00000069287	10877567	0.74	-18	1.2	gr-yel	
	ENSRNOT00000069910	10892067	0.83	-12	1.7	gr-yel	
	ENSRNOT00000070202	10834604	0.67	-43	1.0	l-cn	
	FJ807484	10933538	1.23	23	4.5	turq	
	FQ222240	10714078	1.21	12	4.6	turq	
	FQ222682	10722718	0.61	-182	0.0	grey	
	FQ233900	10765040	0.69	-128	0.0	grey	
	GENSCAN00000039416	10704655	0.80	-30	0.0	grey	
	---	10802189	1.58	61	1.7	black	
	---	10700479	0.81	-106	0.7	black	
	**---**	**10700930**	**1.61**	**268**	**14.8**	**blue**	
	---	10864272	1.66	18	13.6	blue	
	---	10701432	2.11	51	13.6	blue	
	---	10815943	1.87	61	12.8	blue	
	---	10700873	1.90	2161	11.7	blue	
	---	10700829	2.02	67	11.2	blue	
	---	10700478	3.12	130	9.8	blue	
	---	10700654	2.17	52	9.5	blue	
	---	10717953	1.32	25	9.4	blue	
	---	10700423	1.82	79	9.3	blue	
	---	10701535	3.33	279	9.3	blue	
	---	10701243	1.84	103	9.0	blue	
	---	10700950	2.20	61	8.5	blue	
	---	10700545	1.92	39	8.5	blue	
	---	10701137	2.63	107	7.6	blue	
	---	10700397	2.21	83	7.4	blue	
	---	10700826	1.54	21	7.1	blue	
	---	10701418	2.22	74	6.6	blue	
	---	10700464	2.91	95	6.4	blue	
	---	10700219	1.68	20	6.1	blue	
	---	10701140	2.59	196	6.1	blue	
	---	10701580	1.69	25	6.0	blue	
	---	10862453	2.01	283	5.5	blue	
	---	10701393	2.22	44	5.2	blue	
	---	10701233	2.53	60	4.7	blue	
	---	10700657	2.33	66	4.6	blue	
	---	10700535	2.15	65	4.4	blue	
	---	10700759	2.35	65	4.3	blue	
	---	10700127	1.74	30	3.7	blue	
	---	10700727	1.60	18	3.1	blue	
	---	10812357	1.54	16	3.1	blue	
	---	10700343	1.36	13	2.6	blue	
	---	10700898	1.43	21	2.6	blue	
	---	10700460	1.45	20	2.4	blue	
	---	10799156	1.20	25	1.0	blue	
	---	10701059	0.58	-19	0.0	grey	
	---	10700486	0.79	-14	0.0	grey	
	---	10701218	1.63	12	0.0	grey	
	---	10700556	0.75	-217	0.0	grey	
	---	10700623	0.82	-101	0.0	grey	
	---	10700089	0.44	-27	0.0	grey	
	---	10700908	0.68	-19	0.0	grey	
	---	10701180	0.74	-15	0.0	grey	
	---	10700096	1.40	12	0.0	grey	
	---	10701443	1.51	12	0.0	grey	
	---	10700788	1.42	16	0.0	grey	
	---	10701591	1.75	17	0.0	grey	
	---	10701399	1.57	33	0.0	grey	
	---	10701472	1.83	56	0.0	grey	
	---	10701359	2.43	59	0.0	grey	
	---	10701461	1.92	62	0.0	grey	
	---	10865696	1.85	180	0.0	grey	
	---	10700457	1.21	316	0.0	grey	

	---	10701136	0.63	-21	0.7	l-cn	
	---	10921268	1.97	154	12.2	turq	
	---	10714204	1.73	367	11.3	turq	
	---	10700124	1.89	511	8.3	turq	
	---	10701207	2.58	286	8.0	turq	
	---	10908810	1.20	71	8.0	turq	
	---	10932319	2.05	89	6.4	turq	
	---	10700714	1.81	267	6.2	turq	
	---	10939699	1.91	45	5.6	turq	
	---	10701336	1.54	525	4.4	turq	
	---	10701392	1.54	16	3.8	turq	
	---	10700674	0.68	-10	3.7	turq	
	---	10700473	1.67	139	3.6	turq	
	---	10701404	1.43	1454	3.4	turq	
	---	10701260	2.24	195	2.9	turq	
	---	10700703	1.36	144	2.2	turq	
	---	10700573	1.69	87	1.3	turq	

B. Female Kidney

Gene Symbol	RefSeq/GenBank/ Ensemble Number	Affymetrix Probset ID	V/C	Mean Diff.	k.in	Mod	Gene Title
**APOPTOSIS**							
Bcl2l1	NM_031535	10850826	1.22	16	0.0	grey	Bcl2-like 1
Casp1	NM_012762	10907815	0.68	-69	2.2	mgnt	caspase 1
Fastk	NM_001011967	10852896	1.21	55	0.9	mgnt	Fas-activated serine/threonine kinase
**CYTOSKELETON-ECM**							
Clecsf6	NM_001005899	10858573	0.74	-20	1.6	mgnt	C-type (calcium dependent, carbohydrate recognition domain) lectin, superfamily member 6
Col14a1	NM_001130548	10896558	0.69	-50	0.0	grey	collagen, type XIV, alpha 1
Esco1	BC166441	10803194	0.77	-40	0.0	grey	establishment of cohesion 1 homolog 1 (S. cerevisiae)
Kifc3	NM_001103352	10805944	1.21	12	1.9	mgnt	kinesin family member C3
LOC100361793	ENSRNOT00000067139	10832281	1.23	18	0.0	grey	keratin associated protein 12-1-like
Tpm2	NM_001024345	10876324	0.83	-21	0.0	grey	tropomyosin 2, beta
**DEVELOPMENT**							
Acd	NM_001037193	10810662	1.28	36	2.1	red	adrenocortical dysplasia homolog (mouse)
Fance	NM_001191718	10828723	1.28	13	0.0	grey	Fanconi anemia, complementation group E
Lmbr1l	NM_001013950	10907062	1.22	16	1.2	cyan	limb region 1-like homolog (mouse)
Lrrc10	NM_001128494	10895585	0.83	-13	0.0	grey	leucine-rich repeat-containing 10
Luc7l	NM_001024269	10732581	1.21	40	5.9	blue	LUC7-like (S. cerevisiae)
Nov	NM_030868	10896541	0.83	-18	0.0	grey	nephroblastoma overexpressed gene
Pard3b	NM_001191808	10923810	1.23	27	0.8	cyan	par-3 partitioning defective 3 homolog B (C. elegans)
Rapgef3	NM_021690	10906679	1.27	13	0.0	grey	Rap guanine nucleotide exchange factor (GEF) 3
Samd9l	ENSRNOT00000013044	10860806	0.75	-141	0.0	grey	sterile alpha motif domain containing 9-like
Sv2a	NM_057210	10817512	1.59	16	0.0	grey	synaptic vesicle glycoprotein 2a
**EPIGENETICS**							
Dot1l	NM_001108733	10900419	1.31	16	0.7	mgnt	DOT1-like, histone H3 methyltransferase (S. cerevisiae)
RGD1561065	NM_001108253	10933017	1.20	17	0.0	grey	similar to mKIAA1111 protein
Suv420h2	NM_001107475	10718873	1.38	25	0.0	grey	suppressor of variegation 4-20 homolog 2 (Drosophila)
**GROWTH FACTORS**							
Angptl1	NM_001109383	10764862	0.77	-21	1.9	mgnt	angiopoietin-like 1
**IMMUNE RESPONSE**							
C1qb	NM_019262	10880727	0.77	-38	0.0	grey	complement component 1, q subcomponent, B chain
Cd36	NM_031561	10853240	0.61	-223	0.0	grey	CD36 molecule (thrombospondin receptor)
Cd48	NM_139103	10765634	0.80	-307	2.6	mgnt	Cd48 molecule
Ifi44	NM_001107729	10827349	0.83	-43	0.0	grey	interferon-induced protein 44
**Ifi47**	**NM_172019**	**10733056**	**0.81**	**-163**	**2.7**	**mgnt**	**interferon gamma inducible protein 47**
LOC287167	NM_001013853	10741765	1.25	302	1.2	m-blu	globin, alpha
RT1-Bb	NM_001004084	10831567	0.69	-76	0.0	grey	RT1 class II, locus Bb
Slamf9	NM_001105971	10765746	0.72	-12	0.0	grey	SLAM family member 9
**METABOLISM & TRANSPORT**							
Alox15	NM_031010	10744425	1.22	11	0.6	m-blu	arachidonate 15-lipoxygenase
Aqp2	NM_012909	10899145	1.28	11	0.0	grey	aquaporin 2 (collecting duct)
Atp1a2	NM_012505	10769998	0.79	-16	0.0	grey	ATPase, Na+/K+ transporting, alpha 2 polypeptide
Clcnkb	NM_173103	10881084	1.38	16	0.0	grey	chloride channel Kb
Colec12	NM_001025721	10800088	0.79	-47	1.2	mgnt	collectin sub-family member 12
Endog	NM_001034938	10844136	1.31	24	0.0	grey	endonuclease G
Gypc	NM_001013233	10803666	0.80	-54	0.0	grey	glycophorin C (Gerbich blood group)
Hexdc	NM_001142562	10740221	1.55	29	0.7	cyan	hexosaminidase (glycosyl hydrolase family 20, catalytic domain) containing

**Idua**	**NM_001172084**	**10775001**	**1.28**	**68**	**2.7**	**cyan**	**alpha-L-iduronidase**
Kcnj10	NM_031602	10765740	1.26	12	0.9	pink	potassium inwardly-rectifying channel, subfamily J, member 10
Lcp1	NM_001012044	10781496	0.81	-59	2.4	mgnt	lymphocyte cytosolic protein 1
LOC687381	ENSRNOT00000034261	10743622	1.30	18	0.0	grey	similar to COX10 homolog, cytochrome c oxidase assembly protein, heme A: farnesyltransferase
Naaa	NM_001010967	10771690	0.73	-28	0.0	grey	N-acylethanolamine acid amidase
Nat5	NM_001108595	10840460	0.83	-38	0.0	grey	N-acetyltransferase 5
Sh3bgrl	NM_001173339	10934669	0.83	-142	2.7	mgnt	SH3 domain binding glutamic acid-rich protein like
Slc12a3	NM_019345	10809337	1.49	20	0.0	grey	solute carrier family 12 (sodium/chloride transporters), member 3
Slc7a4	NM_001107078	10752433	1.36	17	1.9	mgnt	solute carrier family 7 (cationic amino acid transporter, y+ system), member 4
Trak1	NM_001134565	10914385	1.26	33	0.0	grey	trafficking protein, kinesin binding 1
**RECEPTORS & BINDING PROTEINS**							
Agtr1a	NM_030985	10798194	0.82	-34	1.4	mgnt	angiotensin II receptor, type 1a
Leng8	NM_001037790	10719006	1.36	56	8.2	blue	leukocyte receptor cluster (LRC) member 8
**Lpar6**	**NM_001045843**	**10781441**	**0.83**	**-76**	**3.0**	**mgnt**	**lysophosphatidic acid receptor 6**
RGD1562717	ENSRNOT00000049859	10753629	0.76	-22	1.8	mgnt	similar to ABI gene family, member 3 (NESH) binding protein
Rtp4	NM_001108321	10755088	0.77	-45	2.1	mgnt	receptor (chemosensory) transporter protein 4
**SIGNALING**							
Arhgap8	NM_001004242	10898268	1.21	16	0.0	grey	Rho GTPase activating protein 8
LOC686547	ENSRNOT00000012614	10785618	0.81	-48	0.0	grey	similar to TBC1 domain family member 4 (Akt substrate of 160 kDa) (AS160)
P2ry1	NM_012800	10815652	1.51	44	0.0	grey	purinergic receptor P2Y, G-protein coupled, 1
Plk2	NM_031821	10812954	0.82	-54	0.0	grey	polo-like kinase 2 (Drosophila)
Reps1	NM_001106264	10701880	1.21	22	1.0	red	RALBP1 associated Eps domain containing 1
Rgs18	NM_001047084	10768357	0.83	-46	2.3	mgnt	regulator of G-protein signaling 18
Tbc1d17	NM_001106258	10721486	1.24	47	2.6	salmor	TBC1 domain family, member 17
**TRANSCRIPTION**							
Banp	NM_001106191	10808461	1.26	17	5.1	ylw	Btg3 associated nuclear protein
Cnot7	NM_001107313	10787209	0.81	-71	0.0	grey	CCR4-NOT transcription complex, subunit 7
Gata3	NM_133293	10796220	1.47	11	0.0	grey	GATA binding protein 3
Gmeb1	NM_001109268	10880240	1.22	21	0.7	mgnt	glucocorticoid modulatory element binding protein 1
Ino80d	NM_001191809	10928475	1.22	27	0.6	cyan	INO80 complex subunit D
Klhl3	ENSRNOT00000034877	10793893	1.42	11	0.5	cyan	kelch-like 3 (Drosophila)
LOC100125368	NM_001139487	10900136	0.81	-34	1.6	red	zinc finger protein LOC100125368
LOC100363361	ENSRNOT00000028553	10824904	1.27	15	1.2	red	zinc finger protein 687-like
Nfrkb	NM_001108133	10908833	1.22	23	1.5	cyan	nuclear factor related to kappa B binding protein
Nol6	EF688596	10876076	1.23	22	0.0	grey	nucleolar protein family 6 (RNA-associated)
RGD1564400	ENSRNOT00000033258	10836556	0.78	-79	2.3	black	similar to Eukaryotic translation initiation factor 5 (eIF-5)
Runx1	NM_017325	10753222	0.83	-13	0.0	grey	runt-related transcription factor 1
**Tceb1**	**NM_022593**	**10874923**	**0.82**	**-215**	**2.9**	**mgnt**	**transcription elongation factor B (SIII), polypeptide 1**
Tsc22d2	NM_001191960	10815534	1.27	49	1.9	cyan	TSC22 domain family, member 2
**TRANSLATION & PROTEIN MODIFICATION**							
Mif4gd	NM_001014122	10748991	1.22	21	1.2	mgnt	MIF4G domain containing
Parp14	NM_001191659	10751469	0.82	-74	0.0	grey	poly (ADP-ribose) polymerase family, member 14
RGD1561453	ENSRNOT00000049732	10714411	0.78	-18	2.8	gr-yel	similar to ribosomal protein S12
Rps27a	NM_031113	10879392	0.75	-531	1.8	gr-yel	ribosomal protein S27a
Snrnp70	NM_001108483	10721802	1.20	97	4.6	blue	small nuclear ribonucleoprotein 70 (U1)
Srrm2	ENSRNOT00000006370	10731980	1.28	155	7.3	blue	serine/arginine repetitive matrix 2
**MISCELLANEOUS & UNKNOWN**							
LOC100125385	NM_001103364	10897239	1.26	26	1.9	red	hypothetical protein LOC100125385
RGD1306926	ENSRNOT00000054974	10749620	1.27	11	1.0	cyan	similar to hypothetical protein FLJ22175
**RGD1308616**	**ENSRNOT00000039235**	**10879242**	**1.49**	**36**	**2.4**	**cyan**	**similar to KIAA0467 protein**
RGD1359529	NM_001014193	10872829	1.27	168	7.0	blue	similar to chromosome 1 open reading frame 63
Tc2n	NM_001025152	10891765	0.74	-65	0.0	grey	tandem C2 domains, nuclear
Tmem229b	NM_001109359	10890780	0.79	-12	0.0	grey	transmembrane protein 229B
Tmem38a	NM_001100175	10790651	1.23	19	1.1	cyan	transmembrane protein 38a
Tmem81	ENSRNOT00000003481	10763931	0.81	-71	1.5	mgnt	transmembrane protein 81
**EST's**							
RGD1309220	BC105865	10927332	1.24	30	1.9	cyan	similar to RIKEN cDNA 4632411B12
RGD1310827	NM_001034010	10862605	0.70	-23	0.0	grey	similar to RIKEN cDNA 1200009O22; EST AI316813
	ENSRNOT00000001195	10911925	0.83	-11	2.1	green	
	ENSRNOT00000001469	10760483	1.22	15	0.8	cyan	
	ENSRNOT00000001814	10761917	1.23	17	0.0	grey	
	ENSRNOT00000002539	10755798	1.31	25	0.0	grey	
	ENSRNOT00000027802	10780527	1.27	30	1.4	cyan	
	ENSRNOT00000030293	10718408	0.82	-24	2.4	turq	
	ENSRNOT00000038275	10902016	0.80	-12	0.0	grey	
	ENSRNOT00000052689	10896964	0.82	-29	2.5	gr-yel	
	ENSRNOT00000052749	10779724	0.68	-18	3.3	gr-yel	
	ENSRNOT00000052790	10831150	0.79	-14	2.9	gr-yel	

	ENSRNOT00000053071	10806012	0.70	-11	0.9	gr-yel	
	ENSRNOT00000053086	10811999	0.78	-239	4.1	gr-yel	
	ENSRNOT00000053230	10758033	0.72	-86	4.4	gr-yel	
	ENSRNOT00000053328	10855946	0.62	-162	0.0	grey	
	ENSRNOT00000053394	10900318	2.07	33	0.0	grey	
	**ENSRNOT00000053552**	**10713608**	**0.70**	**-209**	**5.4**	**gr-yel**	
	ENSRNOT00000053557	10916961	0.75	-13	0.0	grey	
	ENSRNOT00000054087	10864356	1.39	23	0.0	grey	
	**ENSRNOT00000054102**	**10723896**	**0.73**	**-66**	**4.9**	**gr-yel**	
	ENSRNOT00000054649	10930226	0.72	-146	1.7	ylw	
	ENSRNOT00000061295	10803438	1.35	11	7.0	blue	
	FQ217306	10825274	0.77	-30	0.0	grey	
	---	10700809	1.28	15	1.4	purple	
	---	10701541	0.77	-225	0.0	grey	
	---	10700106	0.76	-51	0.0	grey	
	---	10700884	0.75	-28	0.0	grey	
	---	10701591	0.60	-15	0.0	grey	
	---	10700097	1.66	11	0.5	cyan	
	---	10700775	1.31	13	0.0	grey	
	---	10701273	1.32	13	0.0	grey	
	---	10700968	1.36	13	0.5	black	
	---	10701062	1.37	14	0.0	grey	
	---	10701563	1.47	15	0.0	grey	
	---	10700535	1.24	18	4.4	blue	
	---	10701555	1.93	18	0.0	grey	
	---	10700957	1.53	19	0.0	grey	
	---	10701067	1.50	21	0.0	grey	
	---	10701532	1.26	22	0.0	grey	
	---	10701619	1.66	23	0.6	cyan	
	---	10700923	1.82	24	4.3	blue	
	---	10700608	1.24	34	0.0	grey	
	---	10700411	1.30	47	2.6	blue	
	---	10700365	1.28	79	2.0	mgnt	
	---	10701121	1.43	89	0.0	grey	
	---	10700854	1.32	95	0.0	grey	
	---	10701261	1.26	126	0.0	grey	
	---	10701605	1.37	325	9.0	blue	
	---	10820008	0.74	-293	1.0	ylw	
	---	10791602	0.76	-22	0.0	grey	
	---	10833416	0.76	-22	0.0	grey	
	---	10834668	1.39	11	0.0	grey	
	---	10862453	1.34	116	5.5	blue	
	---	10811898	1.30	224	0.5	ylw	

C. Female Liver

Gene Symbol	RefSeq/GenBank/ Ensemble Number	Affymetrix Probset ID	V/C	Mean Diff.	k.in	Mod	Gene Title
**CELL CYCLE**							
**Ccnt2**	**NM_001107171**	**10767280**	**0.77**	**-96**	**14.2**	**blue**	**cyclin T2**
**CYTOSKELETON-ECM**							
Ccbe1	ENSRNOT00000034069	10802338	1.33	16	0.0	grey	collagen and calcium binding EGF domains 1
Coil	NM_017360	10737403	0.77	-16	0.0	grey	coilin
Col4a2	XM_001076134	10792997	1.24	61	0.0	grey	collagen, type IV, alpha 2
Col6a1	ENSRNOT00000001679	10829437	1.24	84	0.0	grey	collagen, type VI, alpha 1
Mfap3l	NM_001012049	10791474	0.80	-62	0.0	grey	microfibrillar-associated protein 3-like
Pfn2	NM_030873	10823303	0.81	-65	0.0	grey	profilin 2
**DEVELOPMENT**							
LOC683626	NM_001129880	10888608	1.57	129	0.0	grey	similar to limb-bud and heart
Robo1	NM_022188	10749892	1.22	10	0.0	grey	roundabout homolog 1 (Drosophila)
Serpinh1	NM_017173	10723884	1.25	209	0.0	grey	serine (or cysteine) peptidase inhibitor, clade H, member 1
Skil	ENSRNOT00000013191	10822637	0.73	-69	3.7	brwn	SKI-like oncogene
Wdr37	NM_001107362	10795969	0.82	-39	0.0	grey	WD repeat domain 37
**EPIGENETICS**							
Pcgf6	NM_001013154	10730624	0.77	-19	0.0	grey	polycomb group ring finger 6
**IMMUNE RESPONSE**							
Klra17	NM_001172088	10866098	0.67	-47	0.0	grey	killer cell lectin-like receptor, subfamily A, member 17
Orm1	NM_053288	10869476	0.79	-21	0.0	grey	orosomucoid 1

**METABOLISM & TRANSPORT**							
Alas2	NM_013197	10932912	1.54	60	3.0	m-blu	aminolevulinate, delta-, synthase 2
Aof1	NM_001107343	10797741	0.83	-17	0.0	grey	amine oxidase (flavin containing) domain 1
Apoa2	NM_013112	10765530	1.22	10	0.0	grey	apolipoprotein A-II
Clcn4-2	NM_022198	10933259	0.81	-16	0.0	grey	chloride channel 4-2
Ctps2	NM_001034998	10937777	0.68	-159	0.6	mgnt	CTP synthase II
Hao1	NM_001107780	10850190	0.78	-10	0.0	grey	hydroxyacid oxidase (glycolate oxidase) 1
Inmt	NM_001109022	10862694	1.24	15	0.0	grey	indolethylamine N-methyltransferase
Kynu	NM_053902	10836019	0.80	-18	0.0	grey	kynureninase (L-kynurenine hydrolase)
Mblac2	NM_001108934	10812343	0.78	-28	0.0	grey	metallo-beta-lactamase domain containing 2
Pigl	NM_138901	10734306	0.69	-28	0.0	grey	phosphatidylinositol glycan anchor biosynthesis, class L
Pop4	NM_001009642	10721191	0.81	-38	0.0	grey	processing of precursor 4, ribonuclease P/MRP subunit (S. cerevisiae)
Sh3bgrl3	NM_001106688	10880436	1.26	58	0.0	grey	SH3 domain binding glutamic acid-rich protein-like 3
Slc25a45	ENSRNOT00000051979	10713152	0.76	-31	0.0	grey	solute carrier family 25, member 45
Slc28a2	NM_031664	10849275	0.57	-173	0.0	grey	solute carrier family 28 (sodium-coupled nucleoside transporter), member 2
Slc6a6	NM_017206	10857314	0.70	-217	0.0	grey	solute carrier family 6 (neurotransmitter transporter, taurine), member 6
Sod1	NM_017050	10753005	1.23	345	0.0	grey	superoxide dismutase 1, soluble
Trub1	NM_001012173	10716320	0.83	-18	0.0	grey	TruB pseudouridine (psi) synthase homolog 1 (E. coli)
**PROTEOLYSIS**							
Dcun1d4	NM_001108359	10776536	0.83	-58	0.0	grey	DCN1, defective in cullin neddylation 1, domain containing 4 (S. cerevisiae)
Pgpep1	NM_201988	10787507	0.83	-66	0.5	l-cn	pyroglutamyl-peptidase I
**RECEPTORS & BINDING PROTEINS**							
Cxadr	NM_053570	10749983	0.82	-56	1.4	l-grn	coxsackie virus and adenovirus receptor
Fabp2	NM_013068	10818846	0.52	-12	0.0	grey	fatty acid binding protein 2, intestinal
**SIGNALING**							
Drg2	ENSRNOT00000005101	10734069	0.83	-71	0.0	grey	developmentally regulated GTP binding protein 2
Pex3	NM_031350	10716910	0.77	-81	0.0	grey	peroxisomal biogenesis factor 3
Rgs16	NM_001077589	10764773	1.40	18	0.0	grey	regulator of G-protein signaling 16
Rragd	NM_001106641	10868125	0.76	-36	0.0	grey	Ras-related GTP binding D
**TRANSCRIPTION**							
Gtf2a2	NM_053345	10911309	0.71	-52	3.6	turq	general transcription factor IIA, 2
Gtf3c3	NM_001108239	10928034	0.72	-80	4.3	turq	general transcription factor IIIC, polypeptide 3
LOC100364077	ENSRNOT00000006672	10924669	0.77	-14	3.9	brwn	UTP14, U3 small nucleolar ribonucleoprotein, homolog B-like
Morc2	NM_001106016	10773725	1.20	21	0.0	grey	MORC family CW-type zinc finger 2
Rai14	NM_001011947	10821924	0.80	-23	0.0	grey	retinoic acid induced 14
Zfp212	ENSRNOT00000009054	10855203	0.83	-15	2.4	blue	Zinc finger protein 212
Zfp3	NM_001109034	10735375	0.73	-25	0.0	grey	zinc finger protein 3
**TRANSLATION & PROTEIN MODIFICATION**							
Rps14	NM_022672	10871997	1.26	309	2.8	gr-yel	ribosomal protein S14
Rpsa	NM_017138	10772950	1.21	113	0.7	l-cn	ribosomal protein SA
**MISCELLANEOUS & UNKNOWN**							
Fam175b	NM_001106307	10711723	0.78	-97	0.0	grey	family with sequence similarity 175, member B
LOC686092	ENSRNOT00000050090	10923334	1.22	109	0.8	gr-yel	similar to UPF0197 protein C11orf10 homolog
Ttc21b	NM_001191737	10845829	0.74	-42	0.0	grey	tetratricopeptide repeat domain 21B
**EST's**							
LOC100364132	XM_002727520	10931172	0.81	-11	0.0	grey	rCG42947-like
RGD1304792	NM_001134554	10856502	0.79	-12	0.0	grey	similar to chromosome 2 open reading frame 3; transcription factor 9 (binds GC-rich sequences)
RGD1305014	BC083846	10726244	0.71	-23	1.3	brwn	similar to RIKEN cDNA 2310057M21
	ENSRNOT00000060617	10844248	1.32	30	0.0	grey	
	ENSRNOT00000058436	10788858	0.71	-201	0.0	grey	
	ENSRNOT00000056863	10838153	1.34	22	0.0	grey	
	ENSRNOT00000054530	10805787	1.56	553	0.0	grey	
	ENSRNOT00000054339	10851378	1.37	14	2.5	gr-yel	
	ENSRNOT00000053902	10827450	1.44	988	0.0	grey	
	ENSRNOT00000053444	10824409	0.73	-16	0.0	grey	
	ENSRNOT00000053325	10711125	1.59	68	1.9	gr-yel	
	ENSRNOT00000053318	10727082	1.25	26	1.8	gr-yel	
	ENSRNOT00000052628	10834602	1.90	357	0.0	grey	
	ENSRNOT00000045655	10794749	1.30	109	0.0	grey	
	ENSRNOT00000044978	10894302	0.83	-14	0.0	grey	
	ENSRNOT00000041882	10791650	1.37	338	0.0	grey	
	ENSRNOT00000037015	10846604	0.69	-61	0.0	grey	
	ENSRNOT00000030103	10716570	0.82	-10	0.0	grey	
	ENSRNOT00000024645	10706363	0.79	-30	0.0	grey	
	ENSRNOT00000011530	10891878	0.72	-29	0.0	grey	
	---	10700280	0.47	-41	0.0	grey	
	---	10701202	1.67	15	0.0	grey	
	---	10701117	1.70	33	0.0	grey	

	---	10700358	0.73	-119	0.0	grey	
	---	10700911	0.54	-38	0.0	grey	
	---	10701026	0.67	-36	0.0	grey	
	---	10700351	0.65	-33	0.0	grey	
	---	10700508	0.61	-29	6.4	blue	
	---	10700964	0.71	-29	0.0	grey	
	---	10700072	0.57	-28	0.0	grey	
	---	10700282	0.56	-26	3.9	blue	
	---	10700402	0.71	-24	0.0	grey	
	---	10701484	0.74	-18	0.0	grey	
	---	10701191	0.66	-15	2.0	blue	
	---	10701192	0.79	-14	0.0	grey	
	---	10700826	0.77	-13	7.1	blue	
	---	10700483	0.72	-11	0.0	grey	
	---	10700117	1.25	10	0.7	black	
	---	10700768	1.40	12	0.9	m-blu	
	---	10700999	1.59	18	0.0	grey	
	---	10700958	1.90	20	0.0	grey	
	---	10701309	1.46	418	0.0	grey	
	---	10861211	0.64	-14	0.0	grey	
	---	10854030	2.04	14	0.0	grey	
	---	10874328	1.33	34	0.0	grey	
	---	10868796	1.45	38	0.0	grey	

D. Ovary

Gene Symbol	RefSeq/GenBank/ Ensemble Number	Affymetrix Probset ID	V/C	Mean Diff.	k.in	Mod	Gene Title
**APOPTOSIS**							
Traf3ip2	NM_001044248	10830398	0.74	-19	3.5	brwn	Traf3 interacting protein 2
**CELL CYCLE**							
**Cep350**	**ENSRNOT00000005235**	**10768874**	**1.34**	**47**	**8.5**	**brwn**	**centrosomal protein 350kDa**
**CYTOSKELETON-ECM**							
Cgnl1	NM_001108164	10918600	1.31	85	0.0	grey	cingulin-like 1
Csrp2	NM_177425	10833152	1.37	25	0.0	grey	cysteine and glycine-rich protein 2
Esco1	BC166441	10803194	1.30	41	0.0	grey	establishment of cohesion 1 homolog 1 (S. cerevisiae)
Odf1	NM_024126	10896227	2.23	24	13.6	ylw	outer dense fiber of sperm tails 1
Odf2	NM_017213	10835005	1.29	26	7.7	ylw	outer dense fiber of sperm tails 2
RGD1563284	ENSRNOT00000059800	10787814	1.34	12	0.0	grey	similar to actin
Smc1a	NM_031683	10933138	1.21	94	1.6	green	structural maintenance of chromosomes 1A
Ttll12	NM_001135922	10905827	1.24	14	0.0	grey	tubulin tyrosine ligase-like family, member 12
**DEVELOPMENT**							
Auts2	ENSRNOT00000044800	10761299	0.76	-10	1.1	pink	autism susceptibility candidate 2
**Crisp2**	**NM_001011710**	**10922027**	**3.67**	**29**	**17.0**	**ylw**	**cysteine-rich secretory protein 2**
Emg1	NM_001107888	10865452	1.27	85	1.0	l-yel	EMG1 nucleolar protein homolog (S. cerevisiae)
Far1	NM_001011933	10710051	1.24	92	0.0	grey	fatty acyl CoA reductase 1
Llgl2	NM_001127549	10739642	0.81	-20	0.0	grey	lethal giant larvae homolog 2 (Drosophila)
Lrrc26	NM_001014053	10834187	0.67	-10	0.0	grey	leucine rich repeat containing 26
Lrrc8b	NM_001107204	10775278	1.25	23	0.0	grey	leucine rich repeat containing 8 family, member B
Mkks	NM_001008353	10850221	1.27	35	4.6	brwn	McKusick-Kaufman syndrome
**Smcp**	**NM_031536**	**10824742**	**3.33**	**29**	**14.4**	**ylw**	**sperm mitochondria-associated cysteine-rich protein**
Syt11	NM_031667	10824349	1.39	17	0.0	grey	synaptotagmin XI
Sytl2	BC168993	10708591	0.79	-27	0.0	grey	synaptotagmin-like 2
Tmub1	NM_001080153	10852892	1.27	21	3.2	black	transmembrane and ubiquitin-like domain containing 1
Urb2	NM_001135708	10808764	1.32	18	2.5	ylw	URB2 ribosome biogenesis 2 homolog (S. cerevisiae)
**EPIGENETICS**							
Asmtl	NM_001105915	10757162	1.22	19	0.7	coral	acetylserotonin O-methyltransferase-like
Dnmt1	NM_053354	10915437	1.22	25	0.0	grey	DNA (cytosine-5-)-methyltransferase 1
Icmt	NM_133310	10874319	1.23	22	1.1	black	isoprenylcysteine carboxyl methyltransferase
Prm1	NM_001002850	10731498	1.33	13	9.1	ylw	protamine 1
Rg9mtd1	NM_001008337	10750795	1.45	41	3.3	brwn	RNA (guanine-9-) methyltransferase domain containing 1
Tnp1	NM_017056	10928844	4.07	35	13.2	ylw	transition protein 1
**GOLGI APPARATUS**							
Ap1s1	NM_001108331	10761042	1.21	36	0.6	coral	adaptor-related protein complex 1, sigma 1 subunit
Gbf1	NM_001191634	10715746	1.20	69	0.0	grey	golgi-specific brefeldin A resistant guanine nucleotide exchange factor 1
**GROWTH FACTORS**							
Bmp6	NM_013107	10797949	1.33	19	0.0	grey	bone morphogenetic protein 6
Cmtm2a	NM_001013142	10805724	1.73	12	13.5	ylw	CKLF-like MARVEL transmembrane domain containing 2A

**Jag1**	**NM_019147**	**10850228**	**1.66**	**65**	**4.3**	**green**	**jagged 1**
Npy	NM_012614	10855506	1.53	41	0.0	grey	neuropeptide Y
**IMMUNE RESPONSE**							
Cd226	NM_001107370	10803110	1.48	13	0.0	grey	CD226 molecule
Ifi44l	XM_227820	10834014	1.25	40	0.0	grey	interferon-induced protein 44-like
Klrb1c	NM_001085403	10859095	1.31	11	0.0	grey	killer cell lectin-like receptor subfamily B member 1C
Klri1	NM_001012649	10866067	1.34	20	0.0	grey	killer cell lectin-like receptor family I member 1
Lage3	NM_001106345	10936072	1.31	58	1.0	brwn	L antigen family, member 3
**METABOLISM & TRANSPORT**							
Abhd2	NM_001106275	10708053	1.36	108	0.9	tan	abhydrolase domain containing 2
Alas2	NM_013197	10932912	1.55	61	3.0	m-blu	aminolevulinate, delta-, synthase 2
Aldob	NM_012496	10876719	1.46	75	0.0	grey	aldolase B, fructose-bisphosphate
B4galt5	NM_001108608	10851952	1.34	31	0.0	grey	UDP-Gal:betaGlcNAc beta 1,4-galactosyltransferase, polypeptide 5
Car14	NM_001109655	10825100	0.74	-49	2.8	brwn	carbonic anhydrase 14
Ces6	NM_144743	10808984	0.82	-12	0.9	brwn	carboxylesterase 6
Elovl6	NM_134383	10819005	1.67	51	3.1	green	ELOVL family member 6, elongation of long chain fatty acids (yeast)
Exosc3	NM_001107936	10876520	1.24	29	0.0	grey	exosome component 3
Hba-a2	NM_013096	10741756	1.24	1647	4.5	m-blu	hemoglobin alpha, adult chain 2
**Hbb**	**NM_033234**	**10724315**	**1.69**	**1134**	**4.7**	**m-blu**	**hemoglobin, beta**
Hbb-b1	NM_198776	10724319	1.46	759	4.5	m-blu	hemoglobin, beta adult major chain
Lin7c	NM_021851	10838346	1.20	127	4.6	brwn	lin-7 homolog C (C. elegans)
LOC678704	NM_001109369	10751769	1.23	199	7.7	brwn	hypothetical protein LOC678704
**LOC689064**	**NM_001111269**	**10724311**	**1.44**	**850**	**4.9**	**m-blu**	**beta-globin**
Mepce	NM_001100678	10761020	1.30	24	3.4	black	methylphosphate capping enzyme
Ndufb4	NM_001037338	10839229	0.83	-135	2.5	black	NADH dehydrogenase (ubiquinone) 1 beta subcomplex 4
**Oaz3**	**NM_001101018**	**10824814**	**2.09**	**18**	**15.4**	**ylw**	**ornithine decarboxylase antizyme 3**
Pigm	NM_024144	10765744	1.26	69	0.0	grey	phosphatidylinositol glycan anchor biosynthesis, class M
Ptges2	NM_001107832	10835597	1.32	41	0.0	grey	prostaglandin E synthase 2
Pts	NM_017220	10917209	1.33	22	0.0	grey	6-pyruvoyl-tetrahydropterin synthase
Pus3	NM_001108134	10908990	1.27	20	6.6	brwn	pseudouridylate synthase 3
Slc30a1	NM_022853	10766774	1.33	68	0.4	tan	solute carrier family 30 (zinc transporter), member 1
Slc7a5	NM_017353	10811531	1.37	29	0.0	grey	solute carrier family 7 (cationic amino acid transporter, y+ system), member 5
Uap1	NM_001191930	10769715	1.32	41	1.1	pink	UDP-N-acteylglucosamine pyrophosphorylase 1
Umps	NM_001025402	10751586	1.21	50	0.0	grey	uridine monophosphate synthetase
**PROTEOLYSIS**							
LOC100360708	ENSRNOT00000049610	10873723	1.54	13	0.0	grey	ubiquitin A-52 residue ribosomal protein fusion product 1
**RECEPTORS & BINDING PROTEINS**							
Abcc9	NM_013040	10866724	1.25	42	0.0	grey	ATP-binding cassette, sub-family C (CFTR/MRP), member 9
Abcg2	NM_181381	10855862	1.20	17	2.9	brwn	ATP-binding cassette, sub-family G (WHITE), member 2
Leprotl1	NM_001013188	10788553	1.21	48	0.0	grey	leptin receptor overlapping transcript-like 1
Nab2	NM_001134874	10903166	1.22	12	0.0	grey	Ngfi-A binding protein 2
Rybp	NM_001107879	10864441	1.21	20	1.7	ylw	RING1 and YY1 binding protein
**Trip11**	**NM_001191772**	**10891795**	**1.20**	**46**	**9.0**	**brwn**	**thyroid hormone receptor interactor 11**
**SIGNALING**							
Camsap1l1	NM_001134503	10768093	1.20	49	0.6	tan	calmodulin regulated spectrin-associated protein 1-like 1
Eltd1	NM_022294	10819770	1.35	90	0.0	grey	EGF, latrophilin and seven transmembrane domain containing 1
Enpp6	NM_001107311	10791631	1.67	21	0.0	grey	ectonucleotide pyrophosphatase/phosphodiesterase 6
Ensa	NM_001033974	10834035	1.21	29	0.0	grey	endosulfine alpha
Ern1	NM_001191926	10748314	1.30	27	0.0	grey	endoplasmic reticulum to nucleus signaling 1
Gng11	NM_022396	10853554	1.29	19	0.0	grey	guanine nucleotide binding protein (G protein), gamma 11
Gnl3l	NM_001081958	10937540	1.21	74	1.1	l-yel	guanine nucleotide binding protein-like 3 (nucleolar)-like
Hpcal1	NM_017356	10883801	1.23	31	0.0	grey	hippocalcin-like 1
Ikbkg	NM_199103	10936134	1.22	31	0.0	grey	inhibitor of kappaB kinase gamma
Ippk	NM_001008556	10797681	1.28	15	1.4	pink	inositol 1,3,4,5,6-pentakisphosphate 2-kinase
Lpin1	NM_001012111	10889269	1.23	37	0.0	grey	lipin 1
Ppp1r10	NM_022951	10830989	1.20	26	0.0	grey	protein phosphatase 1, regulatory subunit 10
Ppp1r9b	NM_053474	10737587	1.25	37	0.0	grey	protein phosphatase 1, regulatory subunit 9B
tGap1	NM_001007635	10814217	1.59	10	7.6	ylw	GTPase activating protein testicular GAP1
Traf3ip3	NM_001014132	10766806	1.21	11	0.0	grey	TRAF3 interacting protein 3
Trib1	NM_023985	10896793	1.28	28	0.6	l-cn	tribbles homolog 1 (Drosophila)
**TRANSCRIPTION**							
Ankrd49	NM_001126283	10915000	1.28	44	5.4	brwn	ankyrin repeat domain 49
Asb2	NM_001011984	10891888	0.78	-16	0.0	grey	ankyrin repeat and SOCS box-containing 2
Bmyc	NM_001013163	10834360	1.22	26	0.0	grey	brain expressed myelocytomatosis oncogene
Btg1	NM_017258	10895069	1.26	194	0.0	grey	B-cell translocation gene 1, anti-proliferative
Bwk1	NM_198743	10842879	1.23	86	2.6	pink	Bwk1 leukemia-related gene
Ccdc6	ENSRNOT00000032159	10832705	1.22	20	0.0	grey	coiled-coil domain containing 6
Ccdc7	NM_001011561	10812061	1.65	11	8.1	ylw	coiled-coil domain containing 7
Ddx18	NM_001006996	10767189	1.30	68	4.4	brwn	DEAD (Asp-Glu-Ala-Asp) box polypeptide 18

**Ddx21**	**NM_001037201**	**10937499**	**1.25**	**83**	**2.4**	**l-yel**	**DEAD (Asp-Glu-Ala-Asp) box polypeptide 21 (chrX)**
Ddx21	NM_001037201	10833198	1.25	84	1.6	l-yel	DEAD (Asp-Glu-Ala-Asp) box polypeptide 21 (chr20)
E2f5	ENSRNOT00000014361	10822258	1.23	15	0.9	pink	E2F transcription factor 5
Elf4	NM_001191735	10939584	1.29	15	0.0	grey	E74-like factor 4 (ets domain transcription factor)
Fbxl17	NM_001108235	10930210	1.27	13	2.3	black	F-box and leucine-rich repeat protein 17
Foxp1	NM_001034131	10864404	0.81	-39	0.0	grey	forkhead box P1
Foxp2	ENSRNOT00000034078	10853783	0.63	-12	3.9	blue	forkhead box P2
Hivep2	NM_024137	10701830	1.28	18	0.0	grey	human immunodeficiency virus type I enhancer binding protein 2
LOC499900	BC079070	10840379	1.29	19	0.0	grey	similar to Zinc finger protein 133
Med30	NM_001130539	10896515	1.20	46	0.0	grey	mediator complex subunit 30
Mtf1	NM_001108677	10871857	1.24	21	1.7	ylw	metal-regulatory transcription factor 1
Mtvr2	NM_001015013	10727999	1.21	38	1.6	black	mammary tumor virus receptor 2
Nip7	NM_138847	10807625	1.25	26	0.0	grey	nuclear import 7 homolog (S. cerevisiae)
**Noc3l**	**NM_001108523**	**10729999**	**1.24**	**30**	**8.6**	**brwn**	**nucleolar complex associated 3 homolog (S. cerevisiae)**
Nol10	NM_001014076	10883763	1.25	30	7.7	brwn	nucleolar protein 10
Ppara	NM_013196	10898359	1.29	35	3.0	black	peroxisome proliferator activated receptor alpha
RGD1562997	ENSRNOT00000037955	10800323	1.35	23	2.2	brwn	similar to Transcription initiation factor TFIID 105 kDa subunit (TAFII-105)
Set	NM_001012504	10749869	0.74	-88	3.4	black	SET nuclear oncogene
Tada2b	NM_001170455	10773358	1.25	23	2.9	black	transcriptional adaptor 2 (ADA2 homolog, yeast)-beta
Vhl	NM_052801	10857887	1.24	35	0.0	grey	von Hippel-Lindau tumor suppressor
Zdhhc13	NM_001039037	10707156	1.20	27	0.0	grey	zinc finger, DHHC-type containing 13
Zdhhc21	NM_001039009	10877772	1.23	34	3.7	brwn	zinc finger, DHHC-type containing 21
Zfp35	NM_001013141	10800493	1.29	30	5.9	turq	zinc finger protein 35
Zufsp	NM_001008308	10833265	1.30	22	0.0	grey	zinc finger with UFM1-specific peptidase domain
**TRANSLATION & PROTEIN MODIFICATION**							
Arf2	NM_024150	10738716	1.24	39	0.0	grey	ADP-ribosylation factor 2
Bms1	NM_001100751	10864942	1.24	34	0.0	grey	BMS1 homolog, ribosome assembly protein (yeast)
Clp1	NM_001009599	10846846	1.25	28	1.1	ylw	CLP1, cleavage and polyadenylation factor I subunit, homolog (S. cerevisiae)
Cpsf2	NM_001106753	10886414	1.23	43	0.0	grey	cleavage and polyadenylation specific factor 2
Drap1	NM_001077668	10727846	1.20	53	0.0	grey	Dr1 associated protein 1 (negative cofactor 2 alpha)
Eef1a2	NM_012660	10852464	0.79	-17	2.6	ylw	eukaryotic translation elongation factor 1 alpha 2
Gatc	NM_001108339	10758982	1.21	29	0.0	grey	glutamyl-tRNA(Gln) amidotransferase, subunit C homolog (bacterial)
**Lars**	**NM_001009637**	**10804187**	**1.25**	**85**	**8.7**	**brwn**	**leucyl-tRNA synthetase**
LOC690096	ENSRNOT00000012255	10774432	0.78	-25	1.9	ylw	similar to ribosomal protein L28
Mrpl49	NM_001047883	10728115	1.26	33	0.0	grey	mitochondrial ribosomal protein L49
Phax	NM_173133	10801810	1.20	25	0.0	grey	phosphorylated adaptor for RNA export
Prpf31	NM_001106219	10718539	1.21	27	1.3	green	PRP31 pre-mRNA processing factor 31 homolog (S. cerevisiae)
Qtrtd1	ENSRNOT00000000317	10751196	1.24	11	1.2	l-yel	queuine tRNA-ribosyltransferase domain containing 1
Rbm42	NM_001014159	10720734	1.23	17	2.5	black	RNA binding motif protein 42
RGD1559955	ENSRNOT00000046179	10925757	1.33	10	1.7	ylw	similar to 40S ribosomal protein S17
Rnaseh2b	NM_001007007	10780937	1.20	35	0.0	grey	ribonuclease H2, subunit B
Rpl7l1	ENSRNOT00000021803	10921527	1.25	108	0.0	grey	ribosomal protein L7-like 1
Rrp7a	NM_001130568	10905785	1.20	36	0.5	coral	ribosomal RNA processing 7 homolog A (S. cerevisiae)
Rrp8	NM_001008346	10724516	1.25	11	3.4	brwn	ribosomal RNA processing 8, methyltransferase, homolog (yeast)
Snrnp25	ENSRNOT00000067079	10741793	1.22	26	0.0	grey	small nuclear ribonucleoprotein 25 (U11/U12)
Taf9	NM_184048	10832879	1.36	125	0.0	grey	TAF9 RNA polymerase II, TATA box binding protein (TBP)-associated factor
**MISCELLANEOUS & UNKNOWN**							
Bxdc2	NM_001029915	10821914	1.23	75	6.1	brwn	brix domain containing 2
Ciao1	NM_001008766	10849635	1.32	78	4.1	ylw	cytosolic iron-sulfur protein assembly 1 homolog (S. cerevisiae)
Fam126a	NM_001191969	10852953	0.82	-62	0.0	grey	family with sequence similarity 126, member A
Fam96b	NM_001144854	10805640	1.22	46	0.6	l-yel	family with sequence similarity 96, member B
LOC100188933	NM_001134695	10833598	1.75	16	6.4	ylw	hypothetical protein LOC100188933
LOC314016	ENSRNOT00000006122	10884031	1.26	33	0.0	grey	similar to mKIAA0230 protein
LOC500034	NM_001109221	10861117	1.23	56	0.0	grey	similar to CG3570-PA
LOC500251	NM_001025047	10857107	1.27	20	0.0	grey	hypothetical protein LOC500251
Nacc1	NM_134413	10806677	1.27	30	0.0	grey	nucleus accumbens associated 1, BEN and BTB (POZ) domain containing
RGD1304810	NM_001107314	10791983	1.36	15	0.0	grey	similar to 6430573F11Rik protein
RGD1308430	BC158656	10780237	1.26	66	0.0	grey	similar to 1700123O20Rik protein
RGD1311899	BC088751	10762735	1.30	18	2.6	ylw	similar to RIKEN cDNA 2210016L21 gene
RGD1560175	ENSRNOT00000046208	10754077	1.23	34	2.0	green	similar to hypothetical protein KIAA2018
RGD1564257	NM_001163723	10882034	0.74	-13	0.0	grey	similar to hypothetical protein FLJ32825
Spetex-2A	NM_001009976	10782383	1.60	22	6.3	tan	Spetex-2A protein
**Spetex-2B**	**NM_001011697**	**10779265**	**1.50**	**20**	**6.9**	**tan**	**Spetex-2B protein**
Spetex-2C	NM_001011698	10779260	1.42	17	6.2	tan	Spetex-2C protein
Spetex-2D	NM_001011701	10782409	1.37	15	6.2	tan	Spetex-2D protein
Spetex-2F	NM_001009968	10782379	1.36	13	6.3	tan	Spetex-2F protein
**Spetex-2G**	**NM_001009971**	**10782418**	**1.41**	**19**	**6.6**	**tan**	**Spetex-2G protein**
Spg11	NM_001191571	10849172	1.20	35	0.0	grey	spastic paraplegia 11 (autosomal recessive)
Tmem57	NM_001025699	10880564	1.20	33	0.0	grey	transmembrane protein 57

Utp23	NM_001126266	10896499	1.23	12	0.4	tan	UTP23, small subunit (SSU) processome component, homolog (yeast)
Utp3	NM_001012036	10776064	1.27	29	0.9	pink	UTP3, small subunit (SSU) processome component, homolog (S. cerevisiae)
**EST's**							
LOC304131	NM_001013979	10750074	1.44	17	0.0	grey	similar to C21ORF7
MGC105649	NM_001008518	10839307	1.43	31	0.0	grey	hypothetical LOC302884
RGD1305158	BC167112	10875932	1.25	26	1.9	brwn	similar to RIKEN cDNA 1810030N24
RGD1562502	NM_001108818	10934548	1.31	44	1.6	pink	similar to RIKEN cDNA 2610029G23
RGD1564456	ENSRNOT00000048933	10796094	1.23	30	8.4	brwn	similar to chromosome 10 open reading frame 18
Mir99a	NR_031856	10749975	0.66	-33	0.0	grey	microRNA mir-99a
	FQ224515	10823595	1.49	161	1.4	green	
	FQ221747	10830328	1.28	32	0.0	grey	
	ENSRNOT00000070041	10772554	1.20	13	2.2	green	
	ENSRNOT00000069150	10868277	0.73	-11	2.3	black	
	ENSRNOT00000068804	10840100	1.43	11	2.5	green	
	ENSRNOT00000063593	10907802	0.49	-17	0.9	pink	
	ENSRNOT00000062069	10852719	0.66	-15	0.0	grey	
	ENSRNOT00000060731	10794222	0.81	-12	2.2	ylw	
	ENSRNOT00000057163	10705411	0.76	-160	1.8	pink	
	ENSRNOT00000056161	10913590	0.79	-16	0.0	grey	
	ENSRNOT00000055921	10849643	0.77	-11	2.6	blue	
	ENSRNOT00000055499	10819298	0.78	-13	0.0	grey	
	ENSRNOT00000054509	10795642	0.83	-13	2.2	tan	
	ENSRNOT00000054096	10764538	1.64	10	0.8	coral	
	ENSRNOT00000054014	10722427	1.50	16	3.0	pink	
	ENSRNOT00000053893	10722467	1.49	17	2.6	pink	
	**ENSRNOT00000053882**	**10722421**	**1.43**	**30**	**11.6**	**pink**	
	ENSRNOT00000053529	10722449	1.57	16	0.0	grey	
	ENSRNOT00000053496	10903998	0.75	-17	0.0	grey	
	ENSRNOT00000053463	10802706	0.67	-26	0.0	grey	
	ENSRNOT00000053290	10778375	1.22	20	0.0	grey	
	**ENSRNOT00000052822**	**10868241**	**1.45**	**12**	**4.5**	**black**	
	ENSRNOT00000052743	10935675	1.40	11	2.3	black	
	ENSRNOT00000052635	10871773	1.54	11	2.0	black	
	**ENSRNOT00000052564**	**10722431**	**1.45**	**32**	**8.9**	**pink**	
	ENSRNOT00000052447	10844062	1.51	19	0.0	grey	
	ENSRNOT00000052215	10773706	0.75	-15	0.0	grey	
	ENSRNOT00000051417	10841412	0.82	-103	0.0	grey	
	ENSRNOT00000050715	10772758	0.73	-107	2.3	black	
	**ENSRNOT00000033532**	**10925808**	**0.78**	**-11**	**3.6**	**black**	
	ENSRNOT00000032824	10760514	0.82	-23	0.0	grey	
	ENSRNOT00000029274	10803222	0.81	-15	0.0	grey	
	ENSRNOT00000029150	10751919	0.74	-13	0.0	grey	
	ENSRNOT00000027484	10790462	0.76	-11	0.0	grey	
	**ENSRNOT00000022886**	**10731009**	**1.28**	**60**	**10.1**	**brwn**	
	ENSRNOT00000022577	10719442	0.81	-10	0.0	grey	
	ENSRNOT00000010246	10861406	1.24	20	0.0	grey	
	ENSRNOT00000009205	10890017	0.80	-53	0.0	grey	
	ENSRNOT00000007237	10877569	0.74	-11	0.8	pink	
	ENSRNOT00000005636	10904230	0.78	-22	0.0	grey	
	ENSRNOT00000001163	10828152	0.73	-11	0.0	grey	
	AY539927	10868302	0.53	-49	0.4	coral	
	---	10700209	0.62	-99	1.4	ylw	
	---	10700650	0.70	-20	0.6	tan	
	---	10701059	0.65	-15	0.0	grey	
	---	10700525	0.66	-1135	5.3	turq	
	**---**	**10700366**	**0.54**	**-655**	**1.5**	**coral**	
	---	10700398	0.59	-460	6.6	turq	
	---	10701336	0.72	-401	4.4	turq	
	---	10700124	0.72	-258	8.3	turq	
	---	10700714	0.60	-229	6.2	turq	
	---	10700852	0.69	-217	0.0	grey	
	---	10701035	0.61	-140	4.2	pink	
	---	10701509	0.60	-136	2.9	pink	
	---	10700208	0.63	-106	2.5	blue	
	---	10700400	0.69	-97	0.0	grey	
	---	10701121	0.71	-84	0.0	grey	
	---	10700989	0.74	-63	0.8	pink	
	---	10701373	0.52	-58	0.0	grey	
	---	10700235	0.59	-49	0.0	grey	

	---	10701345	0.70	-43	0.0	grey	
	---	10701467	0.71	-42	0.9	green	
	---	10700605	0.63	-40	0.4	coral	
	---	10700662	0.63	-38	0.0	grey	
	---	10701227	0.69	-33	0.0	grey	
	---	10701273	0.50	-33	0.0	grey	
	---	10700759	0.64	-33	4.3	blue	
	---	10700389	0.52	-31	0.0	grey	
	---	10700142	0.45	-30	0.0	grey	
	---	10700332	0.54	-29	0.8	coral	
	---	10701256	0.61	-29	5.6	blue	
	---	10701234	0.65	-21	0.0	grey	
	---	10701355	0.55	-18	0.0	grey	
	---	10700767	0.58	-16	0.0	grey	
	---	10701606	0.53	-16	0.0	grey	
	---	10700227	0.80	-15	0.0	grey	
	---	10701055	0.83	-15	0.0	grey	
	---	10701180	0.75	-14	0.0	grey	
	---	10701008	0.68	-14	0.0	grey	
	---	10701203	0.46	-14	0.8	tan	
	---	10700308	0.65	-14	0.0	grey	
	---	10700159	0.82	-14	0.0	grey	
	---	10701123	0.54	-14	0.0	grey	
	---	10700377	0.65	-13	0.0	grey	
	---	10700237	0.55	-11	0.0	grey	
	---	10700488	0.64	-11	0.0	grey	
	---	10701551	1.57	16	0.0	grey	
	---	10700174	1.55	17	1.8	black	
	---	10700981	1.42	18	0.0	grey	
	---	10701486	1.39	24	0.0	grey	
	---	10700441	1.80	28	2.1	pink	
	---	10700190	1.23	46	0.0	grey	
	---	10700861	1.24	51	1.3	black	
	---	10700448	1.21	58	0.0	grey	
	---	10700512	1.20	65	0.0	grey	
	---	10701361	1.34	78	1.0	ylw	
	---	10700854	1.32	95	0.0	grey	
	---	10700568	1.21	98	0.0	grey	
	---	10701272	1.69	117	0.0	grey	
	---	10701020	1.21	126	0.0	grey	
	---	10701261	1.30	144	0.0	grey	
	---	10700920	1.21	188	7.4	brwn	
	---	10701500	1.23	189	0.0	grey	
	---	10700716	1.29	347	0.0	grey	
	---	10700960	1.29	482	2.4	ylw	
	---	10714204	0.71	-233	11.3	turq	
	---	10821067	0.76	-160	1.8	pink	
	**---**	**10813353**	**0.70**	**-140**	**4.6**	**black**	
	---	10710152	0.78	-35	2.0	black	
	---	10722191	0.72	-17	0.9	coral	
	---	10913940	0.74	-17	0.0	grey	
	---	10725567	0.76	-15	2.1	black	
	---	10930405	1.32	10	1.1	coral	
	---	10798220	1.39	12	1.6	pink	
	---	10778675	1.38	19	2.4	green	
	---	10937568	1.51	22	0.0	grey	
	---	10902292	1.22	36	4.5	turq	
	---	10811501	1.24	38	0.0	grey	
	---	10805722	1.27	51	0.0	grey	
	---	10769693	1.32	72	0.0	grey	

E. Uterus

Gene Symbol	RefSeq/GenBank/ Ensemble Number	Affymetrix Probset ID	V/C	Mean Diff.	k.in	Mod	Gene Title
**APOPTOSIS**							
Bag3	NM_001011936	10711401	0.74	-65	3.2	salmor	Bcl2-associated athanogene 3
Dapk1	NM_001107335	10797032	0.71	-44	0.0	grey	death associated protein kinase 1

Sharpin	NM_031153	10904910	0.81	-74	3.8	red	SHANK-associated RH domain interactor
Tnfrsf1a	NM_013091	10858967	0.75	-79	0.0	grey	tumor necrosis factor receptor superfamily, member 1a
**CELL CYCLE**							
Anapc1	NM_001107771	10849765	1.22	48	3.1	brwn	anaphase promoting complex subunit 1
**Cep350**	**ENSRNOT00000005235**	**10768874**	**1.21**	**31**	**8.5**	**brwn**	**centrosomal protein 350kDa**
Mnd1	ENSRNOT00000031179	10824010	1.44	10	0.0	grey	meiotic nuclear divisions 1 homolog (S. cerevisiae)
Ptms	NM_031975	10865607	0.81	-101	3.0	red	parathymosin
**CYTOSKELETON-ECM**							
Cldn10	NM_001106058	10782020	1.51	28	4.5	red	claudin 10
Fblim1	NM_001007554	10881124	0.77	-16	0.0	grey	filamin binding LIM protein 1
Fbln1	NM_001127547	10898315	0.79	-31	0.4	gr60	fibulin 1
Lamb2	NM_012974	10793140	0.71	-126	1.2	salmor	laminin, beta 2
LOC691984	ENSRNOT00000050826	10781999	1.47	21	0.0	grey	similar to Glypican-6 precursor
Lrrc32	NM_001170434	10708896	0.69	-11	4.2	green	leucine rich repeat containing 32
Map1b	NM_019217	10820835	0.83	-15	0.0	grey	microtubule-associated protein 1B
**Mast4**	**ENSRNOT00000061032**	**10821072**	**0.81**	**-20**	**3.3**	**salmor**	**microtubule associated serine/threonine kinase family member 4**
Smchd1	ENSRNOT00000019359	10930435	1.24	67	7.2	brwn	structural maintenance of chromosomes flexible hinge domain containing 1
Tspan12	NM_001015026	10861213	1.48	409	3.1	salmor	tetraspanin 12
Zyx	NM_053761	10855062	0.66	-52	3.7	red	zyxin
**DEVELOPMENT**							
Egln2	NM_001004083	10719958	0.81	-42	1.3	salmor	EGL nine homolog 2 (C. elegans)
Fhl3	NM_001107979	10871806	0.67	-26	0.0	grey	four and a half LIM domains 3
Gjb2	NM_001004099	10784117	0.55	-55	0.0	grey	gap junction protein, beta 2
Ihh	NM_053384	10929027	1.54	26	0.0	grey	Indian hedgehog
LOC301748	ENSRNOT00000048256	10861620	1.92	31	3.7	purple	similar to RIKEN cDNA 1700001E04
**LOC501091**	**ENSRNOT00000046852**	**10921318**	**1.79**	**30**	**4.6**	**purple**	**similar to Discs large homolog 5 (Placenta and prostate DLG) (Discs large protein P-dlg) (chr9)**
**LOC501091**	**ENSRNOT00000046852**	**10772815**	**1.87**	**27**	**3.7**	**purple**	**similar to Discs large homolog 5 (Placenta and prostate DLG) (Discs large protein P-dlg) (chr14)**
Lrpprc	NM_001008519	10888210	1.25	153	3.6	brwn	leucine-rich PPR-motif containing
Nde1	NM_053347	10740309	1.23	30	0.0	grey	nudE nuclear distribution gene E homolog 1 (A. nidulans)
Neu1	NM_031522	10831300	0.75	-56	3.7	red	sialidase 1 (lysosomal sialidase)
Neurl2	NM_001107802	10851667	0.81	-12	3.6	red	neuralized homolog 2 (Drosophila)
Osgin2	ENSRNOT00000012394	10875642	1.24	25	0.0	grey	oxidative stress induced growth inhibitor family member 2
Popdc2	NM_199113	10754179	0.59	-64	1.4	m-blu	popeye domain containing 2
Porcn	NM_001173355	10932394	0.65	-20	4.3	brwn	porcupine homolog (Drosophila)
Pzp	NM_145779	10865868	1.23	28	0.0	grey	pregnancy-zone protein
RGD1306820	NM_001107557	10711743	1.32	26	2.0	purple	similar to erythroid differentiation-related factor 1
Rsrc2	NM_001014128	10758354	1.21	50	3.0	brwn	arginine/serine-rich coiled-coil 2
Setx	ENSRNOT00000017984	10834908	1.39	140	6.7	turq	senataxin
Sfrp4	NM_053544	10798610	0.55	-31	0.0	grey	secreted frizzled-related protein 4
Skil	ENSRNOT00000013191	10822637	1.27	52	3.7	brwn	SKI-like oncogene
Sox6	NM_001024751	10725060	1.26	13	0.0	grey	SRY (sex determining region Y)-box 6
Synj2	NM_001113371	10702829	0.69	-22	0.0	grey	synaptojanin 2
Urg4	NM_001077660	10778214	0.83	-32	2.4	salmor	up-regulated gene 4
**DNA REPAIR**							
Cry2	NM_133405	10847525	0.78	-56	2.6	salmor	cryptochrome 2 (photolyase-like)
**ELECTRON TRANSPORT**							
Cyp2d4	NM_138515	10905721	0.77	-15	2.5	salmor	cytochrome P450, family 2, subfamily d, polypeptide 4
Cyp2d5	NM_173304	10905732	1.23	11	0.0	grey	cytochrome P450, family 2, subfamily d, polypeptide 5
**EPIGENETICS**							
Mtrr	NM_001039003	10793369	1.20	50	6.8	turq	5-methyltetrahydrofolate-homocysteine methyltransferase reductase
**GROWTH FACTORS**							
Angpt1	NM_053546	10903529	1.40	58	2.3	red	angiopoietin 1
Gdf10	NM_024375	10786905	1.75	11	1.5	salmor	growth differentiation factor 10
Tgfb1i1	NM_001191840	10711364	0.71	-16	0.0	grey	transforming growth factor beta 1 induced transcript 1
**IMMUNE RESPONSE**							
Bat2d1	ENSRNOT00000030419	10869661	1.22	68	0.0	grey	BAT2 domain containing 1
C1qb	NM_019262	10880727	0.76	-40	0.0	grey	complement component 1, q subcomponent, B chain
Cd8a	NM_031538	10856274	3.04	32	0.0	grey	CD8a molecule
Ifitm3	NM_001136124	10726682	0.80	-336	0.0	grey	interferon induced transmembrane protein 3
Lingo4	NM_001109189	10817222	0.71	-37	2.0	brwn	leucine rich repeat and Ig domain containing 4
Lyar	NM_001011911	10777328	1.47	41	0.0	grey	Ly1 antibody reactive homolog (mouse)
Toag1	NM_001110838	10914442	1.30	73	4.9	brwn	tolerance-associated gene 1
**METABOLISM & TRANSPORT**							
Acsl3	NM_057107	10924672	1.32	95	1.4	gr60	acyl-CoA synthetase long-chain family member 3
Adi1	NM_199097	10883982	1.21	76	1.8	purple	acireductone dioxygenase 1
**Atp6v0c**	**NM_130823**	**10740996**	**0.73**	**-459**	**6.9**	**red**	**ATPase, H+ transporting, lysosomal 16kDa, V0 subunit c**
Atp6v1a	ENSRNOT00000035482	10823611	1.33	12	0.4	brwn	ATPase, H+ transporting, lysosomal V1 subunit A

Cacna1c	NM_012517	10865016	0.65	-20	0.0	grey	calcium channel, voltage-dependent, L type, alpha 1C subunit
Cdo1	NM_052809	10804396	4.34	992	1.8	red	cysteine dioxygenase, type I
**Cmbl**	**NM_001008770**	**10814105**	**1.67**	**456**	**2.2**	**gr60**	**carboxymethylenebutenolidase homolog (Pseudomonas)**
Crls1	NM_001014258	10840131	1.21	132	1.2	purple	cardiolipin synthase 1
Elovl6	NM_134383	10819005	2.34	86	3.1	green	ELOVL family member 6, elongation of long chain fatty acids (yeast)
Gstm1	NM_017014	10825925	2.52	921	0.9	green	glutathione S-transferase mu 1
Hba-a2	NM_013096	10741756	1.67	3985	4.5	m-blu	hemoglobin alpha, adult chain 2
Hbb	NM_033234	10724315	2.04	1551	4.7	m-blu	hemoglobin, beta
Hbb-b1	NM_198776	10724319	2.20	1599	4.5	m-blu	hemoglobin, beta adult major chain
Hsd17b12	NM_032066	10847646	1.21	95	0.0	grey	hydroxysteroid (17-beta) dehydrogenase 12
Ivd	NM_012592	10838704	1.55	357	1.7	green	isovaleryl coenzyme A dehydrogenase
Kynu	NM_053902	10836019	1.37	26	0.0	grey	kynureninase (L-kynurenine hydrolase)
**LOC689064**	**NM_001111269**	**10724311**	**2.04**	**1671**	**4.9**	**m-blu**	**beta-globin**
Mdh1	NM_033235	10778661	1.23	571	4.8	brwn	malate dehydrogenase 1, NAD (soluble)
Ndufs1	NM_001005550	10928486	1.24	409	7.7	brwn	NADH dehydrogenase (ubiquinone) Fe-S protein 1
Nup205	NM_001108620	10854494	1.41	34	0.0	grey	nucleoporin 205
Nup93	NM_001011925	10809370	1.21	22	0.0	grey	nucleoporin 93
Pank3	NM_001108272	10732789	1.25	127	4.4	brwn	pantothenate kinase 3
Pcca	NM_019330	10782166	1.24	134	3.8	brwn	propionyl-coenzyme A carboxylase, alpha polypeptide
Phgdh	NM_031620	10825328	1.26	15	0.0	grey	phosphoglycerate dehydrogenase
Pnpt1	NM_001142371	10774652	1.21	73	5.0	brwn	polyribonucleotide nucleotidyltransferase 1
Prr12	ENSRNOT00000038067	10721616	0.76	-12	1.3	brwn	proline rich 12
Ptgis	NM_031557	10851947	0.68	-24	0.0	grey	prostaglandin I2 (prostacyclin) synthase
RGD1564228	ENSRNOT00000052157	10738261	0.73	-29	1.6	red	similar to Naglu
Scamp4	NM_031725	10900478	0.80	-48	3.7	red	secretory carrier membrane protein 4
Slc15a1	NM_057121	10785943	1.77	19	0.0	grey	solute carrier family 15 (oligopeptide transporter), member 1
Slc16a2	NM_147216	10938830	0.81	-49	2.3	red	solute carrier family 16, member 2 (monocarboxylic acid transporter 8)
Slc27a1	NM_053580	10787313	0.81	-44	1.2	salmon	solute carrier family 27 (fatty acid transporter), member 1
Slc44a2	NM_001134715	10908369	0.81	-57	3.1	salmon	solute carrier family 44, member 2
St3gal4	NM_203337	10916016	0.73	-61	0.0	grey	ST3 beta-galactoside alpha-2,3-sialyltransferase 4
Timm17a	NM_019351	10890170	1.23	83	0.0	grey	translocase of inner mitochondrial membrane 17 homolog A (yeast)
Xpo4	NM_001106042	10784140	1.28	46	6.0	brwn	exportin 4
**PROTEOLYSIS**							
Lonrf2	ENSRNOT00000033964	10927588	1.50	10	1.0	purple	LON peptidase N-terminal domain and ring finger 2
**Mgrn1**	**NM_001013964**	**10740649**	**0.77**	**-78**	**8.8**	**red**	**mahogunin, ring finger 1**
Mmp16	NM_080776	10867761	1.36	11	0.0	grey	matrix metallopeptidase 16
Prss12	NM_053504	10818880	1.32	11	0.0	grey	protease, serine, 12 neurotrypsin (motopsin)
Rnf144b	NM_001108881	10797728	0.71	-60	0.0	grey	ring finger protein 144B
Serpine1	NM_012620	10761047	0.69	-16	0.0	grey	serine (or cysteine) peptidase inhibitor, clade E, member 1
Spsb1	NM_001107994	10881820	0.71	-25	2.2	brwn	splA/ryanodine receptor domain and SOCS box containing 1
Ubc	NM_017314	10758134	0.73	-282	0.0	grey	ubiquitin C
Usp30	NM_001107153	10762857	0.77	-15	4.0	red	ubiquitin specific peptidase 30
Usp46	NM_001191596	10772306	1.26	51	0.6	gr60	ubiquitin specific peptidase 46
**RECEPTORS & BINDING PROTEINS**							
LOC100363145	ENSRNOT00000025394	10790023	0.72	-22	1.4	red	stabilin 1
Ltbp3	NM_001191561	10713123	0.83	-19	1.0	red	latent transforming growth factor beta binding protein 3
Ltbp4	NM_001170336	10720000	0.66	-107	1.9	gr60	latent transforming growth factor beta binding protein 4
Mnt	NM_001105807	10735818	0.70	-12	0.0	grey	max binding protein
Mrc1	NM_001106123	10796476	0.82	-50	0.0	grey	mannose receptor, C type 1
Stambp	NM_138531	10863559	0.74	-48	2.1	red	Stam binding protein
Tfrc	NM_022712	10754785	1.89	236	0.0	grey	transferrin receptor
**Tgfbr3**	**NM_017256**	**10771070**	**0.62**	**-179**	**2.5**	**gr60**	**transforming growth factor, beta receptor III**
Thra	NM_031134	10738056	0.77	-21	1.0	gr60	thyroid hormone receptor alpha
Vom1r1	ENSRNOT00000060021	10718152	1.23	14	0.8	gr60	vomeronasal 1 receptor, 1
**SIGNALING**							
Agfg2	NM_001107131	10760994	0.74	-28	1.8	gr60	ArfGAP with FG repeats 2
Akap2	NM_001011974	10869204	0.80	-31	1.0	l-cn	A kinase (PRKA) anchor protein 2
Cyr61	NM_031327	10827231	0.64	-61	0.0	grey	cysteine-rich, angiogenic inducer, 61
Dbi	NM_031853	10931154	1.43	199	0.0	grey	diazepam binding inhibitor (GABA receptor modulator, acyl-Coenzyme A binding protein)
Errfi1	NM_001014071	10874193	0.82	-204	1.7	red	ERBB receptor feedback inhibitor 1
Gpr107	NM_001107828	10835303	0.83	-77	0.0	grey	G protein-coupled receptor 107
Gpr88	NM_031696	10826261	0.31	-32	0.8	l-yel	G-protein coupled receptor 88
**Hspa4**	**NM_153629**	**10742575**	**1.23**	**307**	**10.0**	**brwn**	**heat shock protein 4**
Hspa4l	NM_001106428	10815099	1.53	63	3.0	brwn	heat shock protein 4 like
Hspa9	NM_001100658	10803851	1.20	506	3.5	brwn	heat shock protein 9
Hspb11	NM_001131002	10870661	1.28	18	2.6	red	heat shock protein family B (small), member 11
Hspd1	NM_022229	10928154	1.36	726	0.0	grey	heat shock protein 1 (chaperonin)
Mapk1ip1l	NM_001108373	10779671	0.80	-75	1.6	salmon	mitogen-activated protein kinase 1 interacting protein 1-like

**Mbrl**	**NM_001100886**	**10893961**	**0.80**	**-100**	**7.6**	**red**	**membralin**
Mknk1	NM_001044267	10871133	0.78	-60	0.0	grey	MAP kinase-interacting serine/threonine kinase 1
Mst4	NM_001191736	10935418	1.36	15	0.0	grey	serine/threonine protein kinase MST4
Nmur2	NM_022275	10742835	0.53	-18	0.8	purple	neuromedin U receptor 2
Pde3a	NM_017337	10859494	0.75	-36	0.9	black	phosphodiesterase 3A, cGMP inhibited
Pdk3	NM_001106581	10938384	1.24	29	1.3	brwn	pyruvate dehydrogenase kinase, isozyme 3
Peli2	NM_001107259	10779726	0.68	-47	3.4	red	pellino 2
Phlda1	NM_017180	10895406	1.26	29	0.0	grey	pleckstrin homology-like domain, family A, member 1
Pign	NM_001100584	10766926	1.25	72	6.6	brwn	phosphatidylinositol glycan, class N
Plekhm2	NM_001191767	10881146	0.80	-28	2.1	salmon	pleckstrin homology domain containing, family M (with RUN domain) member 2
Plekho1	NM_001025119	10825113	0.70	-97	2.9	red	pleckstrin homology domain containing, family O member 1
Prkaa2	NM_023991	10878416	0.78	-107	0.0	grey	protein kinase, AMP-activated, alpha 2 catalytic subunit
Prkar2b	NM_001030020	10889590	1.61	45	0.0	grey	protein kinase, cAMP dependent regulatory, type II beta
Ptpn4	NM_001100479	10767132	1.23	63	11.3	turq	protein tyrosine phosphatase, non-receptor type 4
Ptprv	NM_033099	10764192	0.74	-10	0.0	grey	protein tyrosine phosphatase, receptor type, V
RGD1564641	ENSRNOT00000002443	10754592	1.28	44	0.0	grey	similar to Neurogenic locus notch homolog protein 2 precursor (Notch 2) (hN2)
Ripk4	NM_001107103	10753461	1.27	11	0.0	grey	receptor-interacting serine-threonine kinase 4
Seli	NM_001134754	10889064	1.77	387	4.2	brwn	selenoprotein I
Tbc1d7	NM_001108411	10794576	0.80	-22	0.0	grey	TBC1 domain family, member 7
**Tns1**	**NM_001191810**	**10928855**	**0.66**	**-303**	**3.4**	**salmon**	**tensin 1**
**Tom1**	**NM_001008365**	**10806106**	**0.78**	**-60**	**6.6**	**red**	**target of myb1 homolog (chicken)**
**TRANSCRIPTION**							
Ankrd26	ENSRNOT00000056301	10864979	1.29	15	1.0	purple	ankyrin repeat domain 26
Bcl6	NM_001107084	10751931	0.74	-11	0.4	m-blu	B-cell CLL/lymphoma 6
**Btaf1**	**NM_001191917**	**10715025**	**1.24**	**64**	**9.6**	**brwn**	**BTAF1 RNA polymerase II, B-TFIID transcription factor-associated, (Mot1 homolog, S. cerevisiae)**
Btg1	NM_017258	10895069	0.76	-234	0.0	grey	B-cell translocation gene 1, anti-proliferative
Ccdc124	NM_001106071	10787420	1.27	16	3.3	green	coiled-coil domain containing 124
Ccdc85b	ENSRNOT00000027896	10727854	0.81	-12	0.0	grey	coiled-coil domain containing 85B
Cirbp	NM_031147	10900583	0.68	-98	0.6	gr60	cold inducible RNA binding protein
Ddx18	NM_001006996	10767189	1.20	48	4.4	brwn	DEAD (Asp-Glu-Ala-Asp) box polypeptide 18
Dnajc30	NM_001109024	10761225	0.76	-19	2.6	red	DnaJ (Hsp40) homolog, subfamily C, member 30
Fat4	NM_001191705	10815057	1.27	13	0.0	grey	FAT tumor suppressor homolog 4 (Drosophila)
Fbxl6	NM_001005563	10904976	0.76	-26	1.2	salmon	F-box and leucine-rich repeat protein 6
Fyn	NM_012755	10830382	0.81	-39	0.0	grey	FYN oncogene related to SRC, FGR, YES
Ino80b	NM_001048233	10863496	0.81	-11	0.0	grey	INO80 complex subunit B
Ino80e	NM_001013900	10725846	0.73	-50	5.2	red	INO80 complex subunit E
Kbtbd8	NM_001109250	10857423	1.37	12	1.7	green	kelch repeat and BTB (POZ) domain containing 8
Klf7	NM_001108800	10928524	0.79	-23	0.0	grey	Kruppel-like factor 7 (ubiquitous)
Klhdc3	NM_001012203	10926441	0.80	-111	3.5	red	kelch domain containing 3
Klhl11	NM_001105838	10747368	1.21	52	2.0	purple	kelch-like 11 (Drosophila)
Klhl29	NM_001106713	10889156	0.75	-12	1.8	purple	kelch-like 29 (Drosophila)
Mrvi1	NM_001105211	10724902	0.72	-31	1.2	gr60	murine retrovirus integration site 1 homolog
Nasp	NM_001005543	10878880	1.21	19	3.6	brwn	nuclear autoantigenic sperm protein (histone-binding)
Ndrg1	NM_001011991	10904169	0.74	-88	0.0	grey	N-myc downstream regulated gene 1
Nfix	NM_030866	10806665	0.79	-103	2.1	gr60	nuclear factor I/X (CCAAT-binding transcription factor)
**Noc3l**	**NM_001108523**	**10729999**	**1.31**	**38**	**8.6**	**brwn**	**nucleolar complex associated 3 homolog (S. cerevisiae)**
Npas2	NM_001108214	10922745	0.76	-17	0.0	grey	neuronal PAS domain protein 2
Nudt5	NM_001007733	10799539	1.25	42	0.0	grey	nudix (nucleoside diphosphate linked moiety X)-type motif 5
Pcif1	NM_001108605	10842221	0.72	-45	0.0	grey	PDX1 C-terminal inhibiting factor 1
Polr3b	NM_001191878	10901328	1.24	32	0.8	l-yel	polymerase (RNA) III (DNA directed) polypeptide B
**Rela**	**NM_199267**	**10713089**	**0.71**	**-87**	**7.4**	**red**	**v-rel reticuloendotheliosis viral oncogene homolog A (avian)**
Serf1	ENSRNOT00000024291	10812734	1.53	50	1.2	gr60	small EDRK-rich factor 1
Src	NM_031977	10841624	0.77	-16	3.6	red	v-src sarcoma (Schmidt-Ruppin A-2) viral oncogene homolog (avian)
Tcfe3	ENSRNOT00000012829	10937048	0.76	-35	4.7	red	transcription factor E3
Thoc4	NM_001109602	10749681	1.36	108	1.6	brwn	THO complex 4
Trip4	NM_001134981	10918302	1.21	51	8.8	turq	thyroid hormone receptor interactor 4
Tshz1	ENSRNOT00000021750	10805511	0.82	-35	3.3	salmon	teashirt zinc finger homeobox 1
Zbtb11	NM_001107097	10753720	1.33	51	1.2	purple	zinc finger and BTB domain containing 11
Zbtb22	NM_001009172	10831790	0.80	-18	3.1	red	zinc finger and BTB domain containing 22
Zbtb4	ENSRNOT00000019687	10735015	0.80	-29	0.5	gr60	zinc finger and BTB domain containing 4
Zfp148	NM_031615	10754629	1.22	37	5.0	brwn	zinc finger protein 148
Zfp395	NM_001107271	10781100	0.76	-22	0.0	grey	zinc finger protein 395
Znf580	ENSRNOT00000021772	10718825	0.76	-13	0.0	grey	zinc finger protein 580
Znf688	ENSRNOT00000024858	10726018	0.75	-27	1.1	red	zinc finger protein 688
Zswim4	NM_001107163	10806709	0.75	-11	0.0	grey	zinc finger, SWIM-type containing 4
**TRANSLATION & PROTEIN MODIFICATION**							
Cct8	NM_001105897	10752903	1.22	313	3.8	brwn	chaperonin containing Tcp1, subunit 8 (theta)
Ell	NM_001107304	10790978	0.75	-30	4.9	red	elongation factor RNA polymerase II

Hnrnpa3	NM_001111295	10883583	1.21	378	0.0	grey	heterogeneous nuclear ribonucleoprotein A3
Ints2	BC128757	10745871	1.27	19	2.8	brwn	integrator complex subunit 2
**Lars**	**NM_001009637**	**10804187**	**1.24**	**82**	**8.7**	**brwn**	**leucyl-tRNA synthetase**
Polr1b	NM_031773	10839751	1.23	21	0.0	grey	polymerase (RNA) I polypeptide B
Qrsl1	NM_001014034	10833837	1.25	21	1.7	green	glutaminyl-tRNA synthase (glutamine-hydrolyzing)-like 1
Rbm8a	ENSRNOT00000028807	10817573	1.25	55	0.0	grey	RNA binding motif protein 8A
RGD1561067	ENSRNOT00000046138	10788569	0.68	-161	1.4	salmor	similar to RNA binding protein gene with multiple splicing
Rnasek	NM_001137561	10744391	0.78	-655	6.5	red	ribonuclease, RNase K
Sf3a3	NM_001025698	10871813	1.25	42	0.0	grey	splicing factor 3a, subunit 3
Sf3b4	NM_001011951	10817504	0.80	-19	4.8	red	splicing factor 3b, subunit 4
Sh3bp2	NM_001100684	10777677	0.75	-13	3.3	red	SH3-domain binding protein 2
Snapc2	NM_001013121	10759531	0.69	-51	3.3	red	small nuclear RNA activating complex, polypeptide 2
**MISCELLANEOUS & UNKNOWN**							
Carkd	ENSRNOT00000065043	10789574	1.23	18	0.0	grey	carbohydrate kinase domain containing
Dd25	NM_199403	10769236	1.60	194	1.8	purple	hypothetical protein Dd25
Fam100a	BC079459	10731734	0.70	-40	5.3	red	family with sequence similarity 100, member A
Fam189b	ENSRNOT00000027828	10816712	0.69	-15	0.9	gr60	family with sequence similarity 189, member B
Fam43a	NM_001039002	10754939	0.80	-23	0.0	grey	family with sequence similarity 43, member A
Lin37	NM_001106245	10720648	0.81	-39	2.2	salmor	lin-37 homolog (C. elegans)
LOC100361389	ENSRNOT00000028563	10747847	0.73	-10	3.8	brwn	hypothetical protein LOC100361389
LOC689574	NM_001195503	10854540	0.75	-273	5.1	red	hypothetical protein LOC689574
LOC689826	ENSRNOT00000047742	10880607	0.77	-11	2.8	green	hypothetical protein LOC689826
Nacc2	NM_001100533	10843647	0.81	-24	2.5	salmor	nucleus accumbens associated 2, BEN and BTB (POZ) domain containing
Nkd1	BC168970	10809678	0.78	-11	1.8	gr60	naked cuticle homolog 1 (Drosophila)
Pqlc1	NM_001013189	10802956	0.78	-44	2.5	red	PQ loop repeat containing 1
RGD1311849	ENSRNOT00000058387	10869839	1.43	32	0.8	green	similar to mKIAA1797 protein
RGD1559954	XM_002724794	10757249	1.37	22	0.0	grey	similar to family with sequence similarity 55, member C
RGD1564615	ENSRNOT00000018425	10855765	1.46	59	4.4	red	similar to mKIAA0241 protein
Tmem50b	NM_001025014	10753135	1.26	136	0.0	grey	transmembrane protein 50B
**EST's**							
LOC100361915	ENSRNOT00000028495	10728136	0.83	-31	2.8	salmor	chromosome 11 open reading frame 2-like
LOC501224	BC079060	10930632	1.71	23	2.9	purple	similar to RIKEN cDNA 2610042L04 (chrUn)
LOC501224	BC079060	10772821	1.71	29	3.3	purple	similar to RIKEN cDNA 2610042L04 (chr14)
MGC94207	BC081992	10905124	0.80	-45	4.6	red	similar to RIKEN cDNA C030006K11
RGD1305158	BC167112	10875932	1.25	26	1.9	brwn	similar to RIKEN cDNA 1810030N24
RGD1306583	BC088172	10821134	1.22	152	2.1	red	similar to RIKEN cDNA 2410002O22 gene
RGD1559896	NM_001109134	10811906	0.78	-55	1.2	gr60	similar to RIKEN cDNA 2310022B05
RGD1565096	NM_001134611	10725270	1.34	16	0.4	l-yel	similar to TSG118.1
Mir143	NR_031889	10804712	0.64	-15	0.0	grey	microRNA mir-143
Mir145	NR_031891	10804710	0.61	-21	0.0	grey	microRNA mir-145
Mir22	NR_031824	10735887	0.76	-20	1.5	purple	microRNA mir-22
Mirlet7a-2	NR_031801	10909373	0.65	-11	0.0	grey	microRNA let-7a-2
	ENSRNOT00000063746	10800599	0.68	-12	0.9	purple	
	ENSRNOT00000056831	10847901	0.79	-20	0.0	grey	
	ENSRNOT00000055440	10859772	1.55	12	0.0	grey	
	ENSRNOT00000055404	10899204	1.25	129	4.9	brwn	
	ENSRNOT00000054583	10870010	0.79	-10	0.0	grey	
	ENSRNOT00000054033	10733721	1.38	16	0.0	grey	
	ENSRNOT00000053807	10909360	1.55	205	1.2	green	
	ENSRNOT00000053549	10886261	1.33	14	1.0	gr-yel	
	**ENSRNOT00000053327**	**10722377**	**1.73**	**16**	**15.4**	**green**	
	ENSRNOT00000053268	10909356	1.35	274	1.8	gr-yel	
	ENSRNOT00000052939	10816564	1.46	17	0.0	grey	
	ENSRNOT00000052253	10802934	1.30	13	0.0	grey	
	ENSRNOT00000042358	10779954	1.22	14	0.0	grey	
	ENSRNOT00000037885	10885903	0.71	-23	0.0	grey	
	ENSRNOT00000037100	10767987	0.80	-13	0.0	grey	
	ENSRNOT00000035247	10755897	1.25	44	15.6	turq	
	ENSRNOT00000034355	10920991	0.68	-30	0.0	grey	
	ENSRNOT00000010988	10918706	0.77	-10	0.9	pink	
	ENSRNOT00000008321	10884080	1.21	61	0.0	grey	
	BC105775	10878999	0.82	-13	1.9	brwn	
	---	10700081	0.76	-21	0.0	grey	
	---	10701567	0.55	-10	0.7	m-blu	
	---	10700878	1.77	15	0.0	grey	
	---	10700809	1.77	36	1.4	purple	
	---	10701313	0.80	-129	0.0	grey	
	---	10701106	0.81	-69	0.0	grey	
	---	10701143	0.63	-29	0.0	grey	

	---	10700642	0.53	-29	0.0	grey	
	---	10701577	0.57	-22	0.0	grey	
	---	10701197	0.70	-17	0.0	grey	
	---	10700899	0.62	-16	0.0	grey	
	---	10700724	1.91	11	2.8	turq	
	---	10700198	1.38	13	1.5	green	
	---	10701192	1.25	13	0.0	grey	
	---	10700452	1.54	14	0.0	grey	
	---	10700415	1.29	28	5.6	turq	
	---	10700227	2.07	51	0.0	grey	
	---	10700691	1.30	316	2.7	brwn	
	---	10767985	0.82	-32	0.0	grey	
	---	10866193	0.78	-12	5.0	red	
	---	10898834	1.52	17	0.0	grey	
	---	10710152	1.24	30	2.0	black	
	---	10772272	1.24	43	1.8	red	

F. Male Heart

Gene Symbol	RefSeq/GenBank/ Ensemble Number	Affymetrix Probset ID	V/C	Mean Diff.	k.in	Mod	Gene Title
**CELL CYCLE**							
Cdc73	NM_001024769	10768305	1.25	72	0	grey	cell division cycle 73, Paf1/RNA polymerase II complex component, homolog (S. cerevisiae)
Cp110	NM_001108501	10710179	0.70	-13	6.7	brwn	CP110 protein
Ddit4	NM_080906	10832920	0.52	-56	14.2	blue	DNA-damage-inducible transcript 4
Cdon	NM_017358	10908968	0.69	-29	18.4	brwn	Cdon homolog (mouse)
Col4a4	NM_001008332	10929396	0.76	-12	0	grey	collagen, type IV, alpha 4
Fn1	NM_019143	10928761	0.56	-111	10.9	brwn	fibronectin 1
Kif5b	NM_057202	10795548	1.23	212	0	grey	kinesin family member 5B
Lad1	NM_001107942	10764240	1.46	24	0	grey	ladinin 1
Ninj1	NM_012867	10797705	0.81	-28	0	grey	ninjurin 1
Tspan4	NM_001013070	10712349	0.64	-109	0	grey	tetraspanin 4
Tspan8	NM_133526	10895499	0.70	-97	6.4	blue	tetraspanin 8
Tuba1c	NM_001011995	10899055	0.79	-164	0	grey	tubulin, alpha 1C
**DEVELOPMENT**							
Homez	NM_152849	10783662	0.74	-37	0	grey	homeobox and leucine zipper encoding
Mal	NM_012798	10849700	0.77	-22	13.0	brwn	mal, T-cell differentiation protein
Ophn1	NM_001107848	10938550	1.27	12	0	grey	oligophrenin 1
Per1	NM_001034125	10734882	0.58	-43	0	grey	period homolog 1 (Drosophila)
Slit3	NM_031321	10732750	0.70	-19	12.4	brwn	slit homolog 3 (Drosophila)
Sntb2	NM_001168674	10807601	0.78	-28	0	grey	syntrophin, beta 2
Tor2a	NM_001007744	10835618	0.75	-33	18.4	brwn	torsin family 2, member A
Vangl1	NM_001109584	10825512	1.24	14	0.3	ylw	vang-like 1 (van gogh, Drosophila)
Rapgef4	AB031229	10836890	0.74	-16	0	grey	Rap guanine nucleotide exchange factor (GEF) 4
Ropn1	NM_001025628	10754556	0.71	-14	5.43	green	ropporin, rhophilin associated protein 1
**ELECTRON TRANSPORT**							
Cox6b2	NM_001039085	10703970	0.83	-14	7.10	green	cytochrome c oxidase subunit VIb polypeptide 2
Cyp2j4	NM_023025	10878157	1.35	30	7.2	brwn	cytochrome P450, family 2, subfamily j, polypeptide 4
**EPIGENETICS**							
Ar	NM_012502	10934118	0.79	-54	0	grey	androgen receptor
Brd1	NM_001108103	10906041	0.83	-40	0	grey	bromodomain containing 1
**GOLGI APPARATUS**							
Hs3st1	NM_053391	10773180	0.73	-17	0	grey	heparan sulfate (glucosamine) 3-O-sulfotransferase 1
**GROWTH FACTORS**							
Ccl21b	NM_001008513	10876208	0.79	-13	5.2	ylw	chemokine (C-C motif) ligand 21b
Figf	NM_031761	10937725	0.66	-27	10.3	brwn	c-fos induced growth factor
**IMMUNE RESPONSE**							
Cd274	NM_001191954	10714694	1.39	12	19.2	brwn	CD274 molecule
Cd4	NM_012705	10865585	1.24	31	14.0	brwn	Cd4 molecule
**METABOLISM & TRANSPORT**							
Agpat4	NM_133406	10718001	0.79	-12	0	grey	1-acylglycerol-3-phosphate O-acyltransferase 4 (lysophosphatidic acid acyltransferase, delta)
Alg14	NM_001014176	10818690	0.75	-58	9.0	brwn	asparagine-linked glycosylation 14 homolog (S. cerevisiae)
B3gnt5	NM_053932	10755544	1.20	18	7.4	brwn	UDP-GlcNAc:betaGal beta-1,3-N-acetylglucosaminyltransferase 5
Edem2	NM_001004230	10851076	0.77	-56	19.6	brwn	ER degradation enhancer, mannosidase alpha-like 2
Ephx1	NM_001034090	10770342	0.77	-288	5.0	brwn	epoxide hydrolase 1, microsomal
Etnk2	ENSRNOT00000032489	10764034	0.83	-12	0	grey	ethanolamine kinase 2
Gltp	NM_001134413	10759089	0.79	-25	22.3	blue	glycolipid transfer protein

Gtdc1	NM_001024274	10845060	1.26	25	0	grey	glycosyltransferase-like domain containing 1
Hsd17b11	NM_001004209	10771224	0.81	-120	0	grey	hydroxysteroid (17-beta) dehydrogenase 11
LOC681355	ENSRNOT00000059744	10932891	1.40	10	2.2	brwn	similar to potassium channel tetramerisation domain containing 12b
LOC685152	ENSRNOT00000056606	10824550	0.69	-16	0	grey	similar to Probable phospholipid-transporting ATPase ID (ATPase class I type 8B member 2)
Mthfd2	NM_001109398	10870316	0.81	-20	8.1	ylw	methylenetetrahydrofolate dehydrogenase (NADP+ dependent) 2, methenyltetrahydrofolate cyclohydrolase
Nme3	NM_053507	10732257	0.78	-41	11.4	blue	non-metastatic cells 3, protein expressed in
Nup37	NM_001106775	10894712	0.82	-30	0	grey	nucleoporin 37
Pcbd1	NM_001007601	10830003	0.81	-93	1.2	brwn	pterin-4 alpha-carbinolamine dehydratase/dimerization cofactor of hepatocyte nuclear factor 1 alpha
Pdia5	NM_001014125	10751498	0.78	-15	0	grey	protein disulfide isomerase family A, member 5
Ppic	NM_001004215	10804480	0.82	-35	0	grey	peptidylprolyl isomerase C
Slc29a3	NM_181639	10833005	0.83	-23	11.8	turq	solute carrier family 29 (nucleoside transporters), member 3
St3gal4	NM_203337	10916016	0.78	-20	8.0	brwn	ST3 beta-galactoside alpha-2,3-sialyltransferase 4
Sulf1	NM_134378	10874981	0.80	-18	6.3	brwn	sulfatase 1
Ugp2	NM_001024743	10778647	1.21	284	0	grey	UDP-glucose pyrophosphorylase 2
**PROTEOLYSIS**							
Ctsk	NM_031560	10817419	0.77	-46	7.2	brwn	cathepsin K
Uba52	NM_031687	10787544	0.82	-249	0	grey	ubiquitin A-52 residue ribosomal protein fusion product 1
Ubxn11	NM_138853	10872747	0.83	-14	0.86	green	UBX domain protein 11
**RECEPTORS & BINDING PROTEINS**							
Crebl2	NM_001015027	10859249	0.82	-44	0	grey	cAMP responsive element binding protein-like 2
LOC683722	NM_001101008	10718111	0.81	-23	0	grey	similar to Fgfr1 oncogene partner
Lpar6	NM_001045843	10781441	1.49	92	0	grey	lysophosphatidic acid receptor 6
Lrp1	NM_001130490	10903082	0.55	-180	11.8	brwn	low density lipoprotein-related protein 1 (alpha-2-macroglobulin receptor)
Ltbp3	NM_001191561	10713123	0.71	-18	0	grey	latent transforming growth factor beta binding protein 3
**SIGNALING**							
Akap17a	NM_001127246	10760783	0.77	-22	0	grey	A kinase (PRKA) anchor protein 17A
Arap2	NM_001107216	10772863	1.24	29	2.4	ylw	ArfGAP with RhoGAP domain, ankyrin repeat and PH domain 2
Dtx4	NM_001047855	10729009	1.28	11	9.8	brwn	deltex homolog 4 (Drosophila)
Dusp1	NM_053769	10732652	0.81	-39	0	grey	dual specificity phosphatase 1
Gchfr	NM_133595	10838772	0.76	-16	3.9	ylw	GTP cyclohydrolase I feedback regulator
Itgbl1	NM_001017505	10782187	0.80	-33	3.4	turq	integrin, beta-like 1
Prkcd	NM_133307	10789929	0.78	-67	0	grey	protein kinase C, delta
Ptk2b	NM_017318	10784641	0.80	-14	3.1	blue	PTK2B protein tyrosine kinase 2 beta
Ptpn4	NM_001100479	10767132	1.32	83	0	grey	protein tyrosine phosphatase, non-receptor type 4
RGD1565043	ENSRNOT00000016745	10792662	0.81	-12	0	grey	similar to Rho guanine nucleotide exchange factor (GEF) 10
Rqcd1	NM_001009357	10924326	0.82	-20	1.4	turq	rcd1 (required for cell differentiation) homolog 1 (S. pombe)
Smad1	NM_013130	10806981	0.77	-27	2.2	turq	SMAD family member 1
Srp9	NM_001126095	10766378	0.83	-88	2.5	ylw	signal recognition particle 9
**TRANSCRIPTION**							
Ankrd50	NM_001191606	10822950	0.81	-22	0	grey	ankyrin repeat domain 50
Asb13	NM_001108420	10799376	0.81	-39	0	grey	ankyrin repeat and SOCS box-containing 13
Blzf1	NM_001017494	10769468	1.23	35	0	grey	basic leucine zipper nuclear factor 1
Ccdc126	NM_001109232	10855469	1.31	31	0	grey	coiled-coil domain containing 126
Ccdc80	NM_022543	10753982	0.79	-91	12.9	brwn	coiled-coil domain containing 80
Ccdc92	NM_001083898	10758208	0.82	-21	0.6	ylw	coiled-coil domain containing 92
Chchd5	NM_001106509	10839767	0.79	-53	7.9	blue	coiled-coil-helix-coiled-coil-helix domain containing 5
Dnajb1	NM_001108441	10810295	0.78	-93	0	grey	DnaJ (Hsp40) homolog, subfamily B, member 1
Fam134b	ENSRNOT00000014423	10813951	1.72	50	0	grey	family with sequence similarity 134, member B
Klhl11	NM_001105838	10747368	1.24	51	0	grey	kelch-like 11 (Drosophila)
Lhfp	NM_001109183	10815321	0.74	-31	0	grey	lipoma HMGIC fusion partner
Mageh1	NM_001013250	10937492	0.71	-20	0	grey	melanoma antigen, family H, 1
Mrm1	NM_001108832	10745751	0.72	-13	0	grey	mitochondrial rRNA methyltransferase 1 homolog (S. cerevisiae)
Nfxl1	NM_001170438	10772474	0.71	-54	4.1	blue	nuclear transcription factor, X-box binding-like 1
Os9	NM_001007265	10895826	1.37	22	0	grey	osteosarcoma amplified 9
Tsc22d3	NM_031345	10939516	0.68	-68	0	grey	TSC22 domain family, member 3
**TRANSLATION & PROTEIN MODIFICATION**							
Parp11	ENSRNOT00000027092	10859007	0.82	-13	2.2	brwn	poly (ADP-ribose) polymerase family, member 11
RGD1559951	ENSRNOT00000004303	10936841	1.54	11	0	grey	similar to 60S ribosomal protein L37a
Rngtt	NM_001107923	10868171	1.30	53	5.8	blue	RNA guanylyltransferase and 5'-phosphatase
Rps28	NM_001105730	10774372	0.82	-136	12.2	blue	ribosomal protein S28 (chr14)
Rps28	NM_001105730	10901178	0.83	-220	17.1	blue	ribosomal protein S28 (chr7)
Taf11	NM_001008350	10831892	0.73	-12	8.9	blue	TAF11 RNA polymerase II, TATA box binding protein (TBP)-associated factor
Taf13	NM_001107716	10818378	0.78	-47	4.2	ylw	TAF13 RNA polymerase II, TATA box binding protein (TBP)-associated factor
Thg1l	NM_001013966	10742236	0.78	-17	2.8	ylw	tRNA-histidine guanylyltransferase 1-like (S. cerevisiae)
**MISCELLANEOUS & UNKNOWN**							
Camta1	NM_001195560	10881917	0.82	-59	0	grey	similar to KIAA0833 protein

Fam129a	NM_022242	10764661	0.80	-24	0	grey	family with sequence similarity 129, member A
Fam175b	NM_001106307	10711723	1.21	83	23.4	blue	family with sequence similarity 175, member B
Fam55c	NM_001109435	10750825	1.20	14	0	grey	family with sequence similarity 55, member C
LOC100233176	NM_001142941	10891864	0.78	-43	1.5	ylw	hypothetical protein LOC100233176
LOC679583	ENSRNOT00000050921	10871487	0.71	-138	9.4	ylw	similar to UPF0197 protein C11orf10 homolog (chr5)
LOC686092	ENSRNOT00000050090	10923334	0.71	-157	9.9	ylw	similar to UPF0197 protein C11orf10 homolog (chr9)
Otud1	ENSRNOT00000022734	10796673	0.80	-11	0	grey	OTU domain containing 1
RGD1308059	BC091300	10869656	1.31	14	6.8	brwn	similar to DNA segment, Chr 4, Brigham & Womens Genetics 0951 expressed
RGD1310769	NM_001106749	10891297	0.79	-76	8.0	brwn	similar to HSPC288
Tmem100	NM_001017479	10737426	0.75	-13	9.6	brwn	transmembrane protein 100
Tmem80	NM_001017455	10712270	0.80	-19	0	grey	transmembrane protein 80
Tmem97	NM_001008334	10745266	0.77	-44	0	grey	transmembrane protein 97
Tsku	NM_001009965	10723822	0.77	-25	0	grey	tsukushin
**EST's**							
LOC361346	BC082051	10805100	0.80	-12	0	grey	similar to chromosome 18 open reading frame 54
RGD1303066	NM_212498	10827877	1.27	19	10.9	ylw	similar to RIKEN cDNA 2610110G12
	GENSCAN00000023940	10919226	0.68	-54	0	grey	
	GENSCAN00000023881	10780934	0.81	-14	0	grey	
	FQ230857	10769908	0.83	-44	0.4	pink	
	ENSRNOT00000069216	10841294	0.60	-21	3.0	blue	
	**ENSRNOT00000063429**	**10917318**	**0.80**	**-12**	**43.0**	**blue**	
	ENSRNOT00000061081	10860779	1.89	21	0	grey	
	ENSRNOT00000060584	10809882	0.72	-12	0	grey	
	ENSRNOT00000060432	10804017	0.70	-65	0	grey	
	ENSRNOT00000060163	10894247	0.76	-11	1.7	blue	
	ENSRNOT00000054563	10866957	0.60	-438	9.3	brwn	
	ENSRNOT00000053840	10864773	0.78	-17	4.4	ylw	(chr4)
	ENSRNOT00000053840	10892704	0.78	-17	4.4	ylw	(chr6)
	ENSRNOT00000053549	10886261	0.72	-14	0	grey	
	ENSRNOT00000053169	10728728	1.54	12	0	grey	
	**ENSRNOT00000052893**	**10830298**	**0.81**	**-10**	**48.1**	**blue**	
	**ENSRNOT00000052494**	**10713604**	**1.31**	**42**	**1.5**	**pink**	
	ENSRNOT00000046981	10932793	1.35	24	0	grey	
	ENSRNOT00000045325	10879690	0.82	-45	7.0	ylw	
	ENSRNOT00000021054	10873830	1.24	11	0	grey	
	ENSRNOT00000011389	10855599	0.80	-20	0	grey	
	ENSRNOT00000002991	10772330	0.77	-16	0.4	brwn	
	**---**	**10862453**	**1.67**	**115**	**26.9**	**blue**	
	---	10769693	0.78	-38	7.5	brwn	
	---	10840063	1.45	26	0	grey	
	---	10838324	0.82	-26	17.1	ylw	
	---	10795986	0.72	-28	4.2	ylw	
	---	10701507	1.75	47	10.4	blue	
	---	10700971	0.69	-13	6.1	blue	
	---	10700537	2.29	63	5.6	blue	
	---	10700618	1.59	13	2.1	blue	
	---	10701316	1.56	27	1.8	blue	
	---	10700630	1.73	19	7.0	brwn	
	---	10701411	1.23	24	2.6	brwn	
	---	10701142	0.66	-19	2.0	brwn	
	---	10700106	0.70	-61	0	grey	
	---	10701412	0.52	-43	0	grey	
	---	10701604	0.56	-18	0	grey	
	---	10700766	0.66	-17	0	grey	
	---	10700436	0.46	-12	0	grey	
	---	10700543	1.84	13	0	grey	
	---	10701097	1.89	14	0	grey	
	---	10700470	2.14	17	0	grey	
	---	10700282	1.77	18	0	grey	
	---	10700911	1.69	20	0	grey	
	---	10700098	1.79	21	0	grey	
	---	10701596	1.94	26	0	grey	
	---	10701472	1.53	27	0	grey	
	---	10700362	1.88	33	0	grey	
	---	10701227	1.86	37	0	grey	
	---	10700217	1.64	45	0	grey	
	---	10701194	1.42	45	0	grey	
	---	10701051	1.37	101	0	grey	
	---	10700662	1.75	24	0.4	mgnt	

	---	10700461	1.55	16	0.1	pink	
	---	10700247	1.55	51	3.9	turq	
	---	10700518	0.61	-78	4.0	ylw	
	---	10700795	0.51	-23	1.4	ylw	
	---	10700263	0.66	-79	0.7	ylw	

G. Male Kidney

Gene Symbol	RefSeq/GenBank/ Ensemble Number	Affymetrix Probset ID	V/C	Mean Diff.	k.in	Mod	Gene Title
**APOPTOSIS**							
Dapk3	NM_022546	10900296	0.77	-64	16.6	brwn	death-associated protein kinase 3
Gsdmd	NM_001130553	10897096	0.77	-13	0	grey	gasdermin D
Lsp1	NM_001025420	10712477	0.72	-46	5.7	brwn	lymphocyte-specific protein 1
Prelid1	NM_001009636	10797292	0.76	-131	16.8	turq	PRELI domain containing 1
Snn	NM_001034083	10740408	0.74	-12	0	grey	stannin
Tnfrsf12a	NM_181086	10740869	0.74	-14	0	grey	tumor necrosis factor receptor superfamily, member 12a
**CELL CYCLE**							
Bop1	NM_001024250	10904930	0.83	-20	24.2	turq	block of proliferation 1
Ccnl2	NM_001013094	10874708	1.47	113	6.1	blue	cyclin L2
Cdkl3	NM_001174131	10733401	1.50	47	0	grey	cyclin-dependent kinase-like 3
Cdkn2aipnl	NM_001008278	10733397	0.83	-72	0.6	ylw	CDKN2A interacting protein N-terminal like
Cspp1	NM_001191864	10875089	1.72	83	17.4	blue	centrosome and spindle pole associated protein 1
Ddit4	NM_080906	10832920	0.68	-32	14.2	blue	DNA-damage-inducible transcript 4
G2e3	NM_001106726	10884489	1.39	58	0	grey	G2/M-phase specific E3 ubiquitin ligase
Mcm2	NM_001107873	10863950	0.63	-34	10.9	blue	minichromosome maintenance complex component 2
Orc2l	NM_001012003	10928254	1.25	42	0	grey	origin recognition complex, subunit 2-like (yeast)
**CYTOSKELETON-ECM**							
Actr1b	NM_001039028	10927413	0.77	-46	24.3	turq	ARP1 actin-related protein 1 homolog B (yeast)
Arpc1b	NM_019289	10760196	0.76	-128	17.3	ylw	actin related protein 2/3 complex, subunit 1B
Capg	NM_001013086	10856415	0.70	-33	0	grey	capping protein (actin filament), gelsolin-like
**Ceacam6**	**NM_001103358**	**10705037**	**1.32**	**11**	**9.29**	**green**	**carcinoembryonic antigen-related cell adhesion molecule 6**
Col3a1	NM_032085	10923052	0.77	-64	13.9	brwn	collagen, type III, alpha 1
Col4a1	NM_001135009	10789594	0.82	-14	16.0	brwn	collagen, type IV, alpha 1
Coro1a	NM_130411	10725806	0.73	-28	0	grey	coronin, actin binding protein 1A
Des	NM_022531	10924507	0.70	-57	0	grey	desmin
Emd	NM_012948	10940413	0.74	-32	11.2	turq	emerin
Eml5	ENSRNOT00000057601	10891548	1.45	15	0	grey	echinoderm microtubule associated protein like 5
Esco1	BC166441	10767013	1.33	107	0	grey	establishment of cohesion 1 homolog 1 (S. cerevisiae)
Fxc1	NM_053371	10833834	0.54	-16	0	grey	fractured callus expressed transcript 1
Kif16b	NM_001107783	10850321	1.21	52	0	grey	kinesin family member 16B
Krt40	ENSRNOT00000045189	10747139	1.38	25	4.0	blue	keratin 40
Lmna	NM_001002016	10824322	0.82	-30	21.7	turq	lamin A
LOC494539	AY856054	10722165	1.55	11	2.2	blue	spectrin beta-like
Mcoln1	NM_001105903	10759698	0.83	-33	20.6	blue	mucolipin 1
Mynn	NM_001012178	10822697	1.20	21	0	grey	myoneurin
Nid67	NM_173126	10804562	0.80	-19	19.5	blue	putative small membrane protein NID67
Ninj1	NM_012867	10797705	0.74	-38	0	grey	ninjurin 1
Ninl	ENSRNOT00000068689	10850688	1.52	12	5.8	blue	ninein-like
Pfn1	NM_022511	10744494	0.82	-161	20.1	turq	profilin 1
**Plp2**	**NM_207601**	**10932534**	**0.66**	**-147**	**38.4**	**turq**	**proteolipid protein 2 (colonic epithelium-enriched)**
Shroom2	NM_001047893	10937601	1.23	22	0	grey	shroom family member 2
Sorbs2	NM_053770	10791750	1.23	39	0.76	green	sorbin and SH3 domain containing 2
Tpbg	NM_031807	10912003	0.72	-20	0	grey	trophoblast glycoprotein
Tpm2	NM_001024345	10876324	0.81	-15	0	grey	tropomyosin 2, beta
Tspan4	NM_001013070	10712349	0.73	-76	0	grey	tetraspanin 4
Tspan6	NM_001100672	10939233	1.32	180	11.1	turq	tetraspanin 6
Tubb5	NM_173102	10827906	0.78	-96	8.9	brwn	tubulin, beta 5
**Vim**	**NM_031140**	**10796445**	**0.66**	**-372**	**22.9**	**brwn**	**vimentin**
Zfc3h1	ENSRNOT00000058444	10895458	1.39	87	8.0	blue	zinc finger, C3H1-type containing
**DEVELOPMENT**							
Aamp	NM_001106920	10928890	0.80	-83	18.4	turq	angio-associated, migratory cell protein
Ahi1	NM_001002277	10702025	1.22	25	20.9	brwn	Abelson helper integration site 1
Atxn3	NM_021702	10891818	1.25	46	8.7	blue	ataxin 3
Bbs4	NM_001106826	10917954	1.29	45	0	grey	Bardet-Biedl syndrome 4
Bloc1s3	ENSRNOT00000023457	10719466	0.75	-23	16.0	blue	biogenesis of lysosomal organelles complex-1, subunit 3
Ccm2	NM_001126275	10774104	0.78	-45	14.9	blue	cerebral cavernous malformation 2
Cwh43	NM_001191677	10776547	1.36	21	2.2	brwn	cell wall biogenesis 43 C-terminal homolog (S. cerevisiae)
Diaph2	ENSRNOT00000034461	10934829	1.26	30	0	grey	diaphanous homolog 2 (Drosophila)

Dpy19l4	ENSRNOT00000037869	10875464	1.23	42	0	grey	dpy-19-like 4 (C. elegans)
**Dyt1**	**NM_153303**	**10844229**	**0.76**	**-25**	**21.5**	**brwn**	**dystonia 1**
Far1	NM_001011933	10710051	1.23	147	0	grey	fatty acyl CoA reductase 1
Fxr1	NM_001012179	10814833	1.23	345	0	grey	fragile X mental retardation, autosomal homolog 1
LOC687994	ENSRNOT00000030501	10867109	1.27	51	0.6	pink	similar to 82-kD FMRP Interacting Protein
Neu1	NM_031522	10831300	0.80	-42	0	grey	sialidase 1 (lysosomal sialidase)
Oaf	NM_001014090	10916659	0.82	-32	12.6	brwn	OAF homolog (Drosophila)
Ophn1	NM_001107848	10938550	1.27	12	0	grey	oligophrenin 1
Osgin2	ENSRNOT00000012394	10875642	1.28	15	0.1	black	oxidative stress induced growth inhibitor family member 2
Per1	NM_001034125	10734882	0.58	-43	0	grey	period homolog 1 (Drosophila)
Pkhd1	ENSRNOT00000060760	10926851	1.34	14	0	grey	polycystic kidney and hepatic disease 1
RGD1359378	NM_001007658	10897045	0.76	-21	0	grey	mesenchymal stem cell protein DSCD75
Sectm1b	NM_199082	10749762	0.73	-19	12.6	blue	secreted and transmembrane 1B
Sfrp2	NM_001100700	10816144	1.48	24	3.4	brwn	secreted frizzled-related protein 2
Spata5	NM_001108549	10815032	1.42	46	0	grey	spermatogenesis associated 5
Ssb	NM_031119	10858083	1.25	161	7.6	blue	Sjogren syndrome antigen B
Tex264	NM_001007665	10919960	0.82	-75	4.4	blue	testis expressed 264
Trpm7	AF375874	10849556	1.32	117	0	grey	transient receptor potential cation channel, subfamily M, member 7
Wbscr27	NM_001109499	10761247	0.76	-30	22.6	blue	Williams Beuren syndrome chromosome region 27 (chr12)
Wbscr27	NM_001109499	10768112	0.72	-42	16.6	blue	Williams Beuren syndrome chromosome region 27 (chr13)
**Yif1**	**NM_172017**	**10712999**	**0.82**	**-87**	**33.7**	**brwn**	**Yip1 interacting factor homolog (S. cerevisiae)**
Zfp280d	NM_001108165	10911395	1.34	59	7.6	turq	zinc finger protein 280D
**DNA REPAIR**							
Lig4	NM_001106095	10789670	1.44	74	0.7	pink	ligase IV, DNA, ATP-dependent
Pnkp	NM_001004259	10706650	0.83	-17	0	grey	polynucleotide kinase 3'-phosphatase
**ELECTRON TRANSPORT**							
Cox8a	NM_134345	10728383	0.80	-462	5.7	blue	cytochrome c oxidase subunit VIIIa
Gpx7	NM_001106673	10878635	0.83	-23	4.1	turq	glutathione peroxidase 7
**Prdx2**	**NM_017169**	**10810018**	**0.80**	**-182**	**28.4**	**brwn**	**peroxiredoxin 2**
**EPIGENETICS**							
Akap8l	NM_001013946	10900988	1.46	67	1.4	turq	A kinase (PRKA) anchor protein 8-like
Asmtl	NM_001105915	10757162	0.78	-19	24.1	blue	acetylserotonin O-methyltransferase-like
Atrx	D64059	10938897	1.24	55	3.8	blue	alpha thalassemia/mental retardation syndrome X-linked (RAD54 homolog, S. cerevisiae)
Chd6	NM_001107797	10851407	1.28	28	0	grey	chromodomain helicase DNA binding protein 6
Ddx5	NM_001007613	10748347	1.21	386	0	grey	DEAD (Asp-Glu-Ala-Asp) box polypeptide 5
Dot1l	NM_001108733	10900419	1.27	13	5.6	blue	DOT1-like, histone H3 methyltransferase (S. cerevisiae)
Ercc4	ENSRNOT00000004563	10731331	1.28	24	0	grey	excision repair cross-complementing rodent repair deficiency, complementation group 4
Ercc5	NM_001106910	10923012	1.24	28	0	grey	excision repair cross-complementing rodent repair deficiency, complementation group 5
Gadd45gip1	NM_001100504	10810059	0.77	-26	16.4	brwn	growth arrest and DNA-damage-inducible, gamma interacting protein 1
Hist1h1b	NM_001109417	10795291	0.80	-17	0	grey	histone cluster 1, H1b
Hist1h2ail	NM_001013056	10798455	0.78	-71	10.6	ylw	histone cluster 1, H2ai-like
Hist2h3c2	NM_001107698	10817535	0.79	-26	0	grey	histone cluster 2, H3c2
Kdm3a	NM_175764	10863125	1.32	78	0	grey	lysine (K)-specific demethylase 3A
Kdm4a	NM_001107966	10879110	1.22	27	7.7	turq	lysine (K)-specific demethylase 4A
Mbd3	NM_001108735	10893852	0.73	-76	0	grey	methyl-CpG binding domain protein 3
Mgea5	NM_131904	10730452	1.29	155	5.8	ylw	meningioma expressed antigen 5 (hyaluronidase)
Phf20l1	NM_001025702	10896862	1.30	70	20.0	blue	PHD finger protein 20-like 1
Setdb1	ENSRNOT00000028709	10825003	1.24	50	0	grey	SET domain, bifurcated 1
Suv420h2	NM_001107475	10718873	1.34	17	0	grey	suppressor of variegation 4-20 homolog 2 (Drosophila)
Syne1	AF452647	10717620	1.32	10	6.0	ylw	spectrin repeat containing, nuclear envelope 1
Tbpl1	NM_001127201	10702309	1.27	154	0	grey	TATA box binding protein-like 1
**GOLGI APPARATUS**							
B4galt6	NM_031740	10803394	0.82	-23	0	grey	UDP-Gal:betaGlcNAc beta 1,4-galactosyltransferase, polypeptide 6
Ap1s1	BC168682	10932646	1.23	223	0	grey	adaptor-related protein complex 1, sigma 1 subunit
Ap1s3	NM_001172150	10929267	1.27	35	0	grey	adaptor-related protein complex 1, sigma 3 subunit
**LOC685079**	**ENSRNOT00000030054**	**10842117**	**0.71**	**-122**	**24.9**	**brwn**	**similar to Protein SYS1 homolog**
Manbal	NM_001173380	10841619	0.79	-56	10.3	brwn	mannosidase, beta A, lysosomal-like
Plbd2	NM_139255	10758830	0.77	-86	17.5	turq	phospholipase B domain containing 2
**GROWTH FACTORS**							
Ccr7	NM_199489	10747024	1.45	10	0	grey	chemokine (C-C motif) receptor 7
Cxcl11	NM_182952	10771649	0.73	-13	3.8	blue	chemokine (C-X-C motif) ligand 11
Hdgfl1	NM_133549	10795072	1.43	16	1.7	red	hepatoma derived growth factor-like 1
Kitlg	NM_021843	10895152	1.30	68	6.4	turq	KIT ligand
Ogfr	NM_053340	10842839	0.81	-44	13.4	brwn	opioid growth factor receptor
**IMMUNE RESPONSE**							
C1qa	NM_001008515	10880734	0.65	-80	7.0	brwn	complement component 1, q subcomponent, A chain

C3	NM_016994	10931717	0.74	-27	1.2	brwn	complement component 3
Cfi	NM_024157	10819013	0.77	-10	9.7	brwn	complement factor I
Ctgf	NM_022266	10717233	0.75	-17	7.1	turq	connective tissue growth factor
F8a1	NM_001109323	10935873	0.75	-33	14.3	turq	coagulation factor VIII-associated (intronic transcript) 1
Ica1	NM_030844	10861005	1.36	44	0	grey	islet cell autoantigen 1
Irf3	NM_001006969	10706696	1.35	44	0.6	pink	interferon regulatory factor 3
Klra17	NM_001172088	10866098	0.68	-21	1.5	ylw	killer cell lectin-like receptor, subfamily A, member 17
RT1-DMa	NM_198741	10831628	0.81	-34	1.6	brwn	RT1 class II, locus DMa
RT1-S3	NM_001008886	10827809	0.83	-26	0	grey	RT1 class Ib, locus S3
Unc93b1	NM_001108513	10712720	0.64	-28	10.2	brwn	unc-93 homolog B1 (C. elegans)
Vsig1	NM_001037784	10935234	1.43	28	0	grey	V-set and immunoglobulin domain containing 1
**METABOLISM & TRANSPORT**							
Agpat4	NM_133406	10718001	0.77	-13	0	grey	1-acylglycerol-3-phosphate O-acyltransferase 4 (lysophosphatidic acid acyltransferase, delta)
Akr1c19	NM_001100576	10796050	1.47	24	10.1	brwn	aldo-keto reductase family 1, member C19
Alad	NM_012899	10877433	0.70	-39	15.5	brwn	aminolevulinate, delta-, dehydratase (chr5)
Alad	NM_012899	10884914	0.78	-49	26.1	turq	aminolevulinate, delta-, dehydratase (chr6)
Aldh16a1	NM_001033706	10721649	0.78	-19	5.9	brwn	aldehyde dehydrogenase 16 family, member A1
Asns	NM_013079	10860951	0.68	-58	0	grey	asparagine synthetase
Atox1	NM_053359	10742813	0.83	-39	12.7	brwn	ATX1 antioxidant protein 1 homolog (yeast)
**Atp5e**	**BC058133**	**10773636**	**0.83**	**-194**	**58.2**	**ylw**	**ATP synthase, H+ transporting, mitochondrial F1 complex, epsilon subunit**
B3gnt1	NM_001106324	10712975	0.78	-16	9.1	blue	UDP-GlcNAc:betaGal beta-1,3-N-acetylglucosaminyltransferase 1
Cacna1d	NM_017298	10789869	1.36	10	0	grey	calcium channel, voltage-dependent, L type, alpha 1D subunit
Car8	NM_001009662	10875375	1.37	34	16.0	turq	carbonic anhydrase 8
Cox11	NM_001109575	10737438	1.25	21	0	grey	COX11 homolog, cytochrome c oxidase assembly protein (yeast)
Cpox	NM_001037095	10753574	1.29	54	3.7	turq	coproporphyrinogen oxidase
Dpagt1	NM_199388	10909470	0.80	-72	24.8	turq	dolichyl-phosphate (UDP-N-acetylglucosamine) N-acetylglucosaminephosphotransferase 1 (GlcNAc-1-P transferase)
Endog	NM_001034938	10844136	1.28	25	6.6	brwn	endonuclease G
Ero1lb	NM_001191893	10799038	1.27	70	0	grey	ERO1-like beta (S. cerevisiae)
Esyt1	NM_017249	10899793	0.74	-73	7.5	brwn	extended synaptotagmin-like protein 1
Exosc4	NM_001134860	10897169	0.83	-12	15.4	blue	exosome component 4
G6pd	NM_017006	10936048	0.78	-46	0	grey	glucose-6-phosphate dehydrogenase
Gltp	NM_001134413	10759089	0.72	-35	22.3	blue	glycolipid transfer protein
Gpam	NM_017274	10730898	1.28	126	0	grey	glycerol-3-phosphate acyltransferase, mitochondrial
**Gusb**	**NM_017015**	**10757986**	**0.76**	**-18**	**33.3**	**blue**	**glucuronidase, beta**
Hexdc	NM_001142562	10740221	1.37	12	0.8	pink	hexosaminidase (glycosyl hydrolase family 20, catalytic domain) containing
Hmox2	NM_024387	10740681	1.20	20	0	grey	heme oxygenase (decycling) 2
Impdh2	NM_199099	10913186	0.81	-86	0	grey	IMP (inosine monophosphate) dehydrogenase 2
Klk1l	NM_012593	10706488	1.36	15	0	grey	kallikrein 1-like peptidase
LOC303140	DQ119106	10742657	1.30	18	0	grey	up-regulator of carnitine transporter, OCTN2
**LOC691431**	**BC167066**	**10881812**	**0.71**	**-43**	**22.2**	**brwn**	**similar to mitochondrial carrier protein MGC4399**
Lrba	NM_001108555	10816204	1.21	56	4.4	turq	LPS-responsive vesicle trafficking, beach and anchor containing
Mblac1	NM_001024996	10757247	0.73	-16	10.4	ylw	metallo-beta-lactamase domain containing 1
Mfsd3	NM_001024908	10897372	0.76	-19	12.4	turq	major facilitator superfamily domain containing 3
Mpst	NM_138843	10897446	0.80	-23	7.7	turq	mercaptopyruvate sulfurtransferase
Mri1	NM_001010947	10806698	0.81	-18	15.9	brwn	methylthioribose-1-phosphate isomerase homolog (S. cerevisiae)
Naaa	NM_001010967	10771690	0.73	-24	16.2	brwn	N-acylethanolamine acid amidase
Ndufa11	NM_212517	10931382	0.82	-136	11.8	blue	NADH dehydrogenase (ubiquinone) 1 alpha subcomplex 11
Nme1	NM_138548	10746276	0.76	-41	13.3	brwn	non-metastatic cells 1, protein (NM23A) expressed in
Nqo1	NM_017000	10810867	0.72	-16	0	grey	NAD(P)H dehydrogenase, quinone 1
Nrd1	NM_012993	10870856	1.36	280	15.9	blue	nardilysin 1 (N-arginine dibasic convertase)
Nup205	NM_001108620	10854494	1.25	18	6.6	turq	nucleoporin 205
P4ha2	NM_001108275	10733564	0.77	-27	2.5	turq	prolyl 4-hydroxylase, alpha polypeptide II
Pafah1b3	NM_053654	10719817	0.77	-24	4.8	brwn	platelet-activating factor acetylhydrolase, isoform 1b, subunit 3
Pcmtd2	NM_001107810	10843125	1.22	132	2.2	brwn	protein-L-isoaspartate (D-aspartate) O-methyltransferase domain containing 2
Pea15a	NM_001013231	10769954	0.81	-54	12.1	brwn	phosphoprotein enriched in astrocytes 15A
Plcd4	NM_080688	10924335	1.32	10	1.4	red	phospholipase C, delta 4
Por	NM_031576	10761162	0.76	-78	0	grey	P450 (cytochrome) oxidoreductase
Ptges2	NM_001107832	10835597	0.74	-32	21.1	turq	prostaglandin E synthase 2
RGD1562373	NM_001040019	10920820	0.79	-33	0	grey	similar to 3-ketoacyl-CoA thiolase B, peroxisomal precursor (Beta-ketothiolase B)
RGD1565002	ENSRNOT00000007645	10890537	0.82	-46	7.5	brwn	similar to Dehydrogenase/reductase SDR family member 7 precursor (Retinal short-chain dehydrogenase/reductase 4)
**Rhbdd2**	**NM_001191827**	**10761180**	**0.78**	**-38**	**46.4**	**turq**	**rhomboid domain containing 2**
Sat1	NM_001007667	10933716	1.31	122	4.8	ylw	spermidine/spermine N1-acetyl transferase 1
Selm	NM_001115013	10773716	0.62	-12	0	grey	selenoprotein M
Shmt2	NM_001008322	10903061	0.83	-47	7.0	turq	serine hydroxymethyltransferase 2 (mitochondrial)
**Slc16a7**	**NM_017302**	**10902868**	**1.34**	**179**	**21.5**	**brwn**	**solute carrier family 16, member 7 (monocarboxylic acid transporter 2)**
Slc17a3	NM_153622	10795218	1.20	11	0	grey	solute carrier family 17 (sodium phosphate), member 3

Slc20a2	NM_017223	10792441	0.82	-35	5.6	ylw	solute carrier family 20 (phosphate transporter), member 2
Slc22a1	NM_012697	10703045	0.78	-17	2.2	blue	solute carrier family 22 (organic cation transporter), member 1
Slc25a10	NM_133418	10740159	0.74	-61	7.5	brwn	solute carrier family 25 (mitochondrial carrier; dicarboxylate transporter), member 10
Slc25a2	NM_001106152	10804013	1.41	25	0	grey	solute carrier family 25 (mitochondrial carrier, ornithine transporter) member 2
Slc26a4	NM_019214	10889541	1.45	15	5.0	brwn	solute carrier family 26, member 4
Slc30a2	NM_001083122	10872780	0.82	-17	0	grey	solute carrier family 30 (zinc transporter), member 2
**Slc30a3**	**NM_001013243**	**10883162**	**1.40**	**15**	**2.5**	**red**	**solute carrier family 30 (zinc transporter), member 3**
Slc38a10	ENSRNOT00000006136	10749594	0.80	-36	16.6	turq	solute carrier family 38, member 10
Slc8a1	NM_019268	10888110	1.62	52	5.7	ylw	solute carrier family 8 (sodium/calcium exchanger), member 1
Sod3	NM_012880	10777108	0.74	-110	16.7	brwn	superoxide dismutase 3, extracellular
Thtpa	NM_001007682	10780278	0.80	-29	22.7	blue	thiamine triphosphatase
Timm13	NM_145781	10893680	0.77	-41	19.2	brwn	translocase of inner mitochondrial membrane 13 homolog (yeast)
Tomm6	ENSRNOT00000018959	10921444	0.80	-59	15.4	ylw	translocase of outer mitochondrial membrane 6 homolog (yeast)
Trappc6a	NM_001109410	10704849	0.75	-30	22.0	ylw	trafficking protein particle complex 6A
Tst	NM_012808	10905284	0.80	-141	0	grey	thiosulfate sulfurtransferase
Uck2	NM_001102408	10769621	0.80	-36	0	grey	uridine-cytidine kinase 2
Vat1	NM_001033683	10747633	0.70	-98	4.0	turq	vesicle amine transport protein 1 homolog (T californica)
**PROTEOLYSIS**							
Adam1a	NM_020078	10758697	1.49	20	4.85	green	a disintegrin and metallopeptidase domain 1a
Adam4l1	NM_001109277	10890962	1.74	58	6.81	green	a disintegrin and metallopeptidase domain 4-like 1
Mcpt2	ENSRNOT00000027988	10780561	1.31	12	17.4	turq	mast cell protease 2
**Mmp2**	**NM_031054**	**10809540**	**0.83**	**-33**	**22.0**	**brwn**	**matrix metallopeptidase 2**
Npepo	NM_001012346	10796991	1.37	17	0	grey	aminopeptidase O
Osgepl1	NM_001024787	10927792	1.20	11	0	grey	O-sialoglycoprotein endopeptidase-like 1
Otub1	NM_001106332	10728377	0.77	-59	16.5	blue	OTU domain, ubiquitin aldehyde binding 1
Psmc4	NM_057122	10720135	0.83	-89	11.9	blue	proteasome (prosome, macropain) 26S subunit, ATPase, 4
Psme4	NM_001025140	10774830	1.28	351	0	grey	proteasome (prosome, macropain) activator subunit 4
RGD1564425	ENSRNOT00000058238	10850081	0.78	-12	1.2	blue	similar to Proteasome subunit beta type 3 (Proteasome theta chain)
Senp7	NM_001105888	10753694	1.25	31	0	grey	SUMO1/sentrin specific peptidase 7
Spsb1	NM_001107994	10881820	0.73	-15	8.1	brwn	splA/ryanodine receptor domain and SOCS box containing 1
Tmprss2	NM_130424	10753444	1.47	28	0	grey	transmembrane protease, serine 2
Usp15	NM_145184	10902843	1.21	145	8.4	turq	ubiquitin specific peptidase 15
Usp33	NM_001191094	10819790	1.25	86	0	grey	ubiquitin specific peptidase 33
Usp40	NM_001134885	10929676	1.25	44	1.3	ylw	ubiquitin specific peptidase 40
Usp45	NM_001107918	10867862	1.25	19	10.5	brwn	ubiquitin specific peptidase 45
Wnk1	NM_053794	10865129	1.25	203	23.6	ylw	WNK lysine deficient protein kinase 1
Yme1l1	NM_053682	10796800	1.20	336	5.9	blue	YME1-like 1 (S. cerevisiae)
**RECEPTORS & BINDING PROTEINS**							
Abcg1	NM_053502	10829082	0.68	-24	20.0	brwn	ATP-binding cassette, sub-family G (WHITE), member 1
Apbb3	NM_053957	10803961	1.32	19	0	grey	amyloid beta (A4) precursor protein-binding, family B, member 3
Calb1	NM_031984	10867731	1.43	11	0	grey	calbindin 1
Fnbp4	NM_001013159	10837688	1.49	39	1.2	pink	formin binding protein 4
Fzd6	NM_001130536	10896256	1.30	26	5.0	brwn	frizzled homolog 6 (Drosophila)
Ifi30	NM_001030026	10787491	0.81	-61	0	grey	interferon gamma inducible protein 30
Ifngr1	NM_053783	10701924	0.78	-87	5.8	brwn	interferon gamma receptor 1
Lbp	NM_017208	10841693	0.67	-29	0	grey	lipopolysaccharide binding protein
Leng8	NM_001037790	10719002	1.41	14	17.7	ylw	leukocyte receptor cluster (LRC) member 8
Lifr	NM_031048	10821741	1.23	11	0	grey	leukemia inhibitory factor receptor alpha
Osbpl8	BC168899	10895366	1.26	101	9.4	blue	oxysterol binding protein-like 8
Paqr7	NM_001034081	10872808	0.72	-91	25.5	turq	progestin and adipoQ receptor family member VII
Rtp4	NM_001108321	10755088	0.70	-22	0	grey	receptor (chemosensory) transporter protein 4
**S100a6**	**NM_053485**	**10817065**	**0.60**	**-54**	**44.8**	**ylw**	**S100 calcium binding protein A6**
Tapbp	NM_033098	10831762	0.71	-61	17.1	turq	TAP binding protein
Tax1bp3	NM_001025419	10735662	0.82	-60	0	grey	Tax1 (human T-cell leukemia virus type I) binding protein 3
Tf	NM_001013110	10919637	0.43	-509	10.8	brwn	transferrin
Tfrc	NM_022712	10754785	1.39	126	11.7	brwn	transferrin receptor
Tyrobp	NM_212525	10705874	0.68	-22	0	grey	Tyro protein tyrosine kinase binding protein
Wbp4	NM_053766	10785468	0.56	-35	0	grey	WW domain binding protein 4 (formin binding protein 21)
**SIGNALING**							
Agbl3	ENSRNOT00000032077	10854463	1.34	13	0	grey	ATP/GTP binding protein-like 3
Alcam	NM_031753	10750878	1.38	113	0	grey	activated leukocyte cell adhesion molecule
Alox5ap	NM_017260	10759999	0.70	-22	0	grey	arachidonate 5-lipoxygenase activating protein
Alpk2	ENSRNOT00000042352	10804935	0.75	-10	0	grey	alpha-kinase 2
Anxa2	NM_019905	10911287	0.67	-153	13.0	brwn	annexin A2
Avpi1	NM_134373	10715452	0.82	-21	18.0	brwn	arginine vasopressin-induced 1
Cib1	NM_031145	10723032	0.82	-49	8.6	brwn	calcium and integrin binding 1 (calmyrin)
Csk	NM_001030039	10917795	0.81	-23	8.6	turq	c-src tyrosine kinase
Defb1	NM_031810	10792530	1.25	18	0	grey	defensin beta 1

Dennd4b	ENSRNOT00000049810	10816980	1.29	14	20.2	ylw	DENN/MADD domain containing 4B
Ehd2	NM_001024897	10719204	0.69	-33	14.0	brwn	EH-domain containing 2
Eid2	ENSRNOT00000026114	10705507	0.64	-33	5.1	brwn	EP300 interacting inhibitor of differentiation 2
Frmd8	NM_001008348	10728030	0.81	-16	18.0	turq	FERM domain containing 8
Garnl1	NM_020083	10890031	1.30	97	0	grey	GTPase activating Rap/RanGAP domain-like 1
Gchfr	NM_133595	10838772	0.79	-14	3.9	ylw	GTP cyclohydrolase I feedback regulator
Gna12	NM_031034	10756899	0.82	-70	0	grey	guanine nucleotide binding protein, alpha 12
**Gpr146**	**NM_001109062**	**10760663**	**0.82**	**-35**	**25.5**	**brwn**	**G protein-coupled receptor 146**
Gpr160	NM_001025147	10822663	1.38	52	0	grey	G protein-coupled receptor 160
Gpr21	NM_001109554	10835940	1.54	13	0	grey	G protein-coupled receptor 21
Grk4	NM_022928	10777639	1.31	16	4.0	ylw	G protein-coupled receptor kinase 4
Insig1	NM_022392	10861986	0.71	-278	18.9	turq	insulin induced gene 1
Lcn2	NM_130741	10844331	0.58	-50	0	grey	lipocalin 2
Map3k3	NM_001107058	10739099	0.82	-20	5.3	brwn	mitogen activated protein kinase kinase kinase 3
Map4k4	NM_001106904	10922783	0.81	-26	0	grey	mitogen-activated protein kinase kinase kinase kinase 4
Mknk2	NM_001011985	10893744	0.78	-114	13.8	turq	MAP kinase-interacting serine/threonine kinase 2
Nek6	NM_182953	10835964	0.77	-16	5.3	brwn	NIMA (never in mitosis gene a)-related kinase 6
Pdp1	NM_019372	10875532	1.21	29	14.1	turq	pyruvate dehyrogenase phosphatase catalytic subunit 1
Plekha5	AF468695	10859438	1.24	26	0	grey	pleckstrin homology domain containing, family A member 5
Plekho2	ENSRNOT00000042633	10918288	0.81	-17	8.8	brwn	pleckstrin homology domain containing, family O member 2
Prkx	NM_001033963	10938101	0.83	-21	16.0	turq	protein kinase, X-linked
Pskh1	NM_001108897	10807430	0.76	-17	15.0	turq	protein serine kinase H1
Rab23	NM_001109005	10922279	1.24	29	3.2	brwn	RAB23, member RAS oncogene family
Rab32	NM_001108902	10716785	0.81	-10	14.2	brwn	RAB32, member RAS oncogene family
Rab43	NM_001024331	10863851	0.63	-22	14.9	brwn	RAB43, member RAS oncogene family
**Rabggta**	**NM_031654**	**10783898**	**0.83**	**-19**	**47.0**	**turq**	**Rab geranylgeranyltransferase, alpha subunit**
Rabggtb	NM_138708	10827438	1.21	61	0.5	pink	Rab geranylgeranyltransferase, beta subunit
Snx14	NM_001108174	10919187	1.32	94	0	grey	sorting nexin 14
Stk36	NM_001173986	10924363	1.33	13	15.0	blue	serine/threonine kinase 36 (fused homolog, Drosophila)
Tinagl1	NM_053582	10880074	0.65	-40	4.2	brwn	tubulointerstitial nephritis antigen-like 1
**TRANSCRIPTION**							
Asb1	NM_001108232	10925443	1.36	26	0	grey	ankyrin repeat and SOCS box-containing 1
Bcl6	NM_001107084	10751931	0.52	-44	0	grey	B-cell CLL/lymphoma 6
Bcl7c	NM_001106298	10726045	0.79	-34	0	grey	B-cell CLL/lymphoma 7C
Bclaf1	NM_001047852	10701990	1.26	18	5.1	blue	BCL2-associated transcription factor 1
Bhlhb9	NM_207611	10935025	1.38	22	0	grey	basic helix-loop-helix domain containing, class B, 9
Bmyc	NM_001013163	10834360	0.76	-28	16.3	turq	brain expressed myelocytomatosis oncogene
Casc5	ENSRNOT00000067081	10838743	0.75	-16	10.5	blue	cancer susceptibility candidate 5
Ccdc17	NM_001014067	10871262	1.31	11	0.4	pink	coiled-coil domain containing 17
Ccdc62	NM_001134766	10761792	1.25	13	0	grey	coiled-coil domain containing 62
Cebpa	NM_012524	10706131	0.78	-14	7.6	brwn	CCAAT/enhancer binding protein (C/EBP), alpha
**Ddx17**	**NM_001015018**	**10897683**	**1.40**	**225**	**1.4**	**pink**	**DEAD (Asp-Glu-Ala-Asp) box polypeptide 17**
**Dnajc16**	**NM_001014194**	**10881178**	**0.83**	**-25**	**45.7**	**turq**	**DnaJ (Hsp40) homolog, subfamily C, member 16**
Dnajc30	NM_001109024	10761225	0.65	-49	5.9	turq	DnaJ (Hsp40) homolog, subfamily C, member 30
Eid3	NM_001044304	10901436	1.46	25	0	grey	EP300 interacting inhibitor of differentiation 3
Fbxo15	NM_001108436	10803025	1.36	12	16.3	turq	F-box protein 15
Fbxo6	NM_138917	10881583	0.79	-75	16.4	blue	F-box protein 6
**Hes6**	**NM_001013179**	**10929835**	**0.81**	**-19**	**22.3**	**brwn**	**hairy and enhancer of split 6 (Drosophila)**
Id1	NM_012797	10840890	0.66	-19	3.1	brwn	inhibitor of DNA binding 1
Ino80	ENSRNOT00000068584	10848539	1.23	34	4.5	brwn	INO80 homolog (S. cerevisiae)
Ino80b	NM_001048233	10863496	0.82	-11	0	grey	INO80 complex subunit B
Klhl18	ENSRNOT00000056141	10920449	0.75	-17	0	grey	kelch-like 18 (Drosophila)
LOC679823	ENSRNOT00000004434	10731732	0.83	-40	20.6	brwn	similar to 60S ribosomal protein L37a
LOC681004	ENSRNOT00000002800	10753109	1.48	77	10.1	blue	similar to GC-rich sequence DNA-binding factor homolog
Lxn	NM_031655	10823548	0.73	-18	3.29	green	latexin
Lztr1	NM_001108326	10755705	0.81	-35	11.1	brwn	leucine-zipper-like transcription regulator 1
**Mcrs1**	**NM_001013106**	**10907092**	**0.78**	**-22**	**24.7**	**brwn**	**microspherule protein 1**
**Mdm4**	**NM_001012026**	**10767663**	**1.49**	**145**	**25.1**	**blue**	**Mdm4 p53 binding protein homolog (mouse)**
Med20	NM_001013178	10926323	1.28	26	0	grey	mediator complex subunit 20
Morc3	NM_001107109	10750373	1.25	50	27.2	turq	MORC family CW-type zinc finger 3
Mtvr2	NM_001015013	10727999	0.76	-45	17.1	brwn	mammary tumor virus receptor 2
Nbl1	NM_031609	10882391	0.74	-22	4.6	brwn	neuroblastoma, suppression of tumorigenicity 1
Nfxl1	NM_001170438	10772476	1.23	42	10.4	blue	nuclear transcription factor, X-box binding-like 1
Nudt3	NM_001024243	10831868	0.81	-59	0	grey	nudix (nucleoside diphosphate linked moiety X)-type motif 3
Nudt4	NM_053598	10902003	1.21	220	0	grey	nudix (nucleoside diphosphate linked moiety X)-type motif 4
Nxf1	NM_021579	10713626	1.42	193	20.6	ylw	nuclear RNA export factor 1
Poli	NM_001106137	10805111	1.29	20	11.0	brwn	polymerase (DNA directed), iota
Polr2h	NM_001134789	10755309	1.26	43	0	grey	polymerase (RNA) II (DNA directed) polypeptide H
Polr2i	NM_001106244	10720605	0.77	-35	2.3	blue	polymerase (RNA) II (DNA directed) polypeptide I

Ppargc1b	NM_176075	10804678	0.79	-18	4.9	brwn	peroxisome proliferator-activated receptor gamma, coactivator 1 beta
Preb	NM_001170708	10883200	0.81	-62	17.0	brwn	prolactin regulatory element binding
Rb1cc1	NM_001107901	10875183	1.32	165	0	grey	RB1-inducible coiled-coil 1
RGD1306520	NM_001127485	10818230	1.36	58	10.9	turq	similar to receptor-interacting factor 1
Rictor	ENSRNOT00000029565	10813407	1.35	54	0	grey	RPTOR independent companion of MTOR, complex 2
Rras	NM_001108481	10706705	0.79	-52	0	grey	Harvey rat sarcoma virus oncogene, subgroup R
**Scand1**	**NM_001108599**	**10851192**	**0.81**	**-130**	**43.9**	**blue**	**SCAN domain-containing 1**
Son	NM_001170327	10750219	1.24	147	9.0	blue	Son DNA binding protein (chr11)
Son	NM_001170327	10875322	1.25	166	15.6	blue	Son DNA binding protein (chr5)
Stx16	NM_001108610	10842635	1.30	38	14.5	turq	syntaxin 16
Tada2b	NM_001170455	10773358	0.81	-30	28.4	turq	transcriptional adaptor 2 (ADA2 homolog, yeast)-beta
Thoc1	NM_001047850	10800098	1.26	56	4.2	turq	THO complex 1
Tsc22d3	NM_031345	10939516	0.65	-75	0	grey	TSC22 domain family, member 3
Zbed4	NM_001134800	10898454	0.80	-20	0	grey	zinc finger, BED-type containing 4
Zcchc2	BC100090	10763284	1.25	45	0	grey	zinc finger, CCHC domain containing 2
Zfp187	ENSRNOT00000024693	10795319	1.39	40	2.6	blue	zinc finger protein 187
Zfp317	NM_001134634	10908182	1.29	29	0	grey	zinc finger protein 317
Zfp36	NM_133290	10720215	0.63	-63	10.7	turq	zinc finger protein 36
Zfp385d	NM_001013992	10782437	1.24	13	8.7	brwn	zinc finger protein 385D
Zfp451	NM_001033705	10927233	1.27	33	0	grey	zinc finger protein 451
Zmym4	NM_001107982	10879879	1.26	43	19.8	ylw	zinc finger, MYM-type 4
Zmym6	NM_001108681	10872114	1.29	32	0.6	ylw	zinc finger, MYM-type 6
Znf292	NM_001008879	10875941	1.33	75	1.5	blue	zinc finger protein 292
Zranb2	NM_031616	10819890	1.34	121	1.3	pink	zinc finger, RAN-binding domain containing 2
Zswim5	NM_001107963	10871339	0.82	-18	0	grey	zinc finger, SWIM domain containing 5
**TRANSLATION & PROTEIN MODIFICATION**							
Cpsf7	NM_001014245	10713894	1.29	19	0	grey	cleavage and polyadenylation specific factor 7, 59kDa
Eef1g	NM_001004223	10713789	0.78	-228	19.2	blue	eukaryotic translation elongation factor 1 gamma
Eif1ad	NM_001008305	10713033	0.82	-19	5.9	brwn	eukaryotic translation initiation factor 1A domain containing
Eif3g	NM_001013095	10915427	0.81	-81	16.7	brwn	eukaryotic translation initiation factor 3, subunit G
Eif3h	NM_198751	10903684	0.77	-71	10.9	blue	eukaryotic translation initiation factor 3, subunit H
Eif6	NM_001037352	10778386	0.80	-45	8.5	turq	eukaryotic translation initiation factor 6
Hnrph1	NM_080896	10733243	1.28	271	0.8	pink	heterogeneous nuclear ribonucleoprotein H1
Lsm7	NM_001108732	10893684	0.82	-10	2.9	blue	LSM7 homolog, U6 small nuclear RNA associated (S. cerevisiae)
Luc7l2	NM_001107853	10854626	1.25	151	3.0	blue	LUC7-like 2 (S. cerevisiae)
Mrpl23	NM_022529	10712501	0.79	-97	10.8	blue	mitochondrial ribosomal protein L23
Mrpl34	NM_001006965	10787281	0.83	-21	16.4	blue	mitochondrial ribosomal protein L34
Mrpl38	NM_001009369	10749132	0.73	-41	0	grey	mitochondrial ribosomal protein L38
Mrto4	NM_001106697	10880913	0.79	-29	15.9	brwn	mRNA turnover 4 homolog (S. cerevisiae)
Parp3	NM_001008328	10919928	0.81	-25	0	grey	poly (ADP-ribose) polymerase family, member 3
Pcbp3	NM_001011945	10829418	1.32	28	6.0	ylw	poly(rC) binding protein 3
Pnn	NM_001109023	10884740	1.27	103	17.3	blue	pinin, desmosome associated protein
Polr1b	NM_031773	10839751	0.83	-16	6.0	turq	polymerase (RNA) I polypeptide B
Polr1c	NM_001008330	10921741	0.83	-13	16.6	blue	polymerase (RNA) I polypeptide C
Pop7	NM_001105918	10760952	0.77	-28	20.6	brwn	processing of precursor 7, ribonuclease P/MRP subunit (S. cerevisiae)
Prpf4b	NM_001011923	10798074	1.41	84	1.2	pink	PRP4 pre-mRNA processing factor 4 homolog B (yeast)
Rbm25	NM_001108984	10731611	1.28	170	10.6	blue	RNA binding motif protein 25
**Rbm39**	**NM_001013207**	**10851173**	**1.27**	**330**	**1.4**	**pink**	**RNA binding motif protein 39**
RGD1561841	ENSRNOT00000042248	10823044	1.46	10	0	grey	similar to ribosomal protein L31
Rnh1	NM_139105	10726726	0.82	-39	20.6	blue	ribonuclease/angiogenin inhibitor 1
Rnmt	NM_001008299	10805052	1.21	43	3.7	brwn	RNA (guanine-7-) methyltransferase
Rpl13	NM_031101	10733688	0.82	-166	39.7	ylw	ribosomal protein L13 (chr10)
Rpl13	NM_031101	10777032	0.81	-236	38.0	ylw	ribosomal protein L13 (chr14)
Rpl13	NM_031101	10808595	0.79	-243	38.6	ylw	ribosomal protein L13 (chr19)
Rpl13	NM_031101	10914849	0.82	-179	37.7	ylw	ribosomal protein L13 (chr8)
Rpl13	ENSRNOT00000020635	10883459	0.82	-265	32.9	ylw	ribosomal protein L13 (chr6)
**Rpl7a**	**NM_001114391**	**10753737**	**1.78**	**53**	**28.9**	**blue**	**ribosomal protein L7a (chr11)**
**Rpl7a**	**NM_001114391**	**10797321**	**1.63**	**43**	**32.1**	**blue**	**ribosomal protein L7a (chr17)**
Rps12	NM_031709	10702286	0.82	-92	22.7	ylw	ribosomal protein S12 (chr1)
Rps12	NM_031709	10807514	0.80	-158	19.1	ylw	ribosomal protein S12 (chr19)
Rps12	NM_031709	10839876	0.79	-209	16.7	ylw	ribosomal protein S12 (chr3)
Rps15	NM_017151	10900533	0.77	-142	37.5	ylw	ribosomal protein S15
**Rps21**	**NM_031111**	**10842805**	**0.83**	**-231**	**55.9**	**ylw**	**ribosomal protein S21 (chr3)**
**Rps21**	**NM_031111**	**10938962**	**0.82**	**-194**	**49.4**	**ylw**	**ribosomal protein S21 (chrX)**
Sf3b5	NM_001126092	10701797	0.81	-30	11.9	ylw	splicing factor 3b, subunit 5
Sfrs18	NM_001025274	10867882	1.63	109	18.7	blue	splicing factor, arginine/serine-rich 18
Sfrs5	NM_001195505	10885672	1.56	445	1.2	pink	splicing factor, arginine/serine-rich 5
Sfrs6	NM_001014185	10841912	1.26	86	0	grey	splicing factor, arginine/serine-rich 6
Snrnp70	NM_001108483	10721802	1.26	99	1.1	pink	small nuclear ribonucleoprotein 70 (U1)

Srrm2	ENSRNOT00000006370	10731980	1.32	131	6.4	ylw	serine/arginine repetitive matrix 2
Yars	NM_001025696	10872291	0.79	-75	21.3	turq	tyrosyl-tRNA synthetase
**MISCELLANEOUS & UNKNOWN**							
Cuedc2	NM_001079886	10730559	0.82	-12	3.4	brwn	CUE domain containing 2
Fam108a1	NM_001006983	10893768	0.76	-38	11.2	blue	family with sequence similarity 108, member A1
Fam129a	NM_022242	10764661	0.50	-80	0	grey	family with sequence similarity 129, member A
Fam164c	NM_001014079	10886023	0.79	-16	0	grey	family with sequence similarity 164, member C
Fam175b	NM_001106307	10711723	1.21	86	23.4	blue	family with sequence similarity 175, member B
Fam46a	NM_001106844	10919041	0.77	-39	11.2	blue	family with sequence similarity 46, member A
Fam48a	NM_001014170	10815393	1.43	124	21.0	blue	family with sequence similarity 48, member A
LOC100359595	ENSRNOT00000001051	10833422	1.28	25	5.1	turq	hypothetical protein LOC100359595
LOC100360120	ENSRNOT00000055732	10859203	0.77	-82	0	grey	hypothetical protein LOC100360120
LOC100360664	ENSRNOT00000024010	10926433	0.78	-22	14.2	blue	rCG43434-like
LOC100362431	ENSRNOT00000013565	10846299	0.83	-12	3.3	blue	tetratricopeptide repeat domain 30B
LOC100364734	ENSRNOT00000017292	10747177	1.24	15	0	grey	hypothetical protein LOC100364734
LOC290577	ENSRNOT00000060770	10786995	1.22	136	14.0	blue	hypothetical LOC290577
LOC310721	BC083570	10825403	1.44	125	3.4	blue	similar to 4930431B09Rik protein
LOC366763	ENSRNOT00000063789	10892616	0.64	-13	9.2	blue	rCG63169-like
LOC500300	NM_001024334	10858160	0.71	-18	0	grey	similar to hypothetical protein MGC6835
LOC500392	NM_001024340	10875102	1.32	12	0	grey	similar to hypothetical protein FLJ25692
LOC685634	ENSRNOT00000019224	10872155	0.72	-39	11.6	brwn	hypothetical protein LOC685634
**LOC688452**	**FM212919**	**10703432**	**1.74**	**39**	**21.0**	**brwn**	**hypothetical protein LOC688452**
LOC688786	NM_001195245	10759636	0.79	-61	6.0	ylw	similar to CG14483-PA
**MGC93975**	**BC079041**	**10721350**	**0.74**	**-121**	**42.4**	**turq**	**similar to 2310044H10Rik protein**
Oxr1	NM_001197907	10896353	1.23	185	0	grey	oxidation resistance 1
RGD1304728	ENSRNOT00000018808	10744663	1.25	17	0	grey	similar to 4933427D14Rik protein
RGD1305689	NM_001008297	10789740	0.79	-84	0	grey	similar to DNA segment, Chr 14, ERATO Doi 449, expressed
RGD1307067	BC161971	10893974	0.81	-34	19.4	turq	LOC362840
RGD1308059	BC091300	10869656	0.72	-18	6.8	brwn	similar to DNA segment, Chr 4, Brigham & Womens Genetics 0951 expressed
RGD1308297	BC082039	10785745	1.31	80	0	grey	similar to CG10084-PA
RGD1311899	BC088751	10762735	0.82	-12	0	grey	similar to RIKEN cDNA 2210016L21 gene
RGD1560909	BC158750	10922771	0.78	-41	23.9	turq	similar to DNA segment, Chr 1, Brigham & Womens Genetics 0212 expressed
RGD1561878	ENSRNOT00000045241	10841033	1.25	28	5.6	blue	similar to mKIAA0978 protein
RGD1562079	NM_001177687	10720557	0.76	-26	7.9	blue	RGD1562079
RGD1565059	NM_001127562	10823456	1.31	16	0	grey	similar to hypothetical protein E130311K13
RGD1565192	NM_001134639	10777034	0.74	-13	16.3	blue	similar to 1810013D10Rik protein
Sdccag1	NM_001170586	10719956	1.39	80	2.9	blue	serologically defined colon cancer antigen 1
Tdrd3	NM_001012043	10781752	1.25	54	18.0	turq	tudor domain containing 3
**Tmem134**	**NM_001078647**	**10712816**	**0.79**	**-152**	**29.7**	**blue**	**transmembrane protein 134**
**Tmem147**	**NM_001038494**	**10720767**	**0.77**	**-48**	**28.1**	**brwn**	**transmembrane protein 147**
Tmem160	NM_001106227	10704482	0.83	-40	19.0	blue	transmembrane protein 160
Tmem161b	ENSRNOT00000050072	10820232	1.26	132	5.4	brwn	transmembrane protein 161B
Tmem176a	NM_001039008	10855416	0.69	-100	2.7	brwn	transmembrane protein 176A
Tmem20	NM_001191635	10715149	1.22	12	0	grey	transmembrane protein 20
Tmem223	NM_001191104	10713648	0.81	-53	26.6	ylw	transmembrane protein 223
Tmem38b	NM_001014191	10869151	0.78	-66	4.6	blue	transmembrane protein 38B
Tmem86b	NM_001109604	10704014	1.46	44	5.52	green	transmembrane protein 86B
Tmem88	NM_001128155	10743961	0.75	-14	4.1	brwn	transmembrane protein 88
Tmtc3	NM_001135858	10902024	1.24	70	13.9	blue	transmembrane and tetratricopeptide repeat containing 3
**EST's**							
LOC691286	NM_001109634	10742913	1.33	13	4.84	green	similar to RIKEN cDNA 4930504O13
MGC72612	NM_001009538	10787710	1.21	28	10.1	turq	similar to expressed sequence AI449175
Mir218-1	NR_031931	10777176	2.46	14	0	grey	microRNA mir-218-1
Mir339	NR_031784	10757098	1.47	22	2.4	blue	microRNA mir-339
Mir341	rno-mir-341	10886842	1.47	18	0	grey	rno-mir-341
Mir346	NR_031790	10787029	1.30	20	9.5	blue	microRNA mir-346
Mir598	NR_032286	10781012	1.34	33	0	grey	microRNA mir-598
Mir667	NR_032764	10887072	1.25	18	0	grey	microRNA mir-667
RGD1304587	NM_001100551	10733958	0.81	-34	1.9	blue	similar to RIKEN cDNA 2310033P09
RGD1305587	BC158618	10833144	0.78	-75	16.8	brwn	similar to RIKEN cDNA 2010107G23
RGD1306058	NM_001134572	10794353	0.80	-18	4.4	ylw	similar to RIKEN cDNA 1110007C09
RGD1307934	ENSRNOT00000031133	10730287	1.33	34	14.7	brwn	similar to DNA segment, Chr 19, ERATO Doi 386, expressed
RGD1310553	NM_001008517	10922951	1.44	33	0	grey	similar to expressed sequence AI597479
RGD1311078	BC168942	10739781	0.78	-20	3.7	brwn	LOC360664
**RGD1311660**	**NM_001163320**	**10760677**	**0.78**	**-77**	**39.0**	**brwn**	**similar to RIKEN cDNA 1110007L15**
**RGD1359127**	**ENSRNOT00000021939**	**10893896**	**0.81**	**-11**	**31.3**	**brwn**	**similar to RIKEN cDNA 2310011J03**
RGD1560258	NM_001134570	10761380	1.35	48	2.68	green	similar to RIKEN cDNA 4930579G22
RGD1561557	NM_001108929	10829351	1.29	14	24.4	blue	similar to chromosome 21 open reading frame 29
RGD1564067	NM_001166303	10830906	1.33	16	0	grey	similar to RIKEN cDNA 1700022C21

RGD1565616	NM_001109206	10849967	1.27	54	17.3	brwn	RGD1565616
	Y09171	10780173	0.74	-118	7.1	ylw	
	U15425	10701862	0.69	-23	0	grey	
	NC_001665	10930560	1.36	549	13.8	turq	
	J05014	10876313	0.76	-546	18.2	ylw	
	GENSCAN00000039363	10727226	1.34	20	0	grey	
	GENSCAN00000037350	10701986	1.61	16	0	grey	
	GENSCAN00000035572	10859857	1.69	29	11.9	blue	
	GENSCAN00000021698	10830199	1.55	11	0	grey	
	GENSCAN00000021509	10878967	0.72	-31	18.8	ylw	
	GENSCAN00000006482	10934827	1.95	16	24.5	blue	
	**FQ231323**	**10931346**	**0.80**	**-66**	**37.2**	**brwn**	
	FQ231232	10881468	1.38	114	0	grey	
	FQ230857	10769908	0.69	-89	0.4	pink	
	FQ230643	10930984	1.22	18	0	grey	
	**FQ229993**	**10737194**	**0.69**	**-170**	**44.9**	**ylw**	
	**FQ228798**	**10931010**	**0.81**	**-114**	**23.9**	**brwn**	
	FQ227960	10890297	1.46	10	0	grey	
	FQ224565	10931577	1.28	68	0	grey	
	FQ223177	10926091	1.32	144	0	grey	
	FQ218314	10892515	0.72	-11	10.9	blue	
	FQ213782	10866535	0.76	-12	0	grey	
	FQ211569	10787706	1.35	87	10.5	blue	
	ENSRNOT00000070527	10771730	1.47	12	0	grey	
	ENSRNOT00000070494	10852413	0.71	-10	18.4	blue	
	**ENSRNOT00000070461**	**10837686**	**0.72**	**-10**	**52.5**	**blue**	
	ENSRNOT00000070202	10834604	0.58	-36	18.4	ylw	
	**ENSRNOT00000070005**	**10855976**	**0.70**	**-12**	**45.4**	**blue**	
	ENSRNOT00000069930	10714632	1.98	10	0	grey	
	ENSRNOT00000069734	10911811	0.61	-23	4.0	ylw	
	**ENSRNOT00000069375**	**10750874**	**0.69**	**-19**	**50.4**	**blue**	
	ENSRNOT00000069295	10739864	0.71	-13	22.9	ylw	
	**ENSRNOT00000068983**	**10855978**	**0.74**	**-13**	**47.7**	**blue**	
	ENSRNOT00000067869	10850407	1.28	11	2.6	ylw	
	ENSRNOT00000067296	10805514	1.25	27	0	grey	
	ENSRNOT00000065149	10804301	1.24	37	5.5	brwn	
	ENSRNOT00000064284	10748861	0.72	-19	12.9	turq	
	ENSRNOT00000063746	10800599	0.53	-16	6.4	blue	
	ENSRNOT00000062069	10852719	1.73	13	0.8	blue	
	ENSRNOT00000060862	10782898	1.69	118	12.6	blue	
	ENSRNOT00000060466	10801260	1.69	13	17.0	blue	
	ENSRNOT00000060259	10876463	1.38	15	2.6	blue	
	ENSRNOT00000060124	10932701	0.74	-48	2.7	blue	
	**ENSRNOT00000058123**	**10902762**	**0.74**	**-18**	**28.9**	**blue**	
	ENSRNOT00000057159	10766878	1.65	28	2.4	blue	
	ENSRNOT00000056869	10778718	0.80	-44	10.0	ylw	
	ENSRNOT00000055639	10866603	0.70	-11	16.8	blue	
	ENSRNOT00000054649	10930226	0.67	-188	6.7	ylw	
	ENSRNOT00000054624	10905605	0.80	-46	2.8	ylw	
	**ENSRNOT00000054574**	**10873878**	**0.73**	**-11**	**51.9**	**blue**	
	ENSRNOT00000054438	10745134	0.76	-15	8.7	ylw	
	**ENSRNOT00000054288**	**10717396**	**0.73**	**-13**	**43.1**	**blue**	
	ENSRNOT00000054178	10914316	0.73	-22	30.2	ylw	
	ENSRNOT00000054058	10910768	1.44	11	0	grey	
	ENSRNOT00000054050	10827238	0.53	-18	16.4	blue	
	ENSRNOT00000054014	10722427	0.76	-12	2.5	black	
	ENSRNOT00000053957	10829357	0.63	-27	14.1	blue	
	ENSRNOT00000053950	10749818	1.46	33	0	grey	
	**ENSRNOT00000053512**	**10859302**	**0.72**	**-12**	**26.3**	**blue**	
	ENSRNOT00000053504	10717139	1.66	18	0	grey	
	ENSRNOT00000053479	10851380	0.80	-12	39.9	ylw	
	ENSRNOT00000053447	10765495	0.67	-29	42.8	ylw	
	ENSRNOT00000053432	10856795	1.49	11	1.3	blue	
	**ENSRNOT00000053366**	**10752096**	**0.79**	**-11**	**55.0**	**blue**	
	ENSRNOT00000053274	10754642	1.45	20	10.5	blue	
	ENSRNOT00000053196	10829163	1.32	12	0.5	pink	
	ENSRNOT00000053174	10851382	0.73	-31	43.1	ylw	
	ENSRNOT00000053169	10728728	1.60	13	0	grey	
	**ENSRNOT00000052993**	**10722475**	**0.73**	**-11**	**41.9**	**blue**	

	**ENSRNOT00000052947**	**10826602**	**0.70**	**-71**	**56.8**	**ylw**	
	**ENSRNOT00000052862**	**10801341**	**0.65**	**-12**	**29.1**	**blue**	
	ENSRNOT00000052679	10903965	0.71	-20	3.5	blue	
	ENSRNOT00000052678	10731193	1.90	56	0	grey	
	ENSRNOT00000052610	10910770	1.57	27	4.0	blue	
	**ENSRNOT00000052494**	**10713604**	**1.79**	**90**	**1.5**	**pink**	
	**ENSRNOT00000052431**	**10750458**	**0.69**	**-12**	**60.4**	**blue**	
	ENSRNOT00000051903	10891010	0.65	-16	22.5	blue	
	ENSRNOT00000051851	10939758	0.75	-11	6.0	blue	
	ENSRNOT00000051223	10791274	1.50	18	1.8	blue	
	ENSRNOT00000050121	10940063	1.39	10	4.1	blue	
	ENSRNOT00000049673	10821820	0.76	-11	9.5	ylw	
	ENSRNOT00000048840	10747153	1.41	19	20.6	blue	
	ENSRNOT00000047997	10855525	0.73	-11	35.5	ylw	
	ENSRNOT00000046525	10793427	1.30	18	0	grey	
	ENSRNOT00000046248	10860184	1.50	18	0	grey	
	ENSRNOT00000046129	10798473	1.43	12	0	grey	
	ENSRNOT00000045189	10747141	1.38	25	4.0	blue	
	ENSRNOT00000044597	10797009	1.31	236	0	grey	
	ENSRNOT00000043970	10934560	0.68	-13	17.2	blue	
	ENSRNOT00000043059	10793600	1.33	232	0	grey	
	ENSRNOT00000042358	10779954	1.36	17	0	grey	
	ENSRNOT00000042123	10873790	0.78	-103	0	grey	
	ENSRNOT00000041305	10744937	0.68	-43	6.9	blue	
	ENSRNOT00000041132	10890648	1.22	16	0	grey	
	ENSRNOT00000039420	10911476	1.74	28	16.8	blue	
	ENSRNOT00000039410	10844916	1.32	38	2.6	turq	
	ENSRNOT00000038922	10751923	1.63	16	5.7	blue	
	ENSRNOT00000038531	10774930	0.79	-86	31.7	ylw	
	**ENSRNOT00000036634**	**10810836**	**0.82**	**-23**	**23.0**	**brwn**	
	ENSRNOT00000036104	10805524	1.85	23	0.7	pink	
	ENSRNOT00000035374	10850519	0.79	-17	0	grey	
	ENSRNOT00000035247	10755897	1.28	38	0	grey	
	ENSRNOT00000034446	10787698	1.25	16	0	grey	
	ENSRNOT00000033610	10790346	0.79	-25	9.3	ylw	
	ENSRNOT00000030293	10718408	1.24	24	9.0	ylw	
	ENSRNOT00000030148	10840394	0.83	-20	0	grey	
	ENSRNOT00000029150	10751919	1.63	13	0	grey	
	ENSRNOT00000028670	10728193	0.77	-47	7.7	turq	
	ENSRNOT00000025383	10920986	1.49	13	3.9	blue	
	ENSRNOT00000020883	10823252	1.39	73	0	grey	
	ENSRNOT00000017653	10835777	0.79	-15	8.8	ylw	
	ENSRNOT00000017383	10782229	1.20	26	0	grey	
	ENSRNOT00000010681	10869772	1.20	78	3.4	blue	
	ENSRNOT00000008623	10895391	1.43	67	4.4	turq	
	ENSRNOT00000005478	10732657	0.76	-11	1.9	brwn	
	ENSRNOT00000003373	10733067	1.27	40	8.4	brwn	
	ENSRNOT00000003180	10737422	0.81	-13	3.0	ylw	
	ENSRNOT00000003115	10731326	1.49	13	2.7	ylw	
	BC090353	10728028	1.60	960	11.2	blue	
	**---**	**10818987**	**0.68**	**-15**	**63.1**	**blue**	
	**---**	**10930405**	**0.73**	**-10**	**53.5**	**blue**	
	**---**	**10817191**	**0.66**	**-14**	**42.0**	**blue**	
	**---**	**10862453**	**1.90**	**144**	**26.9**	**blue**	
	---	10851019	1.80	25	20.9	blue	
	---	10902623	1.54	74	18.6	blue	
	---	10937568	0.60	-22	18.2	blue	
	---	10869724	0.69	-11	16.4	blue	
	---	10792331	1.43	36	8.6	blue	
	---	10918865	1.43	36	8.6	blue	
	---	10749886	1.62	10	8.5	blue	
	---	10865163	2.43	45	7.2	blue	
	---	10708587	1.59	25	5.0	blue	
	---	10714204	1.47	270	4.2	blue	
	---	10807793	1.33	177	4.1	blue	
	---	10911847	1.33	20	2.3	blue	
	---	10921268	1.41	72	2.1	blue	
	---	10921095	0.77	-23	18.5	brwn	
	---	10800214	1.79	15	0	grey	

	---	10922266	2.04	15	0	grey	
	---	10933576	1.32	18	0	grey	
	---	10916853	1.44	10	0.8	pink	
	---	10811898	1.36	198	14.7	turq	
	---	10750452	1.23	15	1.9	ylw	
	**---**	**10700903**	**0.77**	**-272**	**35.2**	**blue**	
	**---**	**10700749**	**1.64**	**69**	**34.1**	**blue**	
	**---**	**10700398**	**1.70**	**313**	**33.8**	**blue**	
	---	10700714	1.70	186	23.6	blue	
	---	10700889	0.57	-117	20.2	blue	
	---	10700208	2.38	109	19.9	blue	
	---	10701605	1.63	414	18.4	blue	
	---	10700525	1.32	632	17.1	blue	
	---	10700873	1.45	1046	16.2	blue	
	---	10700831	2.26	91	15.2	blue	
	---	10700124	1.34	197	14.3	blue	
	---	10701140	2.34	103	13.2	blue	
	---	10701572	1.78	40	11.9	blue	
	---	10700423	1.43	31	11.1	blue	
	---	10700968	0.52	-24	9.9	blue	
	---	10700473	2.15	146	8.5	blue	
	---	10700943	0.71	-32	7.8	blue	
	---	10700172	2.04	15	7.0	blue	
	---	10701595	1.90	24	6.7	blue	
	---	10700817	0.69	-12	6.2	blue	
	---	10700464	1.98	40	6.0	blue	
	---	10700366	1.35	247	6.0	blue	
	---	10701302	1.97	68	5.8	blue	
	---	10701243	1.77	45	5.8	blue	
	---	10700735	1.62	67	5.7	blue	
	---	10700537	1.79	43	5.6	blue	
	---	10701029	1.50	47	5.3	blue	
	---	10700654	3.70	48	5.1	blue	
	---	10700826	2.77	36	5.0	blue	
	---	10700112	2.12	35	3.9	blue	
	---	10701252	1.39	19	3.6	blue	
	---	10700080	2.12	25	3.0	blue	
	---	10700725	1.58	10	2.6	blue	
	---	10700343	2.32	26	1.9	blue	
	---	10700855	2.01	32	1.9	blue	
	---	10701300	0.55	-16	1.7	blue	
	---	10700666	2.03	11	1.6	blue	
	---	10700535	1.88	30	1.3	blue	
	---	10700726	0.65	-13	1.1	blue	
	**---**	**10700861**	**0.64**	**-100**	**24.8**	**brwn**	
	---	10701094	0.74	-122	14.8	brwn	
	---	10700571	0.80	-932	13.0	brwn	
	---	10700462	0.83	-321	11.6	brwn	
	---	10700372	0.72	-92	10.4	brwn	
	---	10701413	1.31	190	9.1	brwn	
	---	10701033	1.51	17	2.8	brwn	
	---	10701333	1.25	508	1.8	brwn	
	---	10701440	0.82	-14	1.3	brwn	
	---	10701517	0.76	-165	0	grey	
	---	10701505	0.68	-44	0	grey	
	---	10700614	0.72	-13	0	grey	
	---	10700835	0.78	-11	0	grey	
	---	10700763	0.70	-10	0	grey	
	---	10701237	0.66	-10	0	grey	
	---	10700587	1.30	12	0	grey	
	---	10701117	1.32	12	0	grey	
	---	10701236	1.73	13	0	grey	
	---	10700638	1.39	13	0	grey	
	---	10700353	1.52	14	0	grey	
	---	10700098	1.46	14	0	grey	
	---	10700363	1.83	14	0	grey	
	---	10700650	1.54	14	0	grey	
	---	10700551	1.68	15	0	grey	
	---	10700489	1.77	18	0	grey	

	---	10700282	1.89	20	0	grey	
	---	10701045	1.74	20	0	grey	
	---	10701169	2.10	20	0	grey	
	---	10701268	1.82	21	0	grey	
	---	10700221	1.58	22	0	grey	
	---	10700543	2.84	23	0	grey	
	---	10700715	1.72	23	0	grey	
	---	10700424	1.42	24	0	grey	
	---	10701478	1.72	25	0	grey	
	---	10700541	1.54	26	0	grey	
	---	10701619	2.29	27	0	grey	
	---	10700893	1.30	27	0	grey	
	---	10701489	1.84	27	0	grey	
	---	10700508	2.10	29	0	grey	
	---	10701432	2.03	31	0	grey	
	---	10701156	2.14	34	0	grey	
	---	10701161	2.12	36	0	grey	
	---	10700766	2.41	37	0	grey	
	---	10700657	1.89	37	0	grey	
	---	10701291	2.27	37	0	grey	
	---	10700189	1.60	38	0	grey	
	---	10701030	1.48	42	0	grey	
	---	10700984	1.76	44	0	grey	
	---	10700213	2.63	45	0	grey	
	---	10700400	1.34	47	0	grey	
	---	10700228	1.84	57	0	grey	
	---	10701506	2.19	59	0	grey	
	---	10701121	1.69	75	0	grey	
	---	10700847	2.96	78	0	grey	
	---	10700290	1.87	93	0	grey	
	---	10701051	1.44	115	0	grey	
	---	10701541	1.32	216	0	grey	
	---	10701009	1.51	70	0.4	pink	
	---	10701359	2.11	31	0.4	pink	
	---	10701568	0.56	-70	22.0	turq	
	---	10700815	0.70	-57	17.8	turq	
	---	10700451	0.68	-74	13.8	turq	
	---	10701022	1.88	11	7.2	turq	
	---	10700637	0.64	-79	5.9	turq	
	---	10700950	1.98	37	2.4	turq	
	---	10701516	2.65	71	2.2	turq	
	---	10700925	1.89	54	1.4	turq	
	---	10701067	1.70	23	3.8	ylw	
	---	10701113	1.32	12	3.6	ylw	
	---	10701279	1.28	129	1.3	ylw	
	---	10700770	1.96	23	0.5	ylw	

H. Male Liver

Gene Symbol	RefSeq/GenBank/ Ensemble Number	Affymetrix Probset ID	V/C	Mean Diff.	k.in	Mod	Gene Title
**APOPTOSIS**							
Lsp1	NM_001025420	10712477	1.23	29	5.7	brwn	lymphocyte-specific protein 1
**CELL CYCLE**							
Bop1	NM_001024250	10904930	0.82	-21	24.2	turq	block of proliferation 1
Clock	NM_021856	10772249	1.36	91	2.4	brwn	clock homolog (mouse)
Osgin1	NM_138504	10808302	1.64	45	0	grey	oxidative stress induced growth inhibitor 1
**CYTOSKELETON-ECM**							
Clec4a1	NM_001005890	10858553	1.45	56	12.1	turq	C-type lectin domain family 4, member a1
Enc1	NM_001003401	10812689	0.50	-77	0	grey	ectodermal-neural cortex 1
Fgl2	NM_053455	10860231	1.40	68	6.1	turq	fibrinogen-like 2
Flna	NM_001134599	10936086	1.27	60	4.5	brwn	filamin A, alpha
Gpnmb	NM_133298	10855449	1.42	22	7.0	turq	glycoprotein (transmembrane) nmb
Jam3	NM_001004269	10915910	0.79	-28	1.0	ylw	junctional adhesion molecule 3
Lgals3	NM_031832	10779673	1.31	40	0	grey	lectin, galactoside-binding, soluble, 3
Lmnb1	NM_053905	10801821	1.22	13	0	grey	lamin B1
Msn	NM_030863	10934056	1.41	104	9.1	brwn	moesin
Nrcam	NM_013150	10884430	0.66	-13	13.0	brwn	neuronal cell adhesion molecule
Pkp2	NM_001100499	10752576	0.82	-31	0	grey	plakophilin 2

Sdc4	NM_012649	10851599	0.80	-170	0	grey	syndecan 4
Stom	NM_001011965	10844666	1.38	90	0	grey	stomatin
Tagln2	NM_001013127	10765772	1.43	93	0	grey	transgelin 2
Tspan13	NM_001013244	10889675	1.26	126	6.4	turq	tetraspanin 13
Tspan18	NM_001107750	10847562	1.37	32	13.3	brwn	tetraspanin 18
**DEVELOPMENT**							
Atxn7l1	ENSRNOT00000014054	10884189	1.33	31	12.1	brwn	ataxin 7-like 1
Bank1	NM_001047918	10826969	1.42	12	1.1	turq	B-cell scaffold protein with ankyrin repeats 1
Bxdc1	NM_001106391	10833643	0.76	-59	0	grey	brix domain containing 1
Cryab	NM_012935	10909892	0.70	-55	0	grey	crystallin, alpha B
Dem1	NM_001107973	10879446	1.37	26	2.1	ylw	defects in morphology 1 homolog (S. cerevisiae)
Dpt	NM_001105965	10765280	0.68	-423	0	grey	dermatopontin
Epm2a	ENSRNOT00000061930	10701792	0.73	-17	0	grey	epilepsy, progressive myoclonus type 2A
Gcom1	NM_001014211	10918586	1.25	31	0	grey	GRINL1A complex locus
Homez	NM_152849	10783662	1.26	28	0	grey	homeobox and leucine zipper encoding
LOC691658	ENSRNOT00000057704	10918364	0.73	-52	0	grey	similar to anterior pharynx defective 1b homolog
Lpin1	NM_001012111	10889269	0.51	-218	3.0	ylw	lipin 1
Lrrc10	NM_001128494	10895585	0.53	-110	0	grey	leucine-rich repeat-containing 10
Midn	NM_001191577	10900592	1.32	27	1.4	brwn	midnolin
Mpzl2	NM_001106818	10909590	1.31	34	0	grey	myelin protein zero-like 2
Nedd9	NM_001011922	10794599	1.29	38	10.6	brwn	neural precursor cell expressed, developmentally down-regulated 9
Per1	NM_001034125	10734882	0.43	-68	0	grey	period homolog 1 (Drosophila)
RGD1306820	NM_001107557	10711743	0.79	-32	6.3	brwn	similar to erythroid differentiation-related factor 1
Sh3pxd2a	NM_001107606	10730661	1.37	12	9.2	brwn	SH3 and PX domains 2A
Zpbp2	NM_001007011	10738015	0.82	-13	0	grey	zona pellucida binding protein 2
**DNA REPAIR**							
Rad51l1	ENSRNOT00000006043	10885589	0.75	-15	0	grey	RAD51-like 1 (S. cerevisiae)
Rad54l2	NM_001134520	10919967	1.24	42	11.8	turq	Rad54 like 2 (S. cerevisiae)
Tp53inp1	NM_181084	10867609	0.67	-93	0	grey	tumor protein p53 inducible nuclear protein 1
**ELECTRON TRANSPORT**							
Cyba	NM_024160	10811571	1.23	13	0	grey	cytochrome b-245, alpha polypeptide
Cybb	NM_023965	10936899	1.51	139	16.2	turq	cytochrome b-245, beta polypeptide
Txnip	NM_001008767	10817552	1.36	608	3.0	turq	thioredoxin interacting protein
**EPIGENETICS**							
Hbxip	NM_001106462	10818257	1.22	61	14.7	blue	hepatitis B virus x interacting protein
Id2	NM_013060	10889379	1.33	67	3.1	brwn	inhibitor of DNA binding 2
Jmjd1c	NM_001191719	10832829	1.44	107	15.6	brwn	jumonji domain containing 1C
LOC100365770	XM_002727530	10936923	0.80	-11	0	grey	histone variant H2al2-like
Mbd1	NM_001011924	10802621	0.83	-17	3.6	turq	methyl-CpG binding domain protein 1
Pcgf5	NM_001129882	10714963	0.81	-40	0	grey	polycomb group ring finger 5
Phf15	NM_001106998	10742533	1.37	21	0	grey	PHD finger protein 15
Smyd3	NM_001025762	10770241	0.81	-14	0.8	turq	SET and MYND domain containing 3
**GROWTH FACTORS**							
Ccl5	NM_031116	10745631	1.24	18	0	grey	chemokine (C-C motif) ligand 5
Ccl6	NM_001004202	10745670	1.30	43	13.9	turq	chemokine (C-C motif) ligand 6
Ccr5	NM_053960	10914618	1.36	18	19.1	turq	chemokine (C-C motif) receptor 5
Ccrl1	ENSRNOT00000015265	10919712	1.46	37	8.5	turq	chemokine (C-C motif) receptor-like 1
Creg1	NM_001105966	10765335	0.70	-71	10.4	brwn	cellular repressor of E1A-stimulated genes 1
Csf1r	NM_001029901	10802065	1.43	80	19.4	brwn	colony stimulating factor 1 receptor
Csf2rb	NM_133555	10897428	1.38	13	16.8	brwn	colony stimulating factor 2 receptor, beta, low-affinity (granulocyte-macrophage)
Pdgfra	NM_012802	10776459	1.34	68	13.7	brwn	platelet derived growth factor receptor, alpha polypeptide
Tbrg4	NM_001012154	10778361	0.81	-37	0.5	mgnt	transforming growth factor beta regulator 4
**IMMUNE RESPONSE**							
**C1qc**	**NM_001008524**	**10880731**	**1.31**	**112**	**22.7**	**brwn**	**complement component 1, q subcomponent, C chain**
**Cd163**	**NM_001107887**	**10858626**	**1.37**	**42**	**21.7**	**brwn**	**CD163 molecule**
Cd36	NM_031561	10853240	1.22	45	11.5	brwn	CD36 molecule (thrombospondin receptor)
Cd38	NM_013127	10777232	1.27	18	0	grey	CD38 molecule
Cd4	NM_012705	10865585	1.37	47	14.0	brwn	Cd4 molecule
Cd5l	NM_001025685	10816270	1.65	12	0	grey	Cd5 molecule-like
Cd97	NM_001012164	10806806	1.23	16	17.4	brwn	CD97 molecule
Cfp	NM_001106757	10936510	1.32	24	12.4	brwn	complement factor properdin
**Fcer1g**	**NM_001131001**	**10769825**	**1.40**	**110**	**22.9**	**brwn**	**Fc fragment of IgE, high affinity I, receptor for; gamma polypeptide**
Fcgr1a	NM_001100836	10825153	1.37	23	0	grey	Fc fragment of IgG, high affinity Ia, receptor (CD64)
Ifi47	NM_172019	10733056	1.53	107	15.7	brwn	interferon gamma inducible protein 47
Irf1	NM_012591	10733553	1.30	11	0	grey	interferon regulatory factor 1
Irf8	NM_001008722	10808431	1.45	18	0	grey	interferon regulatory factor 8
Klrc3	NM_001029908	10866052	1.70	15	0	grey	killer cell lectin-like receptor subfamily C, member 3
Klri1	NM_001012649	10866067	1.39	13	1.2	ylw	killer cell lectin-like receptor family I member 1

Ly6c	NM_020103	10904578	1.65	71	0	grey	Ly6-C antigen
**Ly6e**	**NM_001017467**	**10897054**	**1.32**	**89**	**23.3**	**brwn**	**lymphocyte antigen 6 complex, locus E**
Ly86	NM_001106128	10798027	1.41	61	17.5	turq	lymphocyte antigen 86
Mpa2l	ENSRNOT00000040985	10771171	1.50	16	0	grey	macrophage activation 2 like
RGD1561778	NM_001168284	10748857	1.21	11	0	grey	similar to dendritic cell-derived immunoglobulin(Ig)-like receptor 1, DIgR1 - mouse
Siglec5	NM_001106249	10706297	1.34	10	16.1	brwn	sialic acid binding Ig-like lectin 5
**METABOLISM & TRANSPORT**							
Acaca	NM_022193	10736957	0.68	-63	6.4	blue	acetyl-coenzyme A carboxylase alpha
Acsm3	NM_033231	10710295	1.31	23	0	grey	acyl-CoA synthetase medium-chain family member 3
Alas2	NM_013197	10932912	1.28	31	1.7	brwn	aminolevulinate, delta-, synthase 2
Aox3	NM_001008527	10923432	0.74	-81	5.4	brwn	aldehyde oxidase 3
Aspa	NM_024399	10744766	1.29	42	2.7	turq	aspartoacylase
Atp1b1	NM_013113	10769476	1.31	335	0	grey	ATPase, Na+/K+ transporting, beta 1 polypeptide
Cds2	NM_053643	10840085	1.23	99	18.5	turq	CDP-diacylglycerol synthase (phosphatidate cytidylyltransferase) 2
Cth	NM_017074	10827517	1.23	52	0	grey	cystathionase (cystathionine gamma-lyase)
Dnase1l3	NM_053907	10779496	1.30	19	3.8	turq	deoxyribonuclease 1-like 3
Elovl6	NM_134383	10819005	0.39	-59	0	grey	ELOVL family member 6, elongation of long chain fatty acids (yeast)
Hbb	NM_033234	10724315	1.39	434	0	grey	hemoglobin, beta
Hebp1	NM_001108651	10866417	1.22	12	1.4	blue	heme binding protein 1
Lcp1	NM_001012044	10781496	1.34	112	0	grey	lymphocyte cytosolic protein 1
Npl	NM_001013984	10768726	1.38	37	0	grey	N-acetylneuraminate pyruvate lyase
Oscp1	NM_001029923	10871984	0.79	-30	0	grey	organic solute carrier partner 1
Pdhb	NM_001007620	10779485	0.83	-239	3.2	turq	pyruvate dehydrogenase (lipoamide) beta
Plscr2	NM_001014094	10912229	1.22	16	0	grey	phospholipid scramblase 2
Ppox	NM_001105968	10769840	0.82	-17	2.4	brwn	protoporphyrinogen oxidase
Ptgs1	NM_017043	10835817	1.39	11	2.3	brwn	prostaglandin-endoperoxide synthase 1
Qpctl	NM_001106230	10719386	0.80	-15	6.8	brwn	glutaminyl-peptide cyclotransferase-like
Qsox2	NM_001109434	10843664	0.81	-24	8.6	turq	quiescin Q6 sulfhydryl oxidase 2
Scd	NM_031841	10715546	1.30	111	6.7	turq	stearoyl-CoA desaturase (delta-9-desaturase)
Slc16a1	NM_012716	10818090	1.40	133	12.0	brwn	solute carrier family 16, member 1 (monocarboxylic acid transporter 1)
Slc25a32	NM_001173334	10903482	0.78	-43	0	grey	solute carrier family 25, member 32
Slc44a2	NM_001134715	10908369	1.33	49	0	grey	solute carrier family 44, member 2
Slc8a1	NM_019268	10888110	1.27	26	5.7	ylw	solute carrier family 8 (sodium/calcium exchanger), member 1
**St3gal2**	**NM_031695**	**10808034**	**1.27**	**23**	**25.4**	**brwn**	**ST3 beta-galactoside alpha-2,3-sialyltransferase 2**
Upp2	NM_001106481	10836312	0.62	-14	0.7	brwn	uridine phosphorylase 2
**PROTEOLYSIS**							
Ctla2a	NM_001109115	10793675	1.38	34	0	grey	cytotoxic T lymphocyte-associated protein 2 alpha
Ctss	NM_017320	10817429	1.38	124	12.4	brwn	cathepsin S
Dcun1d3	NM_001024886	10718643	0.83	-35	0	grey	DCN1, defective in cullin neddylation 1, domain containing 3 (S. cerevisiae)
Laptm5	NM_053538	10872489	1.24	23	11.5	brwn	lysosomal protein transmembrane 5
Napsa	NM_031670	10706586	1.33	13	0.4	ylw	napsin A aspartic peptidase
**Psmd5**	**NM_001106569**	**10844587**	**0.82**	**-53**	**27.5**	**brwn**	**proteasome (prosome, macropain) 26S subunit, non-ATPase, 5**
Rnf125	NM_001108424	10800434	1.59	47	12.8	brwn	ring finger protein 125
Ubxn2b	NM_001107905	10867505	0.81	-13	0	grey	UBX domain protein 2B
**RECEPTORS & BINDING PROTEINS**							
Adora1	NM_017155	10767771	0.74	-14	1.0	ylw	adenosine A1 receptor
**Axl**	**NM_031794**	**10719900**	**1.30**	**54**	**25.1**	**brwn**	**Axl receptor tyrosine kinase**
Calcrl	NM_012717	10846762	1.25	30	8.8	brwn	calcitonin receptor-like
Epha2	NM_001108977	10873706	1.56	14	0	grey	Eph receptor A2
Esrra	NM_001008511	10728247	0.82	-10	8.2	brwn	estrogen related receptor, alpha
Flt1	NM_019306	10756418	1.29	16	4.4	brwn	fms-related tyrosine kinase 1
Il6ra	NM_017020	10824530	0.66	-54	8.8	brwn	interleukin 6 receptor, alpha
LOC684994	ENSRNOT00000033048	10782469	1.40	27	2.0	brwn	similar to retinoic acid receptor, beta
**Mrc1**	**NM_001106123**	**10796476**	**1.39**	**73**	**20.9**	**brwn**	**mannose receptor, C type 1**
Ms4a11	ENSRNOT00000033795	10714020	1.28	18	14.9	turq	membrane-spanning 4-domains, subfamily A, member 11
Obfc2a	NM_001014216	10923270	0.68	-158	16.4	blue	oligonucleotide/oligosaccharide-binding fold containing 2A
Plxdc2	NM_001108422	10796564	1.28	24	14.9	brwn	plexin domain containing 2
Pth1r	NM_020073	10920524	1.29	21	12.5	brwn	parathyroid hormone 1 receptor
S100a10	NM_031114	10817186	1.24	194	0	grey	S100 calcium binding protein A10
Sort1	NM_031767	10818326	1.25	86	0	grey	sortilin 1
Stbd1	NM_001013988	10775794	0.78	-12	0	grey	starch binding domain 1
**SIGNALING**							
Anxa3	NM_012823	10775628	1.25	23	0	grey	annexin A3
Arap2	NM_001107216	10772863	0.82	-27	2.4	ylw	ArfGAP with RhoGAP domain, ankyrin repeat and PH domain 2
C2cd2l	NM_001011996	10916734	0.77	-25	16.8	turq	C2 calcium-dependent domain containing 2-like
Camk2g	NM_133605	10779031	1.20	16	0	grey	calcium/calmodulin-dependent protein kinase II gamma
**Cdgap**	**NM_001105879**	**10751255**	**1.24**	**13**	**22.0**	**brwn**	**Cdc42 GTPase-activating protein**
Chka	NM_017127	10712706	1.38	50	7.6	brwn	choline kinase alpha

Csnk1d	NM_139060	10749742	0.81	-63	0	grey	casein kinase 1, delta
Emr1	NM_001007557	10931222	1.62	37	13.6	turq	EGF-like module containing, mucin-like, hormone receptor-like 1
Emr4	NM_001007558	10930717	1.46	11	8.9	turq	EGF-like module containing, mucin-like, hormone receptor-like sequence 4
Ficd	NM_001010946	10762893	0.73	-20	16.4	brwn	FIC domain containing
Frmd4b	ENSRNOT00000010717	10864371	0.77	-19	0	grey	FERM domain containing 4B
Gbp2	NM_133624	10819523	1.31	23	6.8	brwn	guanylate binding protein 2
Gnl3	NM_175580	10790002	0.80	-32	0	grey	guanine nucleotide binding protein-like 3 (nucleolar)
Gucy1a3	NM_017090	10823920	1.21	21	5.1	brwn	guanylate cyclase 1, soluble, alpha 3
Insig2	NM_178091	10767175	1.60	188	0.2	mgnt	insulin induced gene 2
Ip6k2	NM_021660	10913242	0.67	-43	4.2	brwn	inositol hexakisphosphate kinase 2
Nek7	NM_001108346	10768177	1.26	113	0	grey	NIMA (never in mitosis gene a)-related kinase 7
Pde2a	NM_031079	10709200	1.31	12	4.2	brwn	phosphodiesterase 2A, cGMP-stimulated
Pdk4	NM_053551	10860900	2.10	79	4.5	brwn	pyruvate dehydrogenase kinase, isozyme 4
Phospho2	NM_001007642	10836633	0.77	-66	3.03	green	phosphatase, orphan 2
Pik3r1	NM_013005	10821050	0.80	-88	0	grey	phosphoinositide-3-kinase, regulatory subunit 1 (alpha)
Pip4k2a	NM_053926	10799909	1.23	18	15.9	brwn	phosphatidylinositol-5-phosphate 4-kinase, type II, alpha
Plk2	NM_031821	10812954	0.78	-44	0	grey	polo-like kinase 2 (Drosophila)
Prkci	NM_032059	10822644	1.22	78	0	grey	protein kinase C, iota
Ptprc	NM_138507	10768138	1.42	48	17.6	turq	protein tyrosine phosphatase, receptor type, C
Rab3il1	NM_134411	10713833	1.28	24	0	grey	RAB3A interacting protein (rabin3)-like 1
Rac2	NM_001008384	10905316	1.47	24	2.5	brwn	ras-related C3 botulinum toxin substrate 2 (rho family, small GTP binding protein Rac2)
Rara	NM_031528	10738130	1.26	21	18.3	brwn	retinoic acid receptor, alpha
Rarb	AJ002942	10786108	1.42	26	5.0	brwn	retinoic acid receptor, beta
RGD1562101	ENSRNOT00000039652	10820145	0.72	-24	0	grey	similar to very large G-protein coupled receptor 1
**Rgl1**	**NM_001105957**	**10768588**	**1.32**	**44**	**28.0**	**brwn**	**ral guanine nucleotide dissociation stimulator,-like 1**
Ror1	NM_001108671	10870190	1.24	14	0	grey	receptor tyrosine kinase-like orphan receptor 1
Sgk1	NM_019232	10717331	1.79	241	0	grey	serum/glucocorticoid regulated kinase 1
Sh3gl3	NM_031238	10708338	0.81	-22	5.28	green	SH3-domain GRB2-like 3
Tek	NM_001105737	10869946	1.29	37	13.4	brwn	TEK tyrosine kinase, endothelial
Vav2	NM_001106563	10843959	0.77	-18	12.9	turq	vav 2 guanine nucleotide exchange factor
**TRANSCRIPTION**							
Ankrd12	NM_001108238	10930268	0.83	-37	0	grey	ankyrin repeat domain 12
Ankrd50	NM_001191606	10822950	1.21	20	0	grey	ankyrin repeat domain 50
Elf1	NM_053520	10781690	1.27	72	0	grey	E74-like factor 1
Elk3	NM_001108743	10901904	1.28	21	9.8	brwn	ELK3, member of ETS oncogene family
Fam134b	ENSRNOT00000014052	10813947	0.83	-45	0	grey	family with sequence similarity 134, member B
**Fyn**	**NM_012755**	**10830382**	**1.28**	**27**	**21.6**	**brwn**	**FYN oncogene related to SRC, FGR, YES**
Id3	NM_013058	10872972	1.65	57	7.6	brwn	inhibitor of DNA binding 3
Klrk1	NM_133512	10866041	1.52	11	0	grey	killer cell lectin-like receptor subfamily K, member 1
LOC100360929	ENSRNOT00000030597	10809086	0.79	-60	4.2	turq	Ab1-108-like
LOC690012	ENSRNOT00000033110	10825094	1.47	28	0	grey	similar to High mobility group protein 2 (HMG-2)
Myg1	NM_001005545	10899537	0.76	-36	5.3	blue	melanocyte proliferating gene 1
Pspc1	NM_001025672	10784091	0.81	-57	0	grey	paraspeckle component 1
Sall1	NM_001107415	10806314	0.46	-21	0	grey	sal-like 1 (Drosophila)
Steap4	NM_001044265	10860597	1.30	45	16.3	brwn	STEAP family member 4
**Stx2**	**NM_012748**	**10758050**	**1.24**	**27**	**26.1**	**brwn**	**syntaxin 2**
Tspyl4	NM_001012075	10830285	1.47	53	0	grey	TSPY-like 4
Zfp354a	NM_052798	10733310	0.15	-128	0	grey	zinc finger protein 354A
Zfyve9	NM_001107952	10878653	0.79	-34	0	grey	zinc finger, FYVE domain containing 9
**TRANSLATION & PROTEIN MODIFICATION**							
Arl4d	NM_001107052	10738477	1.25	23	8.7	brwn	ADP-ribosylation factor-like 4D
Ell	NM_001107304	10790978	1.26	27	8.8	brwn	elongation factor RNA polymerase II
LOC100362277	XM_002728612	10808893	1.33	17	0	grey	60S ribosomal protein L29-like
Prpf3	NM_001108559	10825074	0.79	-18	0	grey	PRP3 pre-mRNA processing factor 3 homolog (S. cerevisiae)
Slfn2	NM_001107031	10736795	1.37	34	0	grey	schlafen 2
Slfn3	NM_053687	10736802	1.56	11	0	grey	schlafen 3
Slfn8	NM_001013970	10745595	1.48	14	13.8	brwn	schlafen 8
Taf8	NM_001108197	10921447	0.76	-77	0	grey	TAF8 RNA polymerase II, TATA box binding protein (TBP)-associated factor
**MISCELLANEOUS & UNKNOWN**							
Depdc5	NM_001107229	10777832	0.79	-26	8.0	ylw	DEP domain containing 5
Fam105a	BC107649	10822107	1.28	18	11.4	turq	family with sequence similarity 105, member A
Fam118b	BC098017	10916052	0.73	-33	3.6	brwn	family with sequence similarity 118, member B
Fam50a	NM_001170573	10940358	0.75	-36	0	grey	family with sequence similarity 50, member A
Fam82b	NM_001031663	10867799	0.78	-38	0	grey	family with sequence similarity 82, member B
LOC100359529	ENSRNOT00000041624	10884408	0.83	-13	0	grey	rCG61688-like
LOC100362431	ENSRNOT00000013565	10846299	1.36	19	3.3	blue	tetratricopeptide repeat domain 30B
LOC500300	NM_001024334	10858160	0.47	-41	0	grey	similar to hypothetical protein MGC6835
R3hdm1	NM_001134867	10763654	0.79	-41	1.4	ylw	R3H domain containing 1

RGD1311422	ENSRNOT00000004718	10808094	1.38	22	9.6	blue	similar to CG8841-PA
Sgtb	NM_181629	10812798	0.71	-15	0	grey	small glutamine-rich tetratricopeptide repeat (TPR)-containing, beta
Tmem109	NM_001007736	10728812	1.23	16	0	grey	transmembrane protein 109
Tmem43	NM_001007745	10857296	1.20	27	12.8	brwn	transmembrane protein 43
**Tmtc4**	**NM_001134414**	**10786042**	**0.83**	**-21**	**24.9**	**brwn**	**transmembrane and tetratricopeptide repeat containing 4**
**Traf3ip3**	**NM_001014132**	**10766806**	**0.80**	**-16**	**23.8**	**brwn**	**TRAF3 interacting protein 3**
**EST's**							
RGD1304827	ENSRNOT00000037460	10914409	0.81	-55	11.9	ylw	hypothetical LOC316091
RGD1310185	ENSRNOT00000032301	10785361	1.39	17	2.1	turq	similar to RIKEN cDNA 9030625A04
RGD1565616	NM_001109206	10849967	1.26	52	17.3	brwn	RGD1565616
	**NC_001665**	**10930555**	**2.24**	**220**	**4.8**	**mgnt**	
	FQ213782	10866535	1.25	10	0	grey	
	FQ212254	10838352	0.77	-56	3.7	brwn	
	FQ210125	10840653	0.70	-60	8.3	blue	
	ENSRNOT00000060917	10932310	0.79	-12	0	grey	
	ENSRNOT00000052679	10903965	1.22	12	3.5	blue	
	ENSRNOT00000050793	10936475	0.81	-102	2.9	blue	
	ENSRNOT00000046708	10719104	0.77	-10	3.8	turq	
	ENSRNOT00000043345	10913501	0.71	-12	8.8	turq	
	ENSRNOT00000034355	10920991	0.81	-12	0	grey	
	ENSRNOT00000024963	10714973	0.80	-21	0.8	turq	
	ENSRNOT00000024698	10710166	0.82	-28	0.9	ylw	
	ENSRNOT00000017688	10716679	1.32	40	17.7	brwn	
	ENSRNOT00000002991	10772330	1.21	12	0.4	brwn	
	---	10864838	0.79	-20	0	grey	
	---	10821585	1.39	25	0	grey	
	---	10701507	0.65	-35	10.4	blue	
	---	10701445	0.58	-49	3.0	brwn	
	---	10701618	0.83	-63	0	grey	
	---	10700196	0.61	-43	0	grey	
	---	10700919	0.69	-30	0	grey	
	---	10700282	0.45	-25	0	grey	
	---	10700614	0.70	-15	0	grey	
	---	10701347	0.48	-14	0	grey	
	---	10700194	1.38	10	0	grey	
	---	10701494	1.58	11	0	grey	
	---	10700779	1.51	16	0	grey	
	---	10700257	1.62	17	0	grey	
	---	10701137	1.32	17	0	grey	
	---	10701596	1.62	19	0	grey	
	---	10701532	1.44	27	0	grey	
	---	10700103	2.40	39	0	grey	
	---	10700445	1.24	44	0	grey	
	---	10700356	1.23	14	0.2	mgnt	
	---	10701313	0.79	-147	27.8	turq	
	---	10701568	0.75	-35	22.0	turq	
	---	10700101	0.66	-77	13.4	turq	
	---	10701269	1.22	77	2.7	turq	
	---	10701017	0.59	-50	2.6	turq	
	---	10700573	1.76	64	1.0	turq	
	---	10700858	1.59	82	6.9	ylw	
	---	10701057	0.65	-10	1.1	ylw	
	---	10701185	0.63	-15	0.7	ylw	

I. Prostate

Gene Symbol	RefSeq/GenBank/ Ensemble Number	Affymetrix Probset ID	V/C	Mean Diff.	k.in	Mod	Gene Title
**APOPTOSIS**							
Atg7	NM_001012097	10857950	0.71	-45	31.6	turq	ATG7 autophagy related 7 homolog (S. cerevisiae)
Bmf	NM_139258	10848444	0.49	-33	4.7	brwn	Bcl2 modifying factor
Bok	NM_017312	10925636	1.30	15	1.1	turq	BCL2-related ovarian killer
Casp1	NM_012762	10907815	2.19	96	22.9	turq	caspase 1
Casp7	NM_022260	10716226	0.61	-41	23.8	turq	caspase 7
Dffa	NM_053679	10874070	0.55	-30	12.1	turq	DNA fragmentation factor, alpha subunit
Lsp1	NM_001025420	10712477	2.31	120	5.7	brwn	lymphocyte-specific protein 1
Pdcd6ip	NM_001029910	10920632	0.61	-421	6.9	turq	programmed cell death 6 interacting protein (chr8)
Pdcd6ip	NM_001029910	10921190	0.71	-36	12.6	turq	programmed cell death 6 interacting protein (chr8_random)
Syvn1	NM_001100739	10713160	0.41	-135	16.8	turq	synovial apoptosis inhibitor 1, synoviolin

Tfg	NM_001012144	10750781	0.65	-325	24.8	turq	Trk-fused gene
**Tmbim6**	**NM_019381**	**10899125**	**0.78**	**-491**	**40.1**	**turq**	**transmembrane BAX inhibitor motif containing 6**
Tnfsf10	NM_145681	10814528	2.32	101	4.8	brwn	tumor necrosis factor (ligand) superfamily, member 10
**CELL CYCLE**							
Anapc7	NM_001107142	10762009	0.69	-66	19.7	turq	anaphase promoting complex subunit 7
Bop1	NM_001024250	10904930	0.52	-71	24.2	turq	block of proliferation 1
Cables2	ENSRNOT00000008279	10852351	0.55	-26	21.2	turq	Cdk5 and Abl enzyme substrate 2
Ccnd1	NM_171992	10727260	0.53	-221	8.8	turq	cyclin D1
Ccnj	NM_001106369	10715301	0.61	-25	0	grey	cyclin J
Ccnl1	NM_053662	10823508	1.62	147	22.3	blue	cyclin L1
Cdc26	NM_001013240	10877411	1.60	139	26.0	turq	cell division cycle 26
Cdk6	NM_001191861	10860765	0.55	-135	18.8	turq	cyclin-dependent kinase 6
Cdkn1a	NM_080782	10828827	0.54	-68	29.4	turq	cyclin-dependent kinase inhibitor 1A
Cnnm2	NM_001011942	10715889	0.38	-106	16.4	turq	cyclin M2
Cnnm4	ENSRNOT00000021434	10922553	0.68	-40	13.6	turq	cyclin M4
Ddit3	NM_001109986	10895861	1.58	46	0	grey	DNA-damage inducible transcript 3
Ddit4	NM_080906	10832920	0.45	-69	14.2	blue	DNA-damage-inducible transcript 4
Gak	NM_031030	10770948	0.66	-88	11.8	turq	cyclin G associated kinase
Gas2l1	NM_001108365	10778146	0.70	-23	32.4	turq	growth arrest-specific 2 like 1
Orc5l	NM_001014186	10853004	1.47	113	13.9	turq	origin recognition complex, subunit 5-like (yeast)
**CYTOSKELETON-ECM**							
Adrm1	NM_031708	10842795	0.73	-85	20.1	turq	adhesion regulating molecule 1
Cap1	NM_022383	10879502	0.83	-174	0	grey	CAP, adenylate cyclase-associated protein 1 (yeast)
Ckap4	NM_001108740	10894549	0.56	-24	0	grey	cytoskeleton-associated protein 4
Clec4a3	NM_001005891	10858559	1.71	74	19.8	turq	C-type lectin domain family 4, member a3
Clecsf6	NM_001005899	10858573	1.75	22	11.8	turq	C-type (calcium dependent, carbohydrate recognition domain) lectin, superfamily member 6
Cltc	NM_019299	10745940	0.80	-390	3.9	turq	clathrin, heavy chain (Hc)
Copz1	NM_001108117	10899633	0.79	-136	10.9	turq	coatomer protein complex, subunit zeta 1
Dcn	NM_024129	10895075	1.73	555	10.2	turq	decorin
Dnm2	NM_013199	10908427	0.67	-99	33.7	turq	dynamin 2
Dsg2	ENSRNOT00000022270	10800410	0.35	-111	20.1	turq	desmoglein 2
Dync1li1	NM_145772	10913995	0.70	-129	16.8	turq	dynein cytoplasmic 1 light intermediate chain 1
Eml4	NM_001108008	10882543	0.69	-102	6.0	turq	echinoderm microtubule associated protein like 4
Epn3	NM_001024791	10746387	0.28	-49	17.3	turq	epsin 3
F11r	NM_053796	10765600	0.36	-311	15.3	turq	F11 receptor
Fxc1	NM_053371	10833834	0.56	-15	0	grey	fractured callus expressed transcript 1
Gjb2	NM_001004099	10784117	0.71	-11	5.7	brwn	gap junction protein, beta 2
Gsn	ENSRNOT00000025919	10835775	0.59	-19	11.2	turq	gelsolin
Inadl2	ENSRNOT00000010211	10870046	0.48	-72	17.6	turq	InaD-like 2 (Drosophila)
**Jup**	**NM_031047**	**10747330**	**0.62**	**-133**	**53.4**	**turq**	**junction plakoglobin**
Kif1b	NM_057200	10881685	0.67	-145	31.1	turq	kinesin family member 1B
Krtap9-1	ENSRNOT00000044342	10738152	1.31	11	11.9	blue	keratin associated protein 9-1
Lad1	NM_001107942	10764240	0.31	-80	0	grey	ladinin 1
Lama5	NM_001191609	10852270	0.40	-32	12.9	turq	laminin, alpha 5
Lamc2	NM_001100640	10768642	0.20	-44	2.41	green	laminin, gamma 2
Lenep	NM_053614	10824502	0.68	-28	19.8	turq	lens epithelial protein
Lgals8	NM_053862	10795724	1.75	198	9.4	turq	lectin, galactoside-binding, soluble, 8
Map1lc3a	NM_199500	10841333	0.79	-25	6.5	turq	microtubule-associated protein 1 light chain 3 alpha
Mapt	NM_017212	10738744	1.31	17	4.6	ylw	microtubule-associated protein tau
Mtm1	NM_001013047	10892884	0.81	-85	3.6	turq	myotubularin 1
Mtmr15	NM_001191633	10722552	0.70	-11	14.5	turq	myotubularin related protein 15
Myh14	NM_001100690	10721433	0.44	-40	21.3	turq	myosin, heavy chain 14
Myo1d	NM_012983	10745509	0.48	-243	6.8	turq	myosin ID
Myo5b	NM_017083	10802650	0.48	-90	10.5	turq	myosin Vb
Myo6	ENSRNOT00000016428	10911882	0.58	-130	4.9	turq	myosin VI
Ociad1	NM_001013874	10776566	0.78	-352	26.8	turq	OCIA domain containing 1
Pcdh10	ENSRNOT00000067470	10815200	0.05	-100	0	grey	protocadherin 10
Pdlim5	NM_053326	10827079	0.58	-174	14.3	turq	PDZ and LIM domain 5
Pfn3	NM_001109487	10797256	0.80	-11	0	grey	profilin 3
Plec	NM_001164304	10904812	0.74	-47	24.7	turq	plectin
**Plp2**	**NM_207601**	**10932534**	**0.52**	**-233**	**38.4**	**turq**	**proteolipid protein 2 (colonic epithelium-enriched)**
Pxn	NM_001012147	10762665	0.73	-70	11.3	turq	paxillin
Scin	NM_198748	10889731	0.57	-31	0	grey	scinderin
Spire1	NM_001107381	10804993	0.54	-86	8.3	turq	spire homolog 1 (Drosophila)
Tecr	NM_138549	10806855	0.59	-1124	37.2	turq	trans-2,3-enoyl-CoA reductase
Ttll12	NM_001135922	10905827	0.50	-42	13.3	turq	tubulin tyrosine ligase-like family, member 12
Unc84a	NM_001007147	10760687	0.82	-51	20.2	turq	unc-84 homolog A (C. elegans)
Zfc3h1	ENSRNOT00000058444	10895458	1.52	113	8.0	blue	zinc finger, C3H1-type containing

**DEVELOPMENT**							
Aamp	NM_001106920	10928890	0.70	-137	18.4	turq	angio-associated, migratory cell protein
Adnp2	NM_001127373	10805382	0.74	-32	5.7	turq	ADNP homeobox 2
Aim1	ENSRNOT00000037448	10833849	0.51	-47	21.6	turq	absent in melanoma 1
Aph1b	NM_001047090	10918368	0.71	-52	8.2	turq	anterior pharynx defective 1 homolog B (C. elegans)
Atxn7	ENSRNOT00000010103	10782564	0.69	-57	16.3	turq	ataxin 7
Cdc42se2	NM_001126089	10742674	0.65	-162	4.90	green	CDC42 small effector 2
**Clptm1**	**NM_001106232**	**10719501**	**0.53**	**-746**	**42.6**	**turq**	**cleft lip and palate associated transmembrane protein 1**
Cops6	NM_001107129	10757211	0.82	-101	7.2	turq	COP9 constitutive photomorphogenic homolog subunit 6 (Arabidopsis)
Cops7b	NM_001108807	10924924	0.74	-12	2.2	turq	COP9 constitutive photomorphogenic homolog subunit 7B (Arabidopsis)
Dach2	ENSRNOT00000006907	10934737	0.32	-43	8.9	brwn	dachshund homolog 2 (Drosophila)
Dcbld1	ENSRNOT00000000462	10830135	0.57	-35	4.9	turq	discoidin, CUB and LCCL domain containing 1
**Ddb1**	**ENSRNOT00000028188**	**10713915**	**0.63**	**-454**	**47.3**	**turq**	**damage-specific DNA binding protein 1**
Dip2b	ENSRNOT00000026289	10899209	0.54	-76	28.4	turq	DIP2 disco-interacting protein 2 homolog B (Drosophila)
Dnajb12	NM_001013907	10829944	0.64	-102	33.5	turq	DnaJ (Hsp40) homolog, subfamily B, member 12 (chr20)
Dnajb12	NM_001013907	10931089	0.64	-102	33.5	turq	DnaJ (Hsp40) homolog, subfamily B, member 12 (chrUn)
Dopey2	NM_001191660	10750333	0.48	-55	27.5	turq	dopey family member 2
Dpy19l3	NM_001135835	10721126	0.50	-94	14.3	turq	dpy-19-like 3 (C. elegans)
Efr3a	NM_001130564	10896836	0.81	-182	0	grey	EFR3 homolog A (S. cerevisiae)
Etl4	ENSRNOT00000057655	10796683	0.52	-69	34.8	turq	enhancer trap locus 4
Fkrp	NM_001025678	10719291	0.67	-11	13.2	turq	fukutin related protein
Flcn	NM_199390	10743171	0.66	-77	12.7	turq	folliculin
Fxr2	NM_001100647	10734994	0.76	-88	26.1	turq	fragile X mental retardation, autosomal homolog 2
Gmfg	NM_181091	10705539	1.41	74	8.0	turq	glia maturation factor, gamma
Harbi1l	ENSRNOT00000023237	10847410	0.61	-245	36.9	turq	harbinger transposase derived 1-like
Hbs1l	NM_001011934	10702049	0.75	-255	18.6	turq	Hbs1-like (S. cerevisiae)
Hps1	NM_040669	10715377	0.53	-43	17.5	turq	Hermansky-Pudlak syndrome 1 homolog (human)
Hps3	NM_001107664	10822463	1.40	34	5.9	turq	Hermansky-Pudlak syndrome 3 homolog (human)
Hps6	NM_181432	10715704	0.60	-14	7.1	turq	Hermansky-Pudlak syndrome 6
Htt	NM_024357	10777571	0.69	-42	11.3	turq	huntingtin
Irx2	NM_001039505	10796845	0.53	-23	8.0	turq	iroquois homeobox 2
Irx5	NM_001030044	10809563	0.64	-17	24.6	turq	iroquois homeobox 5
Lmtk2	NM_001137641	10760307	0.49	-239	34.9	turq	lemur tyrosine kinase 2
LOC100365604	ENSRNOT00000009779	10883651	0.70	-62	8.3	turq	neuroblastoma-amplified protein-like
LOC680155	ENSRNOT00000020271	10786359	1.31	129	24.9	turq	hypothetical protein LOC680155
LOC687620	ENSRNOT00000012282	10884701	1.58	42	2.0	turq	similar to mirror-image polydactyly 1
Lpin3	NM_001014184	10841888	0.58	-49	16.9	turq	lipin 3
Lrrc41	NM_001009710	10878822	0.67	-131	20.6	turq	leucine rich repeat containing 41
Lrrc47	NM_001135666	10874450	0.50	-68	30.0	turq	leucine rich repeat containing 47
Lrrc59	NM_001008280	10737518	0.80	-103	30.6	turq	leucine rich repeat containing 59
Lrrn2	NM_001177368	10763933	0.20	-37	5.1	turq	leucine rich repeat neuronal 2
Mmachc	NM_001107962	10878905	0.62	-178	30.9	turq	methylmalonic aciduria (cobalamin deficiency) cblC type, with homocystinuria
Napa	NM_080585	10704413	0.60	-290	37.6	turq	N-ethylmaleimide-sensitive factor attachment protein, alpha
Nbn	NM_138873	10867743	1.20	44	0	grey	nibrin
Ncf2	NM_001100984	10764722	1.80	12	2.0	turq	neutrophil cytosolic factor 2
Ncf4	NM_001127304	10897419	1.68	15	8.6	brwn	neutrophil cytosolic factor 4
Neu1	NM_031522	10831300	0.71	-65	0	grey	sialidase 1 (lysosomal sialidase)
Neu3	NM_054010	10723922	0.41	-25	17.2	turq	sialidase 3 (membrane sialidase)
Nipal2	NM_001130559	10903234	0.38	-133	28.6	turq	NIPA-like domain containing 2
Nipal3	ENSRNOT00000068004	10880617	0.56	-29	20.2	turq	NIPA-like domain containing 3
**Nomo1**	**NM_001108484**	**10707019**	**0.37**	**-302**	**46.7**	**turq**	**nodal modulator 1**
Nsf	NM_021748	10748118	0.59	-71	20.6	turq	N-ethylmaleimide-sensitive factor
Optn	NM_145081	10796326	0.70	-87	10.2	turq	optineurin
Park7	NM_057143	10881850	0.78	-404	14.2	brwn	Parkinson disease (autosomal recessive, early onset) 7
Per2	NM_031678	10929842	0.63	-23	1.6	turq	period homolog 2 (Drosophila)
Pnkd	NM_001134750	10924260	0.67	-43	28.2	turq	paroxysmal nonkinesiogenic dyskinesia
Popdc2	NM_199113	10754179	0.55	-39	1.49	green	popeye domain containing 2
**Ptov1**	**NM_001008304**	**10721504**	**0.54**	**-67**	**40.6**	**turq**	**prostate tumor overexpressed 1**
RGD1303130	NM_001004226	10816500	0.49	-246	30.3	turq	kidney predominant protein NCU-G1
**Scrib**	**NM_001191879**	**10904714**	**0.65**	**-42**	**38.8**	**turq**	**scribbled homolog (Drosophila)**
Sema4a	NM_001012078	10824305	0.65	-52	5.4	turq	sema domain, immunoglobulin domain (Ig), transmembrane domain (TM) and short cytoplasmic domain, (semaphorin) 4A
Sema4c	NM_001106902	10927379	0.36	-34	12.9	turq	sema domain, immunoglobulin domain (Ig), transmembrane domain (TM) and short cytoplasmic domain, (semaphorin) 4C
Sema4d	NM_001170563	10794242	0.37	-69	26.4	turq	sema domain, immunoglobulin domain (Ig), transmembrane domain (TM) and short cytoplasmic domain, (semaphorin) 4D
Sipa1l3	ENSRNOT00000038929	10720510	0.44	-38	10.6	turq	signal-induced proliferation-associated 1 like 3
Spata13	NM_001191686	10780873	0.67	-15	4.1	turq	spermatogenesis associated 13
Surf4	NM_001033868	10843893	0.63	-367	20.6	turq	surfeit 4

Surf6	NM_001015014	10742324	0.66	-35	0	grey	surfeit 6
Syn1	NM_019133	10932107	0.45	-12	0	grey	synapsin I
Tor1aip2	NM_199100	10764827	0.82	-58	10.2	turq	torsin A interacting protein 2
Trmt2a	NM_001011895	10755604	0.65	-35	0	grey	TRM2 tRNA methyltransferase 2 homolog A (S. cerevisiae)
**Trpc4ap**	**NM_001100748**	**10851056**	**0.68**	**-222**	**55.6**	**turq**	**transient receptor potential cation channel, subfamily C, member 4 associated protein**
Trpm4	NM_001136229	10721739	0.50	-30	35.6	turq	transient receptor potential cation channel, subfamily M, member 4
Uhmk1	NM_017293	10798469	0.67	-291	24.4	turq	U2AF homology motif (UHM) kinase 1
Ulk1	NM_001108341	10759248	0.65	-40	24.6	turq	Unc-51 like kinase 1 (C. elegans)
Ulk3	ENSRNOT00000025885	10910360	0.71	-10	0	grey	unc-51-like kinase 3 (C. elegans)
Wfs1	NM_031823	10773290	0.50	-70	18.9	turq	Wolfram syndrome 1 homolog (human)
Yif1b	NM_198734	10705739	0.77	-30	5.4	brwn	Yip1 interacting factor homolog B (S. cerevisiae)
**DNA REPAIR**							
Atmin	NM_001191786	10808218	0.69	-25	14.4	turq	ATM interactor (chr19)
Atmin	NM_001191786	10903264	0.63	-35	18.0	turq	ATM interactor (chr7)
Bcr	ENSRNOT00000001766	10832602	0.42	-77	31.6	turq	breakpoint cluster region
Cry1	NM_198750	10894525	0.64	-20	10.3	turq	cryptochrome 1 (photolyase-like)
Cry2	NM_133405	10847525	0.58	-144	14.4	turq	cryptochrome 2 (photolyase-like)
Dcaf10	NM_001107935	10868718	0.66	-58	9.6	turq	DDB1 and CUL4 associated factor 10
Dclre1b	NM_001025687	10825602	0.70	-34	4.5	turq	DNA cross-link repair 1B, PSO2 homolog (S. cerevisiae)
Rad54l2	NM_001134520	10919967	0.45	-157	11.8	turq	Rad54 like 2 (S. cerevisiae)
Tp53bp2	ENSRNOT00000004330	10766382	0.43	-68	15.0	turq	tumor protein p53 binding protein, 2
**ELECTRON TRANSPORT**							
Cyb5r1	NM_001013126	10764104	0.59	-99	27.7	turq	cytochrome b5 reductase 1
Prdx4	NM_053512	10933699	1.22	31	7.4	turq	peroxiredoxin 4
Txndc11	NM_001127532	10731484	0.45	-150	12.9	turq	thioredoxin domain containing 11
Txndc12	NM_001100840	10870837	1.37	212	13.4	turq	thioredoxin domain containing 12 (endoplasmic reticulum)
**Ucp2**	**NM_019354**	**10709093**	**0.45**	**-347**	**48.3**	**turq**	**uncoupling protein 2 (mitochondrial, proton carrier)**
**EPIGENETICS**							
**Bahd1**	**ENSRNOT00000013878**	**10838718**	**0.53**	**-35**	**38.3**	**turq**	**bromo adjacent homology domain containing 1**
Brd7	NM_001108440	10806323	0.75	-88	4.4	turq	bromodomain containing 7
Brwd2	ENSRNOT00000027704	10711463	0.77	-68	8.6	turq	bromodomain and WD repeat domain containing 2
Cbx4	XM_576815	10931644	0.48	-34	15.1	turq	chromobox homolog 4 (Pc class homolog, Drosophila)
Cxxc1	NM_001079698	10802603	0.73	-37	13.7	turq	CXXC finger 1 (PHD domain)
Ercc1	NM_001106228	10704808	0.70	-56	10.7	turq	excision repair cross-complementing rodent repair deficiency, complementation group 1
Hist1h2bc	NM_001109400	10798475	0.50	-22	0	grey	histone cluster 1, H2bc
Jmjd5	NM_001037196	10710773	0.64	-43	0	grey	jumonji domain containing 5
Jmjd8	NM_001014116	10732511	0.75	-152	26.2	turq	jumonji domain containing 8
Mpg	NM_012601	10741786	0.72	-11	6.4	brwn	N-methylpurine-DNA glycosylase
Phf1	NM_212538	10828459	0.76	-41	18.4	turq	PHD finger protein 1
Phf20l1	NM_001025702	10896862	1.27	64	20.0	blue	PHD finger protein 20-like 1
Pygo2	NM_001106447	10816805	0.65	-14	0	grey	pygopus 2
Rcc2	ENSRNOT00000008940	10873567	0.54	-121	31.9	turq	regulator of chromosome condensation 2
Setd8	ENSRNOT00000001407	10761727	0.64	-204	8.7	turq	SET domain containing (lysine methyltransferase) 8
Smarca4	NM_134368	10908482	0.72	-66	28.1	turq	SWI/SNF related, matrix associated, actin dependent regulator of chromatin, subfamily a, member 4
Smarcal1	NM_001108222	10924199	0.77	-29	10.5	turq	Swi/SNF related matrix associated, actin dependent regulator of chromatin, subfamily a-like 1
Thap7	NM_001105863	10752451	0.62	-18	12.2	brwn	THAP domain containing 7
**Wdr5**	**NM_001039034**	**10834704**	**0.73**	**-62**	**37.7**	**turq**	**WD repeat domain 5**
**GOLGI APPARATUS**							
**Ergic1**	**ENSRNOT00000005273**	**10741851**	**0.46**	**-345**	**51.2**	**turq**	**endoplasmic reticulum-golgi intermediate compartment (ERGIC) 1**
Ergic3	NM_001106533	10841452	0.67	-296	9.1	turq	ERGIC and golgi 3
Ap1g1	NM_134460	10807768	0.80	-134	8.0	turq	adaptor-related protein complex 1, gamma 1 subunit
Cog1	NM_001107062	10739371	0.75	-43	31.0	turq	component of oligomeric golgi complex 1
**Cog7**	**NM_001033889**	**10725427**	**0.49**	**-56**	**43.7**	**turq**	**component of oligomeric golgi complex 7**
Cog8	NM_001106182	10810839	0.66	-52	15.6	brwn	component of oligomeric golgi complex 8
Copg	NM_001031822	10857080	0.54	-259	32.2	turq	coatomer protein complex, subunit gamma
Golga3	NM_001107847	10763091	0.76	-51	7.3	turq	golgi autoantigen, golgin subfamily a, 3 (chr12)
Golga3	NM_001107847	10860783	0.75	-62	8.0	turq	golgi autoantigen, golgin subfamily a, 3 (chr4)
Golgb1	NM_138885	10754311	0.74	-37	1.8	brwn	golgi autoantigen, golgin subfamily b, macrogolgin 1
Golph3	NM_023977	10813771	0.81	-266	11.2	turq	golgi phosphoprotein 3 (coat-protein)
**Gorasp2**	**NM_001007720**	**10836755**	**0.66**	**-394**	**45.5**	**turq**	**golgi reassembly stacking protein 2**
**LOC100360302**	**ENSRNOT00000026111**	**10843761**	**0.54**	**-127**	**50.3**	**turq**	**SEC16 homolog A-like**
Manba	NM_001031655	10819245	0.55	-131	5.6	turq	mannosidase, beta A, lysosomal
Mgat4a	NM_001160155	10927472	0.53	-76	0	grey	mannosyl (alpha-1,3-)-glycoprotein beta-1,4-N-acetylglucosaminyltransferase, isozyme A

Mgat4b	NM_001127533	10733225	0.53	-83	36.7	turq	mannosyl (alpha-1,3-)-glycoprotein beta-1,4-N-acetylglucosaminyltransferase, isozyme B
Mgat5	NM_023095	10763611	0.57	-61	3.1	turq	mannosyl (alpha-1,6-)-glycoprotein beta-1,6-N-acetyl-glucosaminyltransferase
**Pigs**	**NM_001006602**	**10745081**	**0.45**	**-110**	**56.6**	**turq**	**phosphatidylinositol glycan anchor biosynthesis, class S**
**Rpn2**	**NM_031698**	**10841602**	**0.50**	**-1306**	**77.4**	**turq**	**ribophorin II**
Sec23ip	NM_001134859	10711433	0.74	-119	15.3	turq	SEC23 interacting protein
**GROWTH FACTORS**							
Sept7	NM_022616	10908674	1.39	111	15.6	turq	septin 7
Ccr2	NM_021866	10914614	3.70	107	13.3	turq	chemokine (C-C motif) receptor 2
Ccr5	NM_053960	10914618	1.84	37	19.1	turq	chemokine (C-C motif) receptor 5
Cetn3	NM_001191842	10812346	1.44	146	14.6	turq	centrin, EF-hand protein, 3 (CDC31 homolog, yeast)
Ghitm	NM_001005908	10790585	1.45	917	0	grey	growth hormone inducible transmembrane protein
**Igf2r**	**NM_012756**	**10702996**	**0.45**	**-307**	**46.4**	**turq**	**insulin-like growth factor 2 receptor**
Jag2	ENSRNOT00000019213	10892352	0.67	-12	1.8	brwn	jagged 2
RGD1305464	BC089935	10917748	0.59	-19	2.9	turq	similar to human chromosome 15 open reading frame 39
Tbrg1	NM_001009344	10830133	0.66	-129	17.2	turq	transforming growth factor beta regulator 1 (chr20)
Tbrg1	NM_001009344	10916237	0.67	-149	15.6	turq	transforming growth factor beta regulator 1 (chr8)
**IMMUNE RESPONSE**							
Cd37	NM_017124	10721728	1.51	15	2.2	brwn	CD37 molecule
Cd38	NM_013127	10777232	0.44	-63	0	grey	CD38 molecule
Cd53	NM_012523	10825809	2.04	110	12.6	turq	Cd53 molecule
Cd84	NM_001192006	10765650	1.25	12	5.8	turq	CD84 molecule
Fcgr1a	NM_001100836	10825153	1.71	38	0	grey	Fc fragment of IgG, high affinity Ia, receptor (CD64)
Fcgr2b	NM_175756	10769771	1.92	36	11.1	turq	Fc fragment of IgG, low affinity IIb, receptor (CD32)
Ifi44l	XM_227820	10834014	1.89	46	11.7	turq	interferon-induced protein 44-like
Ifit2	NM_001024753	10714900	2.27	58	13.8	turq	interferon-induced protein with tetratricopeptide repeats 2
IgG-2a	BC088423	10892499	0.08	-77	0	grey	gamma-2a immunoglobulin heavy chain
Ighg	BC095846	10892536	0.59	-11	0	grey	Immunoglobulin heavy chain (gamma polypeptide)
Igsf3	NM_001106455	10817868	0.63	-14	5.3	turq	immunoglobulin superfamily, member 3
Irf7	NM_001033691	10726758	2.53	60	0	grey	interferon regulatory factor 7
Ly49si2	NM_001009498	10866109	2.22	59	25.0	turq	immunoreceptor Ly49si2
Ly6c	NM_020103	10904578	4.43	228	0	grey	Ly6-C antigen
Ly86	NM_001106128	10798027	2.57	173	17.5	turq	lymphocyte antigen 86
Mpa2l	ENSRNOT00000040985	10771171	1.84	23	0	grey	macrophage activation 2 like
Mx2	NM_134350	10750524	2.56	169	0	grey	myxovirus (influenza virus) resistance 2
Prp2l1	ENSRNOT00000043156	10748077	0.80	-43	7.2	turq	proline rich protein 2-like 1
RGD1561778	NM_001168284	10748857	1.84	37	0	grey	similar to dendritic cell-derived immunoglobulin(Ig)-like receptor 1, DIgR1 - mouse
RGD1564284	ENSRNOT00000044584	10892509	0.12	-68	0	grey	similar to immunoglobulin 4G6 heavy chain variable region
RT1-T24-4	NM_001008826	10827820	1.74	56	4.5	turq	RT1 class I, locus T24, gene 4
Thbd	NM_031771	10850525	1.95	19	6.3	turq	thrombomodulin
Tollip	NM_001109668	10726916	0.61	-135	13.0	brwn	toll interacting protein
**METABOLISM & TRANSPORT**							
A2ld1	NM_001025634	10786036	0.64	-66	0	grey	AIG2-like domain 1
Abhd11	BC088239	10761238	0.59	-23	19.7	turq	abhydrolase domain containing 11
Acacb	NM_053922	10762792	0.34	-87	32.1	turq	acetyl-Coenzyme A carboxylase beta
Acp1	NM_021262	10890338	1.25	94	12.6	turq	acid phosphatase 1, soluble
Acp6	NM_001031645	10817675	0.74	-56	7.0	turq	acid phosphatase 6, lysophosphatidic
Afg3l1	NM_001108456	10808723	0.72	-81	16.0	turq	AFG3(ATPase family gene 3)-like 1 (S. cerevisiae)
Ahcyl2	NM_001173510	10854221	0.66	-91	6.2	turq	adenosylhomocysteinase-like 2
Akr1c13	NM_001014240	10799337	0.66	-18	0	grey	aldo-keto reductase family 1, member C13
Akr1cl1	NM_001109900	10799357	1.39	17	0	grey	aldo-keto reductase family 1, member C-like 1
Aldh1l2	NM_001191778	10894582	0.50	-31	8.0	brwn	aldehyde dehydrogenase 1 family, member L2
**Alg3**	**NM_001142363**	**10752137**	**0.54**	**-204**	**44.9**	**turq**	**asparagine-linked glycosylation 3, alpha-1,3- mannosyltransferase homolog (S. cerevisiae)**
Alg8	NM_001034127	10708760	0.73	-36	6.2	turq	asparagine-linked glycosylation 8, alpha-1,3-glucosyltransferase homolog (S. cerevisiae)
**Alkbh5**	**ENSRNOT00000034006**	**10734148**	**0.67**	**-234**	**39.3**	**turq**	**alkB, alkylation repair homolog 5 (E. coli)**
Arv1	NM_001106197	10808836	0.71	-37	5.2	turq	ARV1 homolog (S. cerevisiae)
**Atp13a1**	**NM_001106079**	**10791166**	**0.43**	**-97**	**45.0**	**turq**	**ATPase type 13A1**
Atp13a2	NM_001173432	10873606	0.43	-31	24.1	turq	ATPase type 13A2
Atp2a2	NR_027839	10758555	0.64	-609	12.6	turq	ATPase, Ca++ transporting, cardiac muscle, slow twitch 2
Atp6v0a1	NM_031604	10738237	0.61	-118	29.6	turq	ATPase, H+ transporting, lysosomal V0 subunit A1
**Atp6v0a2**	**NM_053775**	**10761639**	**0.59**	**-25**	**41.3**	**turq**	**ATPase, H+ transporting, lysosomal V0 subunit A2**
**Atp6v0c**	**NM_130823**	**10740996**	**0.61**	**-640**	**68.6**	**turq**	**ATPase, H+ transporting, lysosomal 16kDa, V0 subunit c**
Atp6v1a	NM_001108318	10751161	0.64	-213	0	grey	ATPase, H+ transporting, lysosomal V1 subunit A (chr11)
Atp6v1a	NM_001108318	10823609	0.63	-351	13.8	turq	ATPase, H+ transporting, lysosomal V1 subunit A (chr2)
Atp6v1b2	NM_057213	10791233	0.77	-264	6.3	turq	ATPase, H transporting, lysosomal V1 subunit B2

C1galt1	NM_022950	10853691	1.70	269	28.0	turq	core 1 synthase, glycoprotein-N-acetylgalactosamine 3-beta-galactosyltransferase, 1
Cant1	NM_144754	10749502	0.70	-29	8.6	blue	calcium activated nucleotidase 1
Cds2	NM_053643	10840085	0.60	-248	18.5	turq	CDP-diacylglycerol synthase (phosphatidate cytidylyltransferase) 2
Clstn1	ENSRNOT00000022100	10874095	0.39	-126	32.7	turq	calsyntenin 1
Cmpk2	NM_001108017	10883960	2.25	36	8.2	turq	cytidine monophosphate (UMP-CMP) kinase 2, mitochondrial
Cpt1a	NM_031559	10712657	0.51	-304	35.0	turq	carnitine palmitoyltransferase 1a, liver
**Ddost**	**NM_001012104**	**10873303**	**0.52**	**-947**	**56.0**	**turq**	**dolichyl-diphosphooligosaccharide-protein glycosyltransferase**
Dhcr24	NM_001080148	10870567	0.30	-189	11.8	turq	24-dehydrocholesterol reductase
Dhcr7	NM_022389	10712560	0.37	-130	25.3	turq	7-dehydrocholesterol reductase
Dolpp1	NM_001106567	10835233	0.53	-48	19.1	turq	dolichyl pyrophosphate phosphatase 1
Dpagt1	NM_199388	10909470	0.67	-134	24.8	turq	dolichyl-phosphate (UDP-N-acetylglucosamine) N-acetylglucosaminephosphotransferase 1 (GlcNAc-1-P transferase)
Eri2	NM_001191114	10725331	1.34	48	21.3	turq	exoribonuclease 2
Exoc8	NM_139043	10811947	0.51	-70	21.9	turq	exocyst complex component 8
Exosc6	ENSRNOT00000025179	10808063	0.61	-10	21.2	blue	exosome component 6
**Ganab**	**NM_001106334**	**10713706**	**0.52**	**-354**	**69.2**	**turq**	**glucosidase, alpha; neutral AB**
Gfpt1	NM_001005879	10857036	0.65	-305	0	grey	glutamine fructose-6-phosphate transaminase 1
Glb1l2	NM_001192018	10915843	0.27	-104	22.2	turq	galactosidase, beta 1-like 2
Gsk3a	NM_017344	10719798	0.72	-71	20.0	turq	glycogen synthase kinase 3 alpha
Gstm1	NM_017014	10825925	0.55	-865	6.7	turq	glutathione S-transferase mu 1
Gstt1	NM_053293	10832555	0.58	-96	4.1	turq	glutathione S-transferase theta 1
Guf1	NM_001107215	10776646	1.24	104	17.6	turq	GUF1 GTPase homolog (S. cerevisiae)
Hdlbp	NM_172039	10930015	0.68	-508	33.0	turq	high density lipoprotein binding protein
Herc2	NM_001107520	10707455	0.76	-51	24.2	turq	hect domain and RLD 2
Hexa	NM_001004443	10910490	0.65	-206	8.6	brwn	hexosaminidase A
Hk2	NM_012735	10863430	0.52	-78	35.9	turq	hexokinase 2
Hsdl1	NM_001024896	10811415	0.72	-20	3.7	turq	hydroxysteroid dehydrogenase like 1
Kat2a	NM_001107050	10747439	0.70	-14	7.2	turq	K(lysine) acetyltransferase 2A
Kctd1	ENSRNOT00000061504	10803294	0.45	-49	9.4	turq	potassium channel tetramerisation domain containing 1
Kctd3	NM_001107199	10770613	0.55	-68	29.9	turq	potassium channel tetramerisation domain containing 3
Kdm1	NM_001130098	10880691	0.62	-105	28.9	turq	lysine (K)-specific demethylase 1
Kpna1	NM_198726	10754410	0.70	-205	3.7	turq	karyopherin alpha 1
LOC100362008	ENSRNOT00000007042	10731803	0.50	-43	9.0	turq	adenylate cyclase 9
Lrat	NM_022280	10823949	1.50	23	13.2	turq	lecithin-retinol acyltransferase (phosphatidylcholine-retinol-O-acyltransferase)
Lss	NM_031049	10832378	0.41	-189	12.9	turq	lanosterol synthase (2,3-oxidosqualene-lanosterol cyclase)
Magi1	NM_001030045	10864243	0.60	-67	9.1	turq	membrane associated guanylate kinase, WW and PDZ domain containing 1
Mfsd4	NM_001109069	10767565	0.20	-70	6.1	turq	major facilitator superfamily domain containing 4
Mocos	NM_001108425	10800546	0.62	-19	3.51	green	molybdenum cofactor sulfurase
Mocs3	NM_001107804	10842493	0.45	-34	0	grey	molybdenum cofactor synthesis 3
Mthfr	ENSRNOT00000011384	10873903	0.65	-22	6.9	turq	5,10-methylenetetrahydrofolate reductase (NADPH)
Mvd	NM_031062	10811560	0.41	-49	8.8	turq	mevalonate (diphospho) decarboxylase
Naga	NM_001012120	10905705	0.47	-130	16.0	blue	N-acetyl galactosaminidase, alpha
Nags	NM_001107053	10738522	0.57	-15	0	grey	N-acetylglutamate synthase
Ndst1	NM_024361	10804582	0.57	-58	14.4	turq	N-deacetylase/N-sulfotransferase (heparan glucosaminyl) 1
Necap2	NM_199096	10881047	0.73	-28	12.6	turq	NECAP endocytosis associated 2
Nmt1	NM_148891	10738643	0.75	-131	12.5	turq	N-myristoyltransferase 1
Pcyox1l	NM_001134542	10804714	0.32	-58	19.2	turq	prenylcysteine oxidase 1 like
Pigb	NM_001108166	10918657	0.62	-15	5.7	turq	phosphatidylinositol glycan anchor biosynthesis, class B
Pigo	ENSRNOT00000013213	10876259	0.62	-48	27.7	turq	phosphatidylinositol glycan anchor biosynthesis, class O
Pitpna	NM_017231	10735974	0.73	-365	9.3	turq	phosphatidylinositol transfer protein, alpha
Pitpnm2	NM_001107139	10758302	0.61	-28	29.9	turq	phosphatidylinositol transfer protein, membrane-associated 2
Plch1	NM_001191707	10823432	0.50	-14	8.6	brwn	phospholipase C, eta 1
**Pld3**	**NM_001012167**	**10720097**	**0.46**	**-120**	**41.0**	**turq**	**phospholipase D family, member 3**
Pln	NM_022707	10830174	1.67	44	3.1	ylw	phospholamban
Ppapdc3	NM_001012349	10835498	0.70	-12	0	grey	phosphatidic acid phosphatase type 2 domain containing 3
Ptar1	NM_001105760	10714422	0.79	-33	0	grey	protein prenyltransferase alpha subunit repeat containing 1
Ptges2	NM_001107832	10835597	0.54	-68	21.1	turq	prostaglandin E synthase 2
Pus10	NM_001025278	10774577	1.40	35	0	grey	pseudouridylate synthase 10
RGD1309808	NM_001134801	10897411	0.61	-11	0	grey	similar to apolipoprotein L2; apolipoprotein L-II
**Rhbdd2**	**NM_001191827**	**10761180**	**0.49**	**-115**	**46.4**	**turq**	**rhomboid domain containing 2**
Rtn3	NM_080909	10728425	0.78	-277	26.5	turq	reticulon 3
Sc4mol	NM_080886	10787841	0.60	-374	11.2	turq	sterol-C4-methyl oxidase-like
Scamp1	NM_001100636	10820511	0.60	-280	13.2	turq	secretory carrier membrane protein 1
Scamp4	NM_031725	10900478	0.50	-155	36.3	turq	secretory carrier membrane protein 4
Scamp5	NM_031726	10917761	0.68	-18	0	grey	secretory carrier membrane protein 5
Scnn1g	NM_017046	10710580	0.22	-58	0	grey	sodium channel, nonvoltage-gated 1, gamma
Sec24a	NM_001105780	10742509	0.64	-261	18.9	turq	SEC24 family, member A (S. cerevisiae)
Sec63	NM_001107637	10830583	0.72	-210	6.7	turq	SEC63 homolog (S. cerevisiae)

Shpk	NM_001033682	10735669	0.59	-26	0	grey	sedoheptulokinase
Slc11a1	NM_001031658	10924286	1.58	10	0	grey	solute carrier family 11 (proton-coupled divalent metal ion transporters), member 1
Slc15a4	NM_144758	10758094	0.75	-27	20.9	turq	solute carrier family 15, member 4
Slc16a10	NM_138831	10833635	0.43	-150	32.2	turq	solute carrier family 16 (monocarboxylic acid transporters), member 10
Slc25a29	NM_001010958	10892147	0.24	-28	20.7	turq	solute carrier family 25, member 29
Slc25a44	NM_001108947	10824300	0.50	-98	21.6	turq	solute carrier family 25, member 44
Slc26a6	NM_001143817	10913311	0.52	-21	19.4	turq	solute carrier family 26, member 6
Slc28a2	NM_031664	10849275	3.01	146	2.2	turq	solute carrier family 28 (sodium-coupled nucleoside transporter), member 2
Slc29a3	NM_181639	10833005	0.54	-76	11.8	turq	solute carrier family 29 (nucleoside transporters), member 3
Slc2a1	NM_138827	10871521	0.36	-157	22.1	turq	solute carrier family 2 (facilitated glucose transporter), member 1
Slc35d1	NM_001106668	10878361	0.50	-55	5.9	turq	solute carrier family 35 (UDP-glucuronic acid/UDP-N-acetylgalactosamine dual transporter), member D1
**Slc35e1**	**NM_001109107**	**10787121**	**0.39**	**-120**	**40.8**	**turq**	**solute carrier family 35, member E1**
Slc36a1	NM_130415	10733697	0.47	-95	31.2	turq	solute carrier family 36 (proton/amino acid symporter), member 1
Slc37a1	NM_001011944	10829116	0.55	-34	12.8	turq	solute carrier family 37 (glycerol-3-phosphate transporter), member 1
Slc39a11	NM_001013042	10748765	0.53	-70	22.1	turq	solute carrier family 39 (metal ion transporter), member 11
Slc3a2	NM_019283	10728507	0.80	-286	14.9	turq	solute carrier family 3 (activators of dibasic and neutral amino acid transport), member 2
Slc44a1	NM_001033852	10869106	0.66	-149	6.9	turq	solute carrier family 44, member 1
Slc46a3	NM_001024968	10756411	0.35	-77	29.0	turq	solute carrier family 46, member 3
Slc4a2	NM_017048	10860021	0.61	-48	28.3	turq	solute carrier family 4 (anion exchanger), member 2
Slc7a1	NM_013111	10756393	0.30	-91	27.5	turq	solute carrier family 7 (cationic amino acid transporter, y+ system), member 1
Slc9a3r1	NM_021594	10739490	0.44	-361	5.0	turq	solute carrier family 9 (sodium/hydrogen exchanger), member 3 regulator 1
Slc9a8	NM_001025281	10842440	0.57	-112	22.1	turq	solute carrier family 9 (sodium/hydrogen exchanger), member 8
Slco2a1	NM_022667	10912614	0.54	-51	11.4	turq	solute carrier organic anion transporter family, member 2a1
Soat1	NM_031118	10768979	0.74	-56	5.2	turq	sterol O-acyltransferase 1
Sphk2	NM_001012066	10721883	0.53	-82	0	grey	sphingosine kinase 2
Sqrdl	NM_001047913	10852636	1.25	54	0	grey	sulfide quinone reductase-like (yeast)
Srd5a1	NM_017070	10793310	0.51	-49	9.7	turq	steroid-5-alpha-reductase, alpha polypeptide 1 (3-oxo-5 alpha-steroid delta 4-dehydrogenase alpha 1)
St8sia4	NM_053914	10930094	1.49	15	4.1	brwn	ST8 alpha-N-acetyl-neuraminide alpha-2,8-sialyltransferase 4
Stard4	NM_001106159	10803681	0.49	-47	4.4	brwn	StAR-related lipid transfer (START) domain containing 4
Stt3a	NM_001134749	10916125	0.70	-642	14.9	turq	STT3, subunit of the oligosaccharyltransferase complex, homolog A (S. cerevisiae)
Tk2	NM_001106166	10805731	0.82	-20	6.8	turq	thymidine kinase 2, mitochondrial
Tnpo2	NM_001107166	10809967	0.57	-199	37.3	turq	transportin 2 (importin 3, karyopherin beta 2b)
Trak2	NM_133560	10928307	0.72	-79	9.5	turq	trafficking protein, kinesin binding 2
Trappc10	NM_001173528	10829218	0.46	-155	24.5	turq	trafficking protein particle complex 10
Ttpal	NM_001106537	10842032	0.51	-35	0	grey	tocopherol (alpha) transfer protein-like
Ubiad1	NM_001107993	10881619	0.69	-21	16.3	turq	UbiA prenyltransferase domain containing 1
Ubp1	NM_001191819	10913942	0.70	-56	0	grey	upstream binding protein 1 (LBP-1a)
Uggt1	NM_133596	10927285	0.47	-347	22.9	turq	UDP-glucose glycoprotein glucosyltransferase 1
Xpo1	NM_053490	10774472	1.40	173	11.2	blue	exportin 1, CRM1 homolog (yeast)
**PROTEOLYSIS**							
Aktip	NM_001011926	10806283	0.64	-273	7.0	turq	AKT interacting protein
Capn5	NM_134461	10723792	0.48	-32	2.55	green	calpain 5
Cpa3	NM_019300	10822493	2.30	34	11.9	turq	carboxypeptidase A3, mast cell
Cpd	NM_012836	10736486	0.67	-196	3.3	turq	carboxypeptidase D
Cst3	NM_012837	10850563	1.76	622	21.7	turq	cystatin C
**Dpp9**	**XM_001061312**	**10930990**	**0.56**	**-91**	**58.5**	**turq**	**dipeptidyl peptidase 9**
Faf2	NM_001017445	10797420	0.73	-75	6.4	blue	Fas associated factor family member 2
LOC363188	NM_001178071	10921462	0.62	-150	21.8	turq	similar to ubiquitin protein ligase E3 component n-recognin 2
Mcpt1	U67915	10783998	2.08	38	19.0	turq	mast cell protease 1
Mcpt2	ENSRNOT00000027988	10780561	1.78	25	17.4	turq	mast cell protease 2
**Mgrn1**	**NM_001013964**	**10740649**	**0.60**	**-126**	**45.1**	**turq**	**mahogunin, ring finger 1**
Mon2	BC105870	10902804	0.70	-130	5.6	turq	MON2 homolog (S. cerevisiae)
Pctk3	NM_001100506	10767577	0.53	-58	23.8	turq	PCTAIRE protein kinase 3
**Pmpca**	**NM_001003673**	**10834432**	**0.78**	**-204**	**37.9**	**turq**	**peptidase (mitochondrial processing) alpha**
Psmb8	NM_080767	10831595	1.38	33	0	grey	proteasome (prosome, macropain) subunit, beta type 8 (large multifunctional peptidase 7)
Psmc4	NM_057122	10893366	0.67	-315	18.9	turq	proteasome (prosome, macropain) 26S subunit, ATPase, 4
**Psmd2**	**NM_001031639**	**10755357**	**0.68**	**-450**	**44.9**	**turq**	**proteasome (prosome, macropain) 26S subunit, non-ATPase, 2**
Rce1	NM_001100585	10727606	0.53	-50	0	grey	RCE1 homolog, prenyl protein peptidase (S. cerevisiae)
Rnf10	NM_001011904	10758991	0.64	-201	28.8	turq	ring finger protein 10
Rnf144b	NM_001108881	10797728	0.64	-66	7.8	turq	ring finger protein 144B
Rnf146	NM_022518	10702428	0.78	-25	0	grey	ring finger protein 146
Rnf160	NM_001024235	10752865	0.74	-92	10.5	turq	ring finger protein 160
**Rnf26**	**NM_001113748**	**10916664**	**0.60**	**-72**	**54.6**	**turq**	**ring finger protein 26**

Rnf40	NM_153471	10711139	0.71	-52	28.4	turq	ring finger protein 40
Senp2	NM_023989	10755186	0.70	-59	4.8	turq	Sumo1/sentrin/SMT3 specific peptidase 2
Sppl2b	NM_001014200	10900383	0.77	-27	31.2	turq	signal peptide peptidase-like 2B
Spsb3	NM_001106988	10732243	0.79	-38	4.3	brwn	splA/ryanodine receptor domain and SOCS box containing 3
**Uba1**	**NM_001014080**	**10936582**	**0.57**	**-528**	**42.2**	**turq**	**ubiquitin-like modifier activating enzyme 1**
Uba6	NM_001107213	10772072	1.50	173	24.2	turq	ubiquitin-like modifier activating enzyme 6
Ubc	NM_017314	10758134	0.60	-462	21.2	turq	ubiquitin C
Ube2l6	NM_001024755	10837342	1.64	31	5.4	turq	ubiquitin-conjugating enzyme E2L 6
Ube2o	ENSRNOT00000014699	10749287	0.65	-32	12.9	turq	ubiquitin-conjugating enzyme E2O
Ube4b	ENSRNOT00000055572	10881740	0.56	-99	27.9	turq	ubiquitination factor E4B (UFD2 homolog, yeast)
Ubfd1	NM_001034911	10710611	0.71	-54	6.7	turq	ubiquitin family domain containing 1
Ubqln1	NM_053747	10793878	0.62	-330	23.1	turq	ubiquilin 1
**Usp10**	**NM_001034146**	**10808362**	**0.44**	**-138**	**44.1**	**turq**	**ubiquitin specific peptidase 10**
Usp19	NM_001001516	10793175	0.79	-77	20.0	turq	ubiquitin specific peptidase 19
Usp31	NM_001107548	10725407	0.68	-29	0	grey	ubiquitin specific peptidase 31
Usp4	NM_001135012	10793106	0.72	-157	1.67	green	ubiquitin specific peptidase 4 (proto-oncogene)
Usp48	NM_198785	10873138	0.76	-63	4.6	turq	ubiquitin specific peptidase 48
Vps11	NM_001108138	10916768	0.65	-81	24.0	turq	vacuolar protein sorting 11 homolog (S. cerevisiae)
Vps13a	NM_001100975	10729124	1.46	177	16.0	blue	vacuolar protein sorting 13 homolog A (S. cerevisiae)
Vps33b	NM_022286	10708188	0.56	-55	17.7	turq	vacuolar protein sorting 33 homolog B (yeast)
Vps37c	NM_001107463	10713966	0.68	-30	13.7	turq	vacuolar protein sorting 37 homolog C (S. cerevisiae)
Vps39	NM_001012186	10848801	0.65	-75	22.0	turq	vacuolar protein sorting 39 homolog (S. cerevisiae)
Vps53	NM_001105813	10744999	0.75	-81	8.5	turq	vacuolar protein sorting 53 homolog (S. cerevisiae)
Vps72	NM_001107695	10817324	0.63	-62	29.0	turq	vacuolar protein sorting 72 homolog (S. cerevisiae)
**RECEPTORS & BINDING PROTEINS**							
Abca3	BC088202	10732021	0.40	-94	24.8	turq	ATP-binding cassette, sub-family A (ABC1), member 3
Abca7	NM_207598	10900651	0.43	-23	16.4	turq	ATP-binding cassette, sub-family A (ABC1), member 7
Abcc4	NM_133411	10785826	0.51	-63	7.5	turq	ATP-binding cassette, sub-family C (CFTR/MRP), member 4
**Abcf1**	**NM_001109883**	**10827846**	**0.68**	**-105**	**37.6**	**turq**	**ATP-binding cassette, sub-family F (GCN20), member 1**
Aplp1	NM_001100802	10720572	0.63	-16	2.76	green	amyloid beta (A4) precursor-like protein 1
Becn1	NM_053739	10747620	0.59	-168	30.1	turq	beclin 1, autophagy related
Ctu1	NM_001106251	10706381	0.61	-12	2.2	brwn	cytosolic thiouridylase subunit 1 homolog (S. pombe)
Cxadrl1	NM_199406	10820153	0.47	-13	0	grey	coxsackie virus and adenovirus receptor-like 1 (chr2)
Cxadrl1	NM_199406	10919443	0.47	-13	0	grey	coxsackie virus and adenovirus receptor-like 1 (chr8)
Epha1	NM_001107858	10862262	0.46	-24	0	grey	Eph receptor A1
Fgfbp1	NM_022603	10773146	0.48	-12.9	4.36	blue	fibroblast growth factor binding protein 1
F2rl1	NM_053897	10820577	0.65	-12	6.6	turq	coagulation factor II (thrombin) receptor-like 1
Fzd5	NM_173838	10928533	0.41	-56	28.2	turq	frizzled homolog 5 (Drosophila)
Heatr1	NM_001108418	10799087	0.64	-44	3.0	turq	HEAT repeat containing 1
Heatr5b	NM_001191064	10887861	0.68	-41	23.2	turq	HEAT repeat containing 5B
Htr2b	NM_017250	10929566	1.93	16	0	grey	5-hydroxytryptamine (serotonin) receptor 2B
Htr5b	NM_024395	10767186	0.42	-11	0	grey	5-hydroxytryptamine (serotonin) receptor 5B
Il1r1	NM_013123	10922826	0.51	-79	0	grey	interleukin 1 receptor, type I
Il7r	NM_001106418	10821851	1.65	14	14.8	turq	interleukin 7 receptor
**Kdelr2**	**NM_001013122**	**10760397**	**0.74**	**-233**	**38.0**	**turq**	**KDEL (Lys-Asp-Glu-Leu) endoplasmic reticulum protein retention receptor 2**
Ldlr	NM_175762	10908521	0.33	-215	14.0	turq	low density lipoprotein receptor
Leng8	NM_001037790	10719006	1.77	91	13.9	blue	leukocyte receptor cluster (LRC) member 8
LOC500726	ENSRNOT00000030246	10887516	0.46	-98	30.8	turq	similar to KARP-1 binding protein 1
**Lrp10**	**NM_001037777**	**10780218**	**0.41**	**-190**	**63.8**	**turq**	**low-density lipoprotein receptor-related protein 10**
M6pr	NM_001007700	10934772	0.69	-498	0	grey	mannose-6-phosphate receptor, cation dependent
Maea	NM_001008319	10773613	0.69	-144	16.5	turq	macrophage erythroblast attacher
Ms4a11	ENSRNOT00000033795	10714020	2.31	62	14.9	turq	membrane-spanning 4-domains, subfamily A, member 11
Ms4a6a	ENSRNOT00000032937	10728918	3.11	47	14.6	turq	membrane-spanning 4-domains, subfamily A, member 6A
N4bp1	ENSRNOT00000049285	10806365	0.74	-48	15.1	turq	Nedd4 binding protein 1
Olr1749	NM_001001429	10827669	0.62	-11	0	grey	olfactory receptor 1749
Olr518	NM_001000676	10837485	0.54	-11	17.5	blue	olfactory receptor 518
Olr964	NM_001001073	10893355	0.54	-15	18.4	blue	olfactory receptor 964
Paqr7	NM_001034081	10872808	0.54	-172	25.5	turq	progestin and adipoQ receptor family member VII
**Plxnb2**	**NM_001108106**	**10906169**	**0.50**	**-237**	**48.0**	**turq**	**plexin B2**
Pnpla6	ENSRNOT00000001298	10759662	0.59	-26	13.4	turq	patatin-like phospholipase domain containing 6
Pvrl1	ENSRNOT00000008614	10909394	0.66	-19	6.4	turq	poliovirus receptor-related 1
Pvrl2	NM_001012064	10719549	0.47	-64	22.8	turq	poliovirus receptor-related 2
Ranbp1	NM_001108324	10752334	1.30	153	4.0	turq	RAN binding protein 1
Ranbp3	ENSRNOT00000057538	10837478	0.75	-58	8.4	turq	RAN binding protein 3
Rtp4	NM_001108321	10755088	3.06	72	0	grey	receptor (chemosensory) transporter protein 4
Sorl1	NM_053519	10916493	0.33	-161	22.2	turq	sortilin-related receptor, LDLR class A repeats-containing
Sort1	NM_031767	10818326	0.64	-172	0	grey	sortilin 1
Taar7g	NM_175588	10717274	0.65	-11	0	grey	trace amine-associated receptor 7g
Tyrobp	NM_212525	10705874	1.21	11	0	grey	Tyro protein tyrosine kinase binding protein

Vom1r56	NM_001008929	10704297	0.63	-11	0	grey	vomeronasal 1 receptor, 56
Vom1r74	NM_001008911	10855818	0.62	-14	1.86	green	vomeronasal 1 receptor, 74
**SIGNALING**							
Acvr1b	NM_199230	10899367	0.45	-93	9.1	turq	activin A receptor, type IB
Arhgap27	NM_198759	10748042	0.53	-16	0	grey	Rho GTPase activating protein 27
Arhgef19	NM_001108692	10873688	0.48	-13	0	grey	Rho guanine nucleotide exchange factor (GEF) 19
Bst2	NM_198134	10790811	2.52	138	0	grey	bone marrow stromal cell antigen 2
C2cd2l	NM_001011996	10916734	0.59	-51	16.8	turq	C2 calcium-dependent domain containing 2-like
Camkk1	NM_031662	10735615	0.57	-11	4.1	turq	calcium/calmodulin-dependent protein kinase kinase 1, alpha
Cdc42bpg	NM_001130013	10713278	0.49	-44	10.4	turq	CDC42 binding protein kinase gamma (DMPK-like)
Cdc42ep4	NM_001107063	10748795	0.68	-38	8.4	blue	CDC42 effector protein (Rho GTPase binding) 4
Cdcp1	NM_001106869	10921107	0.29	-27	7.2	turq	CUB domain containing protein 1
Cerk	NM_001134861	10906024	0.65	-39	1.88	green	ceramide kinase
Chn1	NM_032083	10846220	0.62	-41	0	grey	chimerin (chimaerin) 1
Csnk1a1	NM_053615	10802114	0.82	-353	5.0	brwn	casein kinase 1, alpha 1
Csrnp1	NM_001108786	10920967	0.55	-25	5.3	turq	cysteine-serine-rich nuclear protein 1
Cytip	NM_001012086	10845407	1.68	39	0	grey	cytohesin 1 interacting protein
Dab2ip	NM_138710	10835779	0.55	-34	13.6	turq	DAB2 interacting protein
Dapp1	NM_001108568	10826985	1.62	14	0	grey	dual adaptor of phosphotyrosine and 3-phosphoinositides
Dock7	NM_001191574	10878221	0.71	-65	15.7	turq	dedicator of cytokinesis 7
Dok4	NM_001108438	10805976	0.59	-20	1.2	brwn	docking protein 4
Entpd4	NM_001108384	10781290	0.72	-102	23.5	turq	ectonucleoside triphosphate diphosphohydrolase 4
Entpd6	NM_053498	10840684	0.47	-72	32.5	turq	ectonucleoside triphosphate diphosphohydrolase 6
Ern1	NM_001191926	10748314	0.37	-112	23.2	turq	endoplasmic reticulum to nucleus signaling 1
Farp1	NM_001107287	10782080	0.43	-31	11.7	turq	FERM, RhoGEF (Arhgef) and pleckstrin domain protein 1 (chondrocyte-derived)
Fermt1	NM_001106515	10850175	0.61	-13	3.4	ylw	fermitin family homolog 1 (Drosophila)
Glmn	NM_001105993	10771049	1.41	25	7.1	turq	glomulin, FKBP associated protein
Gpr107	NM_001107828	10835303	0.69	-141	22.0	turq	G protein-coupled receptor 107
Gpr157	NM_001012107	10874118	0.56	-12	13.9	turq	G protein-coupled receptor 157
Gprasp1	NM_134386	10940429	0.76	-12	3.7	turq	G protein-coupled receptor associated sorting protein 1
Gprc5c	BC168227	10739455	0.43	-88	0	grey	G protein-coupled receptor, family C, group 5, member C
Gria3	NM_001112742	10936215	1.44	12	2.2	turq	glutamate receptor, ionotrophic, AMPA 3
Hyou1	NM_138867	10909482	0.36	-639	35.3	turq	hypoxia up-regulated 1
Insig1	NM_022392	10861986	0.62	-390	18.9	turq	insulin induced gene 1
Iqgap1	NM_001108489	10723156	0.68	-126	0	grey	IQ motif containing GTPase activating protein 1
Itgb4	NM_013180	10739721	0.44	-33	0	grey	integrin, beta 4
Itpr3	NM_013138	10828516	0.38	-41	21.3	turq	inositol 1,4,5-triphosphate receptor, type 3
LOC363328	XR_007893	10930988	0.59	-13	0.90	green	similar to TICAM-1
Lpar3	NM_023969	10819705	0.64	-60	0	grey	lysophosphatidic acid receptor 3
Madd	NM_053585	10847308	0.56	-30	23.4	turq	MAP-kinase activating death domain
Mapk13	NM_019231	10828778	0.31	-54	28.7	turq	mitogen activated protein kinase 13
**Mapk8ip1**	**NM_053777**	**10847509**	**0.45**	**-114**	**42.6**	**turq**	**mitogen-activated protein kinase 8 interacting protein 1**
Mare	NM_001034936	10732592	0.54	-46	37.4	turq	alpha globin regulatory element containing gene
Mark2	NM_021699	10728396	0.55	-94	27.2	turq	MAP/microtubule affinity-regulating kinase 2
**Mbrl**	**NM_001100886**	**10893961**	**0.75**	**-120**	**45.9**	**turq**	**membralin**
Minpp1	NM_019263	10719148	0.39	-84	13.6	turq	multiple inositol polyphosphate histidine phosphatase 1
Mlec	NM_001013983	10759018	0.74	-159	11.9	turq	malectin
Ncln	NM_001014082	10893484	0.57	-102	17.7	turq	nicalin homolog (zebrafish)
Nkiras2	NM_001105839	10738216	0.71	-25	27.1	turq	NFKB inhibitor interacting Ras-like 2
Pacsin2	NM_130740	10898179	0.62	-188	0	grey	protein kinase C and casein kinase substrate in neurons 2
Pdp1	NM_019372	10875532	1.47	58	14.1	turq	pyruvate dehyrogenase phosphatase catalytic subunit 1
Pex26	NM_001106618	10867065	0.67	-53	10.3	turq	peroxisomal biogenesis factor 26
**Pgap2**	**NM_053895**	**10709336**	**0.47**	**-200**	**44.1**	**turq**	**post-GPI attachment to proteins 2**
Phlda1	NM_017180	10895406	0.61	-29	2.70	green	pleckstrin homology-like domain, family A, member 1
Pi4k2a	NM_053735	10730312	0.74	-74	10.0	turq	phosphatidylinositol 4-kinase type 2 alpha
Pik3r2	NM_022185	10787472	0.71	-27	5.6	turq	phosphoinositide-3-kinase, regulatory subunit 2 (beta)
Plekha7	NM_001144861	10725078	0.66	-11	0	grey	pleckstrin homology domain containing, family A member 7
Plekhm3	ENSRNOT00000032782	10928535	0.52	-56	7.6	turq	pleckstrin homology domain containing, family M, member 3
Ppp2r2a	NM_053999	10784702	0.78	-90	15.0	turq	protein phosphatase 2 (formerly 2A), regulatory subunit B, alpha isoform
Pprc1	NM_001106363	10715706	0.71	-15	6.7	turq	peroxisome proliferator-activated receptor gamma, coactivator-related 1
**Prkcsh**	**NM_001106806**	**10908576**	**0.50**	**-276**	**52.1**	**turq**	**protein kinase C substrate 80K-H**
**Psd3**	**ENSRNOT00000068629**	**10787765**	**4.00**	**454**	**40.3**	**turq**	**pleckstrin and Sec7 domain containing 3**
Psen1	NM_019163	10885808	0.58	-291	36.6	turq	presenilin 1
Pskh1	NM_001108897	10807430	0.65	-27	15.0	turq	protein serine kinase H1
Pstpip2	ENSRNOT00000022833	10802867	0.65	-41	12.3	turq	proline-serine-threonine phosphatase-interacting protein 2
Ptpn1	NM_012637	10842475	0.62	-87	14.7	turq	protein tyrosine phosphatase, non-receptor type 1
Ptpn23	NM_057204	10920422	0.77	-16	1.10	green	protein tyrosine phosphatase, non-receptor type 23
Ptprc	NM_138507	10768138	1.64	69	17.6	turq	protein tyrosine phosphatase, receptor type, C
Ptprf	NM_019249	10879134	0.29	-253	16.5	turq	protein tyrosine phosphatase, receptor type, F

Ptprk	NM_001029902	10717118	0.48	-172	8.2	turq	protein tyrosine phosphatase, receptor type, K, extracellular region
Ptpro	NM_017336	10859342	1.96	18	0	grey	protein tyrosine phosphatase, receptor type, O
Rab11fip1	NM_001191555	10788720	0.37	-45	19.2	turq	RAB11 family interacting protein 1 (class I)
Rab21	NM_001004238	10902393	0.73	-186	12.6	turq	RAB21, member RAS oncogene family
Rab3a	NM_013018	10790912	0.51	-29	3.0	turq	RAB3A, member RAS oncogene family
Rab3gap2	NM_001040154	10766468	0.71	-51	28.4	turq	RAB3 GTPase activating protein subunit 2
Rabep2	NM_030585	10710848	0.75	-13	21.7	turq	rabaptin, RAB GTPase binding effector protein 2
**Rabggta**	**NM_031654**	**10783898**	**0.58**	**-57**	**47.0**	**turq**	**Rab geranylgeranyltransferase, alpha subunit**
Rasal2	NM_001107188	10769047	0.70	-39	6.0	turq	RAS protein activator like 2
Rassf3	NM_001108747	10902708	0.58	-67	7.1	turq	Ras association (RalGDS/AF-6) domain family member 3
RGD1306487	ENSRNOT00000005289	10763636	0.79	-88	25.9	turq	similar to RAB3 GTPase-activating protein
RGD1308221	NM_001134539	10810341	0.35	-69	2.1	turq	similar to TBC1 domain family, member 8 (with GRAM domain); vascular Rab-GAP/TBC-containing
RGD1565350	ENSRNOT00000015338	10876531	0.69	-11	0	grey	similar to Shb protein
Rgnef	NM_001108542	10820713	0.52	-34	0	grey	Rho-guanine nucleotide exchange factor
Rgs18	NM_001047084	10768357	2.84	160	37.0	turq	regulator of G-protein signaling 18
Rqcd1	NM_001009357	10924326	0.81	-23	1.4	turq	rcd1 (required for cell differentiation) homolog 1 (S. pombe)
Safb	NM_022394	10931414	0.70	-68	28.6	turq	scaffold attachment factor B
Samsn1	NM_130821	10752744	1.55	20	6.1	turq	SAM domain, SH3 domain and nuclear localization signals, 1
Seli	NM_001134754	10889064	0.58	-273	6.5	turq	selenoprotein I
Sipa1l3	ENSRNOT00000038929	10720506	0.37	-92	16.4	turq	signal-induced proliferation-associated 1 like 3
Smek2	NM_001108367	10774681	1.28	180	17.1	blue	SMEK homolog 2, suppressor of mek1 (Dictyostelium)
Spred2	NM_001047094	10774345	0.57	-29	0	grey	sprouty-related, EVH1 domain containing 2
Srp72	NM_001170601	10719180	0.71	-232	8.8	turq	signal recognition particle 72 (chr1)
Srp72	NM_001170601	10776340	0.80	-251	3.0	turq	signal recognition particle 72 (chr14)
**Srpr**	**NM_001034150**	**10908944**	**0.53**	**-1047**	**46.1**	**turq**	**signal recognition particle receptor ('docking protein')**
Stim1	NM_001108496	10709347	0.60	-87	17.0	turq	stromal interaction molecule 1
Stk11ip	NM_001106922	10924596	0.67	-20	7.1	turq	serine/threonine kinase 11 interacting protein
Strap	NM_001011969	10859371	0.77	-172	18.6	turq	serine/threonine kinase receptor associated protein
Susd2	NM_001106381	10832496	0.46	-77	3.85	green	sushi domain containing 2
Tbc1d10b	NM_001108921	10725965	0.58	-61	16.9	turq	TBC1 domain family, member 10b
Tbc1d15	ENSRNOT00000005207	10902375	1.24	146	16.7	turq	TBC1 domain family, member 15
Tbc1d16	XM_221188	10931651	0.42	-25	10.4	turq	TBC1 domain family, member 16
Tbc1d22b	NM_001025059	10828892	0.65	-24	5.1	turq	TBC1 domain family, member 22B
**Tbc1d7**	**NM_001108411**	**10794576**	**0.54**	**-52**	**43.5**	**turq**	**TBC1 domain family, member 7**
Tbc1d8	NM_001191067	10927612	0.47	-57	0	grey	TBC1 domain family, member 8
Tbl3	NM_001008277	10741212	0.50	-84	26.5	turq	transducin (beta)-like 3
Tnk1	NM_001107012	10744254	0.42	-18	1.5	brwn	tyrosine kinase, non-receptor, 1
Tnk2	NM_001008336	10754766	0.50	-23	10.2	turq	tyrosine kinase, non-receptor, 2
Trib2	NM_001108015	10889263	0.30	-95	0	grey	tribbles homolog 2 (Drosophila)
Trim30	ENSRNOT00000055203	10724391	1.98	10	6.4	turq	tripartite motif-containing 30
Vav3	NM_001191714	10818454	1.53	16	12.8	turq	vav 3 guanine nucleotide exchange factor
Ywhah	NM_013052	10777829	0.79	-74	13.7	turq	tyrosine 3-monooxygenase/tryptophan 5-monooxygenase activation protein, eta polypeptide
Zmynd8	NM_001100838	10851815	0.60	-68	13.1	turq	zinc finger, MYND-type containing 8
**TRANSCRIPTION**							
Ahr	NM_013149	10889660	0.65	-23	3.8	turq	aryl hydrocarbon receptor
Ankrd22	NM_001191638	10729771	0.45	-23	5.1	ylw	ankyrin repeat domain 22
Ankrd52	NM_001191875	10893139	0.51	-49	26.1	turq	ankyrin repeat domain 52
Anks3	NM_001009676	10731711	0.80	-10	0	grey	ankyrin repeat and sterile alpha motif domain containing 3
Atf6b	NM_001002809	10831425	0.54	-149	37.2	turq	activating transcription factor 6 beta
Brpf1	NM_001191572	10857739	0.60	-40	6.3	turq	bromodomain and PHD finger containing, 1
Ccdc142	ENSRNOT00000035329	10856610	0.68	-11	0	grey	coiled-coil domain containing 142
Ccdc46	NM_001105849	10739277	1.99	61	27.3	turq	coiled-coil domain containing 46
Ccdc49	NM_001108295	10746766	0.68	-26	5.6	turq	coiled-coil domain containing 49
Ccdc55	NM_001037189	10736476	1.68	108	10.4	brwn	coiled-coil domain containing 55
Ccdc6	ENSRNOT00000032159	10832705	0.52	-60	18.9	turq	coiled-coil domain containing 6
Ccdc71	NM_001024904	10793137	0.69	-22	9.7	turq	coiled-coil domain containing 71
Cdig2	ENSRNOT00000016167	10803238	0.42	-103	22.9	turq	Cdig2 protein
Clip2	NM_021997	10757810	0.46	-17	0	grey	CAP-GLY domain containing linker protein 2
Ddx28	NM_001108898	10810791	0.72	-26	20.7	turq	DEAD (Asp-Glu-Ala-Asp) box polypeptide 28
Ddx31	NM_001107824	10834875	0.71	-19	6.3	turq	DEAD (Asp-Glu-Ala-Asp) box polypeptide 31
**Ddx54**	**NM_001191548**	**10762275**	**0.62**	**-37**	**58.2**	**turq**	**DEAD (Asp-Glu-Ala-Asp) box polypeptide 54**
Ddx60	ENSRNOT00000019667	10791358	2.51	48	15.1	turq	DEAD (Asp-Glu-Ala-Asp) box polypeptide 60
Dexi	NM_001109026	10731514	0.48	-159	37.1	turq	dexamethasone-induced transcript
Dnajc10	NM_001106486	10837201	0.57	-676	0	grey	DnaJ (Hsp40) homolog, subfamily C, member 10
**Dnajc16**	**NM_001014194**	**10881178**	**0.56**	**-76**	**45.7**	**turq**	**DnaJ (Hsp40) homolog, subfamily C, member 16**
Fbxl20	NM_022272	10746856	0.59	-98	0	grey	F-box and leucine-rich repeat protein 20
Fbxo28	NM_001107203	10770434	0.69	-115	24.0	turq	F-box protein 28

Foxo3	NM_001106395	10833814	0.67	-49	21.9	turq	forkhead box O3
Foxp4	NM_001108788	10921408	0.51	-25	12.0	turq	forkhead box P4
**Gatad2a**	**NM_001013881**	**10787666**	**0.62**	**-122**	**45.5**	**turq**	**GATA zinc finger domain containing 2A**
Gse1	ENSRNOT00000023607	10808407	0.59	-23	0	grey	genetic suppressor element 1
Gtf3c4	NM_001109473	10844064	0.75	-24	0	grey	general transcription factor IIIC, polypeptide 4
Hip1r	NM_001134763	10761758	0.51	-38	24.1	turq	huntingtin interacting protein 1 related
Hoxb3	NM_001107042	10737730	0.72	-13	0	grey	homeo box B3
Ing3	NM_001034107	10853918	1.33	45	0	grey	inhibitor of growth family, member 3
Jund	NM_138875	10790948	0.53	-56	4.8	turq	jun D proto-oncogene
Klf13	NM_001109147	10722549	0.55	-37	9.9	turq	Kruppel-like factor 13
Klhl32	ENSRNOT00000050653	10875771	0.56	-15	0	grey	kelch-like 32 (Drosophila)
Ldoc1	ENSRNOT00000004529	10935662	0.22	-38	0	grey	leucine zipper, down-regulated in cancer 1
Litaf	NM_001105735	10731493	0.43	-114	7.9	turq	lipopolysaccharide-induced TNF factor
LOC100362324	ENSRNOT00000013854	10813058	0.67	-78	8.9	turq	DEAH (Asp-Glu-Ala-His) box polypeptide 29
LOC100363014	ENSRNOT00000010676	10887259	0.71	-57	4.6	turq	REST corepressor 1-like
LOC100363361	ENSRNOT00000028553	10824904	0.79	-15	5.7	turq	zinc finger protein 687-like
LOC100365635	XR_085597	10767160	1.63	23	6.0	brwn	ankyrin repeat domain 49-like
LOC684558	ENSRNOT00000027268	10787579	0.70	-74	34.3	turq	similar to regulator of nonsense transcripts 1
LOC686781	ENSRNOT00000047105	10704819	0.44	-29	10.8	turq	similar to NFkB interacting protein 1
**Mad2l2**	**NM_001012106**	**10873918**	**0.54**	**-68**	**45.0**	**turq**	**MAD2 mitotic arrest deficient-like 2 (yeast)**
Matr3	NM_019149	10707749	1.48	570	33.5	turq	matrin 3 (chr1)
Matr3	NM_019149	10781787	1.48	626	23.2	turq	matrin 3 (chr15)
**Matr3**	**NM_019149**	**10800943**	**1.47**	**553**	**38.9**	**turq**	**matrin 3 (chr18)**
Mcts2	XM_002726252	10840888	1.60	109	7.7	turq	malignant T cell amplified sequence 2
Med13l	GU566026	10762389	0.69	-49	23.1	turq	mediator complex subunit 13-like
Med16	NM_001106768	10893983	0.80	-51	8.1	turq	mediator complex subunit 16
Med25	NM_001170426	10721517	0.73	-54	24.3	turq	mediator complex subunit 25
Mllt4	NM_013217	10703189	0.73	-72	3.6	turq	myeloid/lymphoid or mixed-lineage leukemia (trithorax homolog, Drosophila); translocated to, 4
Morc3	NM_001107109	10750373	1.23	47	27.2	turq	MORC family CW-type zinc finger 3
Mpp2	NM_053513	10747716	0.54	-15	3.79	green	membrane protein, palmitoylated 2 (MAGUK p55 subfamily member 2)
Mta1	NM_022588	10887593	0.72	-59	17.8	turq	metastasis associated 1
Ndrg3	NM_001013923	10851219	0.66	-78	0	grey	N-myc downstream regulated gene 3
Nfe2l1	NM_001108293	10746602	0.69	-213	16.3	turq	nuclear factor, erythroid derived 2,-like 1
Nfil3	NM_053727	10794225	0.66	-13	0	grey	nuclear factor, interleukin 3 regulated
**Nfix**	**NM_030866**	**10806665**	**0.58**	**-222**	**38.7**	**turq**	**nuclear factor I/X (CCAAT-binding transcription factor)**
Noc4l	NM_001014129	10759342	0.61	-29	33.4	turq	nucleolar complex associated 4 homolog (S. cerevisiae)
N-pac	NM_001007800	10731668	0.70	-168	26.5	turq	cytokine-like nuclear factor n-pac
Os9	NM_001007265	10902892	0.57	-269	16.9	turq	osteosarcoma amplified 9
Pdlim1	NM_017365	10730057	0.49	-155	18.4	turq	PDZ and LIM domain 1
Pelp1	NM_001024270	10744441	0.70	-33	23.1	turq	proline, glutamic acid and leucine rich protein 1
Polr3e	NM_001108503	10710513	0.80	-28	2.5	turq	polymerase (RNA) III (DNA directed) polypeptide E
Pols	NM_001107333	10793317	0.68	-22	3.7	turq	polymerase (DNA directed) sigma
Sart3	NM_001107156	10759179	0.73	-18	19.9	turq	squamous cell carcinoma antigen recognized by T-cells 3
Scyl3	NM_001191828	10765152	0.77	-54	0	grey	SCY1-like 3 (S. cerevisiae)
**Snd1**	**NM_022694**	**10854056**	**0.49**	**-237**	**55.0**	**turq**	**staphylococcal nuclease and tudor domain containing 1**
Sp6	NM_001108833	10737772	0.64	-12	0	grey	Sp6 transcription factor
Spty2d1	ENSRNOT00000008420	10862747	0.62	-34	6.8	blue	SPT2, Suppressor of Ty, domain containing 1 (S. cerevisiae)
Src	NM_031977	10841624	0.43	-42	0	grey	v-src sarcoma (Schmidt-Ruppin A-2) viral oncogene homolog (avian)
**Srebf2**	**NM_001033694**	**10898091**	**0.42**	**-183**	**51.8**	**turq**	**sterol regulatory element binding transcription factor 2**
Srf	NM_001109302	10921550	0.68	-52	9.1	turq	serum response factor (c-fos serum response element-binding transcription factor)
Ssr1	NM_001008891	10794721	0.62	-261	36.0	turq	signal sequence receptor, alpha
Stat2	NM_001011905	10893067	0.69	-52	17.2	turq	signal transducer and activator of transcription 2
Stx16	NM_001108610	10842635	0.73	-45	14.5	turq	syntaxin 16
Taf1d	NM_001014207	10908089	1.58	89	19.8	turq	TATA box binding protein (TBP)-associated factor, RNA polymerase I, D, 41kDa
Tceb3	NM_017103	10880665	0.83	-54	9.6	turq	transcription elongation factor B (SIII), polypeptide 3
Tcf3	NM_001107865	10863277	0.59	-36	0	grey	transcription factor 3
Tcfap2c	NM_201420	10842551	0.43	-17	0	grey	transcription factor AP-2, gamma
Tcfcp2l1	NM_001107170	10763531	0.47	-48	10.2	turq	transcription factor CP2-like 1
Tinf2	NM_001006962	10783868	1.28	26	0	grey	TERF1 (TRF1)-interacting nuclear factor 2
Trip4	NM_001134981	10918302	1.34	99	7.1	turq	thyroid hormone receptor interactor 4
Tssc4	NM_001013194	10712528	0.62	-21	0	grey	tumor suppressing subtransferable candidate 4
Zbtb24	NM_001098667	10830505	0.57	-11	0	grey	zinc finger and BTB domain containing 24
Zc3h7b	NM_001130695	10897970	0.66	-80	24.3	turq	zinc finger CCCH-type containing 7B
Zdhhc24	NM_001039100	10712950	0.38	-36	16.6	turq	zinc finger, DHHC-type containing 24
Zdhhc6	NM_001037652	10730946	0.71	-146	7.6	turq	zinc finger, DHHC-type containing 6
Zdhhc9	NM_001039016	10939570	0.52	-109	20.9	turq	zinc finger, DHHC-type containing 9
Zfat	NM_001134957	10904202	1.85	39	0	grey	zinc finger and AT hook domain containing

Zfhx2	NM_001098803	10783755	0.72	-11	0	grey	zinc finger homeobox 2
Zfhx3	ENSRNOT00000019408	10807688	0.48	-48	22.8	turq	zinc finger homeobox 3
Zfp169	ENSRNOT00000022932	10794448	0.61	-13	0	grey	zinc finger protein 169
Zfp185	ENSRNOT00000023109	10935854	0.37	-62	11.1	turq	zinc finger protein 185
Zfp212	ENSRNOT00000009054	10855203	0.72	-25	5.3	turq	Zinc finger protein 212
Zfp280b	NM_001106384	10832460	0.72	-19	1.0	turq	zinc finger protein 280b
Zfp354c	NM_023988	10742450	1.77	13	9.1	turq	zinc finger protein 354C
Zfp36	NM_133290	10720215	0.63	-63	10.7	turq	zinc finger protein 36
Zfp426	NM_001079943	10915324	1.43	48	22.3	turq	zinc finger protein 426
Zfp458	NM_001024299	10814151	1.87	28	20.2	turq	zinc finger protein 458
Zfp46	NM_001106691	10872991	0.50	-76	12.2	turq	zinc finger protein 46
Zfp462	ENSRNOT00000047225	10876831	0.63	-34	1.90	green	zinc finger protein 462
Zfp709l1	ENSRNOT00000042498	10893420	1.55	171	36.6	turq	zinc finger protein 709-like 1
Zfp800	NM_001109225	10861399	1.31	56	0	grey	zinc finger protein 800
Zfp9	NM_001127635	10864972	0.60	-21	0	grey	zinc finger protein 9
Zmat3	NM_022548	10814717	0.62	-14	4.5	turq	zinc finger, matrin type 3
Znf598	NM_001105770	10732167	0.61	-60	25.0	turq	zinc finger protein 598
Znf652	NM_001080207	10737651	0.73	-77	11.9	turq	zinc finger protein 652
Znf746	NM_001100852	10862388	0.81	-15	0	grey	zinc finger protein 746
Znf76	NM_001134755	10828687	0.61	-31	7.4	turq	zinc finger protein 76 (expressed in testis)
Zswim3	NM_001107801	10842197	0.68	-10	0	grey	zinc finger, SWIM-type containing 3
**TRANSLATION & PROTEIN MODIFICATION**							
**Aars**	**NM_001100517**	**10808041**	**0.60**	**-263**	**43.6**	**turq**	**alanyl-tRNA synthetase**
Aimp2	NM_001037348	10756692	0.62	-104	10.0	blue	aminoacyl tRNA synthetase complex-interacting multifunctional protein 2
**Ap3d1**	**NM_001100719**	**10893706**	**0.63**	**-223**	**38.5**	**turq**	**adaptor-related protein complex 3, delta 1 subunit**
Arf3	NM_080904	10906944	0.59	-333	15.1	turq	ADP-ribosylation factor 3
Arfgef1	ENSRNOT00000007766	10867254	0.65	-217	9.3	turq	ADP-ribosylation factor guanine nucleotide-exchange factor 1(brefeldin A-inhibited)
Bat2	NM_212462	10828046	0.72	-59	25.7	turq	HLA-B associated transcript 2
Cpeb4	NM_001106992	10741814	0.78	-103	6.3	turq	cytoplasmic polyadenylation element binding protein 4
Dph1	NM_001105809	10744907	0.41	-47	11.2	turq	DPH1 homolog (S. cerevisiae)
Eef2	NM_017245	10900306	0.70	-1112	19.7	turq	eukaryotic translation elongation factor 2
Eif1	NM_001105837	10738170	0.49	-73	2.0	brwn	eukaryotic translation initiation factor 1
Eif2b1	NM_172029	10758219	0.83	-52	22.1	turq	eukaryotic translation initiation factor 2B, subunit 1 alpha
Eif2c2	NM_021597	10904358	0.70	-78	9.1	turq	eukaryotic translation initiation factor 2C, 2
Eif3j	NM_001077670	10788079	0.79	-287	7.7	turq	eukaryotic translation initiation factor 3, subunit J
Eif4e2	NM_001108808	10925004	0.82	-49	15.1	turq	eukaryotic translation initiation factor 4E family member 2
Eif4ebp2	NM_001033069	10833147	0.56	-178	30.4	turq	eukaryotic translation initiation factor 4E binding protein 2
**Eif4g1**	**ENSRNOT00000040029**	**10755323**	**0.64**	**-330**	**55.2**	**turq**	**eukaryotic translation initiation factor 4 gamma, 1 (chr11)**
**Eif4g1**	**ENSRNOT00000040029**	**10822489**	**0.65**	**-340**	**51.6**	**turq**	**eukaryotic translation initiation factor 4 gamma, 1 (chr2)**
Eif4g2	NM_001017374	10724923	0.83	-637	5.5	turq	eukaryotic translation initiation factor 4, gamma 2 (chr1)
Eif4g2	NM_001017374	10922257	0.83	-672	6.1	turq	eukaryotic translation initiation factor 4, gamma 2 (chr9)
Eif4g2	NM_001017374	10931777	0.83	-637	5.9	turq	eukaryotic translation initiation factor 4, gamma 2 (chrUn)
Eif4h	NM_001006957	10757786	0.80	-116	31.1	turq	eukaryotic translation initiation factor 4H
Eif6	NM_001037352	10778386	0.66	-85	8.5	turq	eukaryotic translation initiation factor 6
Elac1	NM_001107406	10805181	0.69	-16	0	grey	elaC homolog 1 (E. coli)
**Elac2**	**NM_172326**	**10734395**	**0.54**	**-88**	**52.4**	**turq**	**elaC homolog 2 (E. coli)**
Ftsjd2	NM_001014031	10828913	0.74	-83	27.3	turq	FtsJ methyltransferase domain containing 2
Gemin8	NM_001007756	10937678	0.58	-55	15.9	turq	gem (nuclear organelle) associated protein 8
Hnrnph3	NM_001108532	10832869	1.47	108	4.4	blue	heterogeneous nuclear ribonucleoprotein H3 (2H9)
Hnrph1	NM_080896	10733243	1.25	239	0.8	pink	heterogeneous nuclear ribonucleoprotein H1
**Iars**	**NM_001100572**	**10797597**	**0.55**	**-158**	**37.9**	**turq**	**isoleucyl-tRNA synthetase**
LOC362473	ENSRNOT00000004476	10867320	0.81	-21	7.1	turq	similar to Elongation factor 1-gamma (EF-1-gamma) (eEF-1B gamma)
LOC690096	ENSRNOT00000012255	10774432	0.69	-28	0	grey	similar to ribosomal protein L28
Mrps27	NM_001108543	10812722	0.78	-27	7.5	turq	mitochondrial ribosomal protein S27
Npm1	NM_012992	10741907	1.69	900	3.6	turq	nucleophosmin (nucleolar phosphoprotein B23, numatrin) (chr10)
Npm1	NM_012992	10799502	1.24	254	7.2	turq	nucleophosmin (nucleolar phosphoprotein B23, numatrin) (chr17)
Npm1	NM_012992	10893361	1.32	440	9.8	turq	nucleophosmin (nucleolar phosphoprotein B23, numatrin) (chr7)
Oasl2	NM_001009682	10762747	2.98	34	5.1	turq	2'-5' oligoadenylate synthetase-like 2
**Pabpc4**	**NM_001100538**	**10871750**	**0.53**	**-394**	**54.3**	**turq**	**poly(A) binding protein, cytoplasmic 4**
Parp1	NM_013063	10766170	0.72	-74	34.5	turq	poly (ADP-ribose) polymerase 1
Pdia3	NM_017319	10839154	0.76	-1233	14.4	turq	protein disulfide isomerase family A, member 3
Pnn	NM_001109023	10884740	1.42	151	17.3	blue	pinin, desmosome associated protein
Puf60	NM_001191880	10904756	0.71	-185	21.3	turq	poly-U binding splicing factor 60
Qtrtd1	ENSRNOT00000000317	10751196	0.73	-14	0	grey	queuine tRNA-ribosyltransferase domain containing 1
Rae1	NM_001033708	10842577	0.77	-54	35.1	turq	RAE1 RNA export 1 homolog (S. pombe)
Rbm47	NM_001005882	10772657	0.54	-305	7.5	turq	RNA binding motif protein 47
RGD1561871	ENSRNOT00000046453	10895702	0.72	-12	0	grey	similar to ribosomal protein S10
Rnps1	NM_001011890	10732058	0.73	-108	18.8	turq	ribonucleic acid binding protein S1

**Rpl7a**	**NM_001114391**	**10753737**	**2.25**	**76**	**28.9**	**blue**	**ribosomal protein L7a**
Rpusd4	NM_001025284	10908960	0.56	-23	3.3	turq	RNA pseudouridylate synthase domain containing 4
Scap	NM_001100966	10913640	0.36	-180	34.0	turq	SREBF chaperone
Taf6	NM_001044225	10760846	0.83	-24	9.8	turq	TAF6 RNA polymerase II, TATA box binding protein (TBP)-associated factor
Tars2	NM_001014040	10825042	0.60	-56	18.9	turq	threonyl-tRNA synthetase 2, mitochondrial (putative)
Tfip11	NM_001008291	10806158	0.71	-35	17.4	turq	tuftelin interacting protein 11
Trpt1	NM_001106331	10713472	0.66	-14	2.2	turq	tRNA phosphotransferase 1
Tyw1	NM_001107137	10761311	0.71	-58	16.6	turq	tRNA-yW synthesizing protein 1 homolog (S. cerevisiae)
Wars	NM_001013170	10892152	0.74	-103	3.94	green	tryptophanyl-tRNA synthetase
Yars	NM_001025696	10872291	0.71	-108	21.3	turq	tyrosyl-tRNA synthetase
**MISCELLANEOUS & UNKNOWN**							
Ccdc85c	ENSRNOT00000045676	10892130	0.82	-14	9.6	turq	coiled-coil domain containing 85C
Clptm1l	NM_001108240	10717536	0.67	-429	26.8	turq	CLPTM1-like
Crip2	NM_022501	10937241	0.36	-174	19.8	turq	cysteine-rich protein 2
Dtwd1	NM_001013921	10839438	1.50	18	0	grey	DTW domain containing 1
Erich1	NM_001127680	10789381	0.61	-21	0	grey	glutamate-rich 1
**Fam108c1**	**NM_001100736**	**10723413**	**0.64**	**-53**	**38.4**	**turq**	**family with sequence similarity 108, member C1**
Fam116b	NM_001108749	10906208	0.59	-16	32.7	turq	family with sequence similarity 116, member B
Fam120a	NM_001191816	10794394	0.69	-119	12.3	turq	family with sequence similarity 120A
Fam155b	ENSRNOT00000057920	10934180	0.33	-35	0	grey	family with sequence similarity 155, member B
Fam160b1	ENSRNOT00000023247	10716303	0.61	-45	7.2	turq	family with sequence similarity 160, member B1
Fam189a1	ENSRNOT00000031488	10722580	0.42	-18	2.6	turq	family with sequence similarity 189, member A1
Fam20b	NM_001107187	10769005	0.58	-209	13.9	turq	family with sequence similarity 20, member B
**Ints3**	**ENSRNOT00000020575**	**10824624**	**0.59**	**-111**	**40.7**	**turq**	**integrator complex subunit 3**
Ints5	NM_001107576	10713703	0.65	-25	6.8	brwn	integrator complex subunit 5
**LOC100125385**	**NM_001103364**	**10897239**	**0.58**	**-78**	**41.5**	**turq**	**hypothetical protein LOC100125385**
**LOC300308**	**NM_001013952**	**10894414**	**18.82**	**337**	**####**	**green**	**similar to hypothetical protein 4930509O22**
LOC306766	NM_001014007	10794172	0.67	-119	8.1	turq	hypothetical LOC306766
LOC683674	ENSRNOT00000039191	10756740	0.56	-68	34.5	turq	similar to Protein C7orf26 homolog
**MGC93975**	**BC079041**	**10721350**	**0.57**	**-230**	**42.4**	**turq**	**similar to 2310044H10Rik protein**
**MGC94190**	**BC090318**	**10761329**	**0.52**	**-177**	**48.5**	**turq**	**similar to 0610007L01Rik protein**
Nsmce2	NM_001024876	10896785	1.26	111	5.4	turq	non-SMC element 2, MMS21 homolog (S. cerevisiae)
Omp	NM_012616	10723805	0.33	-22	0	grey	olfactory marker protein
Rcan3	NM_001012746	10880610	0.51	-30	9.4	turq	RCAN family member 3
Rftn2	ENSRNOT00000020910	10928167	1.64	21	9.7	turq	raftlin family member 2
RGD1303117	NR_037147	10720415	0.56	-47	15.2	turq	hypothetical protein LOC292764
RGD1304810	NM_001107314	10791983	0.51	-26	3.9	brwn	similar to 6430573F11Rik protein
RGD1305455	NM_001024969	10760877	0.58	-24	9.1	turq	similar to hypothetical protein FLJ10925
RGD1305572	NM_001024970	10766226	0.75	-57	23.2	turq	similar to hypothetical protein MGC30618
RGD1306151	NM_001108652	10859282	0.33	-85	34.1	turq	similar to hypothetical protein DKFZp761D0211
RGD1306271	ENSRNOT00000040391	10861946	0.66	-11	1.9	turq	similar to KIAA1549 protein
RGD1306717	NM_001107756	10847825	0.79	-22	15.7	turq	similar to hypothetical protein MGC25461
RGD1307264	ENSRNOT00000049592	10828614	0.61	-34	20.9	turq	similar to hypothetical protein FLJ20302; similar to CG31653-PA
**RGD1307394**	**NM_001108308**	**10739832**	**0.46**	**-123**	**47.8**	**turq**	**similar to hypothetical protein ET**
RGD1307615	NM_001108573	10843530	0.62	-114	25.1	turq	similar to hypothetical protein FLJ13045
RGD1307983	ENSRNOT00000022987	10877206	0.75	-43	6.2	turq	similar to HSPC043 protein
RGD1308616	ENSRNOT00000068128	10879213	0.68	-10	0	grey	similar to KIAA0467 protein
RGD1308759	NM_001106077	10787646	0.79	-32	10.8	turq	similar to KIAA0892 protein
RGD1309492	NM_001108044	10886157	0.74	-33	10.7	turq	similar to mKIAA1737 protein
RGD1310348	NM_001108323	10752309	0.39	-134	11.5	brwn	similar to Ser/Thr-rich protein T10 in DGCR region
RGD1311493	ENSRNOT00000028407	10747777	0.53	-45	10.1	turq	similar to CG13379-PA
**RGD1311849**	**ENSRNOT00000058387**	**10869843**	**0.58**	**-84**	**45.8**	**turq**	**similar to mKIAA1797 protein**
RGD1559904	NM_001107915	10875503	0.78	-47	5.4	turq	similar to mKIAA1429 protein
RGD1560909	BC158750	10922771	0.72	-54	23.9	turq	similar to DNA segment, Chr 1, Brigham & Womens Genetics 0212 expressed
RGD1561149	NM_001134629	10879978	0.53	-22	0	grey	similar to mKIAA1522 protein
RGD1561878	ENSRNOT00000045241	10841033	1.31	35	5.6	blue	similar to mKIAA0978 protein
RGD1562310	NM_001109066	10762420	0.64	-14	18.9	turq	similar to hypothetical protein FLJ21415
RGD1562657	ENSRNOT00000036185	10713081	0.60	-35	16.9	turq	similar to hypothetical protein DKFZp761E198
RGD1563422	NM_001077643	10765293	1.43	197	6.3	turq	similar to Brain protein 44
**RGD1564742**	**ENSRNOT00000041819**	**10894438**	**15.84**	**258**	**####**	**green**	**similar to hypothetical protein 4930509O22**
Rtn4ip1	NM_001107644	10830630	0.75	-50	4.3	turq	reticulon 4 interacting protein 1
**Samd9l**	**ENSRNOT00000013044**	**10860815**	**2.35**	**200**	**48.8**	**turq**	**sterile alpha motif domain containing 9-like**
Spata13	NM_001191686	10780871	0.70	-16	4.9	turq	spermatogenesis associated 13
Tctn3	ENSRNOT00000020455	10730116	0.44	-51	7.7	turq	tectonic family member 3
Tdrd3	NM_001012043	10781752	1.57	110	18.0	turq	tudor domain containing 3
Tecpr2	ENSRNOT00000030864	10887239	0.64	-16	17.4	turq	tectonin beta-propeller repeat containing 2
Tm4sf1	NM_001106434	10823278	2.56	195	0	grey	transmembrane 4 L six family member 1
**Tm9sf2**	**NM_001005554**	**10782126**	**0.72**	**-519**	**47.4**	**turq**	**transmembrane 9 superfamily member 2**
**Tm9sf4**	**NM_001025649**	**10840990**	**0.69**	**-155**	**47.2**	**turq**	**transmembrane 9 superfamily protein member 4**

Tmem102	ENSRNOT00000020578	10744233	0.53	-11	13.5	turq	transmembrane protein 102
Tmem129	NM_001126274	10773590	0.60	-88	3.8	turq	transmembrane protein 129
Tmem192	NM_001014141	10791318	0.71	-54	0	grey	transmembrane protein 192
Tmem2	NM_001107596	10714353	0.45	-90	6.9	turq	transmembrane protein 2
Tmem200b	ENSRNOT00000047917	10872529	0.40	-26	0	grey	transmembrane protein 200B
Tmem201	ENSRNOT00000047763	10881801	0.72	-14	21.6	turq	transmembrane protein 201
Tmem63a	NM_001134496	10766253	0.38	-79	20.0	turq	transmembrane protein 63a
Tmem82	ENSRNOT00000016176	10881133	0.49	-26	16.2	turq	transmembrane protein 82
Trim25	NM_001009536	10737411	0.64	-94	0	grey	tripartite motif-containing 25
Trim32	NM_001012103	10869530	0.80	-12	6.0	blue	tripartite motif-containing 32
Tsku	NM_001009965	10723822	0.55	-55	0	grey	tsukushin
Ttc17	BC168714	10847668	0.50	-109	8.8	turq	tetratricopeptide repeat domain 17
Wdr24	NM_001191084	10732500	0.61	-25	17.1	turq	WD repeat domain 24
Wdr75	ENSRNOT00000005230	10923127	0.52	-42	14.8	turq	WD repeat domain 75
**Wipi2**	**NM_001007615**	**10760468**	**0.62**	**-106**	**54.7**	**turq**	**WD repeat domain, phosphoinositide interacting 2**
Wwc1	ENSRNOT00000011061	10742048	0.51	-36	0	grey	WW and C2 domain containing 1
Wwc2	NM_001109111	10788101	0.70	-39	7.4	turq	WW and C2 domain containing 2
**Zinki**	**NM_001142758**	**10759488**	**1.48**	**108**	**43.2**	**turq**	**Arg3.1/Arc mRNA-binding zinc finger protein**
**EST's**							
LOC498750	BC089212	10856187	0.77	-174	12.6	turq	similar to cDNA sequence BC005537
MGC72612	NM_001009538	10787710	1.38	48	10.1	turq	similar to expressed sequence AI449175
RGD1304827	NM_001009437	10914407	0.51	-24	9.7	turq	hypothetical LOC316091
RGD1304884	NM_001107436	10808393	0.37	-111	22.3	turq	similar to RIKEN cDNA 6430548M08
RGD1305007	NM_001013976	10749778	0.70	-33	9.8	turq	similar to RIKEN cDNA 1110031I02
RGD1306284	BC086594	10739826	0.48	-53	19.8	turq	similar to RIKEN cDNA 1110005A03
RGD1307752	NM_001013922	10873258	0.69	-103	19.0	turq	similar to RIKEN cDNA 1110008F13
RGD1309594	BC085944	10832253	0.77	-21	0	grey	similar to RIKEN cDNA 1810043G02; DNA segment, Chr 10, Johns Hopkins University 13, expressed
RGD1310230	NM_001039015	10924762	1.47	309	0	grey	similar to RIKEN cDNA 5230400G24
RGD1310862	ENSRNOT00000028186	10741831	1.32	106	32.9	turq	similar to adult retina protein
RGD1310951	ENSRNOT00000022866	10869282	0.60	-25	1.01	green	similar to RIKEN cDNA E130308A19
RGD1311257	BC083697	10829359	0.72	-16	1.03	green	similar to C21orf70 protein
RGD1359158	BC093390	10729331	1.32	97	11.5	turq	similar to RIKEN cDNA 1110059E24 (chr1)
RGD1359158	BC093390	10877994	1.38	126	19.6	turq	similar to RIKEN cDNA 1110059E24 (chr5)
RGD1560277	ENSRNOT00000032429	10708167	0.50	-60	23.9	turq	similar to RIKEN cDNA D330012F22 gene
RGD1561557	NM_001108929	10829351	1.25	12	24.4	blue	similar to chromosome 21 open reading frame 29
RGD1565222	NM_001109101	10783592	0.75	-14	2.3	turq	similar to RIKEN cDNA 4931414P19
RGD1566266	NM_001177613	10758599	0.71	-27	16.1	turq	similar to hypothetical protein FLJ21127
	U15425	10701862	0.61	-30	0	grey	(chr1)
	U15425	10828861	0.66	-39	0	grey	(chr20)
	U15425	10850255	0.66	-39	0	grey	(chr3)
	U15425	10874216	0.66	-39	0	grey	(chr5)
	U15425	10910193	0.66	-39	0	grey	(chr8)
	NC_001665	10930560	2.01	1259	13.8	turq	
	NC_001665	10930580	2.59	1250	2.8	mgnt	
	GENSCAN00000039363	10727226	1.58	32	0	grey	
	GENSCAN00000029030	10812214	1.44	32	10.9	blue	
	FQ235271	10860801	2.37	59	20.1	turq	
	FQ233235	10875100	1.43	16	2.5	blue	
	FQ228887	10872572	1.49	10	4.3	turq	
	**FQ213989**	**10886864**	**1.80**	**32**	**46.7**	**turq**	
	FQ213737	10931252	0.55	-86	17.1	turq	
	**FQ212998**	**10886986**	**1.97**	**48**	**42.4**	**turq**	
	**ENSRNOT00000070327**	**10805227**	**1.47**	**385**	**45.2**	**turq**	
	ENSRNOT00000069482	10805225	1.36	644	34.7	turq	
	ENSRNOT00000069136	10933753	2.08	19	11.4	blue	
	ENSRNOT00000068960	10904356	0.62	-10	11.4	blue	
	ENSRNOT00000064161	10929321	1.33	10	7.3	turq	
	ENSRNOT00000060779	10741074	0.47	-15	3.0	turq	
	ENSRNOT00000060493	10794544	0.55	-141	7.6	turq	
	ENSRNOT00000059354	10751428	0.52	-11	5.7	blue	
	ENSRNOT00000056514	10706289	0.76	-14	0	grey	
	**ENSRNOT00000056375**	**10897272**	**0.50**	**-149**	**45.3**	**turq**	
	ENSRNOT00000056130	10920498	0.61	-16	7.6	turq	
	ENSRNOT00000055440	10859772	0.63	-13	0	grey	
	ENSRNOT00000054633	10901811	0.72	-10	1.8	brwn	
	ENSRNOT00000054567	10856162	0.71	-19	4.7	blue	
	ENSRNOT00000054532	10819467	0.58	-15	17.1	blue	
	ENSRNOT00000054150	10863359	0.60	-12	2.2	ylw	

	**ENSRNOT00000054133**	**10886872**	**2.17**	**56**	**52.6**	**turq**	
	**ENSRNOT00000054054**	**10886998**	**2.17**	**56**	**52.6**	**turq**	
	ENSRNOT00000054033	10733721	0.66	-13	0	grey	
	**ENSRNOT00000054021**	**10886946**	**2.15**	**61**	**45.1**	**turq**	
	**ENSRNOT00000053882**	**10722421**	**1.72**	**87**	**12.7**	**black**	
	**ENSRNOT00000053832**	**10886878**	**2.17**	**56**	**52.6**	**turq**	
	**ENSRNOT00000053573**	**10886964**	**2.10**	**34**	**44.3**	**turq**	
	ENSRNOT00000053557	10916961	0.67	-10	6.2	ylw	
	**ENSRNOT00000053450**	**10908100**	**1.76**	**533**	**43.3**	**turq**	
	ENSRNOT00000053274	10754642	1.41	19	10.5	blue	
	ENSRNOT00000053081	10722479	0.67	-11	11.8	blue	
	ENSRNOT00000053070	10886848	2.97	34	30.3	turq	
	ENSRNOT00000053006	10910762	1.81	58	12.5	blue	
	ENSRNOT00000052678	10731193	1.77	50	0	grey	
	**ENSRNOT00000052603**	**10792348**	**0.60**	**-21**	**30.7**	**blue**	
	**ENSRNOT00000052564**	**10722431**	**1.53**	**72**	**11.2**	**black**	
	ENSRNOT00000050135	10837097	0.50	-56	4.7	turq	
	ENSRNOT00000048438	10786415	0.54	-11	0	grey	
	ENSRNOT00000048403	10744828	0.55	-14	0	grey	
	ENSRNOT00000048078	10801778	1.35	22	5.8	blue	
	ENSRNOT00000048037	10892490	0.55	-13	0	grey	
	ENSRNOT00000047825	10890930	0.73	-15	8.0	blue	
	ENSRNOT00000046708	10719104	0.70	-14	3.8	turq	
	ENSRNOT00000046555	10892346	1.25	15	3.8	blue	
	ENSRNOT00000044832	10817985	0.72	-15	1.5	turq	
	ENSRNOT00000042956	10823109	0.68	-16	0	grey	
	ENSRNOT00000042764	10820004	0.74	-12	0	grey	
	**ENSRNOT00000042657**	**10814176**	**0.57**	**-17**	**31.1**	**blue**	
	ENSRNOT00000042358	10779954	0.62	-27	0	grey	
	ENSRNOT00000041811	10877530	0.49	-16	0	grey	
	ENSRNOT00000041141	10867488	0.67	-34	0	grey	
	ENSRNOT00000040550	10755985	0.75	-11	1.14	green	
	ENSRNOT00000039311	10789806	1.71	62	3.2	brwn	
	ENSRNOT00000037540	10703508	0.35	-84	0	grey	(chr1)
	ENSRNOT00000037540	10766998	0.35	-99	0	grey	(chr13)
	ENSRNOT00000037540	10781962	0.36	-83	0	grey	(chr15)
	ENSRNOT00000037540	10864711	0.36	-83	0	grey	(chr4)
	ENSRNOT00000036804	10828378	0.54	-26	0	grey	
	ENSRNOT00000035090	10940003	0.61	-15	30.3	turq	
	ENSRNOT00000034750	10710063	1.49	30	1.3	brwn	
	ENSRNOT00000032085	10722546	0.64	-12	0	grey	
	ENSRNOT00000031409	10850625	2.18	23	3.65	green	
	ENSRNOT00000031056	10909123	0.67	-11	4.1	turq	
	ENSRNOT00000030546	10766918	0.67	-17	3.4	turq	
	ENSRNOT00000030319	10878432	0.83	-17	0	grey	
	ENSRNOT00000029708	10822431	0.67	-20	2.9	ylw	
	ENSRNOT00000029274	10803222	0.61	-29	4.9	brwn	
	ENSRNOT00000028670	10728193	0.62	-87	7.7	turq	
	ENSRNOT00000026047	10724013	0.52	-24	16.6	turq	
	ENSRNOT00000025799	10869165	0.60	-31	1.97	green	
	ENSRNOT00000025790	10804783	0.81	-69	15.4	turq	
	ENSRNOT00000025728	10724047	0.68	-10	11.7	blue	
	ENSRNOT00000025594	10717933	0.62	-182	5.0	turq	
	ENSRNOT00000021070	10842200	0.59	-59	19.9	turq	
	ENSRNOT00000020754	10863355	0.81	-11	0	grey	
	ENSRNOT00000008661	10939079	0.66	-20	2.3	brwn	
	ENSRNOT00000008623	10895391	1.27	45	4.4	turq	
	ENSRNOT00000005644	10746483	0.61	-22	9.2	turq	
	ENSRNOT00000004088	10740617	0.63	-84	13.3	turq	
	ENSRNOT00000003373	10733067	1.53	71	8.4	brwn	
	ENSRNOT00000001562	10762723	0.71	-101	32.5	turq	
	ENSRNOT00000020098	10701709	0.72	-143	0	grey	
	BC090353	10728028	1.49	808	11.2	blue	
	**AB014881**	**10886924**	**2.17**	**56**	**52.6**	**turq**	
	**AB014879**	**10886908**	**2.17**	**56**	**52.6**	**turq**	
	**AB014878**	**10886954**	**2.17**	**56**	**52.6**	**turq**	
	**AB014877**	**10886918**	**2.17**	**56**	**52.6**	**turq**	
	**---**	**10862453**	**2.54**	**213**	**26.9**	**blue**	
	---	10788099	0.62	-12	23.1	blue	

	---	10939699	1.66	31	3.7	blue	
	---	10823419	0.49	-39	0	grey	
	---	10875062	0.61	-24	0	grey	
	---	10800513	0.68	-23	0	grey	
	---	10837617	0.41	-19	0	grey	
	---	10871661	0.41	-15	0	grey	
	---	10922266	0.52	-14	0	grey	
	---	10874328	0.49	-68	22.0	turq	
	---	10812130	0.71	-40	21.8	turq	
	---	10715344	0.43	-48	19.9	turq	
	---	10912629	0.48	-61	16.6	turq	
	---	10811898	1.67	331	14.7	turq	
	---	10854284	0.52	-30	12.9	turq	
	---	10781238	1.68	15	7.1	turq	
	---	10924717	0.71	-16	4.9	turq	
	---	10705102	0.69	-14	1.3	turq	
	---	10750452	1.20	14	1.9	ylw	
	**---**	**10700398**	**1.75**	**333**	**33.8**	**blue**	
	---	10701605	2.80	897	18.4	blue	
	---	10700525	2.18	1805	17.1	blue	
	---	10700873	1.99	1969	16.2	blue	
	---	10700831	1.74	61	15.2	blue	
	---	10701404	1.28	880	15.0	blue	
	---	10700423	1.96	58	11.1	blue	
	---	10701507	1.99	58	10.4	blue	
	---	10700415	2.06	50	10.2	blue	
	---	10700473	1.53	79	8.5	blue	
	---	10701134	1.64	15	6.8	blue	
	---	10700853	0.60	-15	6.1	blue	
	---	10700448	0.73	-99	5.7	blue	
	---	10700537	2.11	56	5.6	blue	
	---	10701317	0.46	-16	5.5	blue	
	---	10700643	0.54	-18	5.4	blue	
	---	10701346	1.62	15	3.0	blue	
	---	10701369	0.63	-18	2.8	blue	
	---	10700195	0.42	-15	2.15	green	
	---	10700479	0.66	-192	0	grey	
	---	10701044	0.70	-120	0	grey	
	---	10701407	0.52	-27	0	grey	
	---	10701529	0.72	-23	0	grey	
	---	10701444	0.61	-20	0	grey	
	---	10700906	0.48	-17	0	grey	
	---	10700921	0.43	-15	0	grey	
	---	10701027	0.50	-11	0	grey	
	---	10701552	0.45	-11	0	grey	
	---	10701287	0.38	-11	0	grey	
	---	10700773	1.56	10	0	grey	
	---	10701125	1.52	12	0	grey	
	---	10701495	1.94	13	0	grey	
	---	10701304	2.08	14	0	grey	
	---	10700579	1.49	15	0	grey	
	---	10701062	1.73	22	0	grey	
	---	10701563	2.66	38	0	grey	
	---	10700558	2.21	58	0	grey	
	---	10700311	2.12	63	0	grey	
	---	10701432	4.34	70	0	grey	
	---	10700828	2.23	96	0	grey	
	**---**	**10700160**	**0.60**	**-1277**	**42.0**	**turq**	
	---	10700258	0.63	-1178	34.0	turq	
	---	10700880	0.50	-1405	32.1	turq	
	---	10701239	0.44	-361	28.6	turq	
	---	10701313	0.56	-366	27.8	turq	
	---	10700969	0.61	-622	25.5	turq	
	---	10701109	0.51	-216	21.3	turq	
	---	10700438	1.88	119	21.3	turq	
	---	10700806	0.61	-1058	21.2	turq	
	---	10700138	0.65	-170	19.1	turq	
	---	10700929	0.57	-157	18.7	turq	
	---	10701566	0.71	-1150	18.3	turq	

	---	10700299	0.64	-1023	17.6	turq	
	---	10700791	1.63	1593	17.6	turq	
	---	10700279	0.59	-1726	17.5	turq	
	---	10700304	0.73	-763	13.9	turq	
	---	10700451	0.52	-128	13.8	turq	
	---	10700340	0.62	-565	13.8	turq	
	---	10700101	0.33	-213	13.4	turq	
	---	10701390	0.74	-349	13.4	turq	
	---	10701431	0.62	-157	12.4	turq	
	---	10701312	0.58	-1494	11.9	turq	
	---	10701509	1.71	90	9.8	turq	
	---	10700879	0.42	-265	8.7	turq	
	---	10700151	2.38	63	8.0	turq	
	---	10701257	0.34	-89	7.9	turq	
	---	10701022	2.00	12	7.2	turq	
	---	10700759	2.38	44	6.8	turq	
	---	10700637	0.58	-98	5.9	turq	
	---	10701554	0.83	-958	5.6	turq	
	---	10701356	2.50	16	5.0	turq	
	---	10701576	2.16	19	3.5	turq	
	---	10701018	2.15	33	2.9	turq	
	---	10701124	0.75	-956	2.7	turq	
	---	10701525	1.60	27	2.2	turq	
	---	10700812	2.47	25	2.1	ylw	

J. Seminal Vesicles

Gene Symbol	RefSeq/GenBank/ Ensemble Number	Affymetrix Probset ID	V/C	Mean Diff.	k.in	Mod	Gene Title
**APOPTOSIS**							
Dap3	NM_001011950	10824373	0.83	-28	3.9	brwn	death associated protein 3
**CELL CYCLE**							
Ccnl2	NM_001013094	10874708	0.74	-88	6.1	blue	cyclin L2
Egr1	NM_012551	10800919	1.48	14	0	grey	early growth response 1
Sfi1	NM_001191682	10777878	0.76	-15	0	grey	Sfi1 homolog, spindle assembly associated (yeast)
**CYTOSKELETON-ECM**							
Amigo3	NM_178144	10913153	0.68	-12	5.1	brwn	adhesion molecule with Ig like domain 3
Hook2	NM_001100562	10809998	0.76	-17	2.8	brwn	hook homolog 2 (Drosophila)
Krtcap2	NM_001106444	10816760	0.78	-124	18.0	turq	keratinocyte associated protein 2
Repin1	NM_001005893	10855367	1.32	22	2.1	ylw	replication initiator 1
Rpain	NM_001033060	10735400	1.22	30	0	grey	RPA interacting protein
Sdc4	NM_012649	10851599	1.22	151	0	grey	syndecan 4
Vcp	NM_053864	10876224	0.82	-350	36.2	turq	valosin-containing protein
**DEVELOPMENT**							
Fis1	NM_001105919	10757448	1.27	117	0	grey	fission 1 (mitochondrial outer membrane) homolog (S. cerevisiae)
Lmbr1l	NM_001013950	10907062	0.80	-18	0	grey	limb region 1-like homolog (mouse)
Ltv1	NM_001014157	10716884	0.81	-25	0	grey	LTV1 homolog (S. cerevisiae)
Myadm	NM_183332	10718591	1.26	23	7.2	brwn	myeloid-associated differentiation marker
Osr2	NM_001012118	10896108	1.57	12	0	grey	odd-skipped related 2 (Drosophila)
RGD1566325	ENSRNOT00000024149	10796900	1.43	43	10.0	turq	similar to regulator of sex-limitation candidate 16
Rrm2b	NM_001130543	10903391	0.83	-64	8.0	brwn	ribonucleotide reductase M2 B (TP53 inducible)
**Scrib**	**NM_001191879**	**10904714**	**0.78**	**-24**	**38.8**	**turq**	**scribbled homolog (Drosophila)**
Spats2l	NM_001014102	10923381	1.40	24	9.9	blue	spermatogenesis associated, serine-rich 2-like
Zpbp2	NM_001007011	10738015	0.63	-30	0	grey	zona pellucida binding protein 2
**DNA REPAIR**							
Cry2	NM_133405	10847525	1.24	57	14.4	turq	cryptochrome 2 (photolyase-like)
**ELECTRON TRANSPORT**							
Cybrd1	NM_001011954	10836788	1.29	15	1.8	brwn	cytochrome b reductase 1
Txnip	NM_001008767	10817552	1.21	376	3.0	turq	thioredoxin interacting protein
**EPIGENETICS**							
Chd8	NM_022933	10783280	0.82	-21	0	grey	chromodomain helicase DNA binding protein 8
Ddx5	NM_001007613	10748347	0.81	-435	0	grey	DEAD (Asp-Glu-Ala-Asp) box polypeptide 5
Hist1h2bf	ENSRNOT00000049626	10795282	1.31	12	0	grey	histone cluster 1, H2bf
Hist2h2bb	ENSRNOT00000042005	10817539	1.26	23	3.3	ylw	histone cluster 2, H2bb
**GOLGI APPARATUS**							
Cog4	NM_001108449	10808014	0.73	-99	8.7	brwn	component of oligomeric golgi complex 4
Dpm1	NM_001106544	10851997	0.83	-100	0.7	pink	dolichyl-phosphate mannosyltransferase polypeptide 1, catalytic subunit
Golga5	NM_001033065	10886465	0.80	-46	14.2	brwn	golgi autoantigen, golgin subfamily a, 5
Golgb1	NM_138885	10754311	0.78	-30	1.8	brwn	golgi autoantigen, golgin subfamily b, macrogolgin 1

Uso1	NM_019379	10775821	0.79	-169	9.6	brwn	USO1 homolog, vesicle docking protein (yeast)
**GROWTH FACTORS**							
**Igf2r**	**NM_012756**	**10702996**	**0.76**	**-101**	**46.4**	**turq**	**insulin-like growth factor 2 receptor**
**IMMUNE RESPONSE**							
C3ar1	NM_032060	10865369	1.43	17	16.9	brwn	complement component 3a receptor 1
Cd68	NM_001031638	10744127	1.59	52	19.9	brwn	Cd68 molecule
F5	NM_001047878	10765212	1.37	11	0	grey	coagulation factor V (proaccelerin, labile factor)
**METABOLISM & TRANSPORT**							
Abhd5	NM_212524	10914066	1.34	122	9.0	brwn	abhydrolase domain containing 5
Acbd4	NM_001012013	10738657	1.27	16	1.5	brwn	acyl-Coenzyme A binding domain containing 4
Agpat9	NM_001025670	10775519	1.67	55	7.6	brwn	1-acylglycerol-3-phosphate O-acyltransferase 9
Aox3	NM_001008527	10923432	1.46	104	5.4	brwn	aldehyde oxidase 3
Asns	NM_013079	10860951	0.83	-28	0	grey	asparagine synthetase
Atad3a	NM_001034922	10882201	0.78	-11	2.0	brwn	ATPase family, AAA domain containing 3A
Atp5sl	NM_001009705	10705192	1.22	33	3.5	blue	ATP5S-like
Atpif1	NM_012915	10872692	1.25	318	0	grey	ATPase inhibitory factor 1
Blvrb	NM_001106236	10705358	1.45	19	0	grey	biliverdin reductase B (flavin reductase (NADPH))
Clcn5	NM_017106	10932584	0.80	-22	0	grey	chloride channel 5
Cygb	NM_130744	10749325	1.42	42	7.2	brwn	cytoglobin
Edem3	NM_001191671	10764678	0.82	-77	7.0	brwn	ER degradation enhancer, mannosidase alpha-like 3
Extl2	NM_001100704	10818590	1.30	18	2.8	brwn	exostoses (multiple)-like 2
Farsb	NM_001004252	10929243	0.81	-78	1.2	brwn	phenylalanyl-tRNA synthetase, beta subunit
Gcdh	NM_001108896	10806628	0.71	-214	0	grey	glutaryl-Coenzyme A dehydrogenase
**Gcn1l1**	**NM_001168664**	**10762600**	**0.79**	**-53**	**42.5**	**turq**	**GCN1 general control of amino-acid synthesis 1-like 1 (yeast)**
Gls2	NM_138904	10893008	0.79	-19	0	grey	glutaminase 2 (liver, mitochondrial)
Gstm1	NM_017014	10825925	1.46	538	6.7	turq	glutathione S-transferase mu 1
Herc2	NM_001107520	10707455	0.80	-42	24.2	turq	hect domain and RLD 2
Kcnj16	NM_053314	10739351	1.87	21	0	grey	potassium inwardly-rectifying channel, subfamily J, member 16
Lipe	NM_012859	10719829	1.57	64	8.4	brwn	lipase, hormone sensitive
Lyz2	NM_012771	10902547	1.53	222	0	grey	lysozyme 2
Nars	NM_001025635	10804889	0.79	-93	9.3	brwn	asparaginyl-tRNA synthetase
P4ha2	NM_001108275	10733564	0.70	-37	2.5	turq	prolyl 4-hydroxylase, alpha polypeptide II
Pdia4	NM_053849	10862361	0.82	-143	4.4	brwn	protein disulfide isomerase family A, member 4
Phgdh	NM_031620	10825328	0.79	-20	0	grey	phosphoglycerate dehydrogenase
Plscr3	NM_001012139	10735027	1.24	17	0	grey	phospholipid scramblase 3
Rfk	NM_001014106	10714249	1.34	198	0	grey	riboflavin kinase (chr1)
Rfk	NM_001014106	10847718	1.34	209	0	grey	riboflavin kinase (chr3)
Scn1a	NM_030875	10845859	0.22	-34	3.6	brwn	sodium channel, voltage-gated, type I, alpha
Sec23b	NM_001108593	10840396	0.82	-71	5.5	brwn	Sec23 homolog B (S. cerevisiae)
Sepw1	NM_013027	10719212	1.26	17	0	grey	selenoprotein W, 1
Slc15a3	NM_139341	10713974	1.29	17	4.6	ylw	solute carrier family 15, member 3
Slc1a4	NM_198763	10778620	0.82	-15	0	grey	solute carrier family 1 (glutamate/neutral amino acid transporter), member 4
Slc25a19	NM_001007674	10748999	1.37	17	3.3	brwn	solute carrier family 25 (mitochondrial thiamine pyrophosphate carrier), member 19
Slc26a2	NM_057127	10804672	1.28	35	8.3	brwn	solute carrier family 26 (sulfate transporter), member 2
Slc38a4	NM_130748	10906626	1.41	15	1.9	brwn	solute carrier family 38, member 4
Slc43a1	NM_001107742	10837351	1.75	25	5.3	brwn	solute carrier family 43, member 1
Slc6a6	NM_017206	10857314	1.45	128	10.1	brwn	solute carrier family 6 (neurotransmitter transporter, taurine), member 6
Slc9a3r1	NM_021594	10739490	1.36	131	5.0	turq	solute carrier family 9 (sodium/hydrogen exchanger), member 3 regulator 1
Stard4	NM_001106159	10803681	0.72	-22	4.4	brwn	StAR-related lipid transfer (START) domain containing 4
**PROTEOLYSIS**							
LOC363188	NM_001178071	10921462	0.81	-67	21.8	turq	similar to ubiquitin protein ligase E3 component n-recognin 2
Rnf166	NM_001002279	10811589	1.21	25	2.8	brwn	ring finger protein 166
Uba5	NM_001009669	10919699	0.81	-113	19.0	turq	ubiquitin-like modifier activating enzyme 5
Ubc	NM_017314	10758134	0.73	-276	21.2	turq	ubiquitin C
**RECEPTORS & BINDING PROTEINS**							
Igfbp3	NM_012588	10778390	1.71	84	10.1	brwn	insulin-like growth factor binding protein 3
Ldlr	NM_175762	10908521	0.62	-89	14.0	turq	low density lipoprotein receptor
Ms4a11	ENSRNOT00000033795	10714020	1.40	24	14.9	turq	membrane-spanning 4-domains, subfamily A, member 11
Ms4a6b	NM_001006975	10728924	1.48	19	17.6	turq	membrane-spanning 4-domains, subfamily A, member 6B
Olr1448	NM_001000019	10733888	0.77	-37	0	grey	olfactory receptor 1448
Sorl1	NM_053519	10916493	0.78	-34	22.2	turq	sortilin-related receptor, LDLR class A repeats-containing
Tax1bp1	NM_001004199	10855576	0.83	-156	13.6	brwn	Tax1 (human T-cell leukemia virus type I) binding protein 1
**SIGNALING**							
Akap13	NM_001106271	10707953	0.80	-61	0	grey	A kinase (PRKA) anchor protein 13
Alox5ap	NM_017260	10759999	1.42	21	0	grey	arachidonate 5-lipoxygenase activating protein
Dusp18	NM_001013128	10773754	1.28	21	3.3	brwn	dual specificity phosphatase 18
Ficd	NM_001010946	10762893	0.75	-18	16.4	brwn	FIC domain containing
Hspa5	NM_013083	10835692	0.78	-847	20.3	turq	heat shock protein 5

Hyou1	NM_138867	10909482	0.76	-169	35.3	turq	hypoxia up-regulated 1
Isg15	NM_001106700	10882317	1.50	16	2.6	turq	ISG15 ubiquitin-like modifier
Pde3b	NM_017229	10710089	1.23	70	0	grey	phosphodiesterase 3B, cGMP-inhibited
Pdk2	NM_030872	10746469	1.29	170	13.4	brwn	pyruvate dehydrogenase kinase, isozyme 2
Rab34	NM_001012140	10745202	1.42	62	0	grey	RAB34, member RAS oncogene family
Rhob	NM_022542	10889177	1.23	40	0	grey	ras homolog gene family, member B
Rhog	NM_001037195	10724208	1.27	62	6.4	turq	ras homolog gene family, member G (rho G)
Rhpn2	NM_001107505	10706146	0.68	-23	10.8	brwn	rhophilin, Rho GTPase binding protein 2
Spry2	NM_001012046	10785773	1.28	15	1.7	brwn	sprouty homolog 2 (Drosophila)
Srp54a	NM_053871	10884623	0.80	-278	10.8	brwn	signal recognition particle 54a
Tbccd1	NM_001012016	10751993	0.78	-56	0	grey	TBCC domain containing 1
**TRANSCRIPTION**							
Atf3	NM_012912	10770710	1.42	11	3.7	blue	activating transcription factor 3
Bcas3	NM_001173430	10737075	1.23	31	1.7	ylw	breast carcinoma amplified sequence 3
Bcl7b	NM_001109021	10761220	1.23	44	3.5	turq	B-cell CLL/lymphoma 7B
Bhlhe40	NM_053328	10857610	1.44	112	2.8	brwn	basic helix-loop-helix family, member e40
Bhlhe41	AF009329	10867026	1.94	49	2.2	brwn	basic helix-loop-helix family, member e41
Casc4	ENSRNOT00000044158	10839197	1.35	26	3.3	turq	cancer susceptibility candidate 4
Dnajb11	NM_001015021	10755171	0.81	-156	20.3	turq	DnaJ (Hsp40) homolog, subfamily B, member 11
Gtf2a1	NM_022208	10891436	0.82	-63	0	grey	general transcription factor IIA, 1
Ikzf5	NM_001107555	10726251	1.24	79	0	grey	IKAROS family zinc finger 5
Ino80e	NM_001013900	10725846	1.35	48	0	grey	INO80 complex subunit E
LOC680290	XM_002728448	10796912	1.61	34	0	grey	similar to Zinc finger protein 208
Maf	NM_019318	10811278	1.29	35	9.4	brwn	v-maf musculoaponeurotic fibrosarcoma oncogene homolog (avian)
**Mdm4**	**NM_001012026**	**10767663**	**0.81**	**-78**	**25.1**	**blue**	**Mdm4 p53 binding protein homolog (mouse)**
Nfrkb	NM_001108133	10908833	0.79	-29	0	grey	nuclear factor related to kappa B binding protein
Nfxl1	NM_001170438	10772472	0.80	-27	11.2	blue	nuclear transcription factor, X-box binding-like 1
Nr1d1	NM_001113422	10746955	1.75	109	13.6	brwn	nuclear receptor subfamily 1, group D, member 1
Pir	NM_001009474	10937734	1.42	43	5.4	turq	pirin (iron-binding nuclear protein)
Poli	NM_001106137	10805111	1.28	19	11.0	brwn	polymerase (DNA directed), iota
Rela	NM_199267	10713089	1.28	50	6.7	turq	v-rel reticuloendotheliosis viral oncogene homolog A (avian)
Sdf2l1	NM_001109433	10752531	0.69	-151	19.2	brwn	stromal cell-derived factor 2-like 1
Spdef	NM_001109530	10831880	0.63	-22	12.3	brwn	SAM pointed domain containing ets transcription factor
Tef	NM_019194	10897997	1.40	52	0	grey	thyrotrophic embryonic factor
Tob2	NM_001007146	10905645	1.26	21	0.6	mgnt	transducer of ERBB2, 2
Tsc22d3	NM_031345	10939516	1.31	48	0	grey	TSC22 domain family, member 3
Zbtb34	ENSRNOT00000051893	10844502	0.77	-17	0	grey	zinc finger and BTB domain containing 34
Zfp36	NM_133290	10720215	1.29	35	10.7	turq	zinc finger protein 36
Zgpat	NM_001009656	10843013	0.82	-13	0	grey	zinc finger, CCCH-type with G patch domain
**TRANSLATION & PROTEIN MODIFICATION**							
Arfgap1	NM_145090	10842903	0.74	-14	10.4	brwn	ADP-ribosylation factor GTPase activating protein 1
Cars	NM_001106319	10727084	0.80	-49	14.0	brwn	cysteinyl-tRNA synthetase
Clp1	NM_001009599	10846846	1.23	46	4.1	turq	CLP1, cleavage and polyadenylation factor I subunit, homolog (S. cerevisiae)
Ddx51	NM_001107150	10762985	0.64	-15	8.6	turq	DEAD (Asp-Glu-Ala-Asp) box polypeptide 51
Dhx36	NM_001107678	10823386	0.81	-86	2.0	brwn	DEAH (Asp-Glu-Ala-His) box polypeptide 36
Eif1	NM_001105837	10738170	0.82	-20	2.0	brwn	eukaryotic translation initiation factor 1
Eif2b2	NM_032058	10886014	0.82	-37	2.2	brwn	eukaryotic translation initiation factor 2B, subunit 2 beta
Eif3c	NM_001100662	10725724	0.82	-139	8.6	brwn	eukaryotic translation initiation factor 3, subunit C
Gle1	NM_001025731	10835037	0.78	-14	0.3	ylw	GLE1 RNA export mediator homolog (yeast)
Hars	NM_001025414	10804003	0.80	-61	11.9	brwn	histidyl-tRNA synthetase
Lars	NM_001009637	10804187	0.77	-115	8.1	brwn	leucyl-tRNA synthetase
Mrpl42	NM_001106782	10901999	1.32	253	0	grey	mitochondrial ribosomal protein L42
Prpf4b	NM_001011923	10798074	0.78	-62	1.2	pink	PRP4 pre-mRNA processing factor 4 homolog B (yeast)
Qtrtd1	ENSRNOT00000000317	10751196	0.77	-12	0	grey	queuine tRNA-ribosyltransferase domain containing 1
RGD1563835	ENSRNOT00000042756	10751237	0.71	-59	4.4	brwn	similar to ribosomal protein L27
Rnaset2	NM_001106210	10703139	0.75	-145	8.3	brwn	ribonuclease T2
Sars	NM_001007606	10826039	0.80	-126	14.6	brwn	seryl-tRNA synthetase
Sfrs5	NM_001195505	10885672	0.75	-282	1.2	pink	splicing factor, arginine/serine-rich 5
Yars	NM_001025696	10872291	0.80	-70	21.3	turq	tyrosyl-tRNA synthetase
**MISCELLANEOUS & UNKNOWN**							
Cuedc2	NM_001079886	10730559	1.28	15	3.4	brwn	CUE domain containing 2
Fam110c	NM_001025051	10884075	1.39	12	0	grey	family with sequence similarity 110, member C
Fam115c	ENSRNOT00000050392	10862301	0.78	-12	0	grey	family with sequence similarity 115, member C
Gramd1a	NM_001014160	10720918	0.80	-26	10.8	turq	GRAM domain containing 1A
LOC100360342	ENSRNOT00000021923	10882829	0.81	-23	6.5	brwn	rCG61559-like
LOC100361593	XM_002729223	10840365	0.83	-39	0	grey	hypothetical protein LOC100361593
LOC500420	BC089864	10875751	1.47	52	7.4	turq	similar to CG12279-PA
LOC680761	ENSRNOT00000043552	10708832	0.83	-38	0.8	brwn	hypothetical protein LOC680761
Mir671	NR_032287	10859988	0.68	-37	12.8	brwn	microRNA mir-671

RGD1303127	NM_001004244	10906863	1.26	81	1.0	turq	similar to hypothetical protein FLJ20436
RGD1308059	BC091300	10869656	0.72	-17	6.8	brwn	similar to DNA segment, Chr 4, Brigham & Womens Genetics 0951 expressed
RGD1309621	ENSRNOT00000055437	10859774	1.24	58	5.6	turq	similar to hypothetical protein FLJ10652
RGD1309995	ENSRNOT00000011326	10901367	0.82	-142	6.4	brwn	similar to CG13957-PA
RGD1559575	ENSRNOT00000046438	10742352	1.60	12	12.4	turq	similar to novel protein
RGD1560812	ENSRNOT00000065561	10884158	1.43	15	8.7	brwn	RGD1560812
RGD1564171	NM_001109186	10816879	0.74	-18	0	grey	RGD1564171
Tmem56	NM_001135879	10826442	0.77	-69	1.6	brwn	transmembrane protein 56
Trim39	NM_213562	10827741	0.76	-11	0	grey	tripartite motif-containing 39
Wdr33	NM_001107398	10800672	0.83	-26	3.8	turq	WD repeat domain 33
**EST's**							
RGD1305469	ENSRNOT00000039277	10773638	0.83	-26	4.7	brwn	similar to RIKEN cDNA 9030221M09 gene
RGD1308114	BC079348	10795444	1.63	41	6.1	brwn	similar to cDNA sequence AF397014
RGD1309308	NM_001126279	10875795	0.78	-166	8.4	brwn	similar to RIKEN cDNA 1810074P20
RGD1560108	NM_001109161	10713518	1.33	14	10.3	brwn	similar to RIKEN cDNA 2700081O15
Mir186	NR_031904	10819903	0.82	-11	2.1	blue	microRNA mir-186
	NC_001665	10930593	0.58	-1377	8.3	blue	
	GENSCAN00000034333	10781960	0.57	-30	0	grey	
	GENSCAN00000030433	10932610	0.77	-54	6.9	turq	
	GENSCAN00000025175	10743100	0.81	-12	0	grey	
	FQ227520	10851813	0.81	-35	10.5	turq	
	FQ222426	10842433	1.27	16	0	grey	
	**FQ217081**	**10814142**	**1.24**	**53**	**38.6**	**turq**	
	FQ212372	10891830	1.43	64	8.1	ylw	
	ENSRNOT00000068215	10771201	1.37	11	0	grey	
	ENSRNOT00000063495	10892977	0.68	-18	0.7	pink	
	ENSRNOT00000061085	10779510	1.45	14	9.9	brwn	
	ENSRNOT00000059335	10884765	2.30	58	0	grey	
	ENSRNOT00000057455	10934659	0.77	-11	0	grey	
	ENSRNOT00000053987	10755127	0.71	-38	0	grey	
	ENSRNOT00000053274	10754642	0.80	-12	10.5	blue	
	ENSRNOT00000052462	10802708	0.67	-15	1.0	brwn	
	**ENSRNOT00000046636**	**10779529**	**1.41**	**10**	**43.4**	**blue**	
	ENSRNOT00000046555	10892346	0.77	-17	3.8	blue	
	ENSRNOT00000035259	10832838	1.22	80	2.0	ylw	
	ENSRNOT00000032056	10770924	0.79	-16	0	grey	
	ENSRNOT00000029093	10884738	0.79	-33	8.0	brwn	
	ENSRNOT00000025383	10920986	0.67	-13	3.9	blue	
	ENSRNOT00000021212	10836015	0.78	-159	9.8	brwn	
	ENSRNOT00000015111	10766302	1.23	14	20.8	blue	
	ENSRNOT00000011530	10891878	0.75	-16	2.2	ylw	
	ENSRNOT00000010282	10812881	0.74	-46	3.0	ylw	
	BC158824	10861130	1.37	183	0	grey	
	BC105775	10878999	1.23	14	4.1	blue	
	---	10708589	0.68	-111	4.7	blue	
	---	10761285	1.21	10	0	grey	
	---	10793066	1.28	13	0	grey	
	---	10700831	0.55	-65	15.2	blue	
	---	10701502	0.64	-35	11.2	blue	
	---	10701273	0.53	-19	6.1	blue	
	---	10701243	0.66	-33	5.8	blue	
	---	10700448	1.23	65	5.7	blue	
	---	10700735	0.73	-43	5.7	blue	
	---	10700433	0.58	-25	2.7	blue	
	---	10700469	0.71	-543	1.5	blue	
	---	10700216	2.13	22	1.2	blue	
	---	10700904	0.80	-375	8.9	brwn	
	---	10700786	0.76	-230	8.5	brwn	
	---	10701084	0.82	-280	7.9	brwn	
	---	10700113	0.69	-292	6.6	brwn	
	---	10700653	0.40	-28	1.4	brwn	
	---	10700937	0.80	-351	0	grey	
	---	10700857	0.70	-74	0	grey	
	---	10701035	0.78	-46	0	grey	
	---	10700471	0.66	-40	0	grey	
	---	10700196	0.70	-30	0	grey	
	---	10701458	0.59	-28	0	grey	
	---	10701002	0.52	-25	0	grey	
	---	10700221	0.61	-24	0	grey	

	---	10701395	0.51	-23	0	grey	
	---	10701210	0.50	-18	0	grey	
	---	10701580	0.57	-17	0	grey	
	---	10701565	0.70	-17	0	grey	
	---	10700712	0.68	-15	0	grey	
	---	10700088	0.70	-14	0	grey	
	---	10701001	0.74	-14	0	grey	
	---	10701249	0.51	-13	0	grey	
	---	10700962	0.39	-12	0	grey	
	---	10700246	0.46	-11	0	grey	
	---	10701388	1.69	10	0	grey	
	---	10700638	1.49	16	0	grey	
	---	10700128	2.07	16	0	grey	
	---	10701130	1.41	17	0	grey	
	---	10701259	1.46	35	0	grey	
	---	10700922	1.22	60	0	grey	
	---	10701618	1.34	100	0	grey	
	---	10701477	0.61	-19	0.8	pink	
	---	10700731	0.59	-17	0.7	pink	
	---	10701009	0.74	-50	0.4	pink	
	**---**	**10700160**	**0.76**	**-690**	**42.0**	**turq**	
	---	10700258	0.77	-651	34.0	turq	
	---	10700880	0.79	-463	32.1	turq	
	---	10700799	0.81	-11	28.3	turq	
	---	10700959	0.83	-399	23.8	turq	
	---	10700736	0.79	-391	22.4	turq	
	---	10701198	0.78	-500	19.1	turq	
	---	10700938	0.82	-269	18.4	turq	
	---	10700299	0.79	-526	17.6	turq	
	---	10700270	0.82	-260	16.6	turq	
	---	10700151	0.72	-23	8.0	turq	
	---	10701154	0.70	-32	2.7	turq	
	---	10700475	0.68	-11	1.0	ylw	

K. Testis

Gene Symbol	RefSeq/GenBank/ Ensemble Number	Affymetrix Probset ID	V/C	Mean Diff.	k.in	Mod	Gene Title
**APOPTOSIS**							
Eaf1	NM_001107293	10786703	1.25	20	1.7	turq	ELL associated factor 1
Pdcd11	NM_001107604	10715916	1.24	26	0	grey	programmed cell death 11
Tfg	NM_001012144	10750781	1.21	140	24.8	turq	Trk-fused gene
Wdfy3	NM_001170551	10771287	1.25	32	5.7	ylw	WD repeat and FYVE domain containing 3
**CELL CYCLE**							
Ccndbp1	NM_001013204	10839090	0.76	-56	22.9	ylw	cyclin D-type binding-protein 1
Nubp1	NM_001009619	10740466	0.77	-63	11.6	blue	nucleotide binding protein 1
Terc	NR_001567	10814703	0.56	-24	29.7	ylw	telomerase RNA component
**CYTOSKELETON-ECM**							
Cdon	NM_017358	10908968	1.23	16	18.4	brwn	Cdon homolog (mouse)
Eml5	ENSRNOT00000057601	10891559	1.42	22	6.5	brwn	echinoderm microtubule associated protein like 5
Lman1	NM_053886	10802347	1.23	146	17.3	turq	lectin, mannose-binding, 1
Lmnb1	NM_053905	10801821	1.21	13	0	grey	lamin B1
Map4	NM_001024278	10913570	1.37	127	19.1	brwn	microtubule-associated protein 4
Parva	NM_020656	10709993	1.29	62	4.6	ylw	parvin, alpha
Pxn	NM_001012147	10762665	1.24	48	11.3	turq	paxillin
Tppp2	NM_001109100	10779868	0.59	-17	2.23	green	tubulin polymerization-promoting protein family member 2
**DEVELOPMENT**							
Asap1	NM_001044245	10904031	1.25	37	13.5	brwn	ArfGAP with SH3 domain, ankyrin repeat and PH domain 1
Ccrn4l	NM_138526	10815281	1.36	11	0	grey	CCR4 carbon catabolite repression 4-like (S. cerevisiae)
Cdan1	NM_001107765	10848860	1.20	13	0	grey	congenital dyserythropoietic anemia, type I
Cirh1a	NM_001009640	10807583	1.26	77	2.7	ylw	cirrhosis, autosomal recessive 1A
Dctpp1	NM_138892	10725989	0.73	-11	0	grey	dCTP pyrophosphatase 1 (chr1)
Dctpp1	NM_138892	10789732	0.80	-11	0.8	ylw	dCTP pyrophosphatase 1 (chr16)
Eed	ENSRNOT00000033400	10723623	0.78	-24	11.7	blue	embryonic ectoderm development
Fis1	NM_001105919	10757448	0.79	-114	0	grey	fission 1 (mitochondrial outer membrane) homolog (S. cerevisiae)
Htt	NM_024357	10777571	1.20	21	11.3	turq	huntingtin
Mmachc	NM_001107962	10878905	1.24	80	30.9	turq	methylmalonic aciduria (cobalamin deficiency) cblC type, with homocystinuria
Nenf	NM_001002851	10770714	0.77	-15	17.4	ylw	neuron derived neurotrophic factor
Npdc1	NM_001004231	10834225	1.25	23	11.5	brwn	neural proliferation, differentiation and control, 1

Pcnxl3	ENSRNOT00000064136	10727943	1.40	31	30.3	turq	pecanex-like 3 (Drosophila)
Shfm1	NM_001126090	10860939	0.78	-176	33.5	ylw	split hand/foot malformation (ectrodactyly) type 1
Slit3	NM_031321	10732750	1.27	12	12.4	brwn	slit homolog 3 (Drosophila)
**Tcta**	**NM_001014005**	**10793218**	**0.83**	**-29**	**27.8**	**brwn**	**T-cell leukemia translocation altered gene**
Tesk1	NM_031578	10868494	1.22	34	3.5	ylw	testis-specific kinase 1
Urb2	NM_001135708	10808764	1.25	18	1.1	ylw	URB2 ribosome biogenesis 2 homolog (S. cerevisiae)
Vamp1	NM_013090	10858858	1.32	16	0.15	green	vesicle-associated membrane protein 1
Vamp8	NM_031827	10863218	0.74	-96	7.6	ylw	vesicle-associated membrane protein 8
Wdtc1	NM_001107908	10872585	1.20	57	4.5	ylw	WD and tetratricopeptide repeats 1
**ELECTRON TRANSPORT**							
Cox7a2	NM_022503	10772901	0.79	-629	39.6	ylw	cytochrome c oxidase subunit VIIa polypeptide 2 (chr14)
Cox7a2	NM_022503	10918940	0.80	-643	41.3	ylw	cytochrome c oxidase subunit VIIa polypeptide 2 (chr8)
Fdx1	NM_017126	10917346	0.82	-35	11.8	ylw	ferredoxin 1
Gpx1	NM_030826	10793103	0.78	-442	15.4	blue	glutathione peroxidase 1
LOC685322	NM_001170465	10778108	0.82	-111	38.9	ylw	similar to ubiquinol-cytochrome c reductase complex 7.2kDa protein isoform b
**Prdx2**	**NM_017169**	**10810018**	**0.80**	**-177**	**28.4**	**brwn**	**peroxiredoxin 2**
Prdx4	NM_053512	10933699	0.77	-41	7.4	turq	peroxiredoxin 4
**EPIGENETICS**							
Chrac1	NM_001134880	10896957	0.76	-21	27.9	ylw	chromatin accessibility complex 1
Dot1l	NM_001108733	10900419	1.38	18	5.6	blue	DOT1-like, histone H3 methyltransferase (S. cerevisiae)
Ep400	NM_001107149	10759287	1.21	27	11.1	ylw	E1A binding protein p400
H1f0	NM_012578	10897543	0.81	-65	9.5	blue	H1 histone family, member 0
Jmjd1c	NM_001191719	10832829	1.23	60	15.6	brwn	jumonji domain containing 1C
LOC100365770	XM_002727530	10936923	0.73	-15	0	grey	histone variant H2al2-like
LOC685909	NM_001106019	10805582	0.83	-38	19.4	ylw	similar to H2A histone family, member V isoform 1
Mettl11a	NM_001025019	10835252	0.83	-14	8.5	brwn	methyltransferase like 11A
RGD1564767	NM_001024282	10795196	0.77	-23	0	grey	Histone H2a
Scmh1	NM_001109669	10871629	1.37	26	6.4	brwn	sex comb on midleg homolog 1 (Drosophila)
Smarca5	NM_001107419	10806937	1.24	85	5.8	ylw	SWI/SNF related, matrix associated, actin dependent regulator of chromatin, subfamily a, member 5
**GOLGI APPARATUS**							
Ergic3	NM_001106533	10841452	0.83	-140	9.1	turq	ERGIC and golgi 3
Gcnt1	NM_022276	10729209	0.71	-18	0.2	black	glucosaminyl (N-acetyl) transferase 1, core 2 (beta-1,6-N-acetylglucosaminyltransferase)
Mgat4a	NM_001160155	10927472	0.79	-28	0	grey	mannosyl (alpha-1,3-)-glycoprotein beta-1,4-N-acetylglucosaminyltransferase, isozyme A
Use1	NM_001169100	10787256	0.72	-419	29.8	ylw	unconventional SNARE in the ER 1 homolog (S. cerevisiae)
**GROWTH FACTORS**							
Ik	NM_001005537	10801086	1.26	176	33.5	ylw	IK cytokine
Lass2	NM_001033700	10817383	1.31	152	28.0	turq	LAG1 homolog, ceramide synthase 2
**IMMUNE RESPONSE**							
C4bpb	NM_016995	10767436	1.27	14	2.9	blue	complement component 4 binding protein, beta
Lkap	NM_133421	10731291	1.30	135	14.7	ylw	limkain b1
LOC100361139	ENSRNOT00000057140	10939402	0.66	-14	0	grey	thymosin-like 8-like
LOC100364751	XM_002726462	10866142	0.75	-24	12.3	turq	immunoreceptor Ly49si3-like
Ly49si2	NM_001009498	10866109	0.57	-41	25.0	turq	immunoreceptor Ly49si2
Tsta3	NM_001127455	10904688	0.80	-110	42.5	ylw	tissue specific transplantation antigen P35B
**METABOLISM & TRANSPORT**							
Acads	NM_022512	10759034	0.74	-47	0	grey	acyl-Coenzyme A dehydrogenase, C-2 to C-3 short chain
Agpat1	NM_212458	10831452	1.31	112	27.8	ylw	1-acylglycerol-3-phosphate O-acyltransferase 1 (lysophosphatidic acid acyltransferase, alpha)
Atp11a	NM_001107324	10792859	1.30	70	2.5	ylw	ATPase, class VI, type 11A
**Atp5e**	**BC058133**	**10773636**	**0.66**	**-416**	**58.2**	**ylw**	**ATP synthase, H+ transporting, mitochondrial F1 complex, epsilon subunit**
**Atp5j2**	**ENSRNOT00000033537**	**10756546**	**0.81**	**-517**	**49.2**	**ylw**	**ATP synthase, H+ transporting, mitochondrial F0 complex, subunit F2**
**Atp5l**	**NM_212516**	**10916920**	**0.69**	**-62**	**43.9**	**ylw**	**ATP synthase, H+ transporting, mitochondrial F0 complex, subunit G**
Atp6v1e1	NM_198745	10865212	0.82	-134	12.2	brwn	ATPase, H+ transporting, lysosomal V1 subunit E1
Bckdha	NM_012782	10719863	0.79	-110	0	grey	branched chain ketoacid dehydrogenase E1, alpha polypeptide
Bdh1	NM_053995	10754931	0.83	-55	0	grey	3-hydroxybutyrate dehydrogenase, type 1
Bdh2	NM_001106473	10819192	0.78	-13	0	grey	3-hydroxybutyrate dehydrogenase, type 2
Bloc1s1	NM_001105941	10899959	0.82	-41	3.5	ylw	biogenesis of lysosomal organelles complex-1, subunit 1
Carkd	NM_001108402	10792943	0.79	-23	14.6	brwn	carbohydrate kinase domain containing
**Cdipt**	**NM_138899**	**10710985**	**1.30**	**113**	**47.3**	**turq**	**CDP-diacylglycerol--inositol 3-phosphatidyltransferase (phosphatidylinositol synthase)**
Cox4nb	NM_001012165	10811493	0.81	-16	10.5	blue	COX4 neighbor
Decr1	NM_057197	10875631	0.72	-229	0	grey	2,4-dienoyl CoA reductase 1, mitochondrial
Dhrs1	NM_001007621	10783917	1.29	35	6.1	turq	dehydrogenase/reductase (SDR family) member 1
Dhrs7b	NM_001008507	10734190	0.83	-26	10.9	ylw	dehydrogenase/reductase (SDR family) member 7B
Echs1	NM_078623	10726611	0.82	-159	0	grey	enoyl Coenzyme A hydratase, short chain, 1, mitochondrial
Fa2h	NM_001135583	10811126	1.31	12	1.6	red	fatty acid 2-hydroxylase

Gsta2	NM_017013	10911802	0.75	-47	0	grey	glutathione S-transferase A2
Hmbs	NM_013168	10916753	0.77	-78	18.6	brwn	hydroxymethylbilane synthase
Hmgcl	NM_024386	10872949	0.79	-54	14.2	brwn	3-hydroxymethyl-3-methylglutaryl-Coenzyme A lyase
Hpgd	NM_024390	10791522	0.82	-12	0.4	ylw	hydroxyprostaglandin dehydrogenase 15 (NAD)
Hsd17b10	NM_031682	10933130	0.79	-157	2.6	brwn	hydroxysteroid (17-beta) dehydrogenase 10
Inppl1	NM_022944	10724080	1.21	27	28.4	turq	inositol polyphosphate phosphatase-like 1
**Kctd14**	**ENSRNOT00000016654**	**10708782**	**0.81**	**-11**	**28.4**	**brwn**	**potassium channel tetramerisation domain containing 14**
**LOC100360067**	**ENSRNOT00000047723**	**10927610**	**0.69**	**-20**	**47.3**	**ylw**	**SEC61, gamma subunit-like**
LOC500959	NM_001033072	10915837	0.80	-389	16.3	turq	triosephosphate isomerase
LOC687295	ENSRNOT00000057096	10720161	0.76	-68	0	grey	similar to translocase of inner mitochondrial membrane 50 homolog
Mlph	NM_001012135	10925303	0.82	-11	0	grey	melanophilin
Ndor1	NM_001107818	10843380	1.29	27	22.4	turq	NADPH dependent diflavin oxidoreductase 1
Ndst2	NM_001105740	10779054	1.28	25	2.1	ylw	N-deacetylase/N-sulfotransferase (heparan glucosaminyl) 2
Ndufa1	NM_001108813	10931991	0.82	-104	22.5	ylw	NADH dehydrogenase (ubiquinone) 1 alpha subcomplex, 1
Ndufa2	NM_001106153	10803995	0.82	-25	31.2	ylw	NADH dehydrogenase (ubiquinone) 1 alpha subcomplex, 2
Ndufa3	ENSRNOT00000019141	10718552	0.82	-108	3.0	brwn	NADH dehydrogenase (ubiquinone) 1 alpha subcomplex, 3
Ndufa8	NM_001047862	10844726	0.82	-90	18.7	brwn	NADH dehydrogenase (ubiquinone) 1 alpha subcomplex, 8
Ndufb8	NM_001106360	10730383	0.75	-137	20.1	ylw	NADH dehydrogenase (ubiquinone) 1 beta subcomplex 8
Ndufs8	NM_001106322	10727398	0.82	-104	24.0	blue	NADH dehydrogenase (ubiquinone) Fe-S protein 8
Nme2	NM_031833	10746271	0.83	-309	22.4	ylw	non-metastatic cells 2, protein (NM23B) expressed in
Nme6	NM_001191884	10913545	0.83	-17	1.2	brwn	non-metastatic cells 6, protein expressed in (nucleoside-diphosphate kinase)
Pcyt2	NM_053568	10749689	0.81	-37	19.6	brwn	phosphate cytidylyltransferase 2, ethanolamine
Pdxk	NM_031769	10829191	0.76	-25	0.2	red	pyridoxal (pyridoxine, vitamin B6) kinase
Pigw	NM_194461	10745782	1.27	35	6.4	brwn	phosphatidylinositol glycan anchor biosynthesis, class W
Pop5	NM_001105752	10762717	0.76	-23	5.8	brwn	processing of precursor 5, ribonuclease P/MRP subunit (S. cerevisiae)
Prr14	ENSRNOT00000055002	10711053	1.22	11	8.2	ylw	proline rich 14
Pusl1	NM_001108699	10882238	0.82	-14	0	grey	pseudouridylate synthase-like 1
Pycr2	NM_001012208	10766240	0.82	-12	12.1	brwn	pyrroline-5-carboxylate reductase family, member 2
Sec14l1	NM_001108309	10739866	1.21	31	1.6	ylw	SEC14-like 1 (S. cerevisiae)
**Sec61g**	**NM_001135020**	**10778555**	**0.67**	**-237**	**47.7**	**ylw**	**SEC61, gamma subunit**
Sepw1	NM_013027	10719212	0.76	-20	0	grey	selenoprotein W, 1
Slc25a35	NM_001109033	10734822	1.26	19	10.8	ylw	solute carrier family 25, member 35
Slc25a42	NM_001127590	10787611	1.24	17	0.9	ylw	solute carrier family 25, member 42
Slc29a2	NM_031738	10712962	1.31	12	6.8	ylw	solute carrier family 29 (nucleoside transporters), member 2
Slc38a3	NM_145776	10920122	1.23	38	1.8	turq	solute carrier family 38, member 3
Slc43a2	NM_001105812	10735959	1.27	37	8.7	brwn	solute carrier family 43, member 2
Slco3a1	NM_177481	10722818	1.24	17	24.5	ylw	solute carrier organic anion transporter family, member 3a1
Smpx	NM_053395	10938060	0.77	-32	0.9	ylw	small muscle protein, X-linked
**St3gal2**	**NM_031695**	**10808034**	**1.24**	**21**	**25.4**	**brwn**	**ST3 beta-galactoside alpha-2,3-sialyltransferase 2**
Timm8a1	NM_053370	10939289	0.77	-59	16.3	ylw	translocase of inner mitochondrial membrane 8 homolog a1 (yeast)
Tomm40b	NM_001083338	10769815	0.77	-17	0	grey	translocase of outer mitochondrial membrane 40 homolog B (yeast)
Xpo6	NM_001011935	10725618	1.25	90	4.3	ylw	exportin 6
**PROTEOLYSIS**							
March2	NM_001034108	10901152	0.83	-67	0	grey	membrane-associated ring finger (C3HC4) 2
March8	NM_001107882	10858133	0.79	-107	0	grey	membrane-associated ring finger (C3HC4) 8
Otub1	NM_001106332	10728377	0.83	-44	16.5	blue	OTU domain, ubiquitin aldehyde binding 1
**Psmb5**	**NM_001105727**	**10783599**	**0.82**	**-173**	**24.6**	**brwn**	**proteasome (prosome, macropain) subunit, beta type 5**
Psme2	NM_017257	10793365	0.79	-146	2.0	ylw	proteasome (prosome, macropain) activator subunit 2
Rad23a	NM_001013190	10806650	1.21	50	3.3	brwn	RAD23 homolog A (S. cerevisiae)
Rnf8	NM_001025727	10828904	1.21	17	30.8	turq	ring finger protein 8
Spink8	NM_001106860	10913538	0.83	-17	0	grey	serine peptidase inhibitor, Kazal type 8
**Tmprss2**	**ENSRNOT00000044101**	**10753458**	**0.82**	**-35**	**21.6**	**brwn**	**transmembrane protease, serine 2**
Tpp1	NM_031357	10724529	1.26	93	16.0	ylw	tripeptidyl peptidase I
Usp21	NM_001127638	10769854	1.34	39	19.0	ylw	ubiquitin specific peptidase 21
Vps37b	NM_001105928	10758344	1.20	15	7.9	turq	vacuolar protein sorting 37 homolog B (S. cerevisiae)
**RECEPTORS & BINDING PROTEINS**							
Aip	NM_172327	10727445	0.77	-27	5.5	blue	aryl-hydrocarbon receptor-interacting protein
Il18bp	NM_053374	10724131	1.35	22	16.4	ylw	interleukin 18 binding protein
Itgb3bp	NM_001013213	10878272	0.79	-16	1.06	green	integrin beta 3 binding protein (beta3-endonexin)
Nptn	NM_019380	10910463	1.25	113	0	grey	neuroplastin
P2ry2	NM_017255	10724042	1.25	27	16.0	turq	purinergic receptor P2Y, G-protein coupled, 2
Pgrmc1	NM_021766	10936341	0.73	-195	18.3	brwn	progesterone receptor membrane component 1
**Plxnb2**	**NM_001108106**	**10906169**	**1.20**	**62**	**48.0**	**turq**	**plexin B2**
**S100a6**	**NM_053485**	**10817065**	**0.71**	**-36**	**44.8**	**ylw**	**S100 calcium binding protein A6**
S100b	NM_013191	10832441	0.72	-12	3.0	brwn	S100 calcium binding protein B
Sec24c	BC168663	10782323	1.28	71	36.3	turq	SEC24 related gene family, member C (S. cerevisiae)
Wbp11	NM_001009661	10866488	1.26	54	7.3	ylw	WW domain binding protein 11
**SIGNALING**							
Aak1	NM_001173450	10857002	1.22	27	14.3	ylw	AP2 associated kinase 1

Arhgap8	NM_001004242	10898268	1.29	32	4.4	brwn	Rho GTPase activating protein 8
Btbd12	ENSRNOT00000033919	10731872	1.35	18	0	grey	BTB (POZ) domain containing 12
**Cdgap**	**NM_001105879**	**10751255**	**1.30**	**15**	**22.0**	**brwn**	**Cdc42 GTPase-activating protein**
Cks1b	NM_001135749	10824513	0.81	-52	11.7	ylw	CDC28 protein kinase regulatory subunit 1B (chr2)
Cks1b	NM_001135749	10878032	0.79	-56	18.0	ylw	CDC28 protein kinase regulatory subunit 1B (chr5)
Dab2ip	NM_138710	10835779	1.25	12	13.6	turq	DAB2 interacting protein
Dbil5	NM_021596	10736064	0.73	-11	6.86	green	diazepam binding inhibitor-like 5
Defb27	NM_001037519	10850818	0.64	-12	0	grey	defensin beta 27
Enpp1	NM_053535	10702250	1.25	111	2.5	ylw	ectonucleotide pyrophosphatase/phosphodiesterase 1
Hspb9	NM_001108835	10738223	0.72	-25	7.31	green	heat shock protein, alpha-crystallin-related, B9
Ipmk	NM_134417	10832680	0.81	-57	23.6	blue	inositol polyphosphate multikinase
Letm2	NM_001012158	10788841	1.20	11	7.8	ylw	leucine zipper-EF-hand containing transmembrane protein 2
LOC100361629	ENSRNOT00000061017	10812779	0.81	-156	0	grey	ERBB2 interacting protein-like
Magmas	NM_001100136	10731783	0.74	-25	16.7	ylw	mitochondria-associated protein involved in granulocyte-macrophage colony-stimulating factor signal transduction
Mapk8ip3	NM_001100673	10741268	1.25	11	13.9	ylw	mitogen-activated protein kinase 8 interacting protein 3
Mapkbp1	NM_001108589	10838927	1.33	12	8.2	ylw	mitogen activated protein kinase binding protein 1
Nek6	NM_182953	10835964	0.81	-12	5.3	brwn	NIMA (never in mitosis gene a)-related kinase 6
Nisch	ENSRNOT00000036910	10790096	1.32	106	12.3	ylw	nischarin
Pdk2	NM_030872	10746469	1.24	146	13.4	brwn	pyruvate dehydrogenase kinase, isozyme 2
Pik3r4	NM_001108777	10912757	1.22	25	17.1	turq	phosphoinositide-3-kinase, regulatory subunit 4
Ppfia1	NM_001106320	10727199	1.22	29	1.7	ylw	protein tyrosine phosphatase, receptor type, f polypeptide (PTPRF), interacting protein (liprin), alpha 1
Ppp1r12a	NM_053890	10895310	1.32	59	3.8	ylw	protein phosphatase 1, regulatory (inhibitor) subunit 12A
Ptprs	NM_019140	10930942	1.28	27	36.1	ylw	protein tyrosine phosphatase, receptor type, S
Rab11b	NM_032617	10901158	1.20	116	8.8	brwn	RAB11B, member RAS oncogene family
Rabl5	NM_001011902	10757442	0.76	-19	5.2	turq	RAB, member RAS oncogene family-like 5
Reps1	NM_001106264	10701880	1.25	23	1.4	ylw	RALBP1 associated Eps domain containing 1
RGD1309362	BC098065	10801973	0.77	-41	9.7	turq	similar to interferon-inducible GTPase
**RGD1562638**	**NM_001100944**	**10788692**	**1.23**	**14**	**####**	**green**	**similar to MAP/microtubule affinity-regulating kinase 3**
Rhoc	NM_001106461	10818108	0.78	-45	0	grey	ras homolog gene family, member C
Rnd2	NM_001010953	10738423	0.79	-13	1.5	ylw	Rho family GTPase 2
Rras2	NM_001013434	10724993	0.79	-70	0	grey	related RAS viral (r-ras) oncogene homolog 2
Snx8	NM_001105912	10756953	0.80	-12	2.2	brwn	sorting nexin 8
Stap2	NM_001025026	10931068	0.81	-12	15.3	brwn	signal transducing adaptor family member 2
Stk36	NM_001173986	10924363	1.31	12	15.0	blue	serine/threonine kinase 36 (fused homolog, Drosophila)
**TRANSCRIPTION**							
Arid1a	NM_001106635	10872533	1.29	39	2.8	ylw	AT rich interactive domain 1A (SWI-like)
Asb13	NM_001108420	10799376	0.81	-39	0	grey	ankyrin repeat and SOCS box-containing 13
Brpf3	NM_001107615	10828791	1.29	11	10.7	ylw	bromodomain and PHD finger containing, 3
C1d	NM_001106021	10774331	0.77	-111	24.7	ylw	nuclear DNA binding protein
Ccdc112	NM_001109124	10804371	1.24	15	0.9	ylw	coiled-coil domain containing 112
Ccdc125	NM_001134761	10812756	0.82	-12	5.9	brwn	coiled-coil domain containing 125
Ccdc142	ENSRNOT00000035329	10856601	1.28	13	0	grey	coiled-coil domain containing 142
Ccdc55	NM_001037189	10736476	1.33	59	10.4	brwn	coiled-coil domain containing 55
Cnot3	NM_001107471	10718520	1.30	26	6.0	turq	CCR4-NOT transcription complex, subunit 3
Cnot4	NM_001037782	10861778	1.21	63	16.3	brwn	CCR4-NOT transcription complex, subunit 4
**Dnlz**	**NR_024073**	**10843693**	**0.83**	**-33**	**22.1**	**brwn**	**DNL-type zinc finger**
Dpf2	NM_001108516	10728046	1.21	66	10.4	brwn	D4, zinc and double PHD fingers family 2
Gtf3c2	NM_001025120	10883125	1.42	53	3.4	ylw	general transcription factor IIIC, polypeptide 2, beta
**Hes6**	**NM_001013179**	**10929835**	**0.83**	**-17**	**22.3**	**brwn**	**hairy and enhancer of split 6 (Drosophila)**
Hint1	NM_001109607	10733676	0.83	-156	23.0	ylw	histidine triad nucleotide binding protein 1 (chr10)
Hint1	NM_001109607	10862834	0.82	-195	25.4	ylw	histidine triad nucleotide binding protein 1 (chr4)
Klf4	NM_053713	10876838	1.27	23	2.7	brwn	Kruppel-like factor 4 (gut)
Klf5	NM_053394	10781829	1.28	11	0	grey	Kruppel-like factor 5
LOC100363361	ENSRNOT00000028553	10824904	1.31	18	5.7	turq	zinc finger protein 687-like
LOC678741	ENSRNOT00000068268	10704467	1.22	15	6.1	ylw	similar to Zinc finger CCCH-type domain containing protein 6
LOC680290	XM_002728448	10796912	0.74	-21	0	grey	similar to Zinc finger protein 208
LOC691280	ENSRNOT00000043813	10881482	0.81	-43	3.9	ylw	similar to Zinc finger protein 180 (HHZ168)
Med20	NM_001013178	10926323	0.79	-25	0	grey	mediator complex subunit 20
Med23	ENSRNOT00000032424	10717201	1.28	37	9.6	ylw	mediator complex subunit 23
Ncoa6	ENSRNOT00000024714	10851004	1.28	23	11.9	ylw	nuclear receptor coactivator 6
Nfatc3	NM_001108447	10807452	1.22	27	1.8	ylw	nuclear factor of activated T-cells, cytoplasmic, calcineurin-dependent 3
Nr2c1	NM_145780	10894974	1.22	29	0	grey	nuclear receptor subfamily 2, group C, member 1
Nudt14	NM_001106760	10892381	0.74	-33	33.3	ylw	nudix (nucleoside diphosphate linked moiety X)-type motif 14
Nxf1	NM_021579	10713626	1.30	144	20.6	ylw	nuclear RNA export factor 1
Paxip1	NM_001107844	10852694	1.28	26	8.9	ylw	PAX interacting (with transcription-activation domain) protein 1
Pelp1	NM_001024270	10744441	1.22	18	23.1	turq	proline, glutamic acid and leucine rich protein 1
Phb	NM_031851	10805784	0.77	-128	2.5	brwn	prohibitin

Polr2d	NM_001108886	10800667	0.81	-66	22.2	ylw	polymerase (RNA) II (DNA directed) polypeptide D
Polr2j	NM_001105921	10761070	0.77	-38	16.4	brwn	polymerase (RNA) II (DNA directed) polypeptide J
RGD1560011	NM_001195564	10910619	1.26	41	3.5	ylw	similar to Nuclear membrane binding protein NUCLING
RGD1560058	NM_001105821	10745551	0.81	-11	0.92	green	similar to prohibitin
Sdf2l1	NM_001109433	10752531	0.73	-125	19.2	brwn	stromal cell-derived factor 2-like 1
Snrpd2	NM_001109399	10704755	0.80	-38	16.8	ylw	small nuclear ribonucleoprotein D2 polypeptide
Snrpg	NM_001135085	10856965	0.66	-106	37.9	ylw	small nuclear ribonucleoprotein polypeptide G
Snw1	NM_001109279	10891373	1.21	66	0.2	mgnt	SNW domain containing 1
Tcfcp2l1	NM_001107170	10763531	1.23	13	10.2	turq	transcription factor CP2-like 1
Tle3	NM_053400	10910641	1.32	22	21.0	turq	transducin-like enhancer of split 3 (E(sp1) homolog, Drosophila)
Tnni3k	AY303692	10827484	0.72	-11	2.8	brwn	TNNI3 interacting kinase
Tsc22d2	NM_001191960	10815534	1.29	50	0	grey	TSC22 domain family, member 2
Zcchc2	BC100090	10763284	1.24	44	0	grey	zinc finger, CCHC domain containing 2
Zcchc7	NM_001106658	10868669	1.23	21	0	grey	zinc finger, CCHC domain containing 7
Zcchc9	NM_001013156	10820327	0.65	-128	0	grey	zinc finger, CCHC domain containing 9
Zfand5	NM_001106356	10714338	1.22	89	0	grey	zinc finger, AN1-type domain 5
Zfp219	NM_001007681	10783232	1.52	34	15.9	ylw	zinc finger protein 219
Zfp385d	NM_001013992	10782437	1.28	15	8.7	brwn	zinc finger protein 385D
Zfp709l2	NM_001163062	10901051	0.82	-28	4.6	ylw	zinc finger protein 709-like 2
Zfyve26	NM_001108038	10890809	1.35	20	11.1	ylw	zinc finger, FYVE domain containing 26
Zmym6	NM_001108681	10872114	1.32	35	0.6	ylw	zinc finger, MYM-type 6
Znf574	NM_001024258	10705100	1.24	12	6.0	ylw	zinc finger protein 574
Znf652	NM_001080207	10737651	1.22	49	11.9	turq	zinc finger protein 652
**TRANSLATION & PROTEIN MODIFICATION**							
LOC679154	ENSRNOT00000061278	10803440	1.36	25	0	grey	similar to DNA-directed RNA polymerase II largest subunit (RPB1)
Bat1	NM_133300	10831135	1.23	85	1.4	ylw	HLA-B associated transcript 1
Cpsf7	NM_001014245	10713894	1.29	19	0	grey	cleavage and polyadenylation specific factor 7, 59kDa
Eif2b2	NM_032058	10886014	0.80	-40	2.2	brwn	eukaryotic translation initiation factor 2B, subunit 2 beta
Gga3	NM_001108304	10739588	0.81	-81	0	grey	golgi associated, gamma adaptin ear containing, ARF binding protein 3
Grpel1	NM_024487	10777442	0.79	-49	10.8	ylw	GrpE-like 1, mitochondrial
Hnrnpa3	NM_198132	10837038	1.27	468	10.5	blue	heterogeneous nuclear ribonucleoprotein A3 (chr3)
Hnrnpa3	NM_001111295	10822786	1.28	526	7.9	blue	heterogeneous nuclear ribonucleoprotein A3 (chr2)
Hnrnpa3	NM_001111295	10869264	1.29	489	9.8	blue	heterogeneous nuclear ribonucleoprotein A3 (chr5)
Hnrnpa3	NM_001111295	10883583	1.23	346	1.3	blue	heterogeneous nuclear ribonucleoprotein A3 (chr6)
Hnrnpa3	NM_001111294	10717591	1.29	466	6.7	blue	heterogeneous nuclear ribonucleoprotein A3 (chr1)
Lmf2	NM_001079939	10906270	1.21	32	15.4	brwn	lipase maturation factor 2
Mrpl12	NM_001029900	10740153	0.80	-22	10.7	brwn	mitochondrial ribosomal protein L12
**Mrpl20**	**NM_001109428**	**10874701**	**0.82**	**-34**	**46.6**	**ylw**	**mitochondrial ribosomal protein L20**
Mrpl22	NM_001105781	10733782	0.74	-48	32.3	ylw	mitochondrial ribosomal protein L22
**Mrpl54**	**NM_001106770**	**10893586**	**0.82**	**-154**	**48.5**	**ylw**	**mitochondrial ribosomal protein L54**
mrpl9	NM_001007696	10817252	0.81	-67	6.5	ylw	mitochondrial ribosomal protein L9
Mrps18c	NM_001105996	10775532	0.82	-273	16.5	ylw	mitochondrial ribosomal protein S18C
**Pfdn5**	**NM_001106794**	**10899531**	**0.76**	**-153**	**54.3**	**ylw**	**prefoldin subunit 5**
Prpf8	NM_001191590	10735897	1.21	103	3.0	ylw	PRP8 pre-mRNA processing factor 8 homolog (S. cerevisiae)
Rbm4	ENSRNOT00000026621	10727643	1.27	34	12.8	ylw	RNA binding motif protein 4
RGD1561984	ENSRNOT00000041555	10917299	0.81	-39	41.7	ylw	similar to ribosomal protein L27a
RGD1562404	ENSRNOT00000051652	10896040	0.81	-30	20.0	ylw	similar to ribosomal protein S18
Rnps1	NM_001011890	10732058	1.20	63	18.8	turq	ribonucleic acid binding protein S1
**Rpl13a**	**NM_173340**	**10797935**	**0.78**	**-556**	**47.9**	**ylw**	**ribosomal protein L13A (chr17)**
**Rpl13a**	**NM_173340**	**10833586**	**0.79**	**-527**	**48.4**	**ylw**	**ribosomal protein L13A (chr20)**
Rpl15	NM_139114	10779331	0.82	-17	11.7	brwn	ribosomal protein L15
**Rpl22**	**NM_031104**	**10863772**	**0.79**	**-124**	**53.8**	**ylw**	**ribosomal protein L22 (chr4)**
**Rpl22**	**NM_031104**	**10897802**	**0.80**	**-97**	**57.2**	**ylw**	**ribosomal protein L22 (chr7)**
**Rpl22l1**	**NM_001108548**	**10814650**	**0.74**	**-75**	**43.3**	**ylw**	**ribosomal protein L22 like 1 (chr2)**
Rpl22l1	NM_001108548	10839803	0.73	-84	42.4	ylw	ribosomal protein L22 like 1 (chr3)
**Rpl31**	**NM_022506**	**10760811**	**0.83**	**-395**	**51.4**	**ylw**	**ribosomal protein L31**
**Rpl35a**	**NM_021264**	**10727462**	**0.79**	**-196**	**66.1**	**ylw**	**ribosomal protein L35a (chr1)**
**Rpl35a**	**NM_021264**	**10740558**	**0.80**	**-138**	**65.3**	**ylw**	**ribosomal protein L35a (chr10)**
**Rpl35a**	**NM_021264**	**10754694**	**0.79**	**-201**	**65.8**	**ylw**	**ribosomal protein L35a (chr11)**
**Rpl35a**	**NM_021264**	**10760018**	**0.79**	**-196**	**66.1**	**ylw**	**ribosomal protein L35a (chr12)**
**Rpl35a**	**NM_021264**	**10797473**	**0.79**	**-179**	**68.5**	**ylw**	**ribosomal protein L35a (chr17)**
**Rpl35a**	**NM_021264**	**10847995**	**0.79**	**-173**	**56.9**	**ylw**	**ribosomal protein L35a (chr3)**
**Rpl35a**	**NM_021264**	**10873916**	**0.79**	**-196**	**66.1**	**ylw**	**ribosomal protein L35a (chr5)**
**Rpl35a**	**NM_021264**	**10885205**	**0.79**	**-178**	**65.2**	**ylw**	**ribosomal protein L35a (chr6)**
Rpl36	NM_022504	10890858	0.83	-82	3.7	ylw	ribosomal protein L36
**Rpl39**	**NM_012875**	**10776291**	**0.63**	**-378**	**52.3**	**ylw**	**ribosomal protein L39 (chr14)**
Rpl39	NM_012875	10782917	0.66	-387	41.3	ylw	ribosomal protein L39 (chr15)
**Rpl39**	**NM_012875**	**10803301**	**0.64**	**-353**	**51.9**	**ylw**	**ribosomal protein L39 (chr18)**
Rpl39	NM_012875	10829688	0.66	-387	41.3	ylw	ribosomal protein L39 (chr20)

Rpl39	NM_012875	10895022	0.66	-387	41.3	ylw	ribosomal protein L39 (chr7)
**Rpl39**	**NM_012875**	**10932013**	**0.65**	**-453**	**48.1**	**ylw**	**ribosomal protein L39 (chrX)**
Rpl41	NM_139083	10774894	0.75	-549	39.3	ylw	ribosomal protein L41
Rps10	NM_031109	10742029	0.83	-47	20.0	ylw	ribosomal protein S10
**Rps14**	**NM_022672**	**10802007**	**0.70**	**-329**	**76.2**	**ylw**	**ribosomal protein S14 (chr18)**
**Rps14**	**NM_022672**	**10871997**	**0.69**	**-404**	**76.7**	**ylw**	**ribosomal protein S14 (chr5)**
**Rps14**	**ENSRNOT00000059501**	**10927281**	**0.68**	**-359**	**72.2**	**ylw**	**ribosomal protein S14 (chr9)**
Rps15a	NM_053982	10725159	0.82	-107	39.8	ylw	ribosomal protein S15a (chr1)
Rps15a	NM_053982	10752146	0.83	-103	41.8	ylw	ribosomal protein S15a (chr11)
Rps15a	NM_053982	10760208	0.78	-119	40.9	ylw	ribosomal protein S15a (chr12)
**Rps17**	**NM_017152**	**10723233**	**0.68**	**-313**	**64.2**	**ylw**	**ribosomal protein S17 (chr1)**
**Rps17**	**NM_017152**	**10844946**	**0.69**	**-306**	**64.7**	**ylw**	**ribosomal protein S17 (chr3)**
Rps18	NM_213557	10749058	0.75	-255	34.7	ylw	ribosomal protein S18 (chr10)
Rps18	NM_213557	10828433	0.79	-189	30.1	ylw	ribosomal protein S18 (chr20)
Rps18	NM_213557	10870043	0.76	-189	37.8	ylw	ribosomal protein S18 (chr5)
**Rps20**	**NM_001007603**	**10876420**	**0.83**	**-484**	**61.7**	**ylw**	**ribosomal protein S20**
**Rps24**	**NM_031112**	**10746246**	**0.82**	**-38**	**50.9**	**ylw**	**ribosomal protein S24 (chr10)**
**Rps24**	**NM_031112**	**10786132**	**0.83**	**-38**	**48.0**	**ylw**	**ribosomal protein S24 (chr16)**
Rps27	NM_053597	10824596	0.72	-15	26.0	ylw	ribosomal protein S27
**Rps27a**	**NM_031113**	**10778853**	**0.82**	**-322**	**63.4**	**ylw**	**ribosomal protein S27a (chr14)**
**Rps27a**	**NM_031113**	**10879392**	**0.80**	**-323**	**62.1**	**ylw**	**ribosomal protein S27a (chr5)**
Rps3	NM_001009239	10723891	0.82	-999	25.4	ylw	ribosomal protein S3
**Rps7**	**NM_031570**	**10785933**	**0.82**	**-434**	**61.0**	**ylw**	**ribosomal protein S7 (chr15)**
**Rps7**	**NM_031570**	**10881922**	**0.82**	**-350**	**64.2**	**ylw**	**ribosomal protein S7 (chr5)**
**Rps7**	**NM_031570**	**10889455**	**0.82**	**-358**	**66.9**	**ylw**	**ribosomal protein S7 (chr6)**
Sf1	NM_001110793	10713362	1.39	69	17.8	ylw	splicing factor 1
Sf3a1	NM_001107235	10773809	1.23	18	15.5	turq	splicing factor 3a, subunit 1
Sf3b5	NM_001126092	10701797	0.77	-39	11.9	ylw	splicing factor 3b, subunit 5
Sfrs16	NM_001024294	10812086	1.27	11	0	grey	splicing factor, arginine/serine-rich 16
Tfip11	NM_001008291	10809462	0.83	-12	0	grey	tuftelin interacting protein 11
Thyn1	NM_001007661	10921175	0.82	-22	20.7	ylw	thymocyte nuclear protein 1
Wdr18	NM_001039027	10900710	0.76	-49	20.6	brwn	WD repeat domain 18
**MISCELLANEOUS & UNKNOWN**							
Depdc5	NM_001107229	10777832	1.23	23	8.0	ylw	DEP domain containing 5
**Fam108c1**	**NM_001100736**	**10723413**	**1.21**	**22**	**38.4**	**turq**	**family with sequence similarity 108, member C1**
Fam53c	ENSRNOT00000027499	10800913	1.21	21	11.1	turq	family with sequence similarity 53, member C
Fam75a4	ENSRNOT00000047043	10796941	0.69	-33	9.9	brwn	family with sequence similarity 75, member A4
**Ints3**	**ENSRNOT00000020575**	**10824624**	**1.20**	**39**	**40.7**	**turq**	**integrator complex subunit 3**
LOC100361529	ENSRNOT00000025317	10727493	1.32	25	5.7	ylw	rCG47324-like
LOC100362601	ENSRNOT00000049011	10728593	0.77	-17	0	grey	PBPC1BS-like
LOC100363176	XM_002728834	10728981	0.82	-10	12.8	ylw	mCG145114-like
LOC681410	ENSRNOT00000061231	10793891	1.27	18	33.0	turq	hypothetical protein LOC681410
LOC688786	NM_001195245	10759636	0.77	-67	6.0	ylw	similar to CG14483-PA
R3hdm2	NM_001130557	10895888	1.41	50	6.7	ylw	R3H domain containing 2
RGD1304878	ENSRNOT00000006131	10892097	1.22	23	8.2	turq	similar to 2410024A21Rik protein
RGD1306576	NM_001106648	10876169	0.82	-11	7.2	brwn	similar to hypothetical protein
RGD1307410	ENSRNOT00000038993	10749577	1.31	15	7.6	turq	similar to hypothetical protein FLJ31528
RGD1307597	NM_001106744	10891203	1.26	89	5.7	ylw	similar to mKIAA0317 protein
RGD1308297	BC082039	10785745	1.24	64	0	grey	similar to CG10084-PA
RGD1311309	ENSRNOT00000003061	10772342	1.25	34	29.8	ylw	similar to 2510002A14Rik protein
RGD1559695	ENSRNOT00000040236	10904444	1.26	18	2.82	green	similar to FLJ43860 protein
RGD1566320	NM_001106520	10840415	0.73	-36	40.6	ylw	RGD1566320
**Spryd4**	**NM_001037765**	**10899736**	**0.73**	**-46**	**23.8**	**brwn**	**SPRY domain containing 4**
Tmem140	NM_001009709	10854482	1.33	39	27.4	ylw	transmembrane protein 140
Tmem177	NM_001037776	10767162	0.83	-11	0.6	brwn	transmembrane protein 177
Tmem185b	NM_001191668	10763547	1.22	61	19.3	turq	transmembrane protein 185B
Tmem208	NM_001106179	10807211	0.81	-31	21.1	ylw	transmembrane protein 208
Tmem223	NM_001191104	10713648	0.78	-63	26.6	ylw	transmembrane protein 223
Tmem37	NM_139095	10931159	1.29	18	2.0	ylw	transmembrane protein 37
Tmem39b	NM_001014192	10880047	1.26	18	0	grey	transmembrane protein 39b
Tmem60	NM_001191610	10860269	1.23	47	0	grey	transmembrane protein 60
Tmem9	NM_001105953	10764260	0.72	-26	0	grey	transmembrane protein 9
Trim14	NM_001107934	10876594	1.65	23	1.0	red	tripartite motif-containing 14
Ttc38	NM_001130499	10898368	1.20	19	10.3	turq	tetratricopeptide repeat domain 38
Ttyh3	NM_001107124	10756931	1.39	22	4.2	turq	tweety homolog 3 (Drosophila)
Wdr91	NM_001127298	10861751	1.22	13	0	grey	WD repeat domain 91
**EST's**							
RGD1306917	BC169127	10797493	0.78	-34	5.9	ylw	similar to RIKEN cDNA 2900010M23 (chr17)
RGD1306917	BC169127	10831825	0.79	-29	4.3	ylw	similar to RIKEN cDNA 2900010M23 (chr20)

**RGD1359127**	**ENSRNOT00000021939**	**10893896**	**0.82**	**-10**	**31.3**	**brwn**	**similar to RIKEN cDNA 2310011J03**
**RGD1563155**	**NM_001134598**	**10936897**	**0.66**	**-23**	**9.84**	**green**	**similar to RIKEN cDNA 1700054O13**
RGD1564406	ENSRNOT00000047128	10938521	0.59	-10	0	grey	similar to RIKEN cDNA 1700019M22
RGD1566149	NM_001109038	10745222	1.42	15	4.2	turq	similar to CDNA sequence BC017647
RGD1566266	NM_001177613	10758599	1.27	19	16.1	turq	similar to hypothetical protein FLJ21127
	NC_001665	10930620	2.01	633	3.7	mgnt	
	NC_001665	10930610	1.26	12	3.2	mgnt	
	**J01884**	**10830454**	**0.47**	**-77**	**46.4**	**ylw**	
	GENSCAN00000028117	10845645	0.68	-59	9.7	ylw	
	GENSCAN00000017343	10873814	1.34	17	5.1	ylw	
	FQ234631	10894268	0.62	-11	34.5	ylw	(chr7)
	FQ234631	10918270	0.62	-11	34.5	ylw	(chr8)
	**FQ233900**	**10765040**	**0.57**	**-84**	**44.2**	**ylw**	
	**FQ229993**	**10737194**	**0.60**	**-235**	**44.9**	**ylw**	
	FQ226474	10918820	0.67	-262	28.7	ylw	
	FQ225819	10717949	0.70	-31	32.0	ylw	(chr1)
	FQ225819	10863888	0.70	-31	32.0	ylw	(chr4)
	**FQ223410**	**10855185**	**0.56**	**-309**	**60.6**	**ylw**	
	FQ220555	10906590	0.70	-58	14.4	brwn	(chr7)
	FQ220555	10940411	0.78	-38	14.6	brwn	(chrX
	FQ217715	10702192	0.72	-26	13.1	ylw	(chr1)
	FQ217715	10739209	0.72	-26	13.1	ylw	(chr10)
	FQ217715	10788077	0.72	-26	13.1	ylw	(chr16)
	FQ217715	10863402	0.72	-26	13.1	ylw	(chr4)
	FQ217715	10885957	0.72	-26	13.1	ylw	(chr6)
	FQ217715	10922249	0.72	-26	13.1	ylw	(chr9)
	FQ217715	10937321	0.72	-26	13.1	ylw	(chrX)
	FQ212372	10891830	0.81	-37	8.1	ylw	(chr6)
	ENSRNOT00000069734	10911811	0.44	-39	4.0	ylw	
	ENSRNOT00000069363	10861115	0.73	-17	4.3	ylw	
	ENSRNOT00000069284	10714056	0.70	-13	2.0	ylw	
	ENSRNOT00000061400	10936358	1.86	11	0	grey	
	ENSRNOT00000060756	10915778	1.42	16	3.7	blue	
	ENSRNOT00000060122	10884046	0.73	-32	6.0	ylw	
	ENSRNOT00000059111	10784523	1.34	39	7.4	ylw	
	ENSRNOT00000058749	10795719	0.66	-14	0	grey	
	ENSRNOT00000057365	10924441	0.74	-108	20.6	brwn	
	ENSRNOT00000056936	10878924	1.66	21	16.2	ylw	
	ENSRNOT00000056621	10935626	0.78	-11	0	grey	
	ENSRNOT00000056335	10864908	0.82	-11	5.1	ylw	
	ENSRNOT00000055058	10842742	1.27	29	2.3	turq	
	ENSRNOT00000054517	10797013	0.60	-72	36.8	ylw	(chr17)
	ENSRNOT00000054517	10932228	0.60	-72	36.8	ylw	(chrX)
	ENSRNOT00000054178	10914316	0.60	-35	30.2	ylw	
	ENSRNOT00000054147	10713602	0.74	-103	14.5	ylw	
	ENSRNOT00000054140	10783318	0.72	-31	37.2	ylw	
	ENSRNOT00000053987	10755127	0.81	-23	0	grey	
	ENSRNOT00000053974	10722461	0.55	-42	5.2	black	
	ENSRNOT00000053951	10703224	0.62	-50	26.3	ylw	
	ENSRNOT00000053936	10722443	0.67	-20	0.9	black	
	ENSRNOT00000053934	10722465	0.58	-20	2.8	black	
	**ENSRNOT00000053919**	**10755129**	**0.65**	**-340**	**48.8**	**ylw**	
	ENSRNOT00000053909	10766795	0.64	-16	6.0	ylw	
	ENSRNOT00000053902	10827450	0.63	-692	7.1	ylw	
	**ENSRNOT00000053882**	**10722421**	**0.57**	**-89**	**12.7**	**black**	
	ENSRNOT00000053855	10722481	0.56	-39	7.3	black	
	ENSRNOT00000053847	10767075	0.70	-112	26.0	ylw	
	**ENSRNOT00000053552**	**10713608**	**0.64**	**-132**	**60.6**	**ylw**	
	ENSRNOT00000053542	10871771	0.59	-101	36.3	ylw	
	ENSRNOT00000053479	10851380	0.54	-34	39.9	ylw	
	ENSRNOT00000053476	10702293	0.65	-30	31.2	ylw	
	ENSRNOT00000053459	10765038	0.65	-23	5.6	ylw	
	ENSRNOT00000053447	10765495	0.62	-34	42.8	ylw	
	ENSRNOT00000053408	10918383	0.63	-59	16.7	ylw	
	ENSRNOT00000053402	10721698	0.58	-626	42.0	ylw	
	ENSRNOT00000053328	10855946	0.59	-141	28.7	ylw	
	ENSRNOT00000053325	10711125	0.74	-25	9.9	ylw	
	ENSRNOT00000053296	10805849	0.60	-14	13.2	ylw	
	ENSRNOT00000053279	10708691	0.67	-443	35.6	ylw	

	**ENSRNOT00000053230**	**10758033**	**0.53**	**-116**	**44.5**	**ylw**	
	ENSRNOT00000053208	10774100	0.74	-33	32.9	ylw	
	ENSRNOT00000053184	10722429	0.66	-18	4.4	black	
	ENSRNOT00000053117	10722433	0.53	-42	3.3	black	
	ENSRNOT00000053105	10881643	0.64	-31	11.2	ylw	
	ENSRNOT00000053019	10722451	0.57	-18	5.6	black	
	ENSRNOT00000052995	10723898	0.55	-73	39.5	ylw	
	ENSRNOT00000052978	10721700	0.57	-90	38.5	ylw	
	ENSRNOT00000052964	10801135	0.58	-731	23.9	ylw	
	ENSRNOT00000052956	10755131	0.78	-16	16.3	ylw	
	**ENSRNOT00000052947**	**10826602**	**0.57**	**-114**	**56.8**	**ylw**	
	ENSRNOT00000052871	10709784	0.69	-28	19.8	ylw	
	ENSRNOT00000052797	10828158	0.69	-16	28.8	ylw	
	ENSRNOT00000052790	10831150	0.69	-14	40.2	ylw	
	ENSRNOT00000052727	10910764	0.58	-77	33.5	ylw	
	ENSRNOT00000052710	10838282	0.62	-121	32.8	ylw	(chr3)
	ENSRNOT00000052710	10924172	0.62	-121	32.8	ylw	(chr9)
	ENSRNOT00000052689	10896964	0.63	-33	13.4	ylw	
	ENSRNOT00000052661	10722437	0.63	-29	9.8	black	
	**ENSRNOT00000052632**	**10846928**	**0.58**	**-299**	**55.6**	**ylw**	**(chr3)**
	**ENSRNOT00000052632**	**10871769**	**0.58**	**-351**	**53.8**	**ylw**	**(chr5)**
	**ENSRNOT00000052628**	**10834602**	**0.60**	**-201**	**47.8**	**ylw**	
	ENSRNOT00000052625	10873578	0.67	-12	13.0	ylw	
	ENSRNOT00000052571	10790465	0.74	-14	1.3	blue	
	**ENSRNOT00000052564**	**10722431**	**0.58**	**-93**	**11.2**	**black**	
	ENSRNOT00000052542	10871415	0.62	-15	0	grey	
	ENSRNOT00000052507	10722471	0.67	-25	8.1	black	
	**ENSRNOT00000052442**	**10709825**	**0.56**	**-256**	**57.6**	**ylw**	
	ENSRNOT00000051889	10787722	0.79	-24	0	grey	
	**ENSRNOT00000049614**	**10736693**	**0.73**	**-26**	**59.3**	**ylw**	
	ENSRNOT00000048926	10889421	1.41	17	0.6	blue	
	**ENSRNOT00000048141**	**10839058**	**1.32**	**13**	**21.6**	**brwn**	
	ENSRNOT00000048078	10801778	1.41	26	5.8	blue	
	ENSRNOT00000043131	10774908	0.78	-22	0	grey	
	ENSRNOT00000041344	10785800	0.70	-28	8.1	ylw	
	ENSRNOT00000039311	10789806	1.22	22	3.2	brwn	
	ENSRNOT00000036778	10849861	0.76	-41	0	grey	
	ENSRNOT00000036340	10781280	0.72	-84	28.9	ylw	
	ENSRNOT00000035374	10850519	0.76	-20	0	grey	
	ENSRNOT00000031816	10894159	0.76	-28	0	grey	
	ENSRNOT00000031737	10811706	1.36	58	2.7	ylw	
	ENSRNOT00000030857	10859792	0.72	-26	0	grey	
	ENSRNOT00000030293	10718408	0.76	-30	9.0	ylw	
	ENSRNOT00000025375	10802373	0.83	-40	4.3	brwn	
	ENSRNOT00000018963	10814892	0.73	-36	30.5	ylw	
	ENSRNOT00000017777	10792049	0.69	-63	18.7	ylw	
	ENSRNOT00000016461	10722816	0.71	-21	13.1	ylw	
	ENSRNOT00000016409	10890853	0.63	-10	7.6	ylw	
	ENSRNOT00000013808	10886597	0.81	-11	0.3	turq	
	ENSRNOT00000013307	10784444	0.81	-12	5.2	brwn	
	ENSRNOT00000006999	10939166	0.74	-13	3.7	ylw	
	ENSRNOT00000004332	10768301	0.77	-181	42.4	ylw	
	ENSRNOT00000003899	10934526	0.76	-14	0	grey	
	ENSRNOT00000001661	10760580	1.21	11	2.4	turq	
	ENSRNOT00000001562	10762723	1.28	73	32.5	turq	
	ENSRNOT00000001469	10760483	1.27	16	5.8	turq	
	ENSRNOT00000001338	10761553	1.21	87	1.4	ylw	
	BC099108	10746854	1.47	16	2.5	ylw	
	BC087666	10891594	1.37	103	18.0	ylw	
	**AF272707**	**10867318**	**0.60**	**-80**	**48.6**	**ylw**	
	---	10821115	0.78	-45	13.3	blue	
	---	10868796	0.78	-21	6.5	blue	
	---	10796230	1.53	14	6.8	brwn	
	---	10903259	0.83	-26	0	grey	
	---	10791602	1.32	21	0	grey	
	---	10833416	1.32	21	0	grey	
	---	10812130	1.23	23	21.8	turq	
	---	10931663	0.76	-21	5.7	turq	
	---	10771204	0.82	-10	3.2	ylw	

	---	10825510	1.41	41	1.6	ylw		
	---	10700166	1.46	31	0.4	black		
	---	10700873	1.25	625	16.2	blue		
	---	10701572	1.71	37	11.9	blue		
	---	10701089	0.76	-12	10.7	blue		
	---	10700930	1.24	81	6.9	blue		
	---	10701316	1.84	37	1.8	blue		
	---	10701352	0.73	-10	8.4	brwn		
	---	10701196	0.71	-11	3.6	brwn		
	---	10700331	0.60	-41	0	grey		
	---	10700192	0.74	-34	0	grey		
	---	10700239	0.58	-31	0	grey		
	---	10701598	0.70	-23	0	grey		
	---	10701547	0.51	-21	0	grey		
	---	10700874	0.70	-17	0	grey		
	---	10701169	0.55	-16	0	grey		
	---	10700194	0.62	-15	0	grey		
	---	10701447	1.25	10	0	grey		
	---	10700552	1.58	12	0	grey		
	---	10700098	1.43	13	0	grey		
	---	10700203	1.54	13	0	grey		
	---	10700337	1.86	14	0	grey		
	---	10701588	1.27	15	0	grey		
	---	10701277	1.54	16	0	grey		
	---	10700605	1.33	16	0	grey		
	---	10701373	1.31	17	0	grey		
	---	10701463	1.73	19	0	grey		
	---	10700443	1.64	22	0	grey		
	---	10700424	1.57	31	0	grey		
	---	10700479	1.28	113	0	grey		
	---	10701009	1.60	80	0.4	pink		
	---	10700438	0.77	-49	21.3	turq		
	---	10700304	1.32	689	13.9	turq		
	---	10701325	0.60	-25	2.9	turq		
	---	10700274	0.78	-242	34.8	ylw		
	---	10700266	1.26	23	9.0	ylw		
	---	10700409	0.73	-74	8.6	ylw		
	---	10701067	1.64	21	3.8	ylw		
	---	10700215	0.58	-20	0.8	ylw		

k.in is interconnection index calculated for each gene in a given module.
Top 10% genes of each male or female module with highest k.in value are marked by bold font.
Modules color abbreviaton: brwn - brown, turq - turquoise, l-grn - lightgreen, l-cn - lightcyan, l-ye - lightyellow, mgnt - magenta, gr-yel - greenyellow, ylw - yellow, m-blu - midnightblue, gr60 - grey60 (not in module).
