# Supplementary material for: Epigenetic transgenerational inheritance of somatic transcriptomes and epigenetic control regions
Source: Genome Biol. 2012 Oct 3;13(10):R91. doi: 10.1186/gb-2012-13-10-r91 (PMC3491419; doi:10.1186/gb-2012-13-10-r91)
Supplement: Additional file 6 — Table S2 - pathway enrichment in signature lists. [file gb-2012-13-10-r91-S6.pdf]

Supplemental Table S2

Pathway Enrichment for 11 Male and Female Rat Tissue Signature Lists

Pathway name \ Sex.Tissue	11 Male & fem.Tissues	Male 6 tissues	M.Heart	M.Kidney	M.Liver	M.Prost	M.SV	M.Testis	Fem 5 tissues	F.Heart	F.Kidney	F.Liver	F.Ovary	F.Uterus
**Number of genes in input list**	**4059**	**3046**	**172**	**725**	**266**	**1112**	**274**	**552**	**1298**	**408**	**151**	**99**	**305**	**279**
**Total Number of Affected Pathways**	**230**	**224**	**79**	**151**	**141**	**191**	**90**	**99**	**191**	**108**	**79**	**29**	**80**	**138**
Pathways in cancer	45	32	3	7	6	13	1	2	17	6	3	1	3	4
Protein processing in endoplasmic reticulum	39	38	3	4	1	22	5	4	3	1			1	1
HTLV-I infection	39	31		8	3	14	3	2	10	4	2		1	3
RNA transport	39	31	1	8		17	3	6	11	4		1	2	4
Transcriptional misregulation in cancers	31	26	1	8	6	7	4	3	12	6	3	1		3
Herpes simplex infection	30	23	2	10	3	7	2	1	9	3	1		2	3
Lysosome	29	27	1	5	4	16	2	2	5	1			1	3
Ribosome	29	27	2	5		1		20	4	1	1	2		
Endocytosis	29	26	1	4	8	11	1	3	5	2				3
Phagosome	28	27	1	6	6	12		2	7		3			4
MAPK signaling pathway	28	21	1	7	3	7	1	2	8	3			1	4
Spliceosome	27	17		8	1	1	2	7	12	6	1		1	5
Regulation of actin cytoskeleton	26	24	1	6	6	9		3	5	3		1		1
Alzheimer's disease	26	22	2	4		7		10	5				2	3
Huntington's disease	26	22	1	6		2		14	5	1		1	2	1
Purine metabolism	26	22	1	6	2	4	2	6	7	2				5
Focal adhesion	26	21	2	2	7	8		3	9	3		2		4
Chemokine signaling pathway	24	23	3	4	6	9	1	1	4				2	2
Pyrimidine metabolism	24	20	1	6	1	6	1	5	6	1		1	1	3
Tuberculosis	24	20		6	6	9	1		9	1	1			7
Influenza A	23	21	1	5	2	11	1	3	5	1	2			2
Oxidative phosphorylation	23	20	1	4		5		11	5		1		1	3
Leukocyte transendothelial migration	22	18	2	1	7	8		1	6	4	1			1
Cytokine-cytokine receptor interaction	21	19	1	6	7	6			4	3				1
Osteoclast differentiation	21	18	1	2	6	10	1		5	1			1	3
Cell adhesion molecules (CAMs)	20	14	2	3	4	6	1		7	3	1		1	2
Insulin signaling pathway	19	13		2	3	6	2		6	2				4
mRNA surveillance pathway	19	13	1	4		5	2	4	8	4			2	2

Ribosome biogenesis in eukaryotes	19	13		4	1	6		4	7	1		1	5	
Wnt signaling pathway	18	14	1	3	2	6	1	1	4	2				2
Neuroactive ligand-receptor interaction	18	12	1		3	6	1	1	7	3	3			2
RNA degradation	18	12	1	2		4	2	4	7	1	1		2	4
Tight junction	18	12	1	2	2	6		1	9	6			1	2
Calcium signaling pathway	17	16	1	4	3	7			4	2	1			1
Parkinson's disease	17	15	1	3		3		9	2				1	1
Ubiquitin mediated proteolysis	17	12				12	1		7	3	1		1	2
Measles	16	16		3	3	8	1		4	2				2
Hepatitis C	16	11		1	2	7	1		6	1			2	3
Cell cycle	16	10		2		9	1		4	1			2	1
Phosphatidylinositol signaling system	16	10		1	3	6		2	5	3			1	1
Fc gamma R-mediated phagocytosis	15	16	1	1	5	9		1						
Natural killer cell mediated cytotoxicity	15	14	1	2	6	4		1	4	1	1			2
Systemic lupus erythematosus	15	14		6	2	3	1	2	3	1	2			1
Dopaminergic synapse	15	12	1	2	2	6	1		3	1			1	1
Antigen processing and presentation	15	11	1	4	2	2	1	1	4	1	1			2
Small cell lung cancer	15	11	1	1	2	5	1	1	5		1	1	1	2
Apoptosis	15	10		2	2	5	1		8	2	2		1	3
Toxoplasmosis	15	10		2	2	6	1		6		2		1	3
Rheumatoid arthritis	14	12	1	1	3	5		1	5	1	1			3
Arrhythmogenic right ventricular cardiomyopathy (ARVC)	14	11		5	1	5			3	2				1
Jak-STAT signaling pathway	14	11		2	3	5	1		3	2	1			
Chagas disease (American trypanosomiasis)	14	10		3	3	3	1		5		1		1	4
Inositol phosphate metabolism	14	9		1	1	4		4	4	2			1	1
Serotonergic synapse	14	8	2	2	1	3			6	1	2		1	3
N-Glycan biosynthesis	13	13	1	1		11	1	1						
Dilated cardiomyopathy (DCM)	13	12		7		3			4	2	1			1
Adherens junction	13	11			2	9			5	3				2
Glycerophospholipid metabolism	13	11	2	2	2	3	1	3	3	1				2
T cell receptor signaling pathway	13	10	1		4	4	1	1	5	1			1	3
Complement and coagulation cascades	13	8		3	1	1	2	2	4	2	1			2
Protein digestion and absorption	13	8		3	1	4			5		2	2		1
Adipocytokine signaling pathway	13	6			1	4	1		9	1	1		3	4
Hematopoietic cell lineage	12	12	1	2	5	5			4	1	1			2
Synaptic vesicle cycle	12	12			1	10		1	2					2
Aminoacyl-tRNA biosynthesis	12	11		1		5	7		2				1	2
Amoebiasis	12	11	1	3	1	4	1		3			1		2
Pertussis	12	11		3	3	3	1	1	3		2			2
Salmonella infection	12	11		4	1	4	2		3		1	1		1
Toll-like receptor signaling pathway	12	11	1	3	2	4	1		2				1	1
Axon guidance	12	10	1		3	5		3	3	1		1		1
Bacterial invasion of epithelial cells	12	10	1	1	1	6	1	1	3	2				1

Viral myocarditis	12	10		3	2	4			4	1	1	1		1
Cardiac muscle contraction	12	9	1	4	1	1		2	4	1	2			1
Vascular smooth muscle contraction	12	9	1	3	2	1		1	5	2	1			2
ECM-receptor interaction	12	8	1	2	2	3	1		5		2	2		1
Hypertrophic cardiomyopathy (HCM)	12	8		6		2			5	2	1			2
GnRH signaling pathway	11	11	2	4	1	3			2					2
Neurotrophin signaling pathway	11	11	1	1	3	6	1		1					1
Circadian rhythm - mammal	11	9	1		2	3	4		5	3				2
Cytosolic DNA-sensing pathway	11	9		3	1	3	1	1	4		1		1	2
Mineral absorption	11	9		5	1	2	1		3	1	1		1	
Progesterone-mediated oocyte maturation	11	9		1	1	5	2		2					2
Staphylococcus aureus infection	11	9		4	2	2	1		3	1	2			1
Chronic myeloid leukemia	11	7			1	5	1		5	1	2		1	1
p53 signaling pathway	11	7		1		5	4		2	1				1
PPAR signaling pathway	11	5		1	2	2	1		8		1	2	1	4
B cell receptor signaling pathway	10	10			3	4	1	1	2				1	1
Leishmaniasis	10	10		3	2	4	1		2		1			1
Bile secretion	10	9	1	2	1	4			2		1		1	
Oocyte meiosis	10	9	1	1	1	5			2				1	1
Acute myeloid leukemia	10	8		1	2	4	1		3		1		1	1
Cholinergic synapse	10	8		2	3	2			4	1			1	2
mTOR signaling pathway	10	8	2	1	1	4			3	2				1
Porphyrin and chlorophyll metabolism	10	8		4	2		1	1	3	1	1	1	1	
Prostate cancer	10	8	1		2	4	1		3	1			1	1
Pancreatic cancer	10	7	1		2	3	1		5	2	1		1	1
Basal transcription factors	10	6	2	1	1	1	1		4	1		1	2	
Drug metabolism - other enzymes	10	5		3	1	1			5	3			2	
Malaria	10	5	1		4		1		7		3		4	3
Fc epsilon RI signaling pathway	9	10	1		5	3			1					1
Gap junction	9	10	1	2	3	3			1					1
Endocrine and other factor-regulated calcium reabsorption	9	9		4	2	2			1		1			
Salivary secretion	9	9		1	3	4	1		2	1	1			
GABAergic synapse	9	8		2		3	1	1	3				1	2
RNA polymerase	9	8		4		1		3	2					2
VEGF signaling pathway	9	8			2	5		2	2	1				1
Glutamatergic synapse	9	7		2		2	1	1	3	1			1	1
Legionellosis	9	7		2		3	2		4		1		1	2
Prion diseases	9	7		2	3		2		3		1	1		2
TGF-beta signaling pathway	9	6	1	1	3	1			4	2			2	
Cysteine and methionine metabolism	9	5		3	1	1			4	1			1	2
Renal cell carcinoma	9	5	1		1	3			5	2	1		1	1
Glutathione metabolism	8	8		2		3	2	2	1					1
Proteasome	8	8		3		3		3						
Amino sugar and nucleotide sugar metabolism	8	7	1		1	4		1	1				1	
Gastric acid secretion	8	7		1	2	2	1		2		2			
RIG-I-like receptor signaling pathway	8	7		1	1	3	2		2				1	1
Notch signaling pathway	8	6	1			4		1	3	2			1	

Valine, leucine and isoleucine degradation	8	6		1				5	2					2
Endometrial cancer	8	5			1	4			3	3				
Glycosylphosphatidylinositol(GPI)-anchor biosynthesis	8	4				3		1	4	1		1	1	1
Protein export	7	8	1			3	2	1						
Butanoate metabolism	7	7			2			5						
Glioma	7	7			3	4								
Melanogenesis	7	7		3	1	2			1	1				
Pancreatic secretion	7	7			2	5			2	1	1			
Collecting duct acid secretion	7	6				5		1	3		1			2
Colorectal cancer	7	6			2	3			2	2				
Aldosterone-regulated sodium reabsorption	7	5			3	3			1		1			
Arachidonic acid metabolism	7	5	1	2	1	1		1	3		1		1	1
Bladder cancer	7	5	1	2		2			3	2				1
Hedgehog signaling pathway	7	5		3	1	1			2				1	1
Amyotrophic lateral sclerosis (ALS)	7	4				2		2	4		2	1		1
Metabolism of xenobiotics by cytochrome P450	7	4	1			2	1	1	4	2				3
Melanoma	6	6			2	4								
Non-small cell lung cancer	6	6			2	4								
Sphingolipid metabolism	6	6		2		4		1	1					1
ErbB signaling pathway	6	5			2	3			2	1				1
Regulation of autophagy	6	5				4		1	1					1
Steroid biosynthesis	6	5				5			1	1				
Vasopressin-regulated water reabsorption	6	5		1		2		1	2	1	1			
ABC transporters	6	4		1		3			2				2	
Type I diabetes mellitus	6	4		3		1			2		1			1
Drug metabolism - cytochrome P450	6	3				2	1	1	4	2				3
Other glycan degradation	5	5		2		4			2		1			1
SNARE interactions in vesicular transport	5	5		1	1	1		3						
NOD-like receptor signaling pathway	5	4			1	2	1		3		1		1	1
Primary immunodeficiency	5	4	1		1	2			2				1	1
African trypanosomiasis	5	3			1	1			4		1		3	3
Thyroid cancer	4	4				4		1	1				1	
Glycolysis / Gluconeogenesis	4	3			1	1		1	1				1	
Synthesis and degradation of ketone bodies	3	3						3						

Only Pathways with 3 or more affected genes per signature list are shown
