# Supplementary material for: Epigenetic transgenerational inheritance of somatic transcriptomes and epigenetic control regions
Source: Genome Biol. 2012 Oct 3;13(10):R91. doi: 10.1186/gb-2012-13-10-r91 (PMC3491419; doi:10.1186/gb-2012-13-10-r91)
Supplement: Additional file 7 — Table S3 - pathway enrichment in tissue modules. [file gb-2012-13-10-r91-S7.pdf]

Supplemental Table S3. Pathway Enrichment for Male and Female Rat Tissue Modules

Sex.Tissue / Pathway name	11 Male & Fem.Tissues	6 Male Tissues	Male modules									5 FemaleTissues	Female modules																			
			M.Turquoise	M.Blue	M.Brown	M.Yellow	M.Green	M.Red	M.Black	M.Pink	M.Magenta		F.Turquoise	F.Blue	F.Brown	F.Red	F.Green	F.Black	F.Pink	F.Magenta	F.Purple	F.Greenyellow	F.Tan	F.Salmon	F.Cyan	F.Midnightblue	F.Lightcyan	F.Grey60	F.Lightgreen	F.Lightyellow	F.Yellow	F.Coral
**Number of genes in input list**	**4059**	**3046**	**957**	**363**	**525**	**426**	**48**	**7**	**24**	**26**	**17**	**1298**	**148**	**137**	**78**	**51**	**33**	**28**	**31**	**30**	**20**	**25**	**17**	**20**	**18**	**21**	**16**	**19**	**13**	**14**	**22**	**10**
**Total Number of Pathways**	**230**	**224**	**200**	**65**	**179**	**73**	**17**	**5**	**2**	**12**	**3**	**191**	**48**	**32**	**44**	**61**	**10**	**11**	**7**	**19**	**4**	**8**	**1**	**10**	**2**	**7**	**8**	**7**	**3**	**7**	**2**	**2**
Pathways in cancer	45	32	14	1	14	1	1					17	2	2		1				1		1		2								
Protein processing in endoplasmic reticulum	39	38	26	2	9	1						3	1		1																	
HTLV-I infection	39	31	17	2	4	1						10	3		1	1																
RNA transport	39	31	15	3	7	2						11	1	1	2																1	
Transcriptional misregulation in cancers	31	26	7	1	13	3						12	3	1		2																1
Herpes simplex infection	30	23	7		7	2						9		1	2	1																
Lysosome	29	27	11	3	5	2						5	1			3																
Ribosome	29	27		2	1	23						4		1								2					1					
Endocytosis	29	26	11		9	3	1					5		1		2																
Phagosome	28	27	10		9	1						7			1	1				1												
MAPK signaling pathway	28	21	7		6	2						8	1	1		1																
Spliceosome	27	17	3	3	1	5				2	1	12		4	2	1	1															
Regulation of actin cytoskeleton	26	24	8		7	2						5	1	1	1	1																
Alzheimer's disease	26	22	6	3	4	8	1					5			1			1														
Huntington's disease	26	22	2	5	3	9	1					5	1		2			1														
Purine metabolism	26	22	5	4	7	4						7			1			1												1		
Focal adhesion	26	21	7		9	2	1					9		2		2						1		1								
Chemokine signaling pathway	24	23	12	2	2	3						4				2																
Pyrimidine metabolism	24	20	6	4	6	3						6			1					1										1		
Tuberculosis	24	20	6		7							9	1		1	3																
Influenza A	23	21	8	1	4	2				1		5	1			1				1												
Oxidative phosphorylation	23	20	5	3	3	8	1					5			2	1		1														
Leukocyte transendothelial migration	22	18	9	1	7	1						6	1			1																
Cytokine-cytokine receptor interaction	21	19	6	1	8							4	1									1										
Osteoclast differentiation	21	18	8		6							5	1			1																
Cell adhesion molecules (CAMs)	20	14	6		7	1						7	2			1																
Insulin signaling pathway	19	13	9	1	2	1						6		1				1														1
mRNA surveillance pathway	19	13	6	2	1	2						8	2	1	2																1	
Ribosome biogenesis in eukaryotes	19	13	5	1	3	2						7	1																	2		
Wnt signaling pathway	18	14	5		4	2						4	2		1																	
Neuroactive ligand-receptor interaction	18	12	3	1	3	1						7	1							2	1							1				
RNA degradation	18	12	4	3	2	1					1	7		1	2																	
Tight junction	18	12	7		3	2						9	2	1		2																
Calcium signaling pathway	17	16	4	2	1	2		1				4				1				1							1					
Parkinson's disease	17	15	2	3	3	7	1					2			1			1														
Ubiquitin mediated proteolysis	17	12	11	1								7	2	1	1	1				1												
Measles	16	16	6	1	4					1		4	1	1		1																
Hepatitis C	16	11	7		2					1		6				2		1														
Cell cycle	16	10	10	1	1							4	1		1		1		1													
Phosphatidylinositol signaling system	16	10	8		1			1				5	1			1			1													
Fc gamma R-mediated phagocytosis	15	16	8		2	1																										
Natural killer cell mediated cytotoxicity	15	14	3	1	5	1						4	1			1				1												
Systemic lupus erythematosus	15	14	1	1	4	3						3	1																			
Dopaminergic synapse	15	12	7		3							3	1																			
Antigen processing and presentation	15	11	4		3	1						4	1		1																	
Small cell lung cancer	15	11	5		3	1	1					5				1								1								
Apoptosis	15	10	5		3							8	2			1																
Toxoplasmosis	15	10	6		3		1					6				1								1								
Rheumatoid arthritis	14	12	5		5							5		1	1	2																
Arrhythmogenic right ventricular cardiomyopathy (ARVC)	14	11	5		2	1						3																				
Jak-STAT signaling pathway	14	11	4		4							3	1	1																		
Chagas disease (American trypanosomiasis)	14	10	4		4							5				1																
Inositol phosphate metabolism	14	9	7	1	1			1				4	1			1			1													

Serotonergic synapse	14	8	2		3							6																				
N-Glycan biosynthesis	13	13	10		5					1																						
Dilated cardiomyopathy (DCM)	13	12	4			2						4																				
Adherens junction	13	11	7		4							5	1			1																
Glycerophospholipid metabolism	13	11	5		4	2						3			1						1											
T cell receptor signaling pathway	13	10	6		3	1						5		1		1																
Complement and coagulation cascades	13	8	2	1	5							4																				
Protein digestion and absorption	13	8	4		2	1						5																				
Adipocytokine signaling pathway	13	6	5		2							9				1		1										1				
Hematopoietic cell lineage	12	12	2		6							4															1					
Synaptic vesicle cycle	12	12	10		3							2			1	1																
Aminoacyl-tRNA biosynthesis	12	11	4		6		1					2			1																	
Amoebiasis	12	11	4		3		1					3				1								1								
Pertussis	12	11	4	1	3					1		3				1				1												
Salmonella infection	12	11	6		2	1						3				1				1												
Toll-like receptor signaling pathway	12	11	3	1	3					1		2				1																
Axon guidance	12	10	5		3	1						3																				
Bacterial invasion of epithelial cells	12	10	6		2	1						3		1		1																
Viral myocarditis	12	10	5		3							4		1															1			
Cardiac muscle contraction	12	9	1	1		3	1					4																				
Vascular smooth muscle contraction	12	9	2		2	1						5		1						1								1				
ECM-receptor interaction	12	8	1		4		1					5												1								
Hypertrophic cardiomyopathy (HCM)	12	8	3			1						5																				
GnRH signaling pathway	11	11	3	1	2							2				1																
Neurotrophin signaling pathway	11	11	6		1							1				1																
Circadian rhythm - mammal	11	9	3		4							5			1					1				1								
Cytosolic DNA-sensing pathway	11	9	3	1		1				1		4				1				1										1		
Mineral absorption	11	9	1		4	1						3	1										1									
Progesterone-mediated oocyte maturation	11	9	7									2			1			1														
Staphylococcus aureus infection	11	9	1		6							3																				
Chronic myeloid leukemia	11	7	6									5		1		1																
p53 signaling pathway	11	7	6	1	2							2	1																			
PPAR signaling pathway	11	5	3		2							8						1						1				1				
B cell receptor signaling pathway	10	10	5		1	1						2				1																
Leishmaniasis	10	10	3		5							2				1																
Bile secretion	10	9	6	1	1							2			1																	
Oocyte meiosis	10	9	6									2			1		1															
Acute myeloid leukemia	10	8	4		3							3				1																
Cholinergic synapse	10	8	3		2							4	1																			
mTOR signaling pathway	10	8	3	1	1							3		1								1										
Porphyrin and chlorophyll metabolism	10	8	2	1	4							3														1						
Prostate cancer	10	8	5		1							3	1			1																
Pancreatic cancer	10	7	5		2							5		1		1						1										
Basal transcription factors	10	6	1	1		1						4	1		1																	
Drug metabolism - other enzymes	10	5	1	1	1							5			1	1																
Malaria	10	5			2							7														4						
Fc epsilon RI signaling pathway	9	10	4		3							1																				
Gap junction	9	10	2		3							1				1																
Endocrine and other factor-regulated calcium reabsorption	9	9	3		1	1						1																				
Salivary secretion	9	9	4		1							2															1					
GABAergic synapse	9	8	4		1							3				1																
RNA polymerase	9	8	2	2	1	2						2																		1		
VEGF signaling pathway	9	8	3		1	1						2		1		1																
Glutamatergic synapse	9	7	4		1							3																				
Legionellosis	9	7	5	1	1							4				1																
Prion diseases	9	7	2		3							3																				
TGF-beta signaling pathway	9	6	2		5							4							1													
Cysteine and methionine metabolism	9	5	3		2							4	1			1					1											
Renal cell carcinoma	9	5	3		1							5		1						1		1		1								
Glutathione metabolism	8	8	5	1	1							1					1															
Proteasome	8	8	2	2	2	2																										
Amino sugar and nucleotide sugar metabolism	8	7	2		5	1						1							1													
Gastric acid secretion	8	7	3		1							2							1													
RIG-I-like receptor signaling pathway	8	7	3		1					1		2				1																
Notch signaling pathway	8	6	3		2						1	3			1		1															
Valine, leucine and isoleucine degradation	8	6			4							2			1		1															
Endometrial cancer	8	5	4		1							3	2																			

Glycosylphosphatidylinositol(GPI)-anchor biosynthesis	8	4	4		1							4			1												1					
Protein export	7	8	4		2	2																										
Butanoate metabolism	7	7	2		2																											
Glioma	7	7	4		1																											
Melanogenesis	7	7	3		1							1	1																			
Pancreatic secretion	7	7	4		1							2															1					
Collecting duct acid secretion	7	6	5		1							3			1	1																
Colorectal cancer	7	6	2		1							2	2																			
Aldosterone-regulated sodium reabsorption	7	5	3									1																				
Arachidonic acid metabolism	7	5	2	1	3							3														1						
Bladder cancer	7	5	3		4							3		1								1										
Hedgehog signaling pathway	7	5	1	1	2							2																				
Amyotrophic lateral sclerosis (ALS)	7	4	3	1								4								1												
Metabolism of xenobiotics by cytochrome P450	7	4	2		3							4					1							1								
Melanoma	6	6	4		2																											
Non-small cell lung cancer	6	6	5		1																											
Sphingolipid metabolism	6	6	2				1					1				1																
ErbB signaling pathway	6	5	3									2		1		1																
Regulation of autophagy	6	5	4									1																				
Steroid biosynthesis	6	5	5		2							1																	1			
Vasopressin-regulated water reabsorption	6	5	3		1							2	1																			
ABC transporters	6	4	3		1							2			1																	
Type I diabetes mellitus	6	4	1		1							2																				
Drug metabolism - cytochrome P450	6	3	2									4					1							1								
Other glycan degradation	5	5	3		1					1		2				1									1							
SNARE interactions in vesicular transport	5	5	1		1	2	1																									
NOD-like receptor signaling pathway	5	4	3		1							3				1				1												
Primary immunodeficiency	5	4	2		1							2																				
African trypanosomiasis	5	3	1									4														4						
Thyroid cancer	4	4	3		1							1																				
Glycolysis / Gluconeogenesis	4	3	3		1							1																				
Synthesis and degradation of ketone bodies	3	3			2																											

Only Pathways with 3 or more affected genes per signature list are shown
