# Supplementary material for: Epigenetic transgenerational inheritance of somatic transcriptomes and epigenetic control regions
Source: Genome Biol. 2012 Oct 3;13(10):R91. doi: 10.1186/gb-2012-13-10-r91 (PMC3491419; doi:10.1186/gb-2012-13-10-r91)
Supplement: Additional file 8 — Table S4 - epigenetic control regions and gene expression. [file gb-2012-13-10-r91-S8.pdf]

Table S4. Epigenetic Control Regions (ECR) and Tissue Specific Gene Expression

A. Female

ECR	Female Heart	Female Kidney	Female Liver	Ovary	Uterus
chr1-81.05	ENSRNOT00000057163	LOC687333 Sdccag1		Egln2 Ltbp4	
chr1-93.9	RGD1309326 Snrp70	Snrp70 Tbc1d17		Prr12	LOC499144
chr1-109.35	ENSRNOT00000053327	ENSRNOT00000052564 ENSRNOT00000053529 ENSRNOT00000053882 ENSRNOT00000053893 ENSRNOT00000054014			
chr1-159.65	Hbb MGC72973	Hbb MGC72973 Rrp8			
chr1-185.85	ENSRNOT00000053325	Bag3 Ccdc95 Tgfb1i1 Znf688			
chr1-206.4	Drap1 Mrpl49 Mtvr2	BC090353 RGD1560108		LOC100361915 Ccdc85b Ltbp3 Rela	Slc25a45
chr2-188.8	Gpr89	LOC100363361 FQ217306 Sv2a		Car14 Oaz3	Lingo4 Plekho1 Rbm8 Sf3b4
chr3-107.4	MGC105649 Ndufb4 RGD1562529	Slc28a2		Slc28a2	
chr3-112.8	Ciao1 ENSRNOT00000055921	Ap4e1 ENSRNOT00000055921 Trpm7		Anapc1	
chr3-7.2	Setx	LOC499770		ENSRNOT00000052447 Odf2	Setx
chr4-165.3	Klrb1c Klri1	MGC94282		Pzp	LOC690045
chr4-26.2	Cyp51	RGD1561472 Samd9l		Gng11	
chr5-142.1	ENSRNOT00000052635 Mtf1	LOC100366213 Macf1		Fhl3 Sf3a3	
chr5-151.75	Fusip1 RGD1359529 Smpdl3b Tmem57	RGD1359529	Sh3bgrl3	Tmem57	LOC689826
chr5-59.9	Gba2 Ncbp1 Zcchc7	Tpm2		Exosc3	10868796
chr6-125.35	Atxn3	Tc2n		Asb2 Cpsf2 Trip11	ENSRNOT00000011530
chr7-8.5	ENSRNOT00000048364 Sf3a2	Dot1l ENSRNOT00000053394 LOC100125368		Cirbp Mbrl Scamp4	
chr7-118.8	Rrp7a Ttll12	Cyp2d5 ENSRNOT00000039879 Tnrc6b		Cyp2d4v1 Cyp2d5	
chr7-48.35	10902292*	Bbs10 Nav3 RGD1561474 Zdhhc17		Phlda1	
chr10-13.6	Axin1	LOC287167 Luc7l		Hba-a2 Hba-a2 RGD1310922	Hba-a2 Hba-a2
chr10-55.7	Slc2a4	Alox15		Rnasek Zbtb4	Zfp3
chr12-19.65	Ap1s1 Mepce	Prkrip1		Agfg2 Serpine1	

chr13-85.75	Pcp4l1	Atp1a2 Cd48 Kcnj10 Slamf9		10769693 Pigm Uap1	Apoa2
chr15-3.45		Spetex-2A Spetex-2C Spetex-2D Spetex-2F Spetex-2G Spetex-2H			
chr16-17.3	Ccdc124 Ell Slc27a1	Cnot7 Tmem38a		Pgpep1	
chr19-24.55	Nacc1	Elmod2 Ier2		Nfix Zswim4	
chr20-2.75	ENSRNOT00000059561	ENSRNOT00000052790 RT1-Bb		Ppp1r10 Rpl13-ps1	Neu1 Zbtb22
chrX-39	RGD1561065	RGD1561065		10937568 Alas2 Gnl3l Smc1a	Alas2

B. Male

Cluster Name	Male Heart	Male Kidney	Male Liver	Testis	Seminal Vesicle	Prostate
chr1-78.4		Bloc1s3 Ceacam6 Pafah1b3 Trappc6a	Axl Qpctl	Bckdha Snrpd2 Znf574	Atp5sl Lipe	10705102 Clptm1 Ercc1 Gsk3a LOC686781 Pvrl2
chr1-81.6		Eid2 Polr2i Psmc4 RGD1562079 Sdccag1 Tmem147 Tyrobp Zfp36		LOC687295	Blvrb Gramd1a Zfp36	Sipa1l3 Aplp1 Gmfg Pld3 RGD1303117 Sipa1l3 Tyrobp Yif1b Zfp36
chr1-93.35		Aldh16a1 Irf3 Klk1l LOC494539 MGC93975 Pnkp Rras Snrp70		Napsa Siglec5	ENSRNOT00000052978 ENSRNOT00000053402	Atpbd3 Cd37 Med25 MGC93975 Myh14 Nomo1 Ptov1 RGD1564800 Sphk2 Trpm4
chr1-109.4		ENSRNOT00000052993 ENSRNOT00000054014			ENSRNOT00000052507 ENSRNOT00000052564 ENSRNOT00000052661 ENSRNOT00000053019 ENSRNOT00000053117 ENSRNOT00000053184 ENSRNOT00000053855 ENSRNOT00000053882 ENSRNOT00000053934 ENSRNOT00000053936 ENSRNOT00000053974	ENSRNOT00000052564 ENSRNOT00000053081 ENSRNOT00000053882

chr1-184.85		Bcl7c Coro1a		Cdipt ENSRNOT00000053325 Prr14 Xpo6 Xtp3tpa	Ccdc95 Eif3c	Tbc1d10b Rabep2 Rnf40
chr1-200.8		Lsp1 Mrpl23 Rnh1 Tspan4		Tmem80 Tspan4	Lsp1	Irf7 Lsp1 Tollip Tssc4
chr1-204.75	ENSRNOT00000052494 ENSRNOT00000053169 Ltbp3	Tmem223 B3gnt1 BC090353 Cox8a Cpsf7 Eef1g Eif1ad ENSRNOT00000052494 ENSRNOT00000053169 Frmd8 Mtvr2 Nxf1 Otub1 Ppfia1 RGD1566262 Tmem134 Unc93b1 Yif1	Chka Esrra Ms4a11 Rab3il1 Tmem109	LOC100361529 LOC100362601 LOC100363176 Tmem223 Aip Cpsf7 Dpf2 ENSRNOT00000053552 ENSRNOT00000054147 ENSRNOT00000069284 Ndufs8 Nxf1 Otub1 Pcnxl3 Ppfia1 Rbm14 Rpl35a Sf1 Slc29a2	Ms4a11 Ms4a6b Rela RGD1560108 Slc15a3	BC090353 Ccnd1 Cdc42bpg Cpt1a Ddb1 Ganab Ints5 Mark2 Ms4a11 Ms4a6a Ppfia1 Rce1 RGD1562657 RGD1566262 Rtn3 Slc3a2 Syvn1 Trpt1 Vps37c Zdhhc24
chr2-180.15	LOC685152	Dennd4b Lmna S100a6	Il6ra	Ints3 RGD1561797 Rps27 S100a6	Dap3 Krtcap2 RGD1564171	Ints3 Lenep Pygo2 RGD1303130 Sema4a Slc25a44
chr2-188.65	Ctsk	Setdb1 Hist2h3c2	Ctss Fcgr1a LOC690012 Prpf3 Txnip	LOC100363361 Lass2 mrpl9	Hist2h2bb Txnip	LOC100363361 Acp6 Fcgr1a Tars2 Vps72
chr20-9.25		Abcg1 ENSRNOT00000053196 ENSRNOT00000053957 Pcbp3 RGD1561557			Pdxk S100b	Lss RGD1309594 RGD1311257 RGD1561557 Slc37a1 Trappc10 Zfp280b
chr3-144.75		10851019 Rbm39 Scand1		Edem2 ENSRNOT00000069216	Ergic3 Ncoa6	Ergic3 Map1lc3a Ndrg3 Trpc4ap
chr4-75.4	10862453 Ccdc126	10862453 Tmem176a	Gpnmb	ENSRNOT00000053328 FQ223410	Pdia4 Repin1	10862453 Zfp212 Znf746
chr5-136.75		Ccdc17 GENSCAN00000021509 Kdm4a Zswim5		ENSRNOT00000052542 ENSRNOT00000056936 Mmachc	BC105775	Mmachc Ptprf RGD1308616
chr5-163.75		ENSRNOT00000054574 Fbxo6 LOC500584 LOC691261 LOC691431 Spsb1			LOC691280 ENSRNOT00000053105 Rpl35a	Tmem201 Clstn1 Dffa Gpr157 Kif1b Mad2l2 Mthfr Ube4b Ubiad1
chr6-132.3		Mir667 Mir341				LOC100363014 AB014877 AB014878 AB014879 AB014881 ENSRNOT00000053070 ENSRNOT00000053573 ENSRNOT00000053832 ENSRNOT000000

						54021 ENSRNOT00000054054 ENSRNOT00000054133 FQ212998 FQ213989 LOC690965 Slc25a29 Tecpr2 Wars
chr6-137.4		FQ218314		Nudt14	LOC691616	Adam6 ENSRNOT00000048037 IgG-2a IgG-2a Ighg Jag2 LOC500726 LOC678701 LOC691616 Mta1
chr7-112.3		Bop1 Exosc4 Gsdmd Mfsd3 Mpst RGD1359378 Tst	Bop1 Csf2rb Ly6c Ly6e	Tsta3	Scrib	Bop1 ENSRNOT00000056375 Ly6c Ncf4 Plec1 rCG_59505 RGD1309808 Scrib Siahbp1
chr7-8.5		Dapk3 Dot1l Fam108a1 Lsm7 Mbd3 Mknk2 RGD1307067 RGD1359127 Rps15 Timm13		Midn	Dot1l LOC314605 Mrpl54 RGD1359127 Wdr18	Abca7 Ap3d1 Eef2 Mbrl Med16 Ncln Scamp4 Sppl2b Zfp709l1
chr8-112.8		Impdh2 Klhl18	Ip6k2 Plxnb1 Pthr1	Map4 Nme6 Slc38a3 Spink8	Amigo3	Nbeal2 Ptpn23 Scap Slc26a6
chr8-45.4		10916853 Dpagt1 Oaf	Mpzl2 Tmem24	Atp5l Hmbs	Hyou1	Dpagt1 ENSRNOT00000053557 Hyou1 Pvrl1 Rnf26 Tmem24 Vps11
chr9-8.35		LOC100360664 Med20 Polr1c Tomm6	Taf8	Med20	LOC363188	Foxp4 LOC363188 Srf
chr9-72.25		Aamp Des Plcd4 Stk36		Rqcd1	ENSRNOT00000057365 Stk36	Aamp Pnkd Rqcd1 Slc11a1 Stk11ip
chr10-104.15		Cd300le ENSRNOT00000069295 Mrpl38 RGD1311078	RGD1561778	Mrps7 Rps18 Sec14l1	Cygb Slc25a19 Slc9a3r1	Gprc5c Itgb4 RGD1306284 RGD1307394 RGD1561778 Slc9a3r1 Ube2o
chr10-12.15		Srrm2 Tnfrsf12a		Nme3	Mapk8ip3 Rnps1	Abca3 Atp6v0c ENSRNOT00000060779 Jmjd8 Mare Mpg Rnps1 Spsb3 Tbl3 Wdr24 Znf598
chr10-38.9		Atox1 LOC303140 P4ha2 Rpl13	Irf1	Hint1	P4ha2	Cdc42se2 ENSRNOT00000054033 Slc36a1
chr10-54.75	Per1	Per1 Pfn1 Tmem88	Per1	Pelp1 Slc25a35	Cd68 Plscr3 Rpain	Fxr2 Pelp1 Tmem102 Tnk1

chr10-62.45		ENSRNOT00000041305 ENSRNOT00000054438	Tmem97	Dbil5 Prpf8 RGD1566149 Slc43a2	Rab34	Dph1 Pigs Pitpna Vps53
chr10-86.55	Klhl11	Ccr7 ENSRNOT00000045189 ENSRNOT00000048840 Krt40 LOC680286 Vat1	Arf4l Rara Zpbp2	BC099108 Hspb9 Rnd2 Rpl35a	Eif1 Nr1d1 Zpbp2	Atp6v0a1 Becn1 Eif1 Fbxl20 Gcn5l2 Jup Krtap9-1 Mpp2 Nags Nkiras2 RGD1311493
chr10-9.85		Hmox2 LOC363543 Rbm25			Btbd12 Magmas	LOC100362008 Anks3 Mgrn1 N-pac Ubn1
chr12-15.65		Mir339 Asmtl Gpr146 Mblac1 RGD1311660		LOC288526	Rpl31	Cops6 Taf6 Unc84a
chr12-21.3		Dnajc30 Por Rhbdd2 Wbscr27		Polr2j	10761285 Bcl7b	Abhd11 Clip2 Eif4h Rhbdd2
chr12-31.75		Ccdc62	Ccdc92	Vps37b Zfoc1	Ubc	Atp6v0a2 Eif2b1 Hip1r Pitpnm2 Setd8-ps1 Ubc
chr12-41	Gltp	Gltp RGD1311899	Ficd	Acads Pop5 Pxn Sppl3	Ficd Gcn1l1	Acacb Mlec Oasl2 Pxn Rnf10 Sart3 Sppl3
chr13-85.65		ENSRNOT00000053447 FQ230857 Pea15a	10769693 FQ230857	Fcer1g Ppox Tagln2	ENSRNOT00000053447 Tomm40b Usp21	Cd84 F11r Fcgr2b
chr14-36.1	Nfxl1 ENSRNOT00000002991	Nfxl1 RGD1310958	ENSRNOT00000002991	RGD1311309	Nfxl1 RGD1359201	Ociad1
chr14-82.2		Atp5e Selm	Depdc5	Atp5e Depdc5 LOC685322 Sf3a1	RGD1305469 Dusp18 RGD1560636	Maea Tmem129 Ywhah
chr15-31.9		Mcpt2 Rabggta Thtpa Y09171	Homez	Homez	Dhrs1 Psmb5	Lrp10 Mcpt1 Mcpt2 Rabggta RGD1565222 Tinf2 Zfhx2
chr16-17.45		ENSRNOT00000034446 FQ211569 Ifi30 MGC72612 Mrpl34	Uba52	Ell	ENSRNOT00000051889 Slc25a42 Use1	Atp13a1 Bst2 Gatad2a Jund MGC72612 Pik3r2 Rab3a Rent1 RGD1308759 Slc35e1
chr19-23.6	Dnajb1 RGD1564739	Gadd45gip1 Mri1 Prdx2	Cd97	Prdx2 Rad23a	Gcdh Hook2	Gpsn2 Nfix RGD1308221 Tnpo2
chr19-34.95		LOC690214 Nqo1 Pskh1 Rps12		Sntb2	Cirh1a Nfatc3 Tmem208	Cog8 Ddx28 Pskh1
chr20-2		H2-T23 Hla-dma Neu1 Nudt3 Tapbp Tubb5	RGD1303066 Taf11	Agpat1 Bat1 Brpf3 ENSRNOT00000052790 ENSRNOT00000052797 RGD1306917 Rnf8 Rps18	Spdef Trim39	RGD1307264 Abcf1 Atf6b Bat2 Cdkn1a ENSRNOT00000036804 Ftsjd2 H2-T24 Itpr3 Mapk13 Neu1 Phf1 Psmb8 Tbc1d22b U15425 Znf76

Genes expressed within ECR per tissue presented. * - if EST didn't have any other ID numbers, Affymetrix RatGene 1.0 ST probeset number was used to enlist it into the table.
