# Supplementary material for: Epigenetic transgenerational inheritance of somatic transcriptomes and epigenetic control regions
Source: Genome Biol. 2012 Oct 3;13(10):R91. doi: 10.1186/gb-2012-13-10-r91 (PMC3491419; doi:10.1186/gb-2012-13-10-r91)
Supplement: Additional file 9 — Table S5 - lncRNA and epigenetic control regions. [file gb-2012-13-10-r91-S9.pdf]

Supplemental Table S5.

Long non-coding RNA (lncRNA) Association with ECR

lncRNA Symbol	lnc RNA Genome Location	ECR
antiPeg11	rn4 chr6:134,182,037-134,187,063	chr6:132.3
B2 SINE RNA	N/A	
BC1	N/A	
Beta-MHC antisense transcripts	N/A	
Bsr	rn4 chr6:134,198,113-134,328,710	chr6:132.3
Evf2	rn4 chr4:31,722,073-31,770,275	NA
GAS5	rn4 chr13:76,594,781-76,598,102	NA
H19	rn4 chr1:202,822,926-202,825,601	chr1:200.8
HOTAIR	rn4 chr7:141,707,460-141,711,096	NA
HOTAIRM1	rn4 chr4:80,458,677-80,462,167	NA
Khps1a	rn4 chr10:106,639,811-106,641,100	chr10:104.15
MEG9	rn4 chr6:134,410,685-134,431,542	chr6:132.3
NEAT1	rn4 chr1:208,476,553-208,479,839	chr1:206.4
PINC	rn4 chr9:72,706,829-72,723,152	chr9:72.25
RNCR3	rn4 chr15:43,941,969-43,947,942	NA
SNHG6	rn4 chr5:8,840,469-8,842,569	NA
SRA	rn4 chr18:29,306,556-29,309,970	NA
TUG1	rn4 chr14:84,283,176-84,293,598	chr14:82.2
Zfas1	rn4 chr3:158,214,340-158,216,912	NA
Zfhx2as	rn4 chr15:33,178,036-33,180,752	chr15:31.9
